# Oxy-Polybrominated Diphenyl Ethers from the Indonesian Marine Sponge, *Lamellodysidea herbacea*: X-ray, SAR, and Computational Studies

**DOI:** 10.3390/molecules26216328

**Published:** 2021-10-20

**Authors:** Novriyandi Hanif, Trianda Ayuning Tyas, Lestari Hidayati, Fabians Faisal Dinelsa, Dian Provita, Nyimas Ratna Kinnary, Fauzi Muhamad Prasetiawan, Gibral Abdul Khalik, Zaki Mubarok, Dudi Tohir, Andi Setiawan, Muhamad Farid, Viqqi Kurnianda, Anggia Murni, Nicole J. de Voogd, Junichi Tanaka

**Affiliations:** 1 Department of Chemistry, Faculty of Mathematics and Natural Sciences, IPB University, Bogor 16680, Indonesia; triandaa.tyas@gmail.com (T.A.T.); lestarihidayati99@gmail.com (L.H.); ffdinelsa@gmail.com (F.F.D.); dianprovita6@gmail.com (D.P.); ratna.kinnary@gmail.com (N.R.K.); fauzimuhamad.p@gmail.com (F.M.P.); gibralabdul98@gmail.com (G.A.K.); zakimubarok359@gmail.com (Z.M.); dudi.tohir@yahoo.co.id (D.T.); faridsidik64@gmail.com (M.F.); 2 Department of Chemistry, Biology, and Marine Science, University of the Ryukyus, Nishihara 903-0213, Okinawa, Japan; viqqikurnianda@yahoo.co.id (V.K.); jtanaka@sci.u-ryukyu.ac.jp (J.T.); 3 Department of Chemistry, Lampung University, Bandar Lampung 35145, Indonesia; asetiawan0922@gmail.com; 4 Tropical Biopharmaca Research Center, IPB University, Bogor 16128, Indonesia; anggia_murni@apps.ipb.ac.id; 5 Institute of Environmental Sciences (CML) Leiden University, P.O. Box 9518, 2300 RA Leiden, The Netherlands; nicole.devoogd@naturalis.nl; 6 Naturalis Biodiversity Center, P.O. Box 9517, 2300 RA Leiden, The Netherlands

**Keywords:** antibacterial activity, HEK 293T, PBDE, DFT calculations, NMR, X-ray

## Abstract

Polybrominated diphenyl ether (PBDE) compounds, derived from marine organisms, originate from symbiosis between marine sponges and cyanobacteria or bacteria. PBDEs have broad biological spectra; therefore, we analyzed structure and activity relationships of PBDEs to determine their potential as anticancer or antibacterial lead structures, through reactions and computational studies. Six known PBDEs (**1**–**6**) were isolated from the sponge, *Lamellodysdiea herbacea*; ^13^C NMR data for compound **6** are reported for the first time and their assignments are confirmed by their theoretical ^13^C NMR chemical shifts (RMSE < 4.0 ppm). Methylation and acetylation of **1** (2, 3, 4, 5-tetrabromo-6-(3′, 5′-dibromo-2′-hydroxyphenoxy) phenol) at the phenol functional group gave seven molecules (**7**–**13**), of which **10**, **12**, and **13** were new. New crystal structures for **8** and **9** are also reported. Debromination carried out on **1** produced nine compounds (**1**, **2**, **14**, **16**–**18**, **20**, **23,** and **26**) of which **18** was new. Debromination product **16** showed a significant IC_50_ 8.65 ± 1.11; 8.11 ± 1.43 µM against human embryonic kidney (HEK293T) cells. Compounds **1** and **16** exhibited antibacterial activity against Gram-positive *Staphylococcus aureus* and Gram-negative *Klebsiella pneumoniae* with MID 0.078 µg/disk. The number of four bromine atoms and two phenol functional groups are important for antibacterial activity (*S. aureus* and *K. pneumoniae*) and cytotoxicity (HEK293T). The result was supported by analysis of frontier molecular orbitals (FMOs). We also propose possible products of acetylation and debromination using analysis of FMOs and electrostatic charges and we confirm the experimental result.

## 1. Introduction

Natural marine polybrominated diphenyl ethers (PBDEs) are promising medicinal lead structures for anticancer [1,2] and antibacterial [3] drugs. The latter can be rationalized by the structural resemblance to the man-made antibacterial, triclosan (2-(2′,4′-dicholorophenoxy)-5-chlorophenol). Structures of natural marine polybrominated products are derived from radical coupling of bromophenols catalyzed by a decarboxylative-halogenation enzyme [4]. These structures can be classified into six types, characterized by the presence of 2-bromophenyl (7.9%), 4-bromophenyl (3.2%), 2,4-dibromophenyl (31.7%), 2-hydroxy-5-bromophenyl (3.2%), 2-hydroxy-3,5-dibromophenyl (36.5%), and 2-hydroxy-4,6-dibromophenyl moieties (1.6%), and other PBDEs, including dioxin, benzofuran type (15.9%). Therefore, over 60 natural marine PBDEs based upon these major frameworks have been discovered since 1969. Seven of them have been found in Indonesia [5]. In addition, the unique structure of PBDEs, containing the H/C <1 (aromatic system), and the presence of halogen atoms are challenging for structure determination and correct assignments of ^13^C NMR chemical shifts. Currently, great improvements have made density functional theory (DFT)-based natural product structure determination a powerful method to provide accurate predictions and to evaluate ^13^C chemical shifts of target molecules possessing RMSE (root mean square error) <4.0 ppm [6]. This method has been used to accurately evaluate conformationally flexible moieties [6] and structures with high degree of unsaturation and substitution [6,7]. In general, the key features of computational NMR structure determination consist of conformational searches, DFT structure optimization, DFT energy calculations, NMR shift calculations, and statistical decisions. DFT calculations are also used to provide information about reactivity and kinetic stability of molecules, which may help us to understand the relationship between structures and their activities, as well as their quantum chemical parameters and properties, including frontier molecular orbitals (FMOs) and electronic charge distributions [8,9].

The distribution of natural marine PBDEs includes six phyla, of which the Porifera is the richest source. These molecules are most frequently found in the family *Dysideidae*, especially in *Lamellodysidea herbacea* and *Dysidea granulosa*, which show different chemotaxonomic markers. Initial work on biosynthesis of PBDEs showed that they localize to sponge tissues inhabited by the symbiotic cyanobacterium, *Oscillatoria spongelliae* [10]. Recently, it was suggested that marine polybrominated diphenyl ethers are synthesized by symbiotic marine cyanobacteria (*Hormoscilla spongeliae* or *Prochloron* sp.) or by marine bacteria (*Pseudoaltromonas* spp.) [11,12]. Despite the interesting discussion of PBDE origins in marine organisms, we re-investigated PBDEs from the abundant marine sponge, *L. herbacea* collected from Indonesian waters, to determine their therapeutic potential as anticancer or antibacterial drugs, by chemical reactions and computational studies. The Gram-positive, *Staphyloccoccus aureus* and Gram-negative *Klebisella pneumoniae*, are two important pathogenic targets for searching new antibiotics known as ESKAPE pathogens (*Enterococcus faecium*, *Staphylococcus aureus*, *Klebsiella pneumoniae*, *Acinetobacter baumanii*, *Pseudomonas aeruginosa* and *Enterobacter* species [13]. Moreover, new antibiotic scaffolds for Gram-negative pathogens have been extremely rare in recent decades.

**Figure 1:**
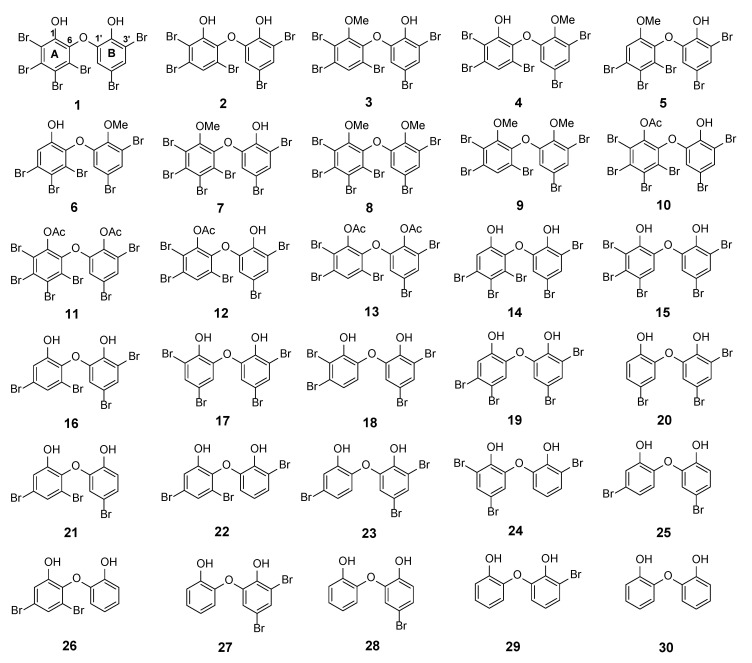
PBDE structures (**1**–**6**) from *Lamellodysidea herbacea* isolated in the present work via methylation (**7**–**9**), acetylation (**10**–**13**), and debromination (**1**, **2**, **14**, **16**–**18**, **20**, **23**, **26**). Compound **30** was commercially available.

The major PBDE **1** was isolated in gram quantities, having the largest number of substituted bromine atoms in two phenol rings. Its structure was determined with X-ray analysis [14]. PBDE **1** is a signature compound of *L. herbacea* collected in Indonesia and is frequently found in high quantities. We observed that the biological activity of PBDE molecules depends on two functional characteristics: The presence of phenol and the number and position of bromine atoms [14,15]. Starting with **1** (2, 3, 4, 5-tetrabromo-6-(3ʹ, 5ʹ-dibromo-2ʹ-hydroxyphenoxy) phenol), debromination via the reversibility of electrophilic aromatic bromination with regioselective debromination [16,17] may be a good way to synthesize various PBDE molecules with a lower number and a variety of positions of bromine atoms, in addition to isolation of other analogues and modifications of phenolic functional groups by methylation and acetylation. With the foregoing background, this article describes isolation of natural PBDE analogues (**1**–**6**) from the sponge, *L. herbacea* including evaluation of their ^13^C NMR chemical shifts using an advanced computational method [6]. It also reports structural modifications involving transformation of phenolic functional groups by methylation and acetylation to give seven molecules (**7**–**13**) and an interesting debromination in the presence of a bromine scavenger to give nine additional molecules (**1**, **2**, **14**, **16**–**18**, **20**, **23**, **26**) (Figure 1). We propose the calculated ^13^C NMR data of 30 PBDEs and evaluate their RMSEs for which we have experimental data. In regard to the exploration reaction, we also found that acetylation occurred in the presence of Ac_2_O and sonication without a catalyst and without solvent at room temperature for 2–3 h. Two compounds (**8** and **9**) have new crystal structures, whereas compounds **10**, **12**, and **13** are entirely new structures. Compound **18** is a new derivative obtained via debromination. One unpublished ^13^C NMR of **6** is also reported and secured its assignment by its calculated ^13^C NMR chemical shifts. This article also describes structure-activity relationships of marine polybrominated natural products against human embryonic kidney (HEK293T) cells and Gram-positive *Staphylococcus aureus*, as well as the Gram-negative bacterium *Klebsiella pneumoniae*. Computational studies further support their activities and account for acetylation and debromination.

## 2. Results and Discussion

**Figure 2:**
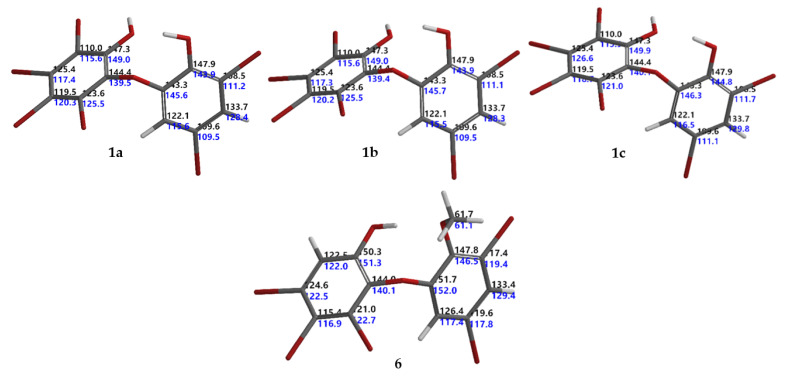
Calculated and experimental ^13^C NMR chemical shifts for **1a**, **1b**, **1c**, and **6** corresponding to their RMSEs 4.3, 4.4, 3.5, and 3.2 ppm, respectively. Black indicates calculated, while blue denotes experimental ^13^C NMR chemical shifts.

Six known O-PBDEs (**1**–**6**) have been isolated from the Indonesian marine sponge, *L*. *herbacea*, collected at Ujung Kulon. MS and ^1^H NMR spectra of **1**−**6** were in good agreement with previously reported data [14,15,18,19,20]. Compound **6** was first isolated by Fu et al., in 1995 [20]. To the best of our knowledge, ^13^C NMR data for compound **6** have not been reported previously. This work also gave us an opportunity to assign the ^13^C NMR for compound **6** (Table 1). Complete ^13^C NMR data of **6** (HREIMS *m/z* 605.6311 [M]^+^, C_13_H_7_O_3_^79^Br_5_, Δ −0.0001 mmu) were derived with the aid of DEPT, HMQC, and HMBC experiments and analogies from the literature. The HMBC experiment showed correlations with H2/C1,3,4,6; H4′/C2′, 3′, 5′, 6′; H6′/C1′,2′, 3′, 5′ and –OMe/C2′ leading to the order of the ^13^C data of **6** (Table 1). Moreover, the assignment was secured by the protocol of Hehre et al. [6] to give RMSE < 4.0 ppm (RMSE obtained was 3.2 ppm; RMSE for aromatic compounds were around 3.2 [6]). Intriguing with the RMSE of the isolated natural products, we conducted a set of experiments in order to obtain theoretical ^13^C NMR chemical shifts of **1**–**5** with six steps: (1) conformational search using the MMFF molecular mechanics model; (2) calculation of equilibrium geometries using the HF/3-21G model; (3) calculation of energies using the ωB97X-D/6-31G* density functional model; (4) calculation of equilibrium geometries using the ωB97X-D/6-31G* density functional model; (5) calculation of energies of using the ωB97X-V/6-311+G(2df,2p)(6-311G*) density functional model; and (6) calculation of ^13^C NMR chemical shifts using the ωB97X-D/6-31G* density functional model, correction of ^13^C NMR chemical shifts based on the empirical parameters, and correction ^13^C NMR chemical shifts based on the Boltzmann weight obtained in step 5. We found that compounds **2**–**6** gave RMSEs <4.0 ppm, except for **1** (RMSE >4.0 ppm, for **1a** 4.3 ppm [18], for **1b** 4.4 ppm [21] in DMSO-*d*_6_). These values suggested a possible error in the ^13^C chemical assignments. The error could be in an assignment of ^13^C chemical shifts in ring A (lack of H atoms). Based on the calculated ^13^C chemical shifts, we propose an RMSE for **1** as 3.5 ppm as in **1c** (Me_2_CO-*d*_6_). The assignment of ^13^C chemical shifts for **1** and **6** can be seen in Figure 2.

In order to see the effect of phenol in our assays, seven-modified phenolic compounds (**7**–**13**) were prepared by methylation (**7**–**9**) and acetylation (**10**–**13**). Two new crystal structures (**8** and **9**) were obtained from a methylation reaction using TMSCHN_2_ with three new synthetic PBDE derivatives (**10**, **12**, **13**) from an acetylation reaction using green chemistry.

Compound **8** (C_14_H_8_Br_6_O_3_) is 2,3,4,5-tetrabromo-6-(3′,5′-dibromo-2′-methoxyphenoxy) anisole, which has torsion angle *φ*_1_ = −8.6° (C6′– C1′–O–C6); *φ*_2_ = −82.3° (C1′–O–C6–C1), while the ether bond and dihedral angle between two aromatics are 116.9 (4)° and 86.3°, respectively. Compound **8** was crystallized in the triclinic space group P-1 (*Z* = 2) using CHCl_3_−MeCN (3:2).

**Figure 3:**
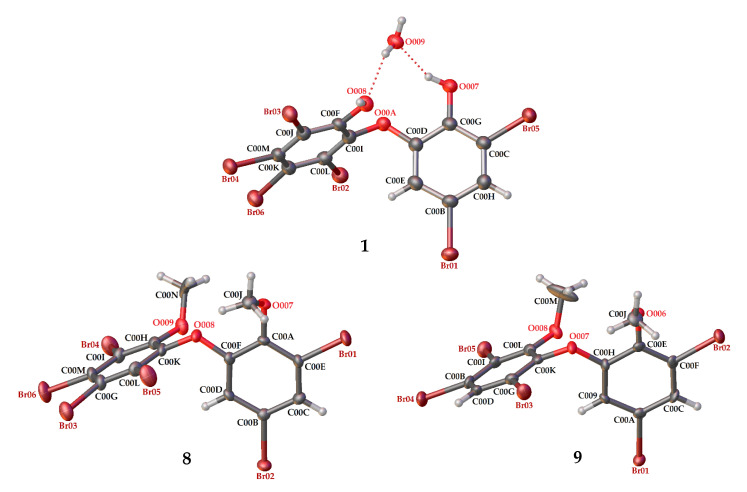
Crystal structures of **1**, **8** and **9** with displacement ellipsoids drawn at the 50% probability level. The crystal structure of **1** is presented to compare the material for structure modification with two phenolic groups and debromination.

**Figure 4:**
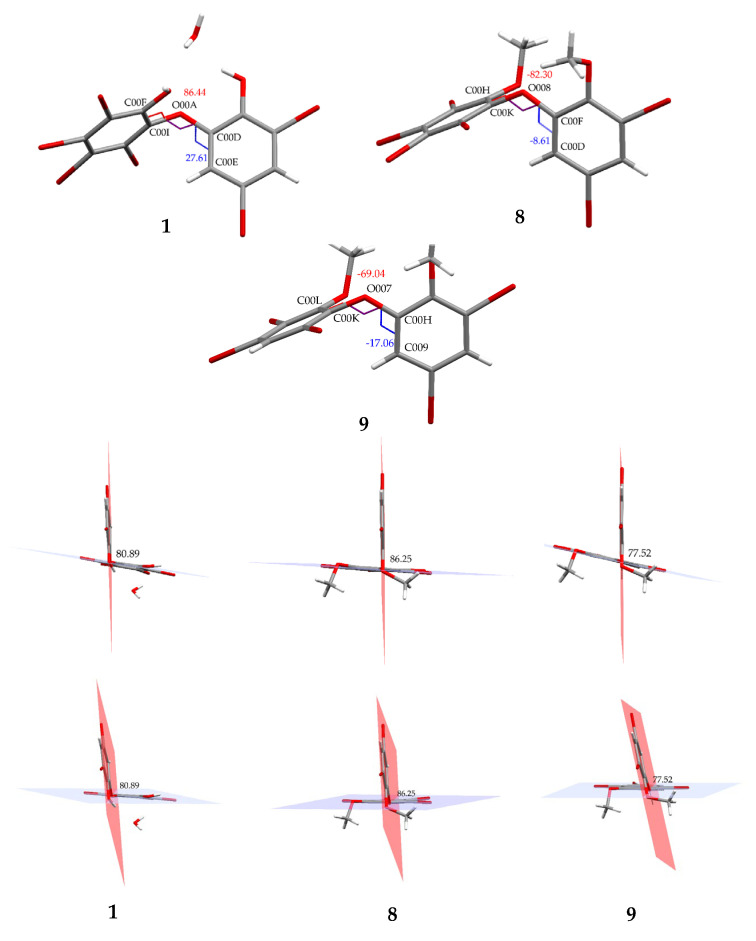
Torsion and dihedral angles of **1**, **8**, and **9**.

**Figure 5:**
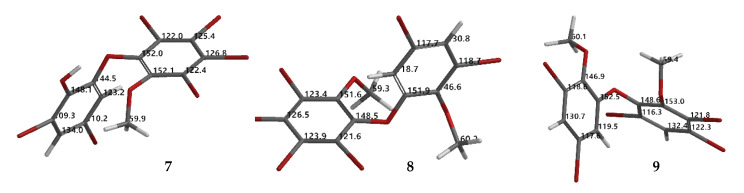
Proposed calculated ^13^C NMR chemical shifts of **7**–**9** using the NMR chemical shift calculation protocol.

Compound **9** (C_14_H_9_Br_5_O_3_) was identified as 2,3,5-tribromo-6-(3′,5′-dibromo-2′-methoxyphenoxy) anisole after X-ray analysis. The torsion angle of **9** is *φ*_1_ = −17.1° (C6′– C1′–O–C6); *φ*_2_ = −69.0° (C1′–O–C6–C1), whereas the rest are 117.3 (6)° and 77.5°. Compound **9** was crystallized as orthorhombic in the space group Pca21 using CHCl_3_−Me_2_CO−MeCN (1:1:1). Crystal structures of **1**, **8** and **9** are depicted in Figure 3 and Figure 4, while theoretical ^13^C NMR chemical shifts of **7**–**9** are shown in Figure 5. The RMSE of **7** showed 3.8 ppm after comparing with the literature data [19].

We initially screened conditions for acetylation and found that **1** reacts with Ac_2_O under sonication for 1 h without DMAP and Et_3_N at room temperature. Conversion of **1** to **11** was estimated at >95% using TLC. A larger amount of **1** was used under the above conditions to give acetylation products (**10**–**13**) with slightly longer time (2–3 h). To date, acetylation with acyl halides or acid anhydrides has been reported using solvents and catalysts at room temperature or higher (>40 °C). Acetylation methods have been reported without catalysts at high solvent temperatures (60–70 °C) or with a variety of catalysts from room temperature to 110 °C [22]. With regard to PBDE molecules, only **11** has been obtained from acetylation using Ac_2_O with pyridine at room temperature for 24 h [23]. Therefore, our method is new and is consistent with green chemistry concepts.

**Figure 6:**
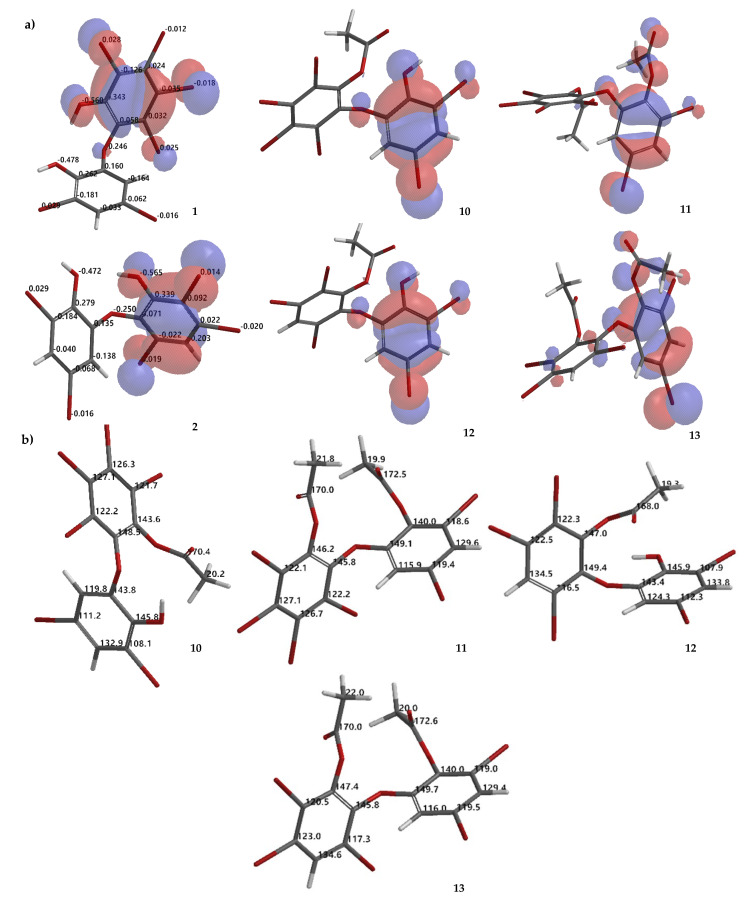
Molecular orbital analysis (HOMO) of **1**, **2**, **10**–**13** calculated using DFT ωB97X-D/6–311 + G(2d,p) along with analysis of electrostatic charges of **1** and **2** (**a**). Proposed calculated ^13^C NMR chemical shifts of **10**–**13** using the NMR chemical shift calculation protocol (**b**).

Compound **10** is a partial acetylation product (*R_f_* 0.23 Hex/EtOAc 8:1, n-silica, UV λ 254 nm) of **1,** based on its HRESIMS [M + H]^+^ 716.5404 (C_14_H_7_O_4_^79^Br_4_^81^Br_2_, Δ 0 mmu). ^1^H NMR data confirmed the presence of one -OAc group by observing *δ* (ppm) 2.47 in CDCl_3_ or 2.40 in Me_2_CO-*d*_6_. Because ring A is fully substituted, no HMBC correlation can be observed. The -OAc group can be placed on ring A by assuming that ring A is more reactive than ring B, on the basis of molecular orbital analysis of **1** with DFT dispersion correction level of theory and a triple-ζ-basis set (Figure 6a). Moreover, analysis of the electrostatic charge of **1** also justified that the -OH group in ring A is more electronegative (−0.560) than that of ring B (−0.478) (Figure 6a). Therefore, the first acetylation is more likely to proceed in ring A. In addition, comparison of **10** with **3** and **7** containing an anisole group on ring A showed that the ^1^H chemical shift is more upfield than ring B. Compound **10** was revealed as 2,3,4,5-tetrabromo-6-(3′,5′-dibromo-2′-hydroxyphenoxy) phenyl acetate.

Compound **11** is 2,3,4,5-tetrabromo-6-(3′,5′-dibromo-2′-acetoxyphenoxy) phenyl acetate, possessing *R_f_* 0.52 (Hex/EtOAc 8:1, n-silica, UV λ 254 nm) and HRESIMS [M + Na]^+^ 780.5329 (C_16_H_8_O_5_^79^Br_4_^81^Br_2_Na, Δ −0.0002 mmu) and after comparing its ^1^H NMR data with the literature [23]. The ^1^H chemical shift of the-OAc group in ring A could be assigned more upfield by comparing these data with the ^1^H NMR of methylated products, as in **8** and **9**. Therefore, *δ* 2.24 could be placed on ring A, while *δ* 2.36 (Me_2_CO-*d*_6_) could be assigned on ring B.

Compound **12** (*R_f_* 0.34 Hex/EtOAc 8:1, n-silica, UV λ 254 nm) is a mono-acetylated product of **2,** evidenced by HRESIMS [M + H]^+^ 640.6278 (C_14_H_8_O_4_^79^Br_2_^81^Br_3_, Δ 0 mmu). The ^1^H chemical shift of the -OAc was observed at *δ* (ppm) 2.47 in CDCl_3_ or 2.40 in Me_2_CO-*d*_6_. The same situation as **10** was also observed for **12**. The -OAc group could be attached to ring A. Highest occupied molecular orbital (HOMO) analysis **2** showed that ring A is more reactive than ring B which is justified by electrostatic charge analysis of the -OH group in ring A (−0.565) and in ring B (−0.472) (Figure 6a). Compound **12** was confirmed as 2,3,5-tribromo-6-(3′, 5′-dibromo-2′-hydroxyphenoxy) phenyl acetate.

Compound **13** (*R_f_* 0.51 Hex/EtOAc 8:1, n-silica, UV λ 254 nm) is a completely acetylated product of **2**. Two attached -OAc groups were verified with HRESIMS [M + Na]^+^ 704.6226 (C_16_H_9_O_5_^79^Br_2_^81^Br_3_Na, Δ 0.0023 mmu). ^1^H chemical shifts of two -OAc groups could be observed at *δ* (ppm) 2.25 and 2.36 in CDCl_3_ or 2.24 and 2.36 in Me_2_CO-*d*_6_. The -OAc group on ring A could be *δ* 2.24, while the other -OAc could assigned to *δ* 2.36 (in Me_2_CO-*d*_6_). Compound **13** is determined as 2,3,5-tribromo-6-(3′,5′-dibromo-2′-acetoxyphenoxy) phenyl acetate. To complete the characterization of acetylation products **10**–**13**, theoretical ^13^C NMR chemical shifts of the compounds are proposed as in Figure 6b.

Next, we examined the effect of lower bromine atoms on two phenol rings by employing debromination. Compound **1,** isolated in gram quantities, and possessing six bromine atoms on two phenol rings, was subjected to refluxing using HBr and Na_2_SO_3_ as scavengers of bromine in the presence of acetic acid. Compounds (**1**, **2**, **14**, **16**–**18**, **20**, **23**, **26**) were isolated and confirmed from the reaction. MS and ^1^H NMR spectra of compounds (**1**, **2**, **16**, **17**, **20**, **23, 26**) were in a good agreement with previous reports [14,15].

**Figure 7:**
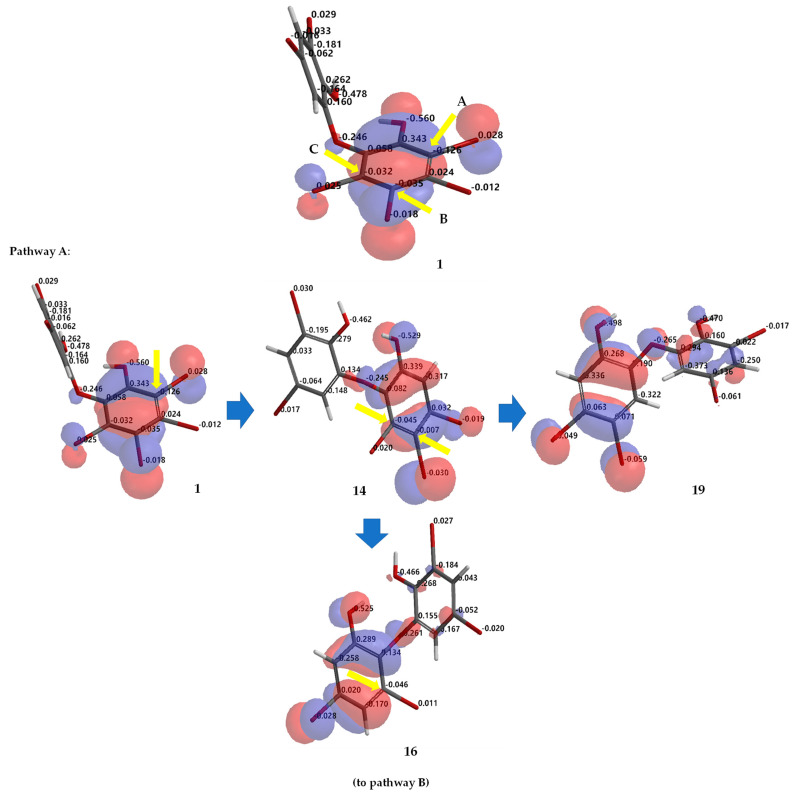

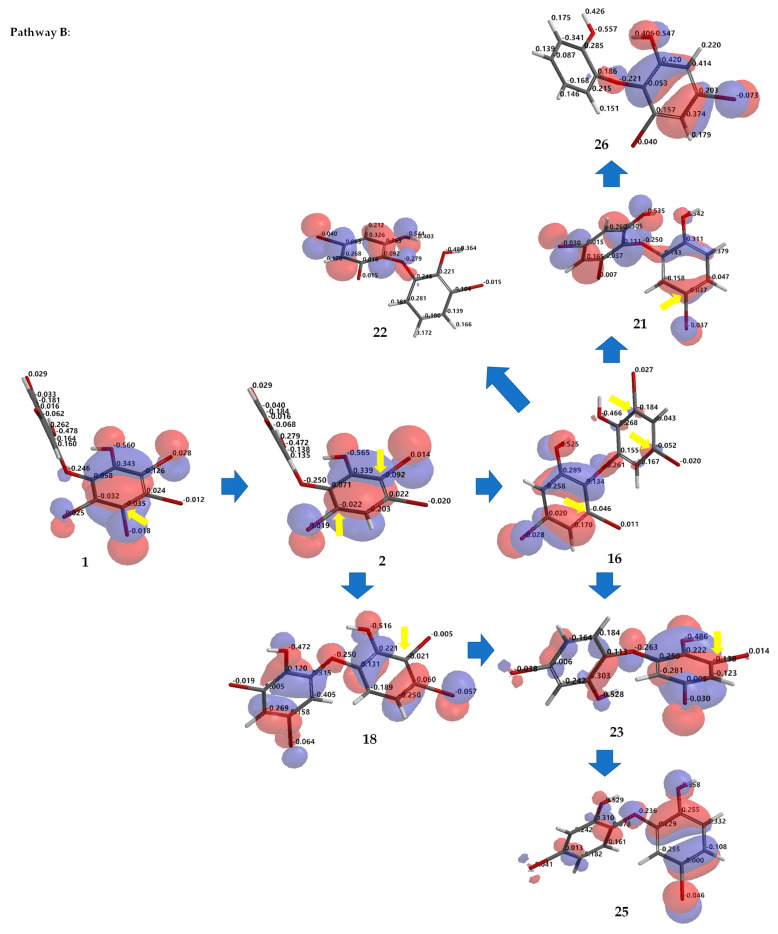

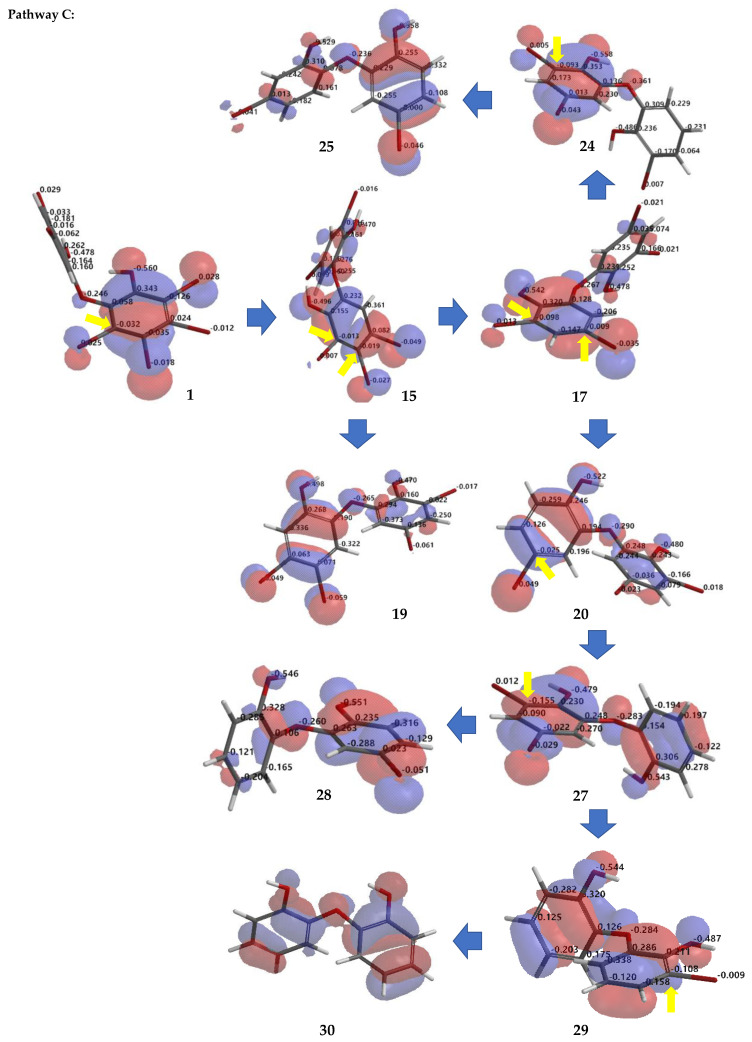
Analysis of frontier molecular orbital (HOMO) and electrostatic charges for **1**, **2**, **14**, **16**–**18**, **20**, **23**, **26** calculated using DFT ωB97X-D/6-311+G(2d,p). The yellow arrow shows the reactive site for the debromination reaction possessing negative charges in HOMO region.

Debromination of **1**, including its product, can be supported by DFT ωB97X-D/6–311 + G(2d,p) calculations. Combination analysis of frontier molecular orbital (HOMO) for **1**, **2**, **14**, **16**–**18**, **20**, **23, 26** and their electrostatic charge are shown in Figure 7 allowing us to propose the three putative debromination pathways.

Only *o*, *p*-bromines were selectively reduced; hence, we could deduce the isolated products with the three putative debromination pathways **A**–**C** shown in Figure 7. Debromination in this study gave nine compounds, (**1**, **2**, **14**, **16**–**18**, **20**, **23, 26**). In addition, there were **8** hypothetical compounds (**21**, **22**, **24**, **25**, **27**–**30**) that we have predicted on the basis of computational studies. This includes **15,** which was reported previously [21,24,25]. Based on analysis of FMO and electrostatic charge for **1** on ring A, three reactive sites were readily identified in HOMO region as C2 (electrostatic charge: −0.125), C5 (−0.035), and C6 (−0.032) corresponding to Br atoms in the *ortho* and *para* positions toward the -OH group and the Br atom in the *ortho* position toward the −OR group, respectively which led to the three putative pathways **A**–**C**. Moreover, combination analysis of FMOs and electrostatic charges for the three pathways support the presence of debromination products as in **1**, **2**, **14**, **16**–**18**, **20**, **23**, and **26**. The high electron density and negative charge gave the possibility of attack through the *ortho* or *para* positions of the ring toward the lowest unoccupied molecular orbital (LUMO) HBr orbital leading to the selectivity of the reaction. Reaction products were predicted to occur via the *ortho* and *para* pathways toward hydroxy or phenoxy groups. This was because of competition between two activating groups (regioselective). Reaction products predicted to occur via the *ortho* toward hydroxy groups (pathway **A**) were **1** → **14** → **16** (the scheme continues on the pathway **B**); **1 14** → **19**. Reaction products predicted to occur via the *para* toward hydroxy groups (pathway **B**) were **1** → **2** → **16** → **21** → **26**; **1** → **2** → **16** → **22**; **1** → **2** → **16** → **23** → **25**; **1** → **2** → **18** → **23** → **25**. Reaction products predicted to occur via the *para* toward phenoxy groups (pathway **C**) were **1** → **15** → **17** → **20** → **27** → **28; 1** → **15** → **17** → **20** → **27** → **29** → **30**; **1** → **15** → **17** → **24**; **1** → **15** → **19**.

**Figure 8:**
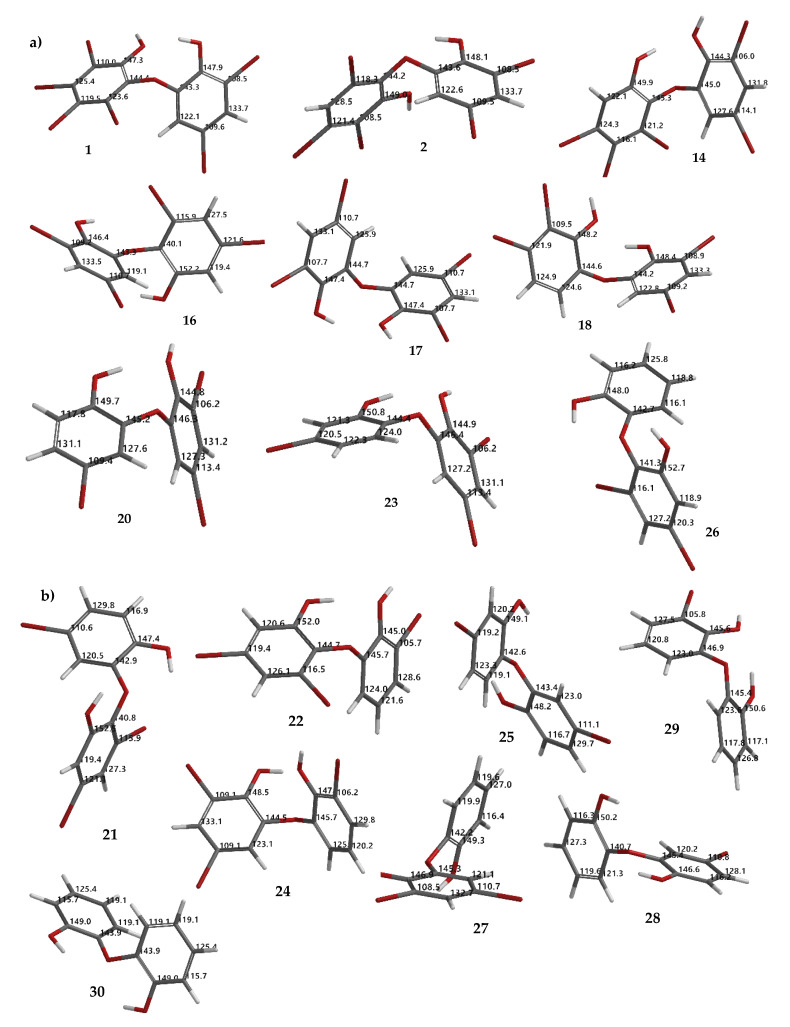
Theoretical ^13^C NMR chemical shifts of debromination products (**1**, **2**, **14**, **16**–**18**, **20**, **23, 26**) (**a**) and hypothetical molecules (**21**, **22**, **24**, **25**, **27**–**30**) calculated using the NMR chemical shift calculation protocol (**b**).

Based on Figure 7, we also observed the possibility of isomerization, which also supports the debromination mechanism proposed by Effenberger [17]. This may explain other products that are not directly obtained by removing bromine atoms in the *ortho* or *para* positions, to the electron-donating groups (EDG) -OH or-OR. Among the debromination products, compound **26** was the most stable (ΔE_LUMO-HOMO_ 9.38 eV) followed by **16** (9.14 eV), while less stable compound was **1** (8.62 eV). All hypothetical compounds (**21**, **22**, **24**, **25**, **27**–**30**) were relatively stable (9.03–9.29 eV) except for **24** (8.86 eV). Apparently, the molecules may exist during the reaction, offering a challenging task to isolate and to assay them with our interest assay panel because of nature of the molecules. In this computational study, we also proposed theoretical ^13^C NMR data for the experimental (**1**, **2**, **14**, **16**–**18**, **20**, **23**, and **26**) (Figure 8a) and hypothetical (**21**, **22**, **24**, **25**, **27**–**30**) (Figure 8b) molecules.

**Figure 9:**
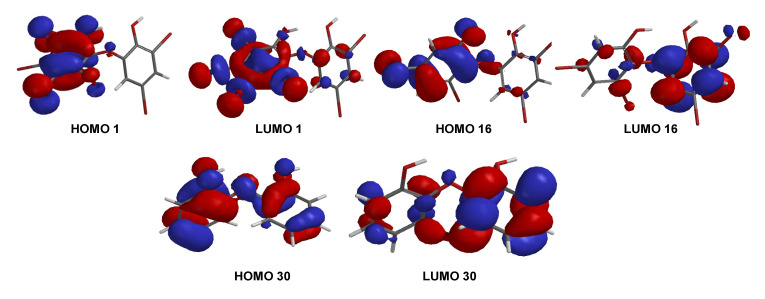
FMOs (HOMO and LUMO) for **1**, **16**, and **30**.

A series of PBDE molecules was evaluated for cytotoxicity using human embryonic kidney (HEK293T) cells, Gram-positive *Staphylococcus aureus*, and Gram-negative *Klebsiella pneumoniae*. Compound **1** (2, 3, 4, 5-tetrabromo-6-(3′, 5′-dibromo-2′-hydroxyphenoxy) phenol) showed cytotoxicity against HEK293T cells at IC_50_ 16.16 ± 1.68 µM, whereas compound **16** (3, 5-dibromo-6-(3′, 5′-dibromo-2′-hydroxyphenoxy) phenol) was checked twice against HEK293T cells from different reactions, showing IC_50_ 8.65 ± 1.11 and IC_50_ 8.11 ± 1.43 µM, respectively. Compound **30** (2, 2′-oxydiphenol) showed no cytotoxicity against HEK293T cells with an IC_50_ >197.82 µM. This result suggests that four bromines and their positions are important for cytotoxicity against HEK293T cells, as in **16**. Moreover, both bacteria showed MID = 0.078 µg/disk for compounds **1** and **16** (Table 2). The result was supported by the shape of HOMO and LUMO for **1**, **16**, and **30** (Figure 9). Compounds **1** and **16** showed the HOMO–LUMO orbitals located in different molecular regions and this characteristic may explain their potent antibacterial activity, as shown in many studies [9,26,27]. In contrast, **30** had HOMO–LUMO orbitals dispersed across almost the entire molecular region. While the MID of **1** and **16** were the same, the clear inhibition zone of **1** is slightly stronger than that of **16**. This was supported by E_LUMO_ of **1** (−0.05 eV) < E_LUMO_ of **16** (0.38 eV) while E_LUMO_ of **30** was 1.13 eV. It is clear that the higher number of bromine atoms, as in **1** stabilizes LUMO energy and gives slightly stronger antibacterial activity against the Gram-positive bacterium, *S. aureus* [27]. In contrast, the antibacterial activity of **16** against Gram-negative bacterium, *K. pneumoniae*, was slightly more potent than that of **1**.

Compound **16** was discovered previously in the marine sponge, *Dysidea herbacea* collected from Australia [29] and later in *Lamellodysidea* sp. [24] collected from Papua New Guinea. It showed a wide range of antibiotic activities against *Staphylococcus aureus* (MIC 1.25–1.6 µg/mL), *Enterococcus faecium* (MIC 1.6–3.1 µg/mL), *Escherichia coli* (MIC 50 µg/mL), *Pseudomonas aeruginosa* and *Candida albicans* (MIC >50 µg/mL), while it also had an IC_50_ >50 µg/mL against Bsc-1 cells [24]. Methylation and acetylation of **1** reduced cytotoxicity, with an IC_50_ >10 µg/mL against HEK293T, as in **7**–**13**. Meanwhile, antibacterial activity of methylation and acetylation products was weaker, with MID >0.078 µg/disk against Gram-positive *Staphylococcus aureus* and Gram-negative *Klebsiella pneumoniae*.

In summary, we found new derivatives **10**, **12**, **13** from acetylation by comparing data published between 1969 and 2020. We performed the acetylation with a new method using green chemistry at room temperature and determined the structures using analysis of FMOs and electrostatic charges. In addition, we also discovered a new debromination product **18** and new crystal structures of methylated **8** and **9** with twist conformations (*φ*_1_, *φ*_2_ > 0°) [30]. The presence of debromination products (**1**, **2**, **14**, **16**–**18**, **20**, **23**, **26**) and hypothetical molecules (**21**, **22**, **24**, **25**, **27**–**30**) were predicted using analysis of FMOs and electrostatic charges showing regioselectivity *ortho* and *para* positions toward the EDG -OH or -OR groups in PBDEs. The analysis also showed the possibility of isomerization among the PBDEs. All products methylations, acetylations, and debrominations were characterized for their theoretical ^13^C NMR chemical shifts. Novel ^13^C NMR data **6** are also reported (RMSE 3.2 ppm). PBDE compounds that lose hydroxyl groups, due to methylation and acetylation, have weaker biological activity. Fewer bromines, as in **16**, resulted in a significant IC_50_ 8.65 ± 1.11; 8.11 ± 1.43 µM against HEK293T cells. Compound **16** also showed antibacterial activity against Gram-positive *Staphylococcus aureus*, as well as Gram-negative *Klebsiella pneumoniae*, with an MID = 0.078 µg/disk. Cytotoxicity and antibacterial assays of derived compounds show that two phenolic hydroxyl groups and four bromine atoms are important for these activities. The result of active compounds was supported by analysis of FMOs. Additional information can be found in the Supplementary Materials.

## 3. Materials and Methods

### 3.1. General Methods

NMR spectra were measured on a 500 MHz Bruker Avance III spectrometer (MA, USA) or a 500 MHz JEOL (Tokyo, Japan) or a 500 MHz Varian (CA, USA). Chemical shifts were referenced to tetramethylsilane (TMS) or acetone (Me_2_CO) signals. MS spectra were recorded on a Waters Acquity Xevo G2-S ESIQTOF in positive mode or an HRESITOFMS JEOL T100LP, or EIMS were measured on a Hitachi M-2500 instrument. UV and IR spectra were obtained on a Perkin Elmer Spectrum One FTIR and on a Shimadzu Pharmaspec 1700 spectrophotometer. X-ray analysis was performed on a Rigaku AFC10 goniometer equipped with a Saturn 724+ detector. High-performance liquid chromatography (HPLC) separations were carried out on a Hitachi L-6000 pump fitted with Shodex RI-101 refractive index and SPD-20A Shimadzu UV detectors, or a Shimadzu HPLC with Prominence LC-20AD, DGU-20A5, SPD-20A. A Cosmosil 5SL-II-MS (10 × 250 mm) column was used for HPLC. Analytical thin-layer chromatography (TLC) was performed on Merck silica gel 60 F_254_ plates and visualized with sulfuric acid with cerium sulfate. All solvents used were reagent grade.

### 3.2. Animal Material

Marine sponges were collected by hand while scuba diving in Banten Province, Indonesia at a depth of 5–10 m. Samples were then stored in EtOH. Sponges were identified as *Lamellodysidea herbacea* by NJdeV.

### 3.3. Extraction and Isolation

A fresh marine sponge specimen (wet weight 300 g) stored in EtOH was extracted with MeOH. The combined extract was concentrated under vacuum, and the resulting residue was partitioned between hexane and aqueous MeOH (90%). The latter layer was further partitioned between CH_2_Cl_2_ and aqueous MeOH (50%). Finally, the aqueous MeOH (50%) was removed and adjusted with water, followed by extraction with *n*-BuOH. The three layers: Hexane, CH_2_Cl_2_ and BuOH layer were evaluated for activity against Gram-positive and Gram-negative bacteria. Recrystallization of the CH_2_Cl_2_ layer using CHCl_3_–Me_2_CO–MeCN gave **1** (1.36 g). A non-crystalline fraction was separated using either a silica gel column or silica HPLC eluted with hexane/EtOAc/MeOH, followed by recrystallization to give compound **2** (5.4 mg), a mixture of compounds **2** and **3** (5.6 mg), compound **4** (7.3 mg), and compound **5** (0.8 mg). Additional **2** (8.3 mg) and **3** (11.9 mg) were isolated from the hexane fraction after open column chromatography. Compounds **4** (163.5 mg) and **6** (50.6 mg) were isolated from the hexane layer collected from another *L. herbacea.*

### 3.4. Methylation

To a solution of **1** (9.1 mg) in MeOH (1 mL), 2 M TMSCHN_2_ in hexane was added dropwise. The reaction was monitored by TLC. The solution was allowed to stand at room temperature and concentrated to dryness under a stream of nitrogen followed by purification using HPLC (RP 18, MeOH, MeCN + 0.1% TFA) to give **7** (3 mg) and **8** (2.4 mg). Compound **7**: *R_f_* 0.38 (Hex/EtOAc 8:1, n-silica, UV λ 254 nm), ^1^H NMR (Me_2_CO-*d*_6_) *δ* 3.87 (3H, s), 6.88 (1H, d, *J* = 2.2 Hz), 7.43 (1H, d, *J* = 2.2 Hz), HRESIMS *m/z* 690.0626 [M + H]^+^ (C_13_H_6_O_3_^79^Br_5_^81^Br, Δ −0.4808 mmu). Compound **8**: *R_f_* 0.57 (Hex/EtOAc 8:1, n-silica, UV λ 254 nm), ^1^H NMR (CDCl_3_) *δ* 3.85 (3H, s), 4.00 (3H, s), 6.97 (1H, d, *J* = 2.2 Hz), 7.50 (1H, d, *J* = 2.2 Hz), HRESIMS *m/z* 702.6289 [M + H]^+^ (C_14_H_9_O_3_^79^Br_4_^81^Br_2_, Δ 0.0678 mmu).

To a solution of **2** (5.2 mg) in MeOH (1 mL) excess 2 M TMSCHN_2_ in hexane was added and monitored by TLC. The solution was allowed to stand at room temperature and concentrated to dryness under a stream of nitrogen to give the total methyl derivative **9** (5.2 mg). Compound **9**: *R_f_* 0.63 (Hex/EtOAc 8:1, n-silica, UV λ 254 nm), ^1^H NMR (CDCl_3_) *δ* 3.82 (3H, s), 4.00 (3H, s), 6.50 (1H, d, *J* = 2.2 Hz), 7.40 (1H, d, *J* = 2.2 Hz), 7.76 (1H, s), HRESIMS *m/z* 630.5989 [M + H]^+^ (C_14_H_10_O_3_^81^Br_5_ Δ −0.0456 mmu).

A solution of **4** (8.9 mg) in MeOH (1 mL) was treated similarly with diluted TMSCHN_2_ to give compound **9** (8.9 mg). Compound **9**: *R_f_* 0.63 (Hex/EtOAc 8:1, n-silica, UV λ 254 nm, ^1^H NMR (CDCl_3_) *δ* 3.82 (3H, s), 4.00 (3H, s), 6.50 (1H, d, *J* = 2.2 Hz), 7.40 (1H, d, *J* = 2.2 Hz), 7.76 (1H, s), HRESIMS *m/z* 630.5989 [M + H]^+^ (C_14_H_10_O_3_^81^Br_5_ Δ −0.0456 mmu).

### 3.5. Acetylation

Screening of acetylation was performed under seven conditions with a variety of catalysts and solvents, without catalyst and solvent, with or without sonication. Acetylation of **1** with Ac_2_O and sonication for 1 h proceeded to give **10** and **11**. Acetylation of **2** with Ac_2_O and sonication for 1 h proceeded to give **12** and **13**. The reaction was performed without DMAP, Et_3_N, and solvent.

To **1** (15 mg) Ac_2_O (2.1 mmol) was added and sonicated for 2–2.5 h at room temperature to give **10** (1.1 mg) and **11** (6.4 mg) after purification by HPLC (RP18, MeCN + 0.1% TFA). Compound **10**: *R_f_* 0.23 (Hex/EtOAc 8:1, n-silica, UV λ 254 nm), ^1^H NMR (Me_2_CO-*d*_6_) *δ* 2.40 (3H, s), 7.12 (1H, d, *J* = 2.2 Hz), 7.62 (1H, d, *J* = 2.2 Hz), ^1^H NMR (CDCl_3_) *δ* 2.47 (3H, s), 6.62 (1H, d, *J* = 2.1 Hz), 7.51 (1H, d, *J* = 2.1 Hz), HRESIMS *m/z* 716.5404 [M + H]^+^ (C_14_H_7_O_4_^79^Br_4_^81^Br_2_, Δ 0 mmu). Compound **11**: *R_f_* 0.23 (Hex/EtOAc 8:1, n-silica, UV λ 254 nm), ^1^H NMR (Me_2_CO-*d*_6_) *δ* 2.24 (3H, s), 2.36 (3H, s), 7.14 (1H, d, *J* = 2.1 Hz), 7.64 (1H, d, *J* = 2.1 Hz), HRESIMS *m/z* 780.5329 [M + Na]^+^ (C_16_H_8_O_5_^79^Br_4_^81^Br_2_Na, Δ −0.0002 mmu).

To **2** (15.3 mg) Ac_2_O (2.1 mmol) was added and sonicated for 2–2.5 h at room temperature to give **12** (3.7 mg) and **13** (2.2 mg) after purification using HPLC (RP 18, MeCN + 0.1% TFA). Compound **12**: *R_f_* 0.34 (Hex/EtOAc 8:1, n-silica, UV λ 254 nm), ^1^H NMR (Me_2_CO-*d*_6_) *δ* 2.40 (3H, s), 7.00 (1H, d, *J* = 2.1 Hz), 7.62 (1H, d, *J* = 2.1 Hz), 7.66 (1H, s), ^1^H NMR (CDCl_3_) *δ* 2.47 (3H, s), 6.63 (1H, d, *J* = 2.1 Hz), 7.50 (1H, d, *J* = 2.1 Hz), 7.54 (1H, s), HRESIMS *m/z* 640.6278 [M + H]^+^ (C_14_H_8_O_4_^79^Br_2_^81^Br_3_, Δ 0 mmu). Compound **13**: *R_f_* 0.51 (Hex/EtOAc 8:1, n-silica, UV λ 254 nm), ^1^H NMR (Me_2_CO-*d*_6_) *δ* 2.24 (3H, s), 2.36 (3H, s), 7.01 (1H, d, *J* = 2.1 Hz), 7.63 (1H, d, *J* = 2.1 Hz), 8.13 (1H, s), ^1^H NMR (CDCl_3_) *δ* 2.25 (3H, s), 2.36 (3H, s), 6.58 (1H, d, *J =* 2.0 Hz), 7.47 (1H, d, *J =* 2.0 Hz), 7.87 (1H, s), HRESIMS *m/z* 704.6226 [M + Na]^+^ (C_16_H_9_O_5_^79^Br_2_^81^Br_3_Na, Δ 0.0023 mmu).

### 3.6. Debromination

HBr (10 mL) and Na_2_SO_3_ (20 equivalents) in AcOH (20 mL) were added to compound **1** (60 mg). The mixture was refluxed for 6 h and then neutralized with dilute KOH to pH 7 and partitioned using EtOAc and H_2_O. The organic layer formed was separated and purified by HPLC (RP18, MeOH) to give **1** (1.8 mg) and **2** (0.3 mg). Compound **1**: *R_f_* 0.51 (EtOAc/Hexane 1:2, n-silica, UV *λ* 254 nm); ^1^H NMR (Me_2_CO-*d*_6_) *δ* 6.83 (1H, d, *J* = 2.2 Hz), 7.40 (1H, d, *J* = 2.2 Hz). Compound **2**: *R_f_* 0.43 (EtOAc/Hexane 1:2, n-silica, UV *λ* 254 nm), ^1^H NMR (Me_2_CO-*d*_6_) *δ* 6.79 (1H, d, *J* = 2.3 Hz), 7.38 (1H, d, *J* = 2.3 Hz), 7.47 (1H, s).

HBr (2.5 mL) and Na_2_SO_3_ (15 equivalents) in AcOH (20 mL) were added to compound **1** (55.5 mg). The mixture was refluxed for 6 h and then dilute KOH was added to raise the pH to 10–11, after which it was partitioned using EtOAc and H_2_O. The organic layer formed was separated and purified by HPLC (RP18, MeOH, MeOH-H_2_O 2:1 + 0.1% TFA) to give **1** (1.26 mg), **2** (10.5 mg), **14** (1.2 mg), **16** (1.0 mg), **17** (2.8 mg), and **18** (0.5 mg). Compound **1**: ^1^H NMR (CD_3_OD) *δ* 6.47 (1H, d, *J* = 2.2 Hz), 7.35 (1H, d, *J* = 2.2 Hz). Compound **14** in mixture form with **2.** Compound **2**: ^1^H NMR (CD_3_OD) *δ* 6.65 (1H, bs), 7.32 (1H, d, *J* = 2.3 Hz), 7.39 (1H, bs). Compound **14**: ^1^H NMR (CD_3_OD) *δ* 6.45 (1H, d, *J* = 2.3 Hz), 7.31 (1H, d, *J* = 2.3 Hz), 7.38 (1H, s). Compound **16**: ^1^H NMR (Me_2_CO-*d*_6_) *δ* 6.64 (1H, d, *J* = 2.5 Hz), 7.24 (1H, d, *J* = 2.5 Hz), 7.37 (1H, d, *J* = 2.5 Hz), 7.38 (1H, d, *J* = 2.5 Hz), HREIMS *m/z* 515.7007 [M]^+^ (C_12_H_6_O_3_^79^Br_3_^81^Br, Δ −0.0023 mmu). Compound **17**: ^1^H NMR (Me_2_CO-*d*_6_) 6.69 (1H, d, *J* = 2.5 Hz), *δ* 6.74 (1H, d, *J* = 2.5 Hz), 7.27 (1H, d, *J* = 2.5 Hz,), 7.78 (1H, d, *J* = 2.5 Hz), LREIMS *m/z* 517.7 [M]^+^ (C_12_H_6_O_3_^81^Br_2_^79^Br_2_). Compound **18**: ^1^H NMR (Me_2_CO*-d*_6_) *δ* 6.67 (d, overlapped), 7.03 (2H, dd, *J* = 8.5 Hz), 7.81 (1H, d, *J* = 2.5 Hz), LREIMS *m/z* 517.7 [M]^+^ (C_12_H_6_O_3_^79^Br_2_^81^Br_2_).

To **1** (60.6 mg) in AcOH HBr (10 mL) and Na_2_SO_3_ (20 equivalents) in AcOH (20 mL) were added. The mixture was refluxed for 24 h and then neutralized with dilute KOH to pH 7 and partitioned using EtOAc and water. The organic layer formed was separated and purified by HPLC (RP 18, MeCN + 0.1% TFA) to give **16** (4.1 mg) and **20** (0.5 mg). Compound **16**: *R_f_* 0.79 (EtOAc/Hexane 1:2, n-silica, UV *λ* 254 nm), ^1^H NMR (Me_2_CO-*d*_6_) *δ* 6.84 (1H, d, *J* = 2.3 Hz), 7.16 (1H, d, *J* = 2.3 Hz), 7.27 (1H, d, *J* = 2.3 Hz), 7.36 (1H, d, *J* = 2.3 Hz). Compound **20**: *R_f_*0.65 (EtOAc/Hexane 1:2, n-silica, UV *λ* 254 nm), ^1^H NMR (Me_2_CO-*d*_6_) *δ* 6.60 (1H, d, *J* = 2.3 Hz), 6.91 (1H, d, *J* = 8.5 Hz), 7.06 (1H, dd, *J* = 8.5, 2.3 Hz), 7.27 (1H, d, *J* = 2.3 Hz), 7.37 (1H, d, *J* = 2.3 Hz), HRESIMS *m/z* 441.2803 [M + H]^+^ C_12_H_7_O_3_^81^Br_3_ (Δ −1.5159 mmu).

To compound **1** (60.1 mg) HBr (10 mL) and Na_2_SO_3_ (20 equivalents) in AcOH (20 mL) were added. The mixture was refluxed for 31 h and then the pH was raised to 10–11 with dilute KOH. The solution was partitioned using EtOAc and H_2_O. The organic layer was separated and purified by HPLC (RP18, MeOH, CH_3_CN-H_2_O 2:1) to give **17** (4.6 mg), **20** (2.2 mg), **23** (2.4 mg), and **26** (0.2 mg). Compound **20**: ^1^H NMR (CD_3_OD) *δ* 6.74 (1H, d, *J* = 8.5 Hz), 6.79 (2H, q, *J* = 2.0 Hz), 6.97 (1H, dd, *J* = 2.0, 8.5 Hz), 7.07 (1H, d, *J* = 2.0 Hz). Compound **23**: ^1^H NMR (Me_2_CO*-d*_6_) *δ* 6.67 (1H, d, *J* = 2.0 Hz), 6.75 (1H, d, *J* = 8.5 Hz), 6.93 (1H, dd, *J* = 2.0, 8.5 Hz), 7.03 (1H, d, *J* = 2.0 Hz), 7.12 (1H, d, *J* = 2.0 Hz). HREIMS *m/z* 435.7957 [M]^+^ C_12_H_7_O_3_^79^Br_2_^81^Br (Δ 0.0012 mmu). Compound **26**: ^1^H NMR (CD_3_OD) *δ* 6.48 (1H, d, *J* = 8.0 Hz), 6.62 (1H, td, *J* = 8.0, 2.0 Hz), 6.87 (1H, dq, *J* = 8.0, 2.0 Hz), 6.87 (1H, d, *J* = 2.0 Hz), 7.05 (1H, d, *J* = 2.5 Hz), 7.22 (1H, d, *J* = 2.5 Hz). HREIMS *m/z* 357.8824 [M]^+^ C_12_H_9_O_3_^79^Br_2_ (Δ −0.0016 mmu).

### 3.7. Computational Study

Equilibrium geometries were calculated for all structures using the ωB97X-D/6–311 + G(2d,p) density functional model implemented by Spartan ’20 (Wavefunction Inc, Irvine, CA, USA). Then, HOMO, LUMO, and electrostatic charge data can be used to support analysis of synthesized and natural compounds. Calculation of theoretical ^13^C NMR chemical shifts was performed according the protocol of Hehre et al. [6], comprising six steps: (1) A conformer search using the MMFF molecular mechanic model was performed and high energy conformers (40 kJ/mol) were removed. (2) Equilibrium geometries were calculated using the HF/3-21G model and duplicate conformers were removed. Then, high-energy conformers (40 kJ/mol) were removed. (3) Energies were calculated using the ωB97X-D/6-31G* density functional model and high-energy conformers (15 kJ/mol) were removed. (4) Equilibrium geometries were calculated using the ωB97X-V/6–311 + G(2df,2p) (6–311G*) density functional model and high-energy conformers (10 kJ/mol) were removed. (5) Energies were calculated using the ωB97X-V/6–311 + G(2df,2p) (6–311G*) density functional model. (6) ^13^C NMR chemical shifts were calculated using the ωB97X-D/6–31G* followed by correction of ^13^C NMR chemical shifts based on the empirical parameters followed by correction ^13^C NMR chemical shifts based on the Boltzmann weight obtained in step 5.

### 3.8. X-ray Study

Single crystals of C_12_H_6_Br_6_O_4_ (**1**), C_14_H_8_Br_6_O_3_ (**8**), and C_14_H_9_Br_5_O_3_ (**9**) were supplied. A suitable crystal was selected and mounted on Rigaku Saturn 724 Plus with AFC10. The crystal was kept at 113 K or 123.15 K during data collection. Using Olex2 [31], the structure was solved with the SHELXT [32] structure solution program using direct methods and refined with the SHELXL [33] refinement package using least squares minimization.

Crystal data for **1**, C_12_H_6_Br_6_O_4_ (M =693.63 g/mol): Monoclinic, space group P2_1_/n (no. 14), a = 4.74979(10) Å, b = 18.7360(3) Å, c = 18.3463(4) Å, β = 91.6135(19)°, V = 1632.02(6) Å^3^, Z = 4, T = 123.15 K, μ(MoKα) = 14.772 mm^−1^, Dcalc = 2.823 g/cm^3^, 20780 reflections measured (3.108° ≤ 2Θ ≤62.354°), 4898 unique (R_int_ = 0.0374, R_sigma_ = 0.0252) which were used in all calculations. The final R_1_ was 0.0435 (I >2σ(I)) and wR_2_ was 0.1269 (all data).

Crystal data for **8**, C_14_H_8_Br_6_O_3_ (*M* =703.66 g/mol): Triclinic, space group P-1 (no. 2), *a* = 8.733(3) Å, *b* = 8.911(3) Å, *c* = 12.769(4) Å, *α* = 104.185(4)°, *β* = 100.011(3)°, *γ* = 101.892(3)°, *V* = 916.0(5) Å^3^, *Z* = 2, *T* = 113 K, μ(MoKα) = 13.158 mm^−1^, *Dcalc* = 2.551 g/cm^3^, 7554 reflections measured (6.588° ≤ 2Θ ≤54.968°), 4046 unique (*R*_int_ = 0.0369, R_sigma_ = 0.0427) which were used in all calculations. The final R_1_ was 0.0422 (I >2σ(I)) and wR_2_ was 0.0997 (all data).

Crystal data for **9**, C_14_H_9_Br_5_O_3_ (M =624.76 g/mol): Orthorhombic, space group Pca2_1_ (no. 29), a = 6.9014(15) Å, b = 11.940(3) Å, c = 21.090(5) Å, V = 1737.9(7) Å^3^, Z = 4, T = 113.15 K, μ(MoKα) = 11.569 mm^−1^, Dcalc = 2.388 g/cm^3^, 17770 reflections measured (6.82° ≤ 2Θ ≤ 54.952°), 3584 unique (R_int_ = 0.0623, R_sigma_ = 0.0509) which were used in all calculations. The final R_1_ was 0.0359 (I > 2σ(I)) and wR_2_ was 0.0681 (all data).

### 3.9. Agar-Plate Diffusion Assay

*Staphylococcus aureus* ATCC 6538 and *Klebsiella pneumoniae* were used for biological evaluation. Concentrations assayed ranged from 0.08 to 1.25 µg/disks [14,15]. DMSO was used to dissolve the compounds, while vancomycin and oxacillin were used as positive controls for *Staphylococcus aureus* and gentamycin was used as a positive control for *Klebsiella pneumoniae*. The minimum inhibitory dose (MID, μg/disk) was defined as the minimum dose that induced an obvious inhibition zone (1–1.5 mm) [28]. The disk diameter was 6 mm.

### 3.10. In Vitro Cytotoxicity Assay

In vitro cytotoxicity was determined against human embryonic kidney (HEK293T) cells. The assay was performed in 96-well treated tissue culture plates. Cells were seeded in the wells (5000 cells in 100 µL media containing RPMI1640, FBS, penicillin and streptomycin) and incubated for 24 h. Samples were then added and plates were again incubated for 48 h. MTT was then added and the plates were incubated for 4 h at 37 °C. Formazan crystals formed were dissolved in EtOH and absorbances were read at λ = 595 nm. The result was analyzed by using Prism 9 software (Graphpad, San Diego, CA, USA) to obtain IC_50_ values.

## Figures and Tables

**Table 1:** ^13^C (125 MHz) and ^1^H NMR (500 MHz) data of **6** in Me_2_CO-*d*_6_.

Position	δ_C_ (Mult.)	δ_H_ (*J* in Hz)	HMBC
1	151.3 C		
2	122.0 CH	7.50 (1H, s)	1, 3, 4, 6
3	122.5 C		
4	116.9 C		
5	122.7 C		
6	140.1 C		
2′-OMe	61.1 CH_3_	3.99 (3H, s)	2′
1′	152.0 C		
2′	146.5 C		
3′	119.4 C		
4′	129.4 CH	7.40 (1H, d, *J* = 2.5)	2′, 3′, 5′, 6′
5′	117.8 C		
6′	117.4 CH	6.78 (1H, d, *J* = 2.5)	1′, 2′, 3′, 5′

**Table 2:** Minimum inhibitory doses and inhibitory concentrations of compounds **1**, **16**, **30**.

Compound	Cytotoxicity HEK293T	Antibacterial Activity Gram-Positive *S. aureus*	Antibacterial Activity Gram-Negative *K. pneumoniae*
	IC_50_ (µM)	MID (µg/Disk) ^a^/Clear Inhibition Zone mm	
**1**	16.16 ± 1.17	0.078/7.17 ± 0.24	0.078/7.00 ± 0.00
**16**	8.65 ± 1.11; 8.11 ± 1.43	0.078/7.00 ± 0.00	0.078/7.50 ± 0.00
**30**	>197.82	NA	NA
Doxorubicin	0.22 ± 0.09		
Vancomycin		0.078/7.00 ± 0.00	0.078/7.33 ± 0.24
Gentamycin		0.078/7.67 ± 0.47	0.078/7.33 ± 0.47
Oxacillin		<0.078/11.33 ± 0.24	0.078/7.33 ± 0.24

^a^ The minimum inhibitory dose (MID, μg/disk) is defined as the minimum dose that induced an obvious inhibition zone (1–1.5 mm) [28].

## Data Availability

The data presented in this study are available from the corresponding authors on request.

## Supplementary Materials

The following are available online, Figure S1: 1H NMR spectrum of 1 (Me2CO-d6, 500 MHz), Figure S2: 1H NMR spectrum of 1 (CD3OD, 500 MHz), Figure S3: Crystal Structure of 1, Figure S4: HOMO-LUMO of 1, Figure S5: Calculated 13C NMR Chemical Shift of 1, Figure S6: 1H NMR spectrum of 2 (Me2CO-d6, 500 MHz), Figure S7: HOMO-LUMO of 2, Figure S8: Calculated 13C NMR Chemical Shift of 2, Figure S9: 1H NMR spectrum of 3 (CDCl3, 500 MHz), Figure S10: HOMO-LUMO of 3, Figure S11: Calculated 13C NMR Chemical Shift of 3, Figure S12: 1H NMR spectrum of 4 (Me2CO-d6, 500 MHz), Figure S13: HOMO-LUMO of 4, Figure S14: Calculated 13C NMR Chemical Shift of 4, Figure S15: 1H NMR spectrum of 5 (CDCl3, 500 MHz), Figure S16: HOMO-LUMO of 5, Figure S17: Calculated 13C NMR Chemical Shift of 5, Figure S18: 1H NMR spectrum of 6 (Me2CO-d6, 500 MHz), Figure S19: 13C NMR spectrum of 6 (Me2CO-d6, 500 MHz), Figure S20: 1H-13C HSQC spectrum of 6 (Me2CO-d6, 500 MHz), Figure S21: 1H-13C HMBC spectrum of 6 (Me2CO-d6, 500 MHz), Figure S22: HREIMS of 6, Figure S23: HOMO-LUMO of 6, Figure S24: Calculated 13C NMR Chemical Shift of 6, Figure S25: 1H NMR spectrum of 7 (Me2CO-d6, 500 MHz), Figure S26: HRESIMS of 7, Figure S27: HOMO-LUMO of 7, Figure S28: Calculated 13C NMR Chemical Shift of 7, Figure S29: 1H NMR spectrum of 8 (CDCl3, 500 MHz), Figure S30: HRESIMS of 8, Figure S31: Crystal Structure of 8, Figure S32: HOMO-LUMO of 8, Figure S33: Calculated 13C NMR Chemical Shift of 8, Figure S34: 1H NMR spectrum of 9 (CDCl3, 500 MHz), Figure S35: HRESIMS of 9, Figure S36: Crystal Structure of 9, Figure S37: HOMO-LUMO of 9, Figure S38: Calculated 13C NMR Chemical Shift of 9, Figure S39: 1H NMR spectrum of 10 (Me2CO-d6, 500 MHz), Figure S40: 1H NMR spectrum of 10 (CDCl3, 500 MHz), Figure S41: 1H-1H COSY spectrum of 10 (CDCl3, 500 MHz), Figure S42: 1H-13C HSQC spectrum of 10 (CDCl3, 500 MHz), Figure S43: 1H-13C HMBC spectrum of 10 (CDCl3, 500 MHz), Figure S44: HRESIMS of 10, Figure S45: HOMO-LUMO of 10, Figure S46: Calculated 13C NMR Chemical Shift of 10, Figure S47: 1H NMR spectrum of 11 (Me2CO-d6, 500 MHz), Figure S48: HRESIMS of 11, Figure S49: HOMO-LUMO of 11, Figure S50: Calculated 13C NMR Chemical Shift of 11, Figure S51: 1H NMR spectrum of 12 (Me2CO-d6, 500 MHz), Figure S52: 1H NMR spectrum of 12 (CDCl3, 500 MHz), Figure S53: 1H-1H COSY spectrum of 12 (CDCl3, 500 MHz), Figure S54: 1H-13C HSQC spectrum of 12 (CDCl3, 500 MHz), Figure S55: 1H-13C HMBC spectrum of 12 (CDCl3, 500 MHz), Figure S56: HRESIMS of 12, Figure S57: HOMO-LUMO of 12, Figure S58: Calculated 13C NMR Chemical Shift of 12, Figure S59: 1H NMR spectrum of 13 (Me2CO-d6, 500 MHz), Figure S60: 1H NMR spectrum of 13 (CDCl3, 500 MHz), Figure S61: 1H-1H COSY spectrum of 13 (CDCl3, 500 MHz), Figure S62: 1H-13C HSQC spectrum of 13 (CDCl3, 500 MHz), Figure S63: 1H-13C HMBC spectrum of 13 (CDCl3, 500 MHz), Figure S64: 1H-13C HMBC spectrum of 13 (CDCl3, 500 MHz), Figure S65: HRESIMS of 13, Figure S66: HOMO-LUMO of 13, Figure S67: Calculated 13C NMR Chemical Shift of 13, Figure S68: 1H NMR spectrum of 14 (as a mixture with compound 2) (CD3OD, 500 MHz), Figure S69: HOMO-LUMO of 14, Figure S70: Calculated 13C NMR Chemical Shift of 14, Figure S71: HOMO-LUMO of 15, Figure S72: Calculated 13C NMR Chemical Shift of 15, Figure S73: 1H NMR spectrum of 16 (Me2CO-d6, 500 MHz), Figure S74: 1H NMR spectrum of 16 (Me2CO-d6, 500 MHz), Figure S75: 1H-1H COSY spectrum of 16 (Me2CO-d6, 500 MHz), Figure S76: 1H-13C HSQC spectrum of 16 (Me2CO-d6, 500 MHz), Figure S77: 1H-13C HMBC spectrum of 16 (Me2CO-d6, 500 MHz), Figure S78: HREIMS of 16, Figure S79: HOMO-LUMO of 16, Figure S80: Calculated 13C NMR Chemical Shift of 16, Figure S81: 1H NMR spectrum of 17 (Me2CO-d6, 500 MHz), Figure S82: LREIMS of 17, Figure S83: HOMO-LUMO of 17, Figure S84: Calculated 13C NMR Chemical Shift of 17, Figure S85: 1H NMR spectrum of 18 (Me2CO-d6, 500 MHz), Figure S86: LREIMS of 18, Figure S87: HOMO-LUMO of 18, Figure S88: Calculated 13C NMR Chemical Shift of 18, Figure S89: HOMO-LUMO of 19, Figure S90: Calculated 13C NMR Chemical Shift of 19, Figure S91: 1H NMR spectrum of 20 (Me2CO-d6, 500 MHz), Figure S92: 1H NMR spectrum of 20 (CD3OD, 500 MHz), Figure S93: HRESIMS of 20, Figure S94: HOMO-LUMO of 20, Figure S95: Calculated 13C NMR Chemical Shift of 20, Figure S96: HOMO-LUMO of 21, Figure S97: Calculated 13C NMR Chemical Shift of 21, Figure S98: HOMO-LUMO of 22, Figure S99: Calculated 13C NMR Chemical Shift of 22, Figure S100: 1H NMR spectrum of 23 (Me2CO-d6, 500 MHz), Figure S101: HREIMS of 23, Figure S102: HOMO-LUMO of 23, Figure S103: Calculated 13C NMR Chemical Shift of 23, Figure S104: HOMO-LUMO of 24, Figure S105: Calculated 13C NMR Chemical Shift of 24, Figure S106: HOMO-LUMO of 25, Figure S107: Calculated 13C NMR Chemical Shift of 25, Figure S108: 1H NMR spectrum of 26 (CD3OD, 500 MHz), Figure S109: HREIMS of 26, Figure S110: HOMO-LUMO of 26, Figure S111: Calculated 13C NMR Chemical Shift of 26, Figure S112: HOMO-LUMO of 27, Figure S113: Calculated 13C NMR Chemical Shift of 27, Figure S114: HOMO-LUMO of 28, Figure S115: Calculated 13C NMR Chemical Shift of 28, Figure S116: HOMO-LUMO of 29, Figure S117: Calculated 13C NMR Chemical Shift of 29, Figure S118: HOMO-LUMO of 30, Figure S119: Calculated 13C NMR Chemical Shift of 30, Table S1: Crystal Data & Structure Refinement for 1, Table S2: Fractional Atomic Coordinates (× 104) and Equivalent Isotropic Displacement Parameters (Å2 × 103) for 1. Ueq is defined as 1/3 of the trace of the orthogonalized UIJ tensor., Table S3: Anisotropic Displacement Parameters (Å2 × 103) for 1. The anisotropic displacement factor exponent takes the form: -2π2[h2a*2U11+2hka*b*U12+…], Table S4: Bond Lengths for 1, Table S5: Bond Angles for 1, Table S6: Torsion Angles for 1, Table S7: Hydrogen Atom Coordinates (Å × 104) and Isotropic Displacement Parameters (Å2 × 103) for 1, Table S8: Refinement Model Description of 1, Table S9: Equilibrium Geometry DFT ωB97X-D/6–311 + G(2d,p) of 1, Table S10: Equilibrium Geometry DFT ωB97X-D/6–311 + G(2d,p) of 2, Table S11: Equilibrium Geometry DFT ωB97X-D/6–311 + G(2d,p) of 3, Table S12: Equilibrium Geometry DFT ωB97X-D/6-311 + G(2d,p) of 4, Table S13: Equilibrium Geometry DFT ωB97X-D/6–311 + G(2d,p) of 5, Table S14: Equilibrium Geometry DFT ωB97X-D/6–311 + G(2d,p) of 6, Table S15: Equilibrium Geometry DFT ωB97X-D/6–311 + G(2d,p) of 7, Table S16: Crystal Data & Structure Refinement for 8, Table S17: Fractional Atomic Coordinates (×104) and Equivalent Isotropic Displacement, Parameters (Å2 × 103) for 8. Ueq is defined as 1/3 of the trace of the orthogonalized UIJ tensor., Table S18: Anisotropic Displacement Parameters (Å2 × 103) for 8. The anisotropic displacement factor exponent takes the form: -2π2[h2a*2U11+2hka*b*U12+…]., Table S19: Bond Lengths for 8, Table S20: Bond Angles for 8, Table S21: Torsion Angles for 8, Table S22: Hydrogen Atom Coordinates (Å × 104) and Isotropic Displacement Parameters (Å2 × 103) for 8, Table S23: Refinement Model Description of 8, Table S24: Equilibrium Geometry DFT ωB97X-D/6–311 + G(2d,p) of 8, Table S25: Crystal Data and Structure Refinement for 9, Table S26: Fractional Atomic Coordinates (×104) and Equivalent Isotropic Displacement Parameters (Å2 × 103) for 9. Ueq is defined as 1/3 of the trace of the orthogonalized UIJ tensor., Table S27: Anisotropic Displacement Parameters (Å2 × 103) for 9. The anisotropic displacement factor exponent takes the form: -2π2[h2a*2U11+2hka*b*U12+…]., Table S28: Bond Lengths for 9, Table S29: Bond Angles for 9, Table S30: Torsion Angles for 9, Table S31: Hydrogen Atom Coordinates (Å × 104) and Isotropic Displacement Parameters (Å2 × 103) for 9, Table S32: Refinement model description for 9, Table S33: Equilibrium Geometry DFT ωB97X-D/6–311 + G(2d,p) of 9, Table S34: Equilibrium Geometry DFT ωB97X-D/6–311 + G(2d,p) of 10, Table S35: Equilibrium Geometry DFT ωB97X-D/6–311 + G(2d,p) of 11, Table S36: Equilibrium Geometry DFT ωB97X-D/6–311 + G(2d,p) of 12, Table S37: Equilibrium Geometry DFT ωB97X-D/6–311 + G(2d,p) of 13, Table S38: Equilibrium Geometry DFT ωB97X-D/6–311 + G(2d,p) of 14, Table S39: Equilibrium Geometry DFT ωB97X-D/6–311 + G(2d,p) of 15, Table S40: Equilibrium Geometry DFT ωB97X-D/6-311+G(2d,p) of 16, Table S41: Equilibrium Geometry DFT ωB97X-D/6-311+G(2d,p) of 17, Table S42: Equilibrium Geometry DFT ωB97X-D/6-311+G(2d,p) of 18, Table S43: Equilibrium Geometry DFT ωB97X-D/6-311 + G(2d,p) of 19, Table S44: Equilibrium Geometry DFT ωB97X-D/6-311+G(2d,p) of 20, Table S45: Equilibrium Geometry DFT ωB97X-D/6-311+G(2d,p) of 21, Table S46: Equilibrium Geometry DFT ωB97X-D/6–311 + G(2d,p) of 22, Table S47: Equilibrium Geometry DFT ωB97X-D/6-311 + G(2d,p) of 23, Table S48: Equilibrium Geometry DFT ωB97X-D/6-311+G(2d,p) of 24, Table S49: Equilibrium Geometry DFT ωB97X-D/6–311 + G(2d,p) of 25, Table S50: Equilibrium Geometry DFT ωB97X-D/6-311+G(2d,p) of 26, Table S51: Equilibrium Geometry DFT ωB97X-D/6–311 + G(2d,p) of 27, Table S52: Equilibrium Geometry DFT ωB97X-D/6–311 + G(2d,p) of 28, Table S53: Equilibrium Geometry DFT ωB97X-D/6-311 + G(2d,p) of 29, Table S54: Equilibrium Geometry DFT ωB97X-D/6-311 + G(2d,p) of 30, Table S55: 1H NMR Data of 1–5, Table S56: NMR Data of 6 in Me2CO-d6, Table S57: 1H NMR Data of 7–9, Table S58: 1H NMR Data of 10–13, Table S59: 1H NMR Data of 2, 14, 16–18, Table S60: 1H NMR Data of 20, 24, 26. Table S61: EHOMO, ELUMO, and ΔE for 1–30.

## Sample Availability

Some samples of the compounds are available from the authors.

## References

[1] Mayer S., Prechtl M., Liebfried P., Cadeddu R.P., Stuhldreier F., Kohl M., Wenzel F., Stork B., Wesselborg S., Proksch P. (2019). First result from a screening of 300 naturally occurring compounds: 4,6-dibromo-2-(2′,4′-dibromophenoxy) phenol, 4,5,6-tribromo-2-(2′,4′dibromophenoxy) phenol, and 5-epi-nakijinone Q as substances with the potential for anticancer therapy. Mar. Drugs..

[2] Schmitt L., Hinxlage I., Cea P.A., Gohlke H., Wesselborg S. (2021). 40 years of research on polybrominated diphenyl ethers (PBDEs)-A historical overview and newest data of a promising anticancer drug. Molecules.

[3] Sun S., Canning C.B., Bhargava K., Sun X., Zhu W., Zhou N., Zhang Y., Zhou K. (2015). Polybrominated diphenyl ethers with potent and broad spectrum antimicrobial activity from the marine sponge Dysidea. Bioorg. Med. Chem. Lett..

[4] Agarwal V., El Gamal A., Yamanaka K., Poth D., Kersten R.D., Schorn M., Allen E.E., Moore B.S. (2014). Biosynthesis of polybrominated aromatic organic compounds by marine bacteria. Nat. Chem. Biol..

[5] Hanif N., Murni A., Tanaka C., Tanaka J. (2019). Marine natural products from Indonesian waters. Mar. Drugs..

[6] Hehre W., Klunzinger P., Deppmeier B., Driessen A., Uchida N., Hashimoto M., Fukushi E., Takata Y. (2019). Efficient protocol for accurately calculating 13C chemical shifts of conformationally flexible natural products: Scope, assessment, and limitations. J. Nat. Prod..

[7] Tirla A., Wernke K.M., Herzon S.B. (2021). On the stability and spectroscopic properties of 5-hydroxyoxazole-4-carboxylic acid derivatives. Org. Lett..

[8] Srivastava R. (2021). Theoritical studies on the molecular properties, toxicity, and biological efficacy of 21 new chemical entities. ACS Omega.

[9] Cortes E., Mora J., Márquez E. (2020). Modelling the anti-methicillin-resistant Staphylococcus aureus (MRSA) activity of cannabinoids: A QSAR and docking study. Crystals.

[10] Faulkner D.J., Unson M.D., Bewley C.A. (1994). The chemistry of some sponges and their symbionts. Pure Appl. Chem..

[11] Agarwal V., Blanton J.M., Podell S., Taton A., Schorn M.A., Busch J., Lin Z., Schmidt E.W., Jensen P.R., Paul V.J. (2017). Metagenomic discovery of polybrominated diphenyl ether biosynthesis by marine sponges. Nat. Chem. Biol..

[12] Podell S., Blanton J.M., Oliver A., Schorn M.A., Agarwal V., Biggs J.S., Moore B.S., Allen E.E. (2020). A genomic view of trophic and metabolic diversity in clade-specific Lamellodysidea sponge microbiomes. Microbiome..

[13] Walsh C.T., Wencewicz T.A. (2014). Prospects for new antibiotics: A molecule-centered perspective. J. Antibiot..

[14] Hanif N., Ardan S., Tohir D., Setiawan A., de Voogd N.J., Farid M., Murni A., Tanaka J. (2019). Polybrominated diphenyl ethers with broad spectrum antibacterial activity from the Indonesian marine sponge Lamellodysidea herbacea. J. App. Pharm. Sci..

[15] Hanif N., Tanaka J., Setiawan A., Trianto A., de Voogd N.J., Murni A., Tanaka C., Higa T. (2007). Polybrominated diphenyl ethers from the Indonesian sponge Lamellodysidea herbacea. J. Nat. Prod..

[16] Choi H.Y., Chi D.Y. (2001). A facile debromination reaction: Can bromide now be used as a protective group in aromatic systems?. J. Am. Chem. Soc..

[17] Effenberger F. (2002). How attractive is bromine as a protecting group in aromatic chemistry?. Angew. Chem. Int. Ed..

[18] Norton R.S., Croft K.D., Wells R.J. (1981). Polybrominated oxydiphenol derivatives from the sponge Dysidea herbacea: Structure determination by analysis of 13C spin-lattice relaxation data for quaternary carbons and 13C−1H coupling constants. Tetrahedron.

[19] Liu H., Namikoshi M., Meguro S., Nagai H., Kobayashi H., Yao X. (2004). Isolation and characterization of polybrominated diphenyl ethers as inhibitor of microtubule assembly from the marine sponge Phyllospongia dendyi at Palau. J. Nat. Prod..

[20] Fu F., Schmitz F.J., Govindan M., Abbas S.A., Hanson K.M., Horton P.A., Crews P., Laney M., Schatzman R.C. (1995). Enzyme inhibitors: New and known polybrominated phenols and diphenyl ethers from four Indo-Pacific Dysidea sponges. J. Nat. Prod..

[21] Calcul L., Chow R., Oliver A.G., Tenne K., White K.N., Wood A.W., Fiorilla C., Crews P. (2009). NMR strategy for unraveling structures of bioactive sponge-derived oxy-polyhalogenated diphenyl ethers. J. Nat. Prod..

[22] Anbu N., Nagarjun N., Jacob M., Kalaiarasi J.M.V.K., Dhakshinamoorthy A. (2019). Acetylation of alcohols, amines, phenols, thiols under catalyst and solvent-free conditions. Chemistry.

[23] De La Fuente J.Á., Manzanaro S., Martín M.J., de Quesada T.G., Reymundo I., Luengo S.M., Gago F. (2003). Synthesis, activity, and molecular modeling studies of novel human aldose reductase inhibitors based on a marine natural product. J. Med. Chem..

[24] Liu H., Lohith K., Rosario M., Pulliam T.H., O’Connor R.D., Bell L.J., Bewley C.A. (2016). Polybrominated diphenyl ethers: Structure determination and trends in antibacterial activity. J. Nat. Prod..

[25] Radwan M.M., Wanas A.S., Fronczek F.R., Jacob M.R., Ross S.A. (2015). Polybrominated diphenyl ethers from the marine organisms Lendenfeldia dendyi and Sinularia dura with anti-MRSA activity. Med. Chem. Res..

[26] Sathya A., Prabhu T., Ramalingam S. (2020). Structural, biological and pharmaceutical importance of antibiotic agent chloramphenicol. Heliyon.

[27] Kumar S., Saini V., Maurya I.K., Sindhu J., Kumari M., Kataria R., Kumar V. (2018). Design, synthesis, DFT, docking studies and ADME prediction of some new coumarinyl linked pyrazolylthiazoles: Potential standalone or adjuvant antimicrobial agents. PLoS ONE.

[28] Soe T.W., Han C., Fudou R., Kaida K., Sawaki Y., Tomura T., Ojika M. (2019). Clavariopsins C−I, antifungal cyclic depsipeptides from the aquatic hyphomycete Clavariopsis aquatica. J. Nat. Prod..

[29] Utkina N.K., Denisenko V.A. (2006). New polybrominated diphenyl ether from the marine sponge Dysidea herbacea. Chem. Nat. Compd..

[30] Klösener J., Swenson D.C., Robertson L.W., Luthe G. (2008). Electrostatic and aspheric influence of the fluoro-substitution of 4-bromodiphenyl ether (PBDE 3). Acta Crystallogr. B.

[31] Dolomanov O.V., Bourhis L.J., Gildea R.J., Howard J.A.K., Puschmann H. (2009). OLEX2: A complete structure solution, refinement and analysis program. J. Appl. Cryst..

[32] Sheldrick G.M. (2015). SHELXT-integrated space-group and crystal-structure determination. Acta Cryst..

[33] Sheldrick G.M. (2015). Crystal structure refinement with SHELXL. Acta Cryst..

