# Supplementary material for: Oxy-Polybrominated Diphenyl Ethers from the Indonesian Marine Sponge, Lamellodysidea herbacea: X-ray, SAR, and Computational Studies
Source: Molecules. 2021 Oct 20;26(21):6328. doi: 10.3390/molecules26216328 (PMC8588277; doi:10.3390/molecules26216328)
Supplement: Supplementary file 1 [file molecules-26-06328-s001.zip › molecules-1392181-supplementary.pdf]

Supplementary Materials

Oxy-polybrominated diphenyl ethers from the Indonesian marine sponge, *Lamellodysidea herbacea:* X-ray, SAR, and computational studies

Novriyandi Hanif[1,*], Trianda Ayuning Tyas[1,3], Lestari Hidayati[1], Fabians Faisal Dinelsa[1], Dian Provita[1], Nyimas Ratna Kinnary[1], Fauzi Muhamad Prasetiawan[1], Gibral Abdul Khalik[1], Zaki Mubarok[1], Dudi Tohir[1], Andi Setiawan[2], Muhamad Farid[1], Viqqi Kurnianda[3], Anggia Murni[4], Nicole J. de Voogd[5,6], Junichi Tanaka[3]

[1]Department of Chemistry, Faculty of Mathematics and Natural Sciences, IPB University, Bogor 16680, Indonesia
[2]Department of Chemistry, Lampung University, Bandar Lampung 35145, Indonesia
[3]Department of Chemistry, Biology, and Marine Science, University of the Ryukyus, Nishihara 903-0213, Okinawa, Japan
[4]Tropical Biopharmaca Research Center, IPB University, Bogor 16128, Indonesia
[5]Institute of Environmental Sciences, CML, Leiden University, P.O. Box 9518, 2300 RA Leiden, the Netherlands
[6]Naturalis Biodiversity Center, P. O. Box 9517, 2300 RA Leiden, the Netherlands
*Correspondence: nhanif@apps.ipb.ac.id; Telp.: +62-251-862-4567

Table of Contents

Title
Figure S1: ^{1}H NMR spectrum of **1** (Me_2CO-d_6, 500 MHz)
Figure S2: ^{1}H NMR spectrum of **1** (CD_3OD, 500 MHz)
Figure S3: Crystal Structure of **1**
Figure S4: HOMO-LUMO of **1**
Figure S5: Calculated ^{13}C NMR Chemical Shift of **1**
Figure S6: ^{1}H NMR spectrum of **2** (Me_2CO-d_6, 500 MHz)
Figure S7: HOMO-LUMO of **2**
Figure S8: Calculated ^{13}C NMR Chemical Shift of **2**
Figure S9: ^{1}H NMR spectrum of **3** ($CDCl_3$, 500 MHz)
Figure S10: HOMO-LUMO of **3**
Figure S11: Calculated ^{13}C NMR Chemical Shift of **3**
Figure S12: ^{1}H NMR spectrum of **4** (Me_2CO-d_6, 500 MHz)
Figure S13: HOMO-LUMO of **4**
Figure S14: Calculated ^{13}C NMR Chemical Shift of **4**
Figure S15: ^{1}H NMR spectrum of **5** ($CDCl_3$, 500 MHz)
Figure S16: HOMO-LUMO of **5**
Figure S17: Calculated ^{13}C NMR Chemical Shift of **5**
Figure S18: ^{1}H NMR spectrum of **6** (Me_2CO-d_6, 500 MHz)
Figure S19: ^{13}C NMR spectrum of **6** (Me_2CO-d_6, 500 MHz)
Figure S20: ^{1}H-^{13}C HSQC spectrum of **6** (Me_2CO-d_6, 500 MHz)
Figure S21: ^{1}H-^{13}C HMBC spectrum of **6** (Me_2CO-d_6, 500 MHz)
Figure S22: HREIMS of **6**
Figure S23: HOMO-LUMO of **6**
Figure S24: Calculated ^{13}C NMR Chemical Shift of **6**
Figure S25: ^{1}H NMR spectrum of **7** (Me_2CO-d_6, 500 MHz)
Figure S26: HRESIMS of **7**
Figure S27: HOMO-LUMO of **7**
Figure S28: Calculated ^{13}C NMR Chemical Shift of **7**
Figure S29: ^{1}H NMR spectrum of **8** ($CDCl_3$, 500 MHz)
Figure S30: HRESIMS of **8**
Figure S31: Crystal Structure of **8**
Figure S32: HOMO-LUMO of **8**
Figure S33: Calculated ^{13}C NMR Chemical Shift of **8**
Figure S34: ^{1}H NMR spectrum of **9** ($CDCl_3$, 500 MHz)
Figure S35: HRESIMS of **9**
Figure S36: Crystal Structure of **9**
Figure S37: HOMO-LUMO of **9**
Figure S38: Calculated ^{13}C NMR Chemical Shift of **9**
Figure S39: ^{1}H NMR spectrum of **10** (Me_2CO-d_6, 500 MHz)
Figure S40: ^{1}H NMR spectrum of **10** ($CDCl_3$, 500 MHz)
Figure S41: ^{1}H-^{1}H COSY spectrum of **10** ($CDCl_3$, 500 MHz)
Figure S42: ^{1}H-^{13}C HSQC spectrum of **10** ($CDCl_3$, 500 MHz)
Figure S43: ^{1}H-^{13}C HMBC spectrum of **10** ($CDCl_3$, 500 MHz)
Figure S44: HRESIMS of **10**
Figure S45: HOMO-LUMO of **10**
Figure S46: Calculated ^{13}C NMR Chemical Shift of **10**
Figure S47: ^{1}H NMR spectrum of **11** (Me_2CO-d_6, 500 MHz)
Figure S48: HRESIMS of **11**
Figure S49: HOMO-LUMO of **11**
Figure S50: Calculated ^{13}C NMR Chemical Shift of **11**
Figure S51: ^{1}H NMR spectrum of **12** (Me_2CO-d_6, 500 MHz)
Figure S52: ^{1}H NMR spectrum of **12** ($CDCl_3$, 500 MHz)
Figure S53: ^{1}H-^{1}H COSY spectrum of **12** ($CDCl_3$, 500 MHz)
Figure S54: ^{1}H-^{13}C HSQC spectrum of **12** ($CDCl_3$, 500 MHz)
Figure S55: ^{1}H-^{13}C HMBC spectrum of **12** ($CDCl_3$, 500 MHz)
Figure S56: HRESIMS of **12**
Figure S57: HOMO-LUMO of **12**
Figure S58: Calculated ^{13}C NMR Chemical Shift of **12**
Figure S59: ^{1}H NMR spectrum of **13** (Me_2CO-d_6, 500 MHz)
Figure S60: ^{1}H NMR spectrum of **13** ($CDCl_3$, 500 MHz)

Table of Contents

Figure S61: ^{1}H-^{1}H COSY spectrum of **13** ($CDCl_3$, 500 MHz)
Figure S62: ^{1}H-^{13}C HSQC spectrum of **13** ($CDCl_3$, 500 MHz)
Figure S63: ^{1}H-^{13}C HMBC spectrum of **13** ($CDCl_3$, 500 MHz)
Figure S64: ^{1}H-^{13}C HMBC spectrum of **13** ($CDCl_3$, 500 MHz)
Figure S65: HRESIMS of **13**
Figure S66: HOMO-LUMO of **13**
Figure S67: Calculated ^{13}C NMR Chemical Shift of **13**
Figure S68: ^{1}H NMR spectrum of **14** (as a mixture with compound 2) (CD_3OD, 500 MHz)
Figure S69: HOMO-LUMO of **14**
Figure S70: Calculated ^{13}C NMR Chemical Shift of **14**
Figure S71: HOMO-LUMO of **15**
Figure S72: Calculated ^{13}C NMR Chemical Shift of **15**
Figure S73: ^{1}H NMR spectrum of **16** (Me_2CO-d_6, 500 MHz)
Figure S74: ^{1}H NMR spectrum of **16** (Me_2CO-d_6, 500 MHz)
Figure S75: ^{1}H-^{1}H COSY spectrum of **16** (Me_2CO-d_6, 500 MHz)
Figure S76: ^{1}H-^{13}C HSQC spectrum of **16** (Me_2CO-d_6, 500 MHz)
Figure S77: ^{1}H-^{13}C HMBC spectrum of **16** (Me_2CO-d_6, 500 MHz)
Figure S78: HREIMS of **16**
Figure S79: HOMO-LUMO of **16**
Figure S80: Calculated ^{13}C NMR Chemical Shift of **16**
Figure S81: ^{1}H NMR spectrum of **17** (Me_2CO-d_6, 500 MHz)
Figure S82: LREIMS of **17**
Figure S83: HOMO-LUMO of **17**
Figure S84: Calculated ^{13}C NMR Chemical Shift of **17**
Figure S85: ^{1}H NMR spectrum of **18** (Me_2CO-d_6, 500 MHz)
Figure S86: LREIMS of **18**
Figure S87: HOMO-LUMO of **18**
Figure S88: Calculated ^{13}C NMR Chemical Shift of **18**
Figure S89: HOMO-LUMO of **19**
Figure S90: Calculated ^{13}C NMR Chemical Shift of **19**
Figure S91: ^{1}H NMR spectrum of **20** (Me_2CO-d_6, 500 MHz)
Figure S92: ^{1}H NMR spectrum of **20** (CD_3OD, 500 MHz)
Figure S93: HRESIMS of **20**
Figure S94: HOMO-LUMO of **20**
Figure S95: Calculated ^{13}C NMR Chemical Shift of **20**
Figure S96: HOMO-LUMO of **21**
Figure S97: Calculated ^{13}C NMR Chemical Shift of **21**
Figure S98: HOMO-LUMO of **22**
Figure S99: Calculated ^{13}C NMR Chemical Shift of **22**
Figure S100: ^{1}H NMR spectrum of **23** (Me_2CO-d_6, 500 MHz)
Figure S101: HREIMS of **23**
Figure S102: HOMO-LUMO of **23**
Figure S103: Calculated ^{13}C NMR Chemical Shift of **23**
Figure S104: HOMO-LUMO of **24**
Figure S105: Calculated ^{13}C NMR Chemical Shift of **24**
Figure S106: HOMO-LUMO of **25**
Figure S107: Calculated ^{13}C NMR Chemical Shift of **25**
Figure S108: ^{1}H NMR spectrum of **26** (CD_3OD, 500 MHz)
Figure S109: HREIMS of **26**
Figure S110: HOMO-LUMO of **26**
Figure S111: Calculated ^{13}C NMR Chemical Shift of **26**
Figure S112: HOMO-LUMO of **27**
Figure S113: Calculated ^{13}C NMR Chemical Shift of **27**
Figure S114: HOMO-LUMO of **28**
Figure S115: Calculated ^{13}C NMR Chemical Shift of **28**
Figure S116: HOMO-LUMO of **29**
Figure S117: Calculated ^{13}C NMR Chemical Shift of **29**
Figure S118: HOMO-LUMO of **30**
Figure S119: Calculated ^{13}C NMR Chemical Shift of **30**

Table of Contents

Table S1: Crystal Data & Structure Refinement for **1**
Table S2: Fractional Atomic Coordinates ($\times10^4$) and Equivalent Isotropic Displacement Parameters ($Å^2\times10^3$) for **1**. U_{eq} is defined as 1/3 of the trace of the orthogonalized U_{IJ} tensor.
Table S3: Anisotropic Displacement Parameters ($Å^2\times10^3$) for **1**. The anisotropic displacement factor exponent takes the form: $-2\pi^2[h^2a^{*2}U_{11}+2hka^*b^*U_{12}+\ldots]$
Table S4: Bond Lengths for **1**
Table S5: Bond Angles for **1**
Table S6: Torsion Angles for **1**
Table S7: Hydrogen Atom Coordinates ($Å\times10^4$) and Isotropic Displacement Parameters ($Å^2\times10^3$) for **1**
Table S8: Refinement Model Description of **1**
Table S9: Equilibrium Geometry DFT ωB97X-D/6-311+G(2d,p) of **1**
Table S10: Equilibrium Geometry DFT ωB97X-D/6-311+G(2d,p) of **2**
Table S11: Equilibrium Geometry DFT ωB97X-D/6-311+G(2d,p) of **3**
Table S12: Equilibrium Geometry DFT ωB97X-D/6-311+G(2d,p) of **4**
Table S13: Equilibrium Geometry DFT ωB97X-D/6-311+G(2d,p) of **5**
Table S14: Equilibrium Geometry DFT ωB97X-D/6-311+G(2d,p) of **6**
Table S15: Equilibrium Geometry DFT ωB97X-D/6-311+G(2d,p) of **7**
Table S16: Crystal Data & Structure Refinement for **8**
Table S17: Fractional Atomic Coordinates ($\times10^4$) and Equivalent Isotropic Displacement Parameters ($Å^2\times10^3$) for **8**. Ueq is defined as 1/3 of the trace of the orthogonalized UIJ tensor.
Table S18: Anisotropic Displacement Parameters ($Å^2\times10^3$) for **8**. The anisotropic displacement factor exponent takes the form: $-2\pi^2[h^2a^{*2}U_{11}+2hka^*b^*U_{12}+\ldots]$.
Table S19: Bond Lengths for **8**
Table S20: Bond Angles for **8**
Table S21: Torsion Angles for **8**
Table S22: Hydrogen Atom Coordinates ($Å\times10^4$) and Isotropic Displacement Parameters ($Å^2\times10^3$) for **8**
Table S23: Refinement Model Description of **8**
Table S24: Equilibrium Geometry DFT ωB97X-D/6-311+G(2d,p) of **8**
Table S25: Crystal Data and Structure Refinement for **9**
Table S26: Fractional Atomic Coordinates ($\times10^4$) and Equivalent Isotropic Displacement Parameters ($Å^2\times10^3$) for **9**. Ueq is defined as 1/3 of the trace of the orthogonalized UIJ tensor.
Table S27: Anisotropic Displacement Parameters ($Å^2\times10^3$) for **9**. The anisotropic displacement factor exponent takes the form: $-2\pi^2[h^2a^{*2}U_{11}+2hka^*b^*U_{12}+\ldots]$.
Table S28: Bond Lengths for **9**

Table of Contents

Table S29: Bond Angles for **9**
Table S30: Torsion Angles for **9**
Table S31: Hydrogen Atom Coordinates (Å×10^4) and Isotropic Displacement Parameters (Å^2×10^3) for **9**
Table S32: Refinement model description for **9**
Table S33: Equilibrium Geometry DFT ωB97X-D/6-311+G(2d,p) of **9**
Table S34: Equilibrium Geometry DFT ωB97X-D/6-311+G(2d,p) of **10**
Table S35: Equilibrium Geometry DFT ωB97X-D/6-311+G(2d,p) of **11**
Table S36: Equilibrium Geometry DFT ωB97X-D/6-311+G(2d,p) of **12**
Table S37: Equilibrium Geometry DFT ωB97X-D/6-311+G(2d,p) of **13**
Table S38: Equilibrium Geometry DFT ωB97X-D/6-311+G(2d,p) of **14**
Table S39: Equilibrium Geometry DFT ωB97X-D/6-311+G(2d,p) of **15**
Table S40: Equilibrium Geometry DFT ωB97X-D/6-311+G(2d,p) of **16**
Table S41: Equilibrium Geometry DFT ωB97X-D/6-311+G(2d,p) of **17**
Table S42: Equilibrium Geometry DFT ωB97X-D/6-311+G(2d,p) of **18**
Table S43: Equilibrium Geometry DFT ωB97X-D/6-311+G(2d,p) of **19**
Table S44: Equilibrium Geometry DFT ωB97X-D/6-311+G(2d,p) of **20**
Table S45: Equilibrium Geometry DFT ωB97X-D/6-311+G(2d,p) of **21**
Table S46: Equilibrium Geometry DFT ωB97X-D/6-311+G(2d,p) of **22**
Table S47: Equilibrium Geometry DFT ωB97X-D/6-311+G(2d,p) of **23**
Table S48: Equilibrium Geometry DFT ωB97X-D/6-311+G(2d,p) of **24**
Table S49: Equilibrium Geometry DFT ωB97X-D/6-311+G(2d,p) of **25**
Table S50: Equilibrium Geometry DFT ωB97X-D/6-311+G(2d,p) of **26**
Table S51: Equilibrium Geometry DFT ωB97X-D/6-311+G(2d,p) of **27**
Table S52: Equilibrium Geometry DFT ωB97X-D/6-311+G(2d,p) of **28**
Table S53: Equilibrium Geometry DFT ωB97X-D/6-311+G(2d,p) of **29**
Table S54: Equilibrium Geometry DFT ωB97X-D/6-311+G(2d,p) of **30**
Table S55: ^{1}H NMR Data of **1–5**
Table S56: NMR Data of **6** in Me_2CO-d_6
Table S57: ^{1}H NMR Data of **7–9**
Table S58: ^{1}H NMR Data of **10–13**
Table S59: ^{1}H NMR Data of **2**, **14**, **16–18**
Table S60: ^{1}H NMR Data of **20**, **24**, **26**
Table S61: E_{HOMO}, E_{LUMO}, and ΔE for **1–30**

Figure S1. ^{1}H NMR spectrum of **1** (Me_2CO-d_6, 500 MHz)

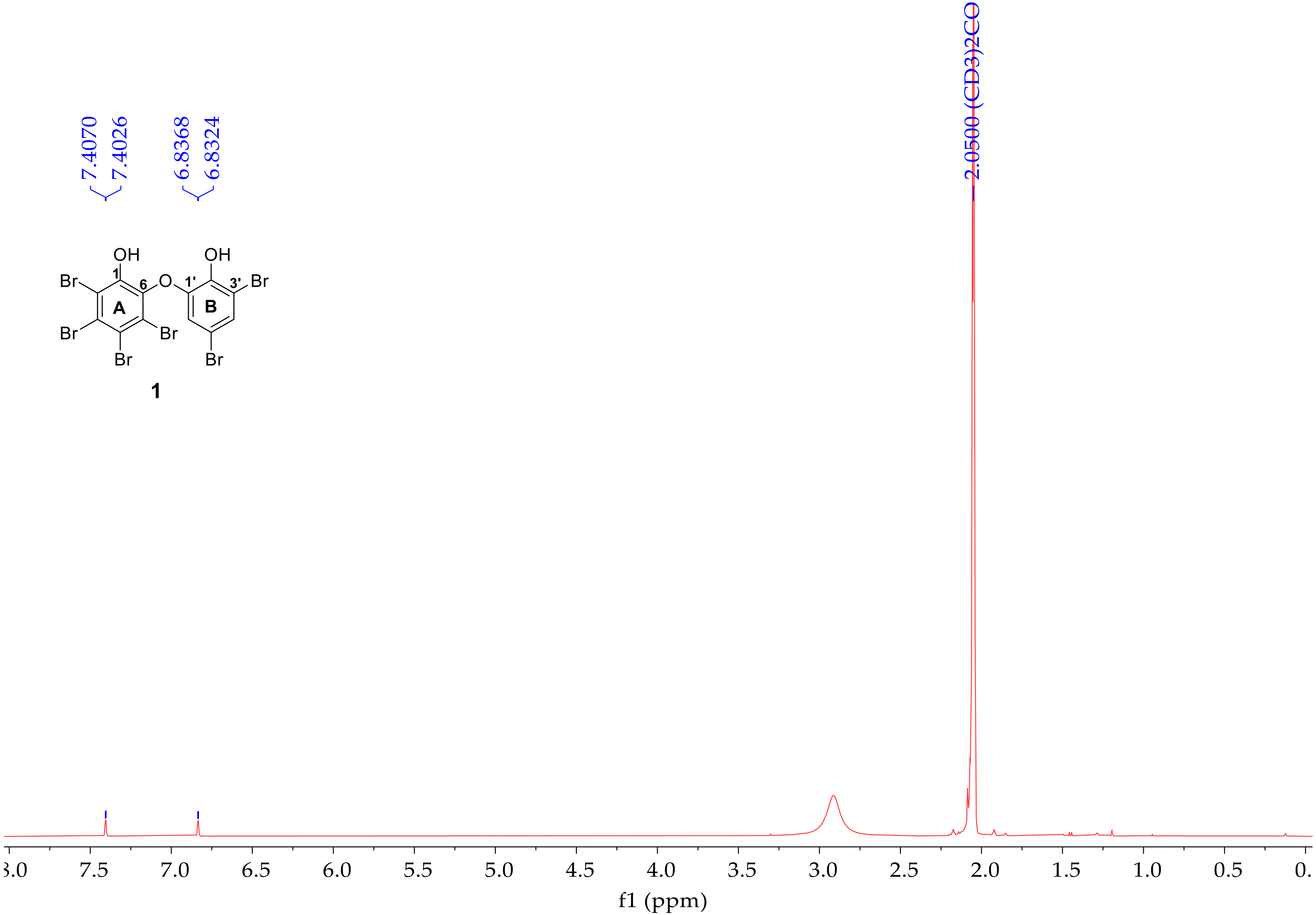

Figure S2. ^{1}H NMR spectrum of **1** (CD_3OD, 500 MHz)

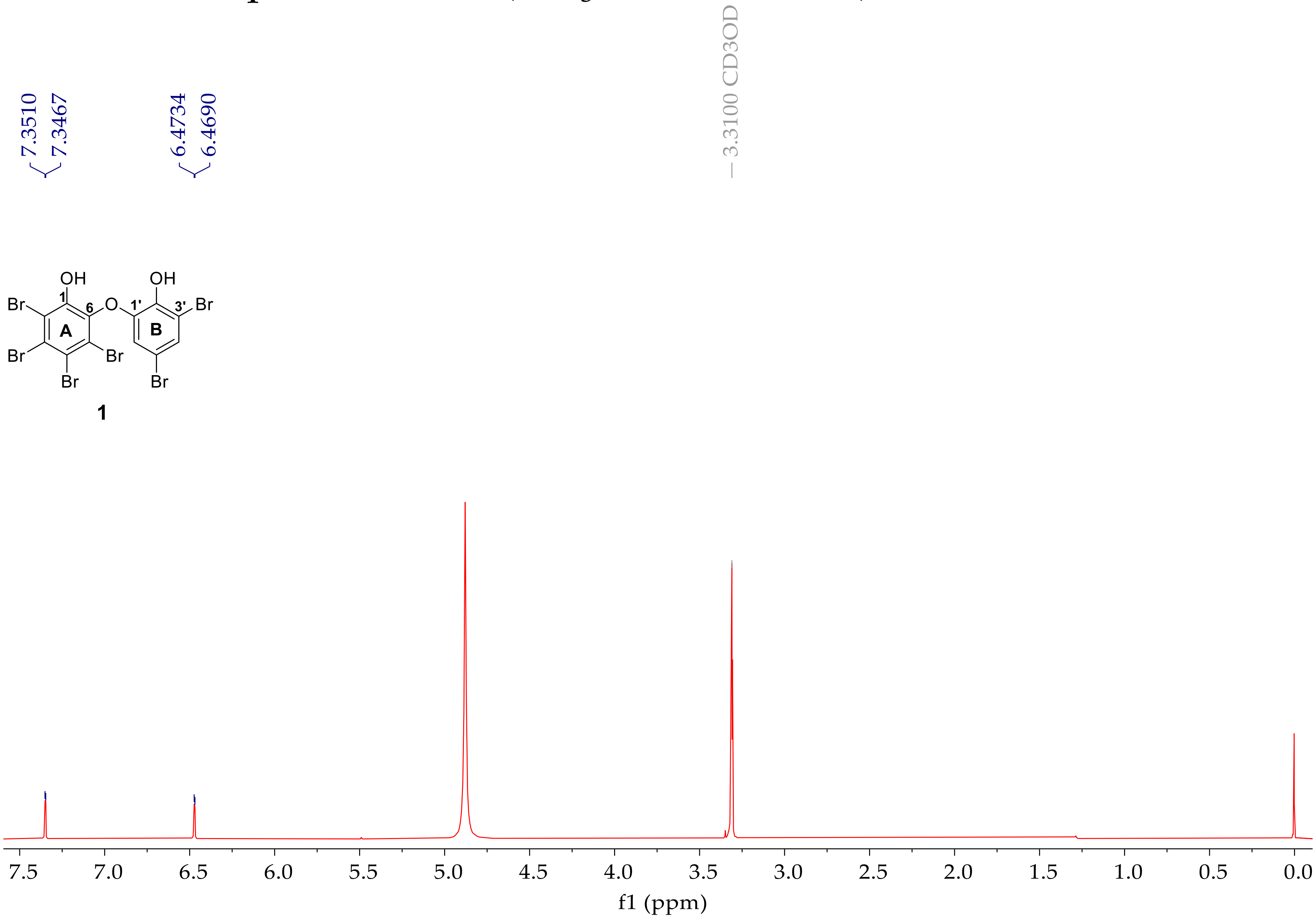

Figure S3. Crystal Structure of **1**

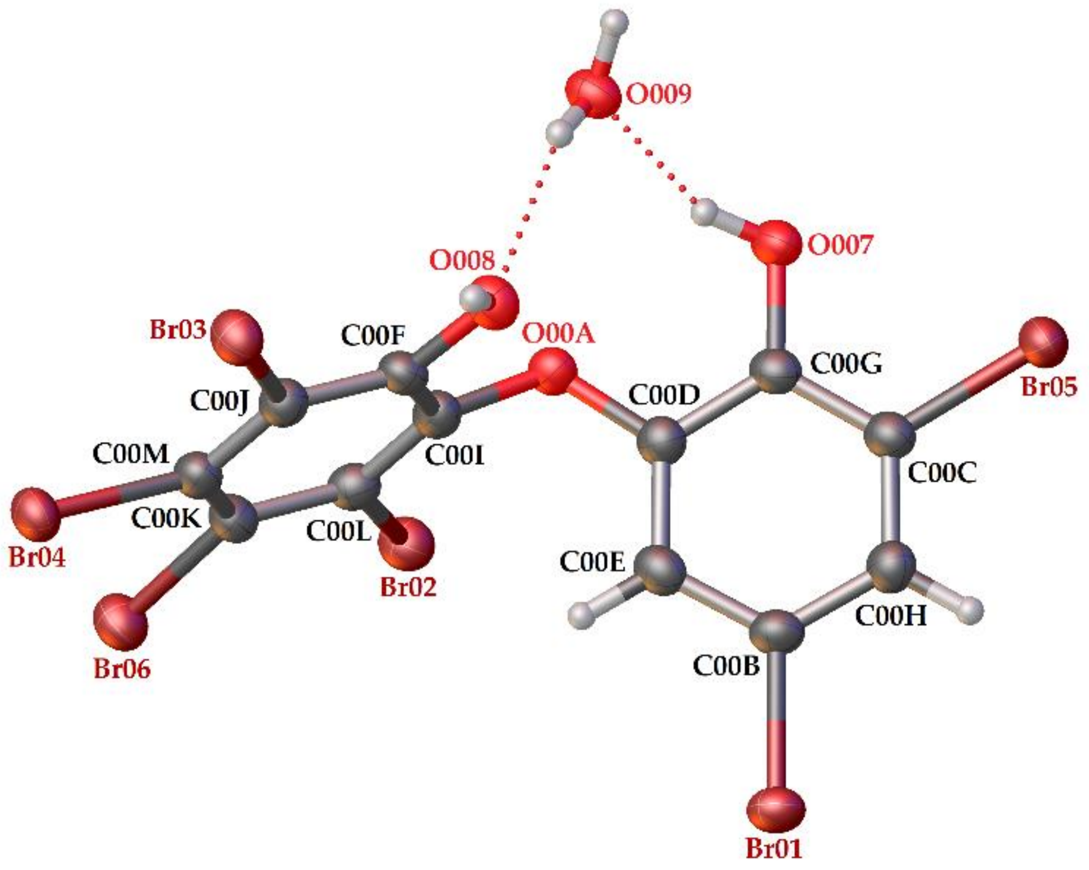

2, 3, 4, 5-tetrabromo-6-(3', 5'-dibromo-2'-hydroxyphenoxy) phenol

Table S1. Crystal Data & Structure Refinement for **1**

Identification code	180726irai
Empirical formula	$C_{12}H_6Br_6O_4$
Formula weight	693.63
Temperature/K	123.15
Crystal system	monoclinic
Space group	$P2_1/n$
a/Å	4.74979(10)
b/Å	18.7360(3)
c/Å	18.3463(4)
α/°	90
β/°	91.6135(19)
γ/°	90
Volume/Å^3	1632.02(6)
Z	4
ρ_{calc}g/cm^3	2.823
μ/mm^{-1}	14.772
F(000)	1280.0
Crystal size/mm^3	0.281 × 0.279 × 0.221
Radiation	MoKα (λ = 0.71073)
2Θ range for data collection/°	3.108 to 62.354
Index ranges	-6 ≤ h ≤ 6, -25 ≤ k ≤ 25, -26 ≤ l ≤ 25
Reflections collected	20780
Independent reflections	4898 [R_{int} = 0.0374, R_{sigma} = 0.0252]
Data/restraints/parameters	4898/0/208
Goodness-of-fit on F^2	1.086
Final R indexes [I>=2σ (I)]	R_1 = 0.0435, wR_2 = 0.1179
Final R indexes [all data]	R_1 = 0.0526, wR_2 = 0.1269
Largest diff. peak/hole / e Å^{-3}	1.42/-0.77

Table S2. Fractional Atomic Coordinates (×10^4) and Equivalent Isotropic Displacement Parameters (Å^2×10^3) for **1**. U_{eq} is defined as 1/3 of the trace of the orthogonalized U_{IJ} tensor.

Atom	*x*	*y*	*z*	U(eq)
Br01	-227.5(9)	8111.4(2)	6204.8(3)	34.75(12)
Br02	7616.6(9)	6700.4(2)	8071.2(3)	34.93(12)
Br03	-790.8(10)	3995.6(3)	7420.9(3)	34.86(12)
Br04	-89.4(10)	4628.6(2)	9099.8(2)	34.71(12)
Br05	6901.4(10)	6977.5(3)	4062.1(3)	36.74(13)
Br06	4059.1(11)	5999.0(3)	9432.1(3)	37.98(13)
O00A	6453(6)	5900.9(16)	6644.2(17)	29.8(6)
O007	8319(7)	5945.1(17)	5269.5(19)	34.9(7)
O008	2752(7)	4874.0(18)	6353.4(17)	33.3(6)
C00B	2409(9)	7428(2)	5905(2)	30.4(8)
C00C	5445(9)	6955(2)	5004(3)	31.1(8)
C00D	5283(9)	6417(2)	6184(2)	29.1(8)
C00E	3283(9)	6900(2)	6398(3)	31.4(8)
C00F	2945(9)	5093(2)	7060(2)	28.8(8)
C00G	6383(9)	6428(2)	5484(2)	29.7(8)
C00H	3461(9)	7463(2)	5213(3)	32.7(9)
C00I	4826(9)	5659(2)	7212(2)	29.7(8)
C00J	1528(9)	4788(2)	7626(2)	29.3(8)
C00K	3673(9)	5619(2)	8478(2)	30.9(8)
C00L	5196(9)	5926(2)	7910(2)	29.6(8)
C00M	1877(9)	5049(2)	8338(2)	31.0(8)
O009	7827(7)	4570.4(18)	5686.3(19)	33.8(7)

Table S3. Anisotropic Displacement Parameters ($Å^2×10^3$) for **1**.
The anisotropic displacement factor exponent takes the form:
$-2\pi^2[h^2a^{*2}U_{11}+2hka^*b^*U_{12}+\ldots]$

Atom	U_{11}	U_{22}	U_{33}	U_{23}	U_{13}	U_{12}
Br01	31.5(2)	30.9(2)	42.1(3)	-1.96(17)	6.89(17)	4.77(16)
Br02	33.7(2)	33.4(2)	37.9(2)	-2.53(17)	4.49(17)	-4.54(17)
Br03	36.4(2)	34.0(2)	34.4(2)	-0.82(16)	6.55(17)	-7.00(17)
Br04	38.1(2)	35.6(2)	30.9(2)	1.45(16)	9.31(17)	-2.63(17)
Br05	44.1(3)	34.4(2)	32.3(2)	2.96(16)	10.48(18)	4.25(18)
Br06	43.6(3)	38.1(3)	32.4(2)	-3.13(17)	5.03(18)	-4.09(19)
O00A	26.9(14)	30.5(14)	32.2(15)	2.5(12)	6.2(11)	1.3(11)
O007	39.7(17)	29.3(15)	36.2(17)	1.9(12)	12.3(13)	4.3(13)
O008	30.4(15)	36.2(16)	33.5(16)	-1.4(13)	3.7(12)	-4.4(13)
C00B	25.9(18)	29(2)	37(2)	-2.7(16)	4.4(15)	1.9(15)
C00C	32(2)	29(2)	33(2)	-1.1(16)	6.9(16)	-0.1(16)
C00D	26.7(18)	28.7(19)	32(2)	1.2(15)	4.0(15)	-2.0(15)
C00E	29.5(19)	32(2)	33(2)	-2.3(16)	7.3(16)	-3.5(16)
C00F	28.2(18)	29.7(19)	28.6(19)	-2.4(15)	5.7(15)	2.8(15)
C00G	30.0(19)	26.3(18)	33(2)	-2.4(15)	5.7(15)	-1.0(15)
C00H	32(2)	32(2)	34(2)	3.7(17)	4.3(16)	-0.1(17)
C00I	26.7(18)	29.4(19)	33(2)	3.7(16)	6.2(15)	1.4(15)
C00J	28.6(18)	27.6(19)	32(2)	1.2(15)	2.8(15)	-0.5(15)
C00K	33(2)	32(2)	27.5(19)	-1.5(16)	4.4(15)	1.6(17)
C00L	26.0(18)	26.6(19)	36(2)	-1.1(16)	2.6(15)	-0.4(15)
C00M	31(2)	31(2)	32(2)	2.2(16)	7.2(16)	3.7(16)
O009	32.8(16)	33.0(16)	36.0(17)	-4.2(13)	9.6(13)	-1.7(13)

Table S4. Bond Lengths for **1**

Atom	Atom	Length/Å
Br01	C00B	1.884(4)
Br02	C00L	1.869(4)
Br03	C00J	1.881(4)
Br04	C00M	1.876(4)
Br05	C00C	1.880(5)
Br06	C00K	1.893(4)
O00A	C00D	1.389(5)
O00A	C00I	1.390(5)
O007	C00G	1.357(5)
O008	C00F	1.361(5)
C00B	C00E	1.396(6)
C00B	C00H	1.380(6)
C00C	C00G	1.387(6)
C00C	C00H	1.400(6)
C00D	C00E	1.378(6)
C00D	C00G	1.400(6)
C00F	C00I	1.410(6)
C00F	C00J	1.377(6)
C00I	C00L	1.382(6)
C00J	C00M	1.400(6)
C00K	C00L	1.408(6)
C00K	C00M	1.386(6)

Table S5. Bond Angles for **1**

Atom	Atom	Atom	Angle/°
C00D	O00A	C00I	117.5(3)
C00E	C00B	Br01	118.7(3)
C00H	C00B	Br01	119.8(3)
C00H	C00B	C00E	121.5(4)
C00G	C00C	Br05	118.6(3)
C00G	C00C	C00H	121.1(4)
C00H	C00C	Br05	120.3(3)
O00A	C00D	C00G	114.6(4)
C00E	C00D	O00A	123.4(4)
C00E	C00D	C00G	122.0(4)
C00D	C00E	C00B	118.4(4)
O008	C00F	C00I	116.1(4)
O008	C00F	C00J	124.9(4)
C00J	C00F	C00I	118.9(4)
O007	C00G	C00C	120.0(4)
O007	C00G	C00D	121.9(4)
C00C	C00G	C00D	118.1(4)

Atom	Atom	Atom	Angle/°
C00B	C00H	C00C	118.9(4)
O00A	C00I	C00F	117.4(4)
C00L	C00I	O00A	121.2(4)
C00L	C00I	C00F	121.2(4)
C00F	C00J	Br03	118.2(3)
C00F	C00J	C00M	120.8(4)
C00M	C00J	Br03	121.1(3)
C00L	C00K	Br06	119.5(3)
C00M	C00K	Br06	120.3(3)
C00M	C00K	C00L	120.3(4)
C00I	C00L	Br02	119.2(3)
C00I	C00L	C00K	118.9(4)
C00K	C00L	Br02	121.8(3)
C00J	C00M	Br04	120.0(3)
C00K	C00M	Br04	120.2(3)
C00K	C00M	C00J	119.9(4)

Table S6. Torsion Angles for **1**

A	B	C	D	Angle/˚
Br01	C00B	C00E	C00D	-177.8(3)
Br01	C00B	C00H	C00C	179.2(3)
Br03	C00J	C00M	Br04	-2.3(5)
Br03	C00J	C00M	C00K	177.8(3)
Br05	C00C	C00G	O007	0.2(6)
Br05	C00C	C00G	C00D	-179.9(3)
Br05	C00C	C00H	C00B	179.3(3)
Br06	C00K	C00L	Br02	0.3(5)
Br06	C00K	C00L	C00I	-177.9(3)
Br06	C00K	C00M	Br04	-2.2(5)
Br06	C00K	C00M	C00J	177.7(3)
O00A	C00D	C00E	C00B	175.4(4)
O00A	C00D	C00G	O007	3.5(6)
O00A	C00D	C00G	C00C	-176.3(4)
O00A	C00I	C00L	Br02	7.4(6)
O00A	C00I	C00L	C00K	-174.4(4)
O008	C00F	C00I	O00A	-4.9(6)
O008	C00F	C00I	C00L	-180.0(4)
O008	C00F	C00J	Br03	1.6(6)
O008	C00F	C00J	C00M	179.6(4)
C00D	O00A	C00I	C00F	86.5(5)
C00D	O00A	C00I	C00L	-98.5(5)
C00E	C00B	C00H	C00C	0.1(7)
C00E	C00D	C00G	O007	-178.9(4)
C00E	C00D	C00G	C00C	1.3(7)
C00F	C00I	C00L	Br02	-177.7(3)
C00F	C00I	C00L	C00K	0.5(7)
C00F	C00J	C00M	Br04	179.7(3)
C00F	C00J	C00M	C00K	-0.2(7)
C00G	C00C	C00H	C00B	-0.8(7)
C00G	C00D	C00E	C00B	-2.0(7)
C00H	C00B	C00E	C00D	1.3(7)
C00H	C00C	C00G	O007	-179.6(4)
C00H	C00C	C00G	C00D	0.2(7)
C00I	O00A	C00D	C00E	27.7(6)
C00I	O00A	C00D	C00G	-154.8(4)
C00I	C00F	C00J	Br03	-176.1(3)
C00I	C00F	C00J	C00M	1.9(6)
C00J	C00F	C00I	O00A	173.0(4)
C00J	C00F	C00I	C00L	-2.1(6)
C00L	C00K	C00M	Br04	178.7(3)
C00L	C00K	C00M	C00J	-1.5(7)
C00M	C00K	C00L	Br02	179.4(3)
C00M	C00K	C00L	C00I	1.3(7)

Table S7. Hydrogen Atom Coordinates (Å×10^4) and Isotropic Displacement Parameters (Å^2×10^3) for **1**

**Atom**	*x*	*y*	*z*	**U(eq)**
H007	8230	5579	5532	52
H008	1318	4616	6292	50
H00E	2518	6874	6871	38
H00H	2849	7826	4883	39
H00A	6146	4500	5816	51
H00B	8140(110)	4250(30)	5380(30)	31(13)

Table S8. Refinement Model Description of **1**

Number of restraints - 0, number of constraints - unknown.

Details:

1. Fixed Uiso

   At 1.2 times of: All C(H) groups

   At 1.5 times of: All O(H) groups

2a. Free rotating group: O009(H00A)

2b. Aromatic/amide H refined with riding coordinates: C00E(H00E), C00H(H00H)

2c. Idealized tetrahedral OH refined as rotating group: O007(H007), O008(H008)

Table S9. Equilibrium Geometry DFT ωB97X-D/6-311+G(2d,p) of **1**

```
SPARTAN'20 Quantum Mechanics Driver:  (Win/64b)          Release  1.0.0

Job type: Geometry optimization.
Method: RWB97X-D
Basis set: 6-311+G(2D,P)
Number of basis functions: 717
Number of electrons: 310
Parallel Job: 16 threads

SCF model:
 A restricted hybrid HF-DFT SCF calculation will be
 performed using Pulay DIIS + Geometric Direct Minimization


Optimization:
        Step      Energy           Max Grad.      Max Dist.
           1   -16130.324682       0.029469       0.075204
           2   -16130.331007       0.007593       0.063575
           3   -16130.331542       0.001578       0.004060
           4   -16130.331567       0.000786       0.008361
           5   -16130.331582       0.000337       0.001851

  Reason for exit: Successful completion
  Quantum Calculation CPU Time :   5:01:41.34
  Quantum Calculation Wall Time:     20:20.21
```

Figure S4. HOMO-LUMO of **1**

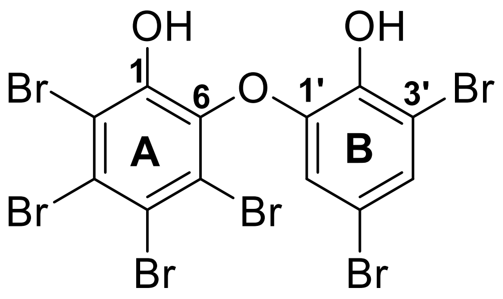

1

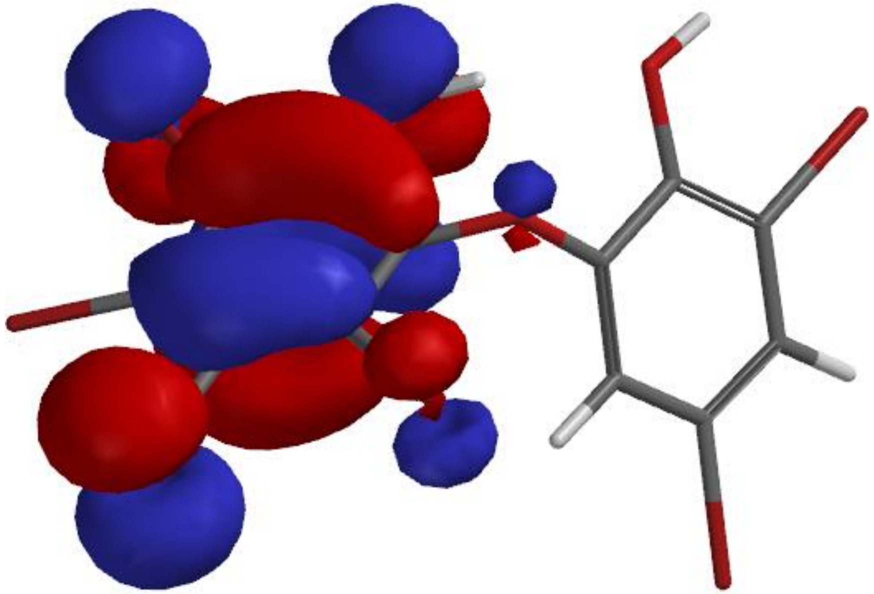

HOMO 1

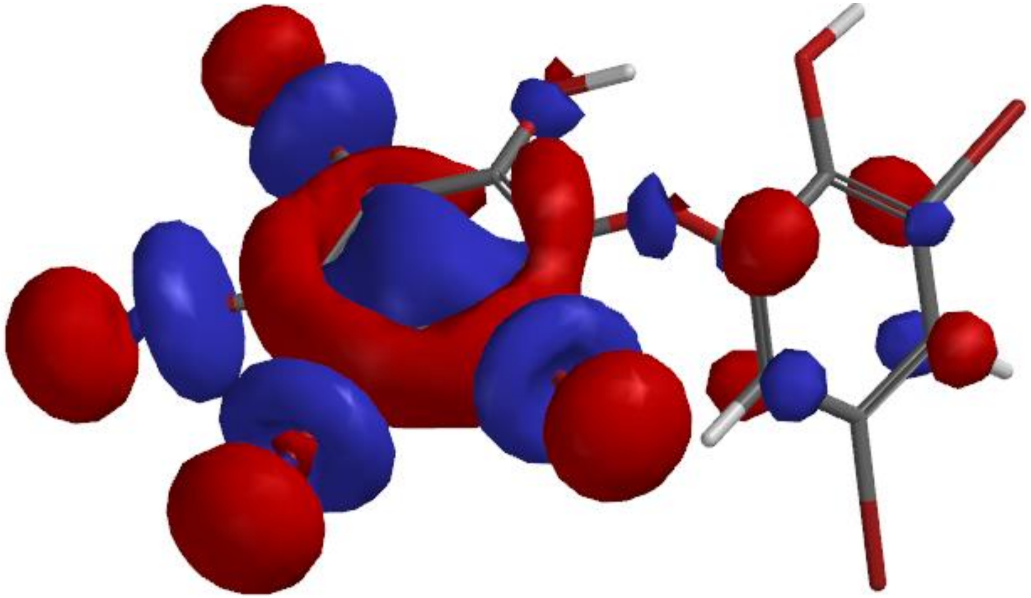

LUMO 1

Figure S5. Calculated ^{13}C NMR Chemical Shift of **1**

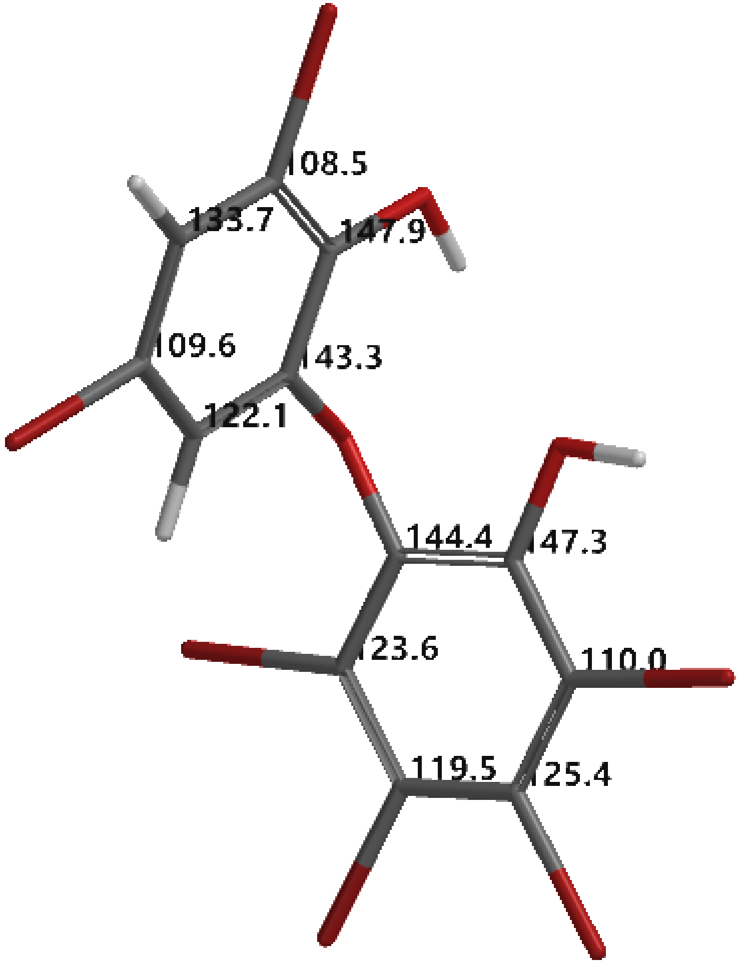

Figure S6. ^{1}H NMR spectrum of **2** (Me_2CO-d_6, 500 MHz)

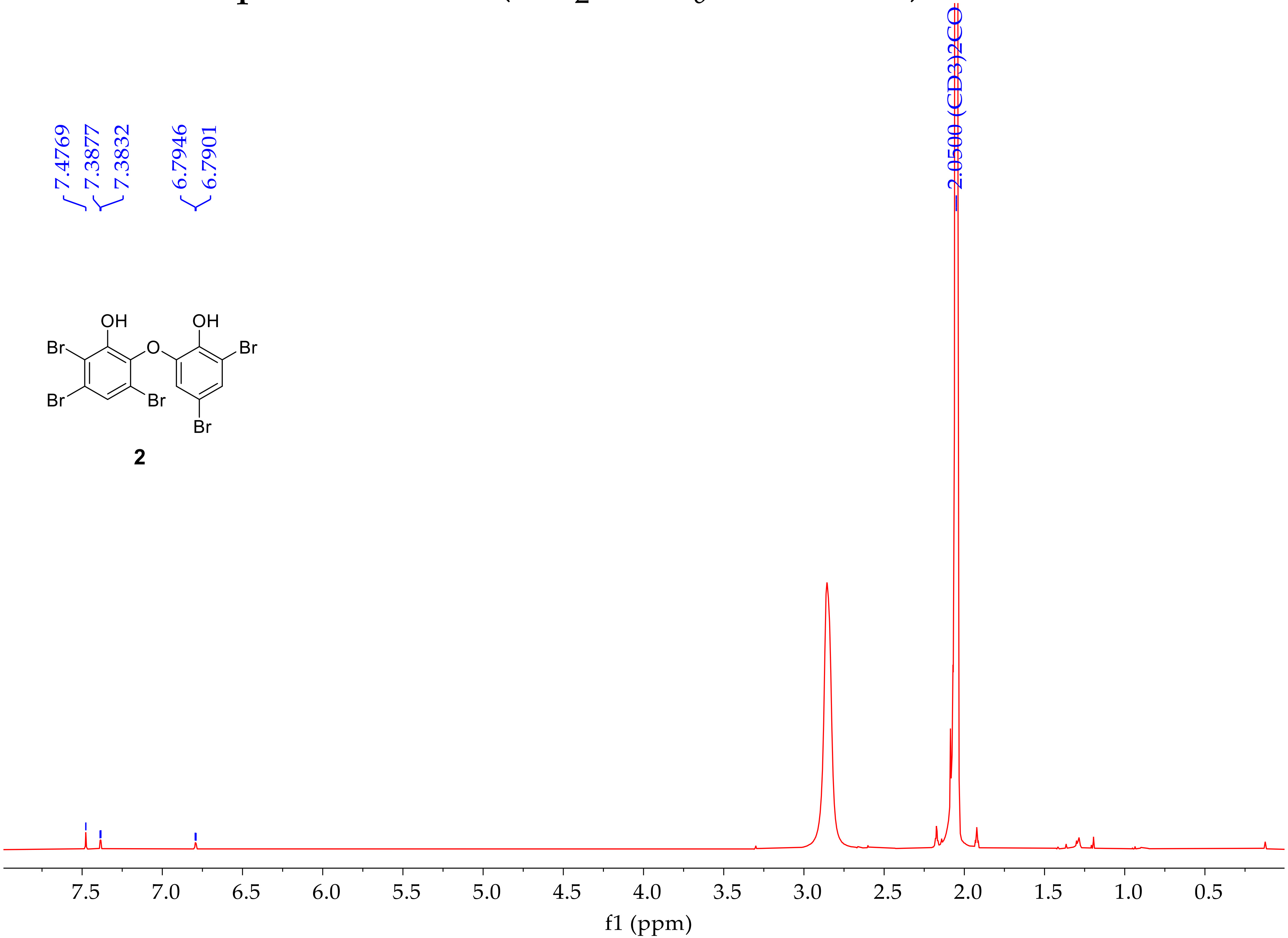

Table S10. Equilibrium Geometry DFT ωB97X-D/6-311+G(2d,p) of **2**

```
Job type: Geometry optimization.
Method: RWB97X-D
Basis set: 6-311+G(2D,P)
Number of basis functions: 675
Number of electrons: 276
Parallel Job: 16 threads

SCF model:
 A restricted hybrid HF-DFT SCF calculation will be
 performed using Pulay DIIS + Geometric Direct Minimization


Optimization:
         Step      Energy           Max Grad.       Max Dist.
            1   -13556.767302       0.022717        0.081068
            2   -13556.772247       0.007810        0.053125
            3   -13556.772709       0.001135        0.001814
            4   -13556.772720       0.000908        0.013479
            5   -13556.772746       0.000390        0.002851
            6   -13556.772747       0.000208        0.001519
```

Figure S7. HOMO-LUMO of **2**

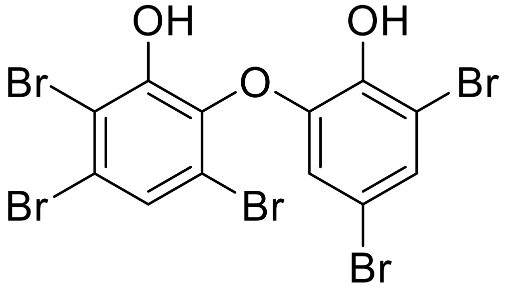

2

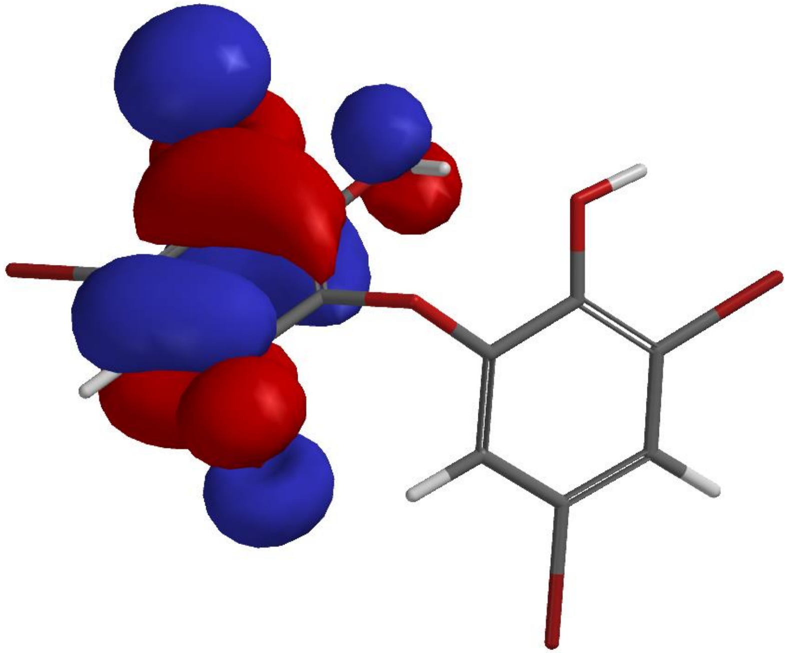

HOMO 2

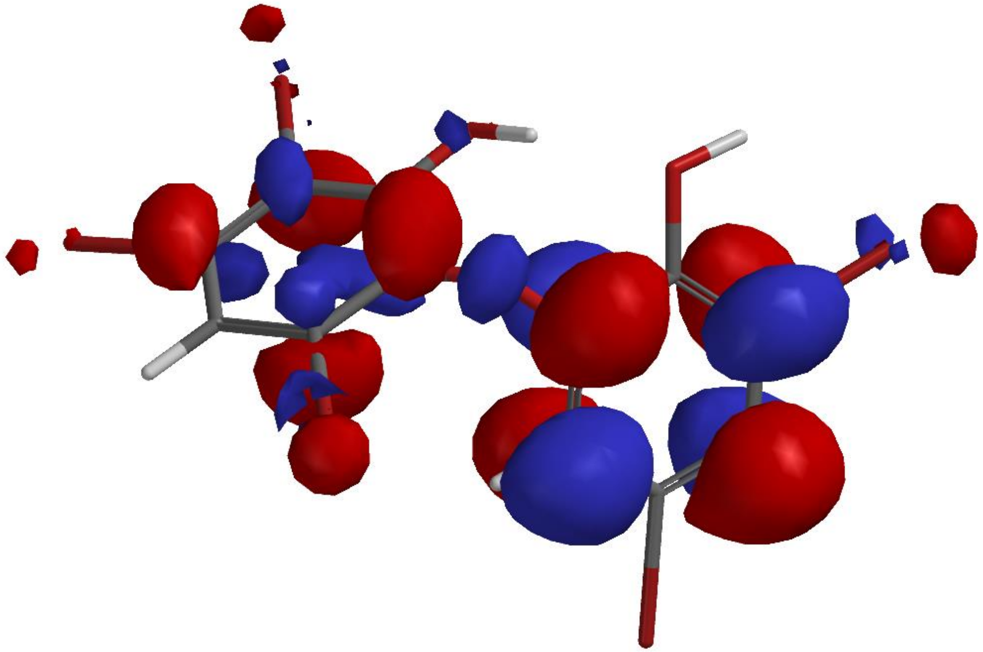

LUMO 2

Figure S8. Calculated ^{13}C NMR Chemical Shift of **2**

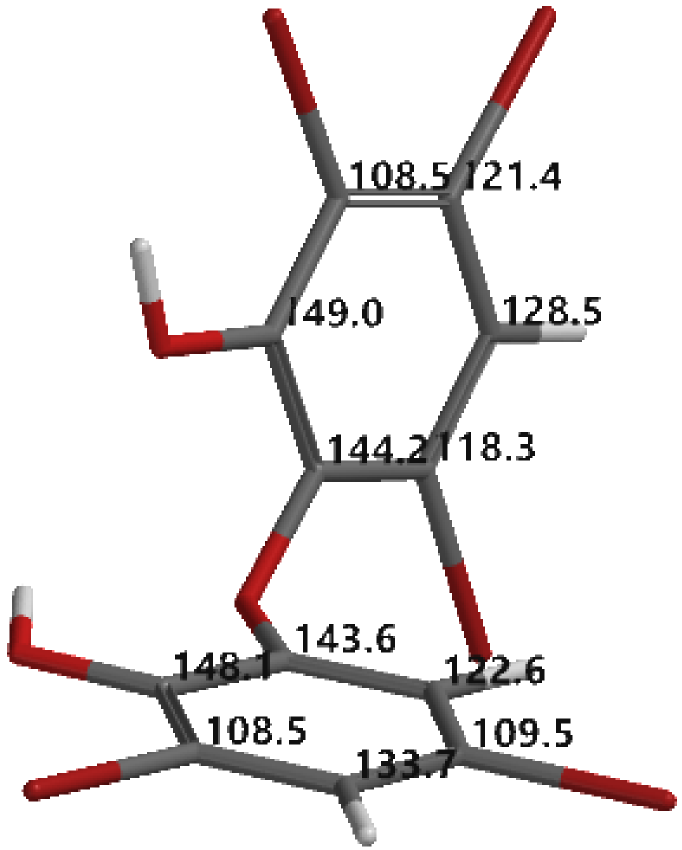

Figure S9. ^{1}H NMR spectrum of **3** ($CDCl_3$, 500 MHz)

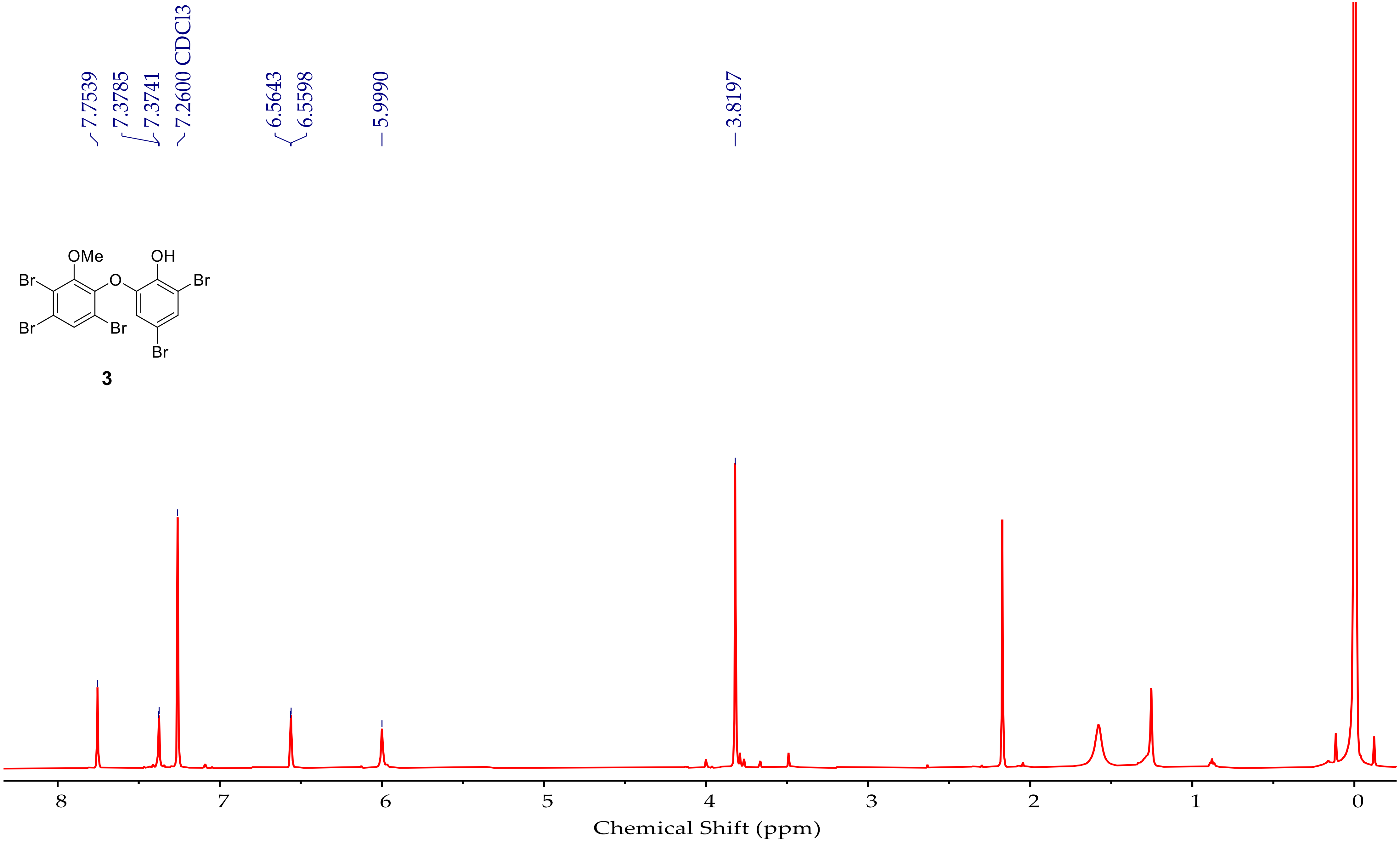

Table S11. Equilibrium Geometry DFT ωB97X-D/6-311+G(2d,p) of **3**

```
Job type: Geometry optimization.
Method: RWB97X-D
Basis set: 6-311+G(2D,P)
Number of basis functions: 714
Number of electrons: 284
Parallel Job: 16 threads

SCF model:
 A restricted hybrid HF-DFT SCF calculation will be
 performed using Pulay DIIS + Geometric Direct Minimization


Optimization:
         Step       Energy          Max Grad.       Max Dist.
            1   -13596.053627       0.022713        0.083976
            2   -13596.059595       0.007325        0.138126
            3   -13596.060478       0.001646        0.025831
            4   -13596.060560       0.001320        0.153568
            5   -13596.060814       0.001554        0.062408
            6   -13596.060876       0.001640        0.049467
            7   -13596.060903       0.001004        0.038755
            8   -13596.060924       0.000578        0.008867
            9   -13596.060929       0.000183        0.003442
```

Figure S10. HOMO-LUMO of **3**

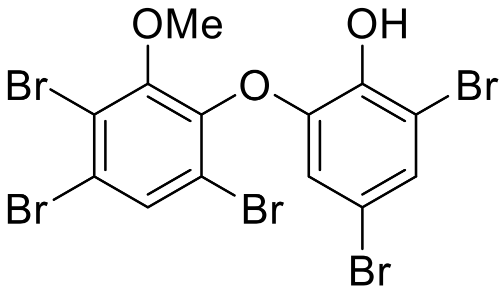

3

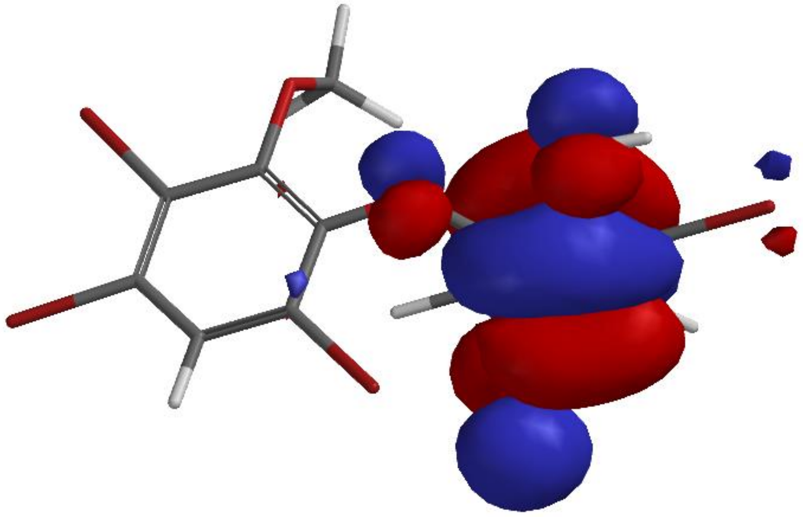

HOMO 3

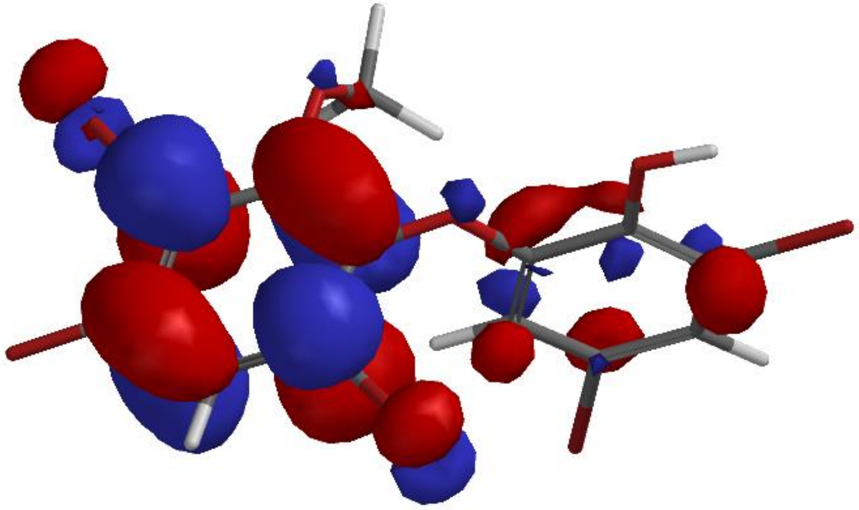

LUMO 3

Figure S11. Calculated ^{13}C NMR Chemical Shift of **3**

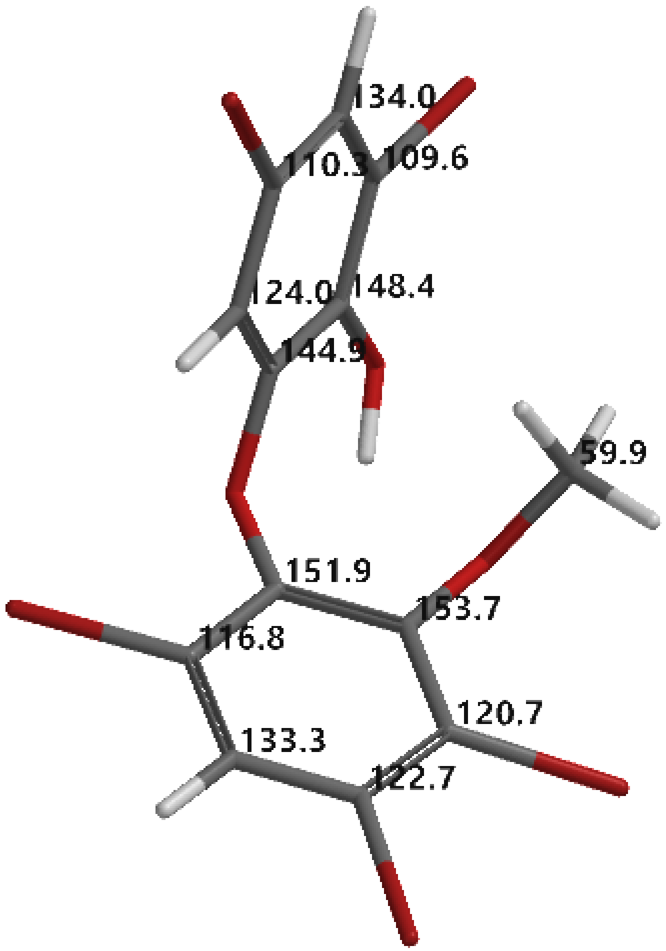

Figure S12. ^{1}H NMR spectrum of **4** (Me_2CO-d_6, 500 MHz)

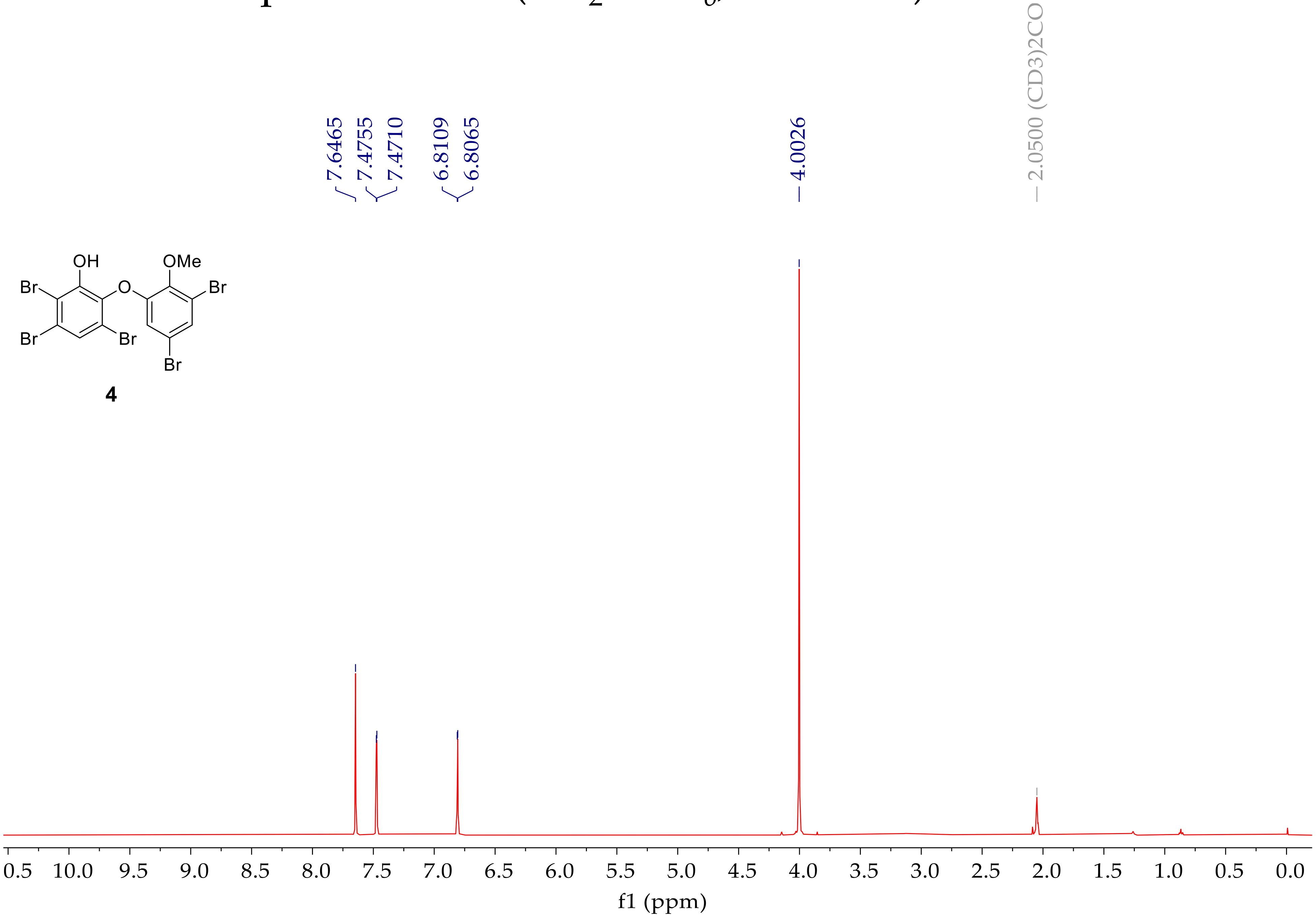

Table S12. Equilibrium Geometry DFT ωB97X-D/6-311+G(2d,p) of **4**

```
Job type: Geometry optimization.
Method: RWB97X-D
Basis set: 6-311+G(2D,P)
Number of basis functions: 714
Number of electrons: 284
Parallel Job: 16 threads

SCF model:
 A restricted hybrid HF-DFT SCF calculation will be
 performed using Pulay DIIS + Geometric Direct Minimization


Optimization:
         Step       Energy           Max Grad.       Max Dist.
            1   -13596.056101        0.022110        0.075185
            2   -13596.061698        0.008615        0.116440
            3   -13596.062567        0.003925        0.073192
            4   -13596.062888        0.004495        0.088281
            5   -13596.063157        0.003611        0.080883
            6   -13596.063326        0.001687        0.060938
            7   -13596.063428        0.001079        0.119373
            8   -13596.063575        0.003974        0.042723
            9   -13596.063618        0.003396        0.071338
           10   -13596.063520        0.005150        0.048847
           11   -13596.063706        0.000446        0.010202
           12   -13596.063710        0.000324        0.001582
```

Figure S13. HOMO-LUMO of **4**

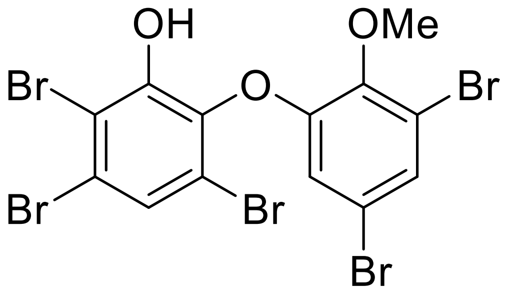

4

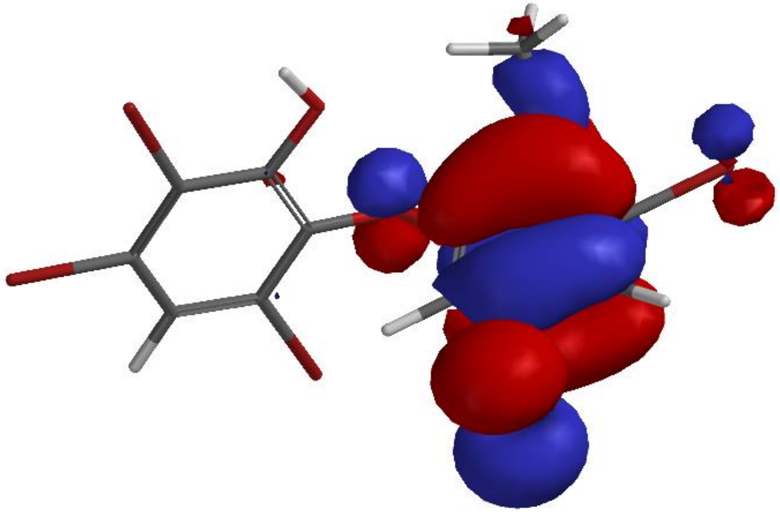

HOMO 4

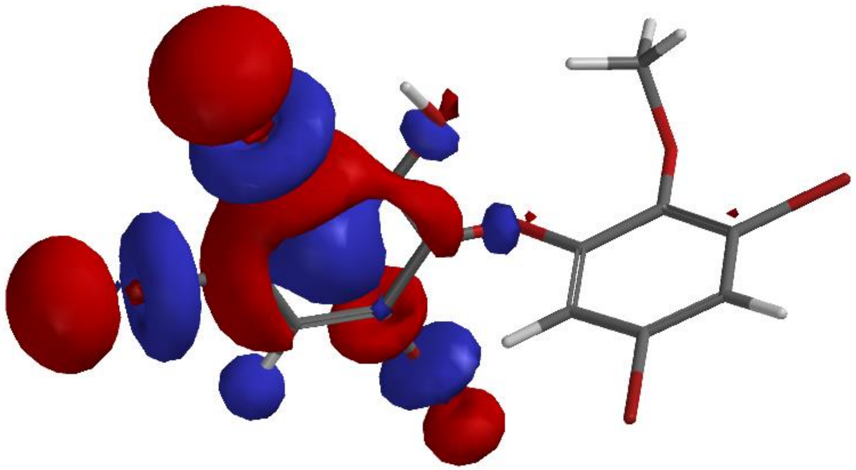

LUMO 4

Figure S14. Calculated ^{13}C NMR Chemical Shift of **4**

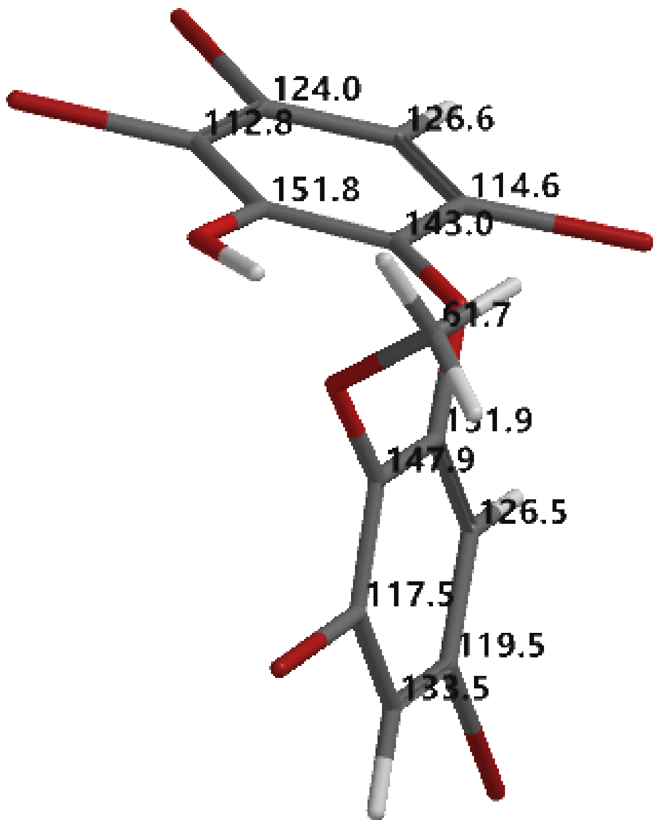

Figure S15. ^1H NMR spectrum of **5** ($CDCl_3$, 500 MHz)

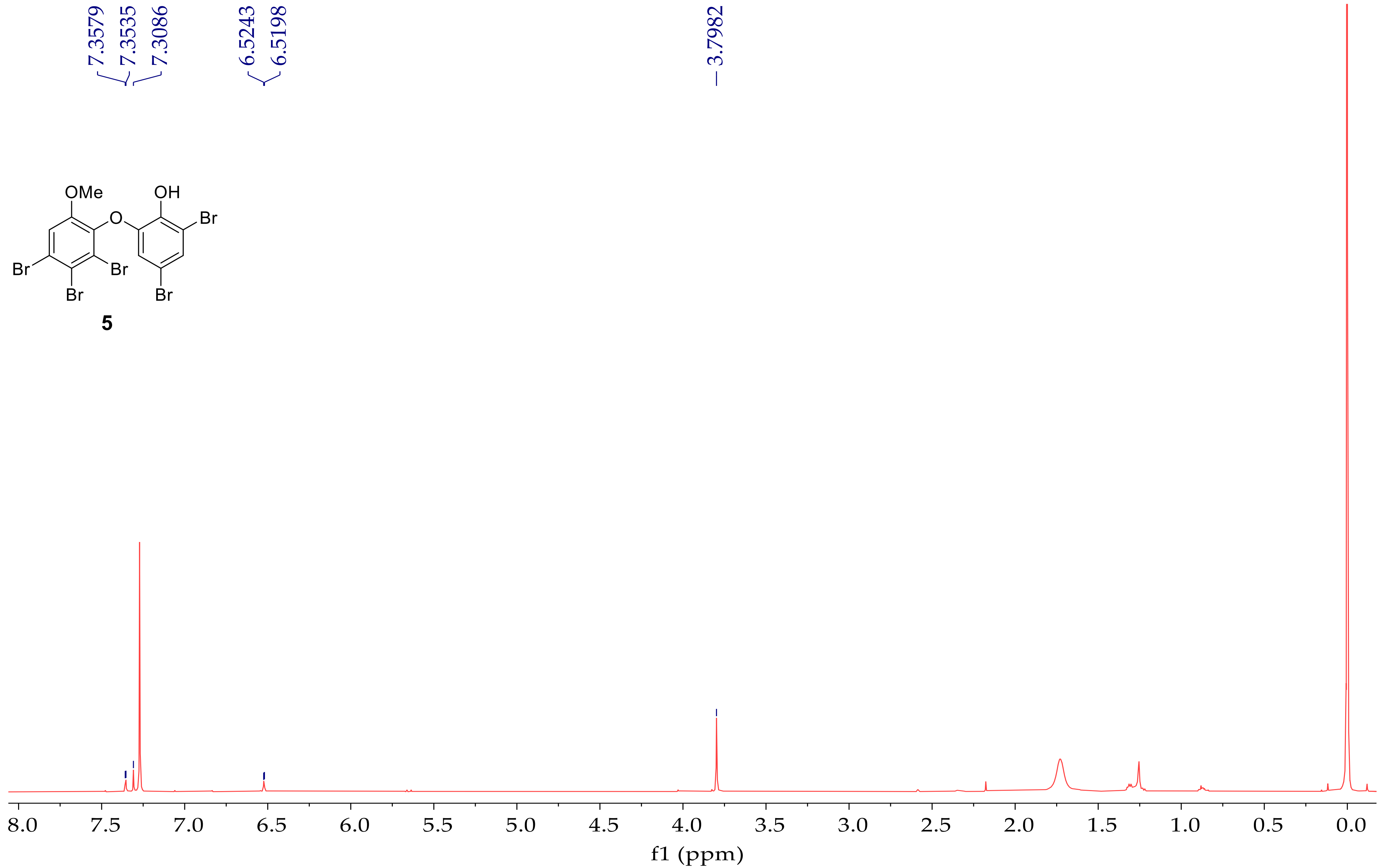

Table S13. Equilibrium Geometry DFT ωB97X-D/6-311+G(2d,p) of **5**

```
Job type: Geometry optimization.
Method: RWB97X-D
Basis set: 6-311+G(2D,P)
Number of basis functions: 714
Number of electrons: 284
Parallel Job: 16 threads

SCF model:
 A restricted hybrid HF-DFT SCF calculation will be
 performed using Pulay DIIS + Geometric Direct Minimization


Optimization:
         Step       Energy            Max Grad.       Max Dist.
            1   -13596.053210         0.024364        0.077730
            2   -13596.058728         0.007035        0.047576
            3   -13596.059286         0.002251        0.011393
            4   -13596.059338         0.002116        0.055522
            5   -13596.059466         0.000895        0.037523
            6   -13596.059496         0.001018        0.008389
            7   -13596.059503         0.000458        0.008994
```

Figure S16. HOMO-LUMO of **5**

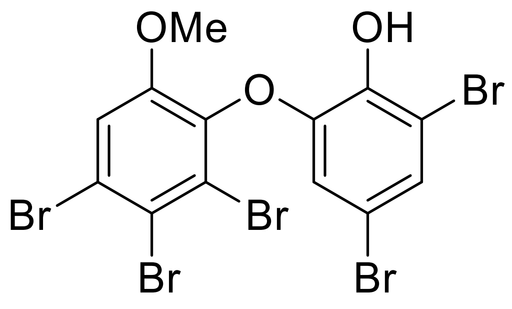

5

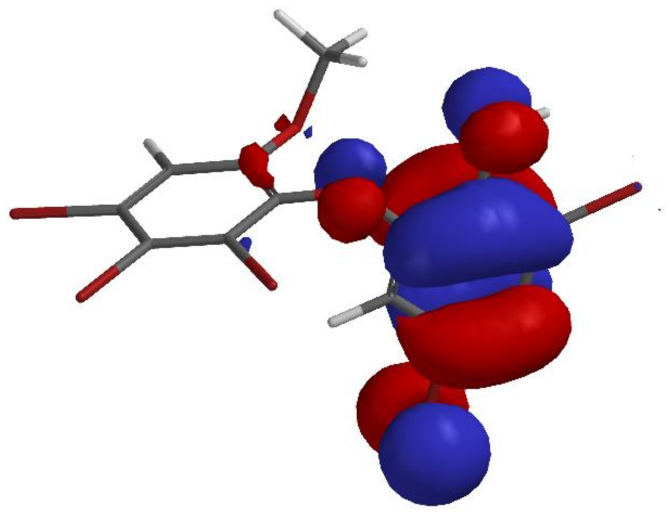

HOMO 5

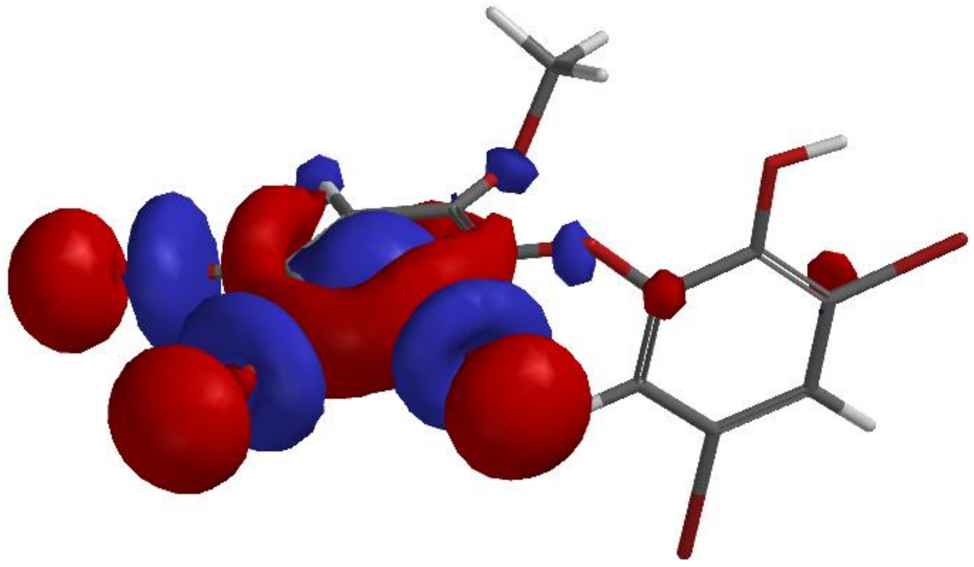

LUMO 5

Figure S17. Calculated ^{13}C NMR Chemical Shift of **5**

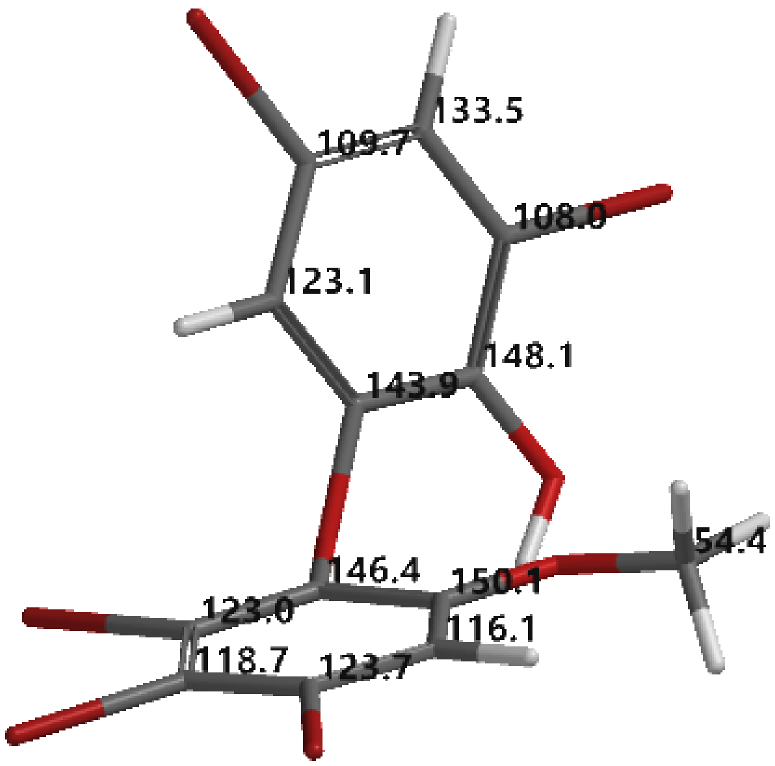

Figure S18. ^{1}H NMR spectrum of **6** (Me_2CO-d_6, 500 MHz)

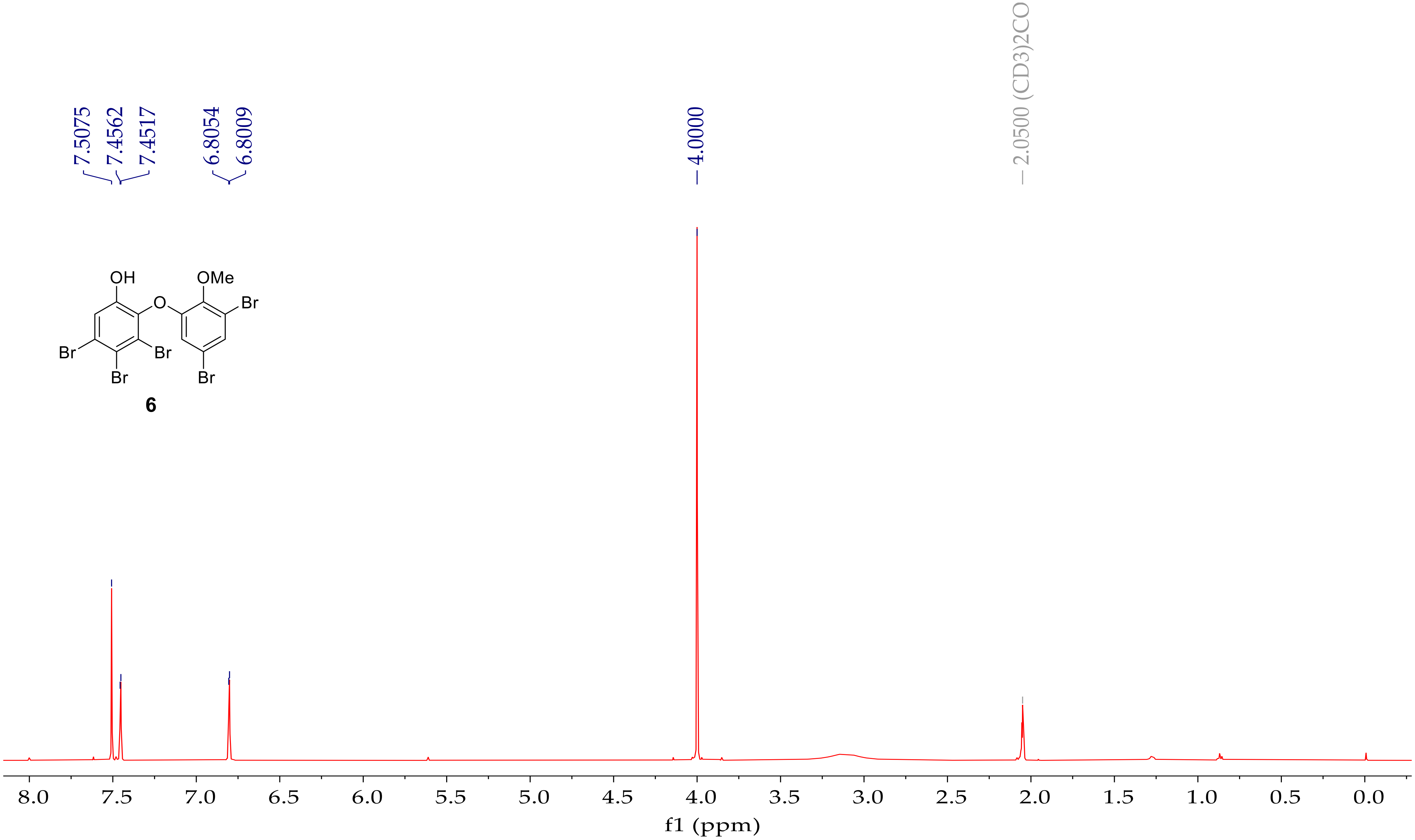

Figure S19. ^{13}C NMR spectrum of **6** (Me_2CO-d_6, 500 MHz)

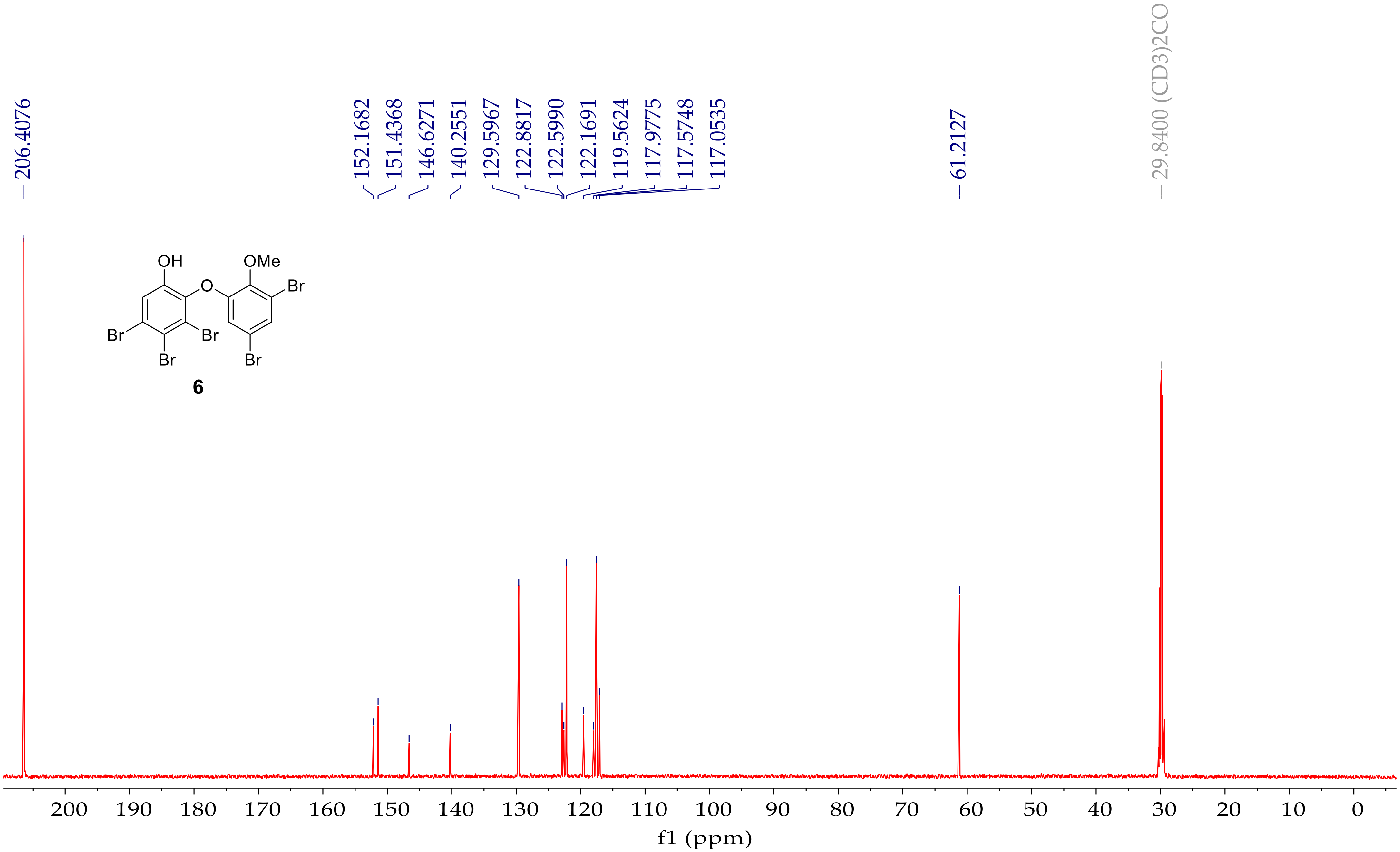

6

Figure S20. ^{1}H-^{13}C HSQC spectrum of **6** (Me_2CO-d_6, 500 MHz)

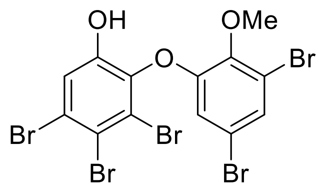

6

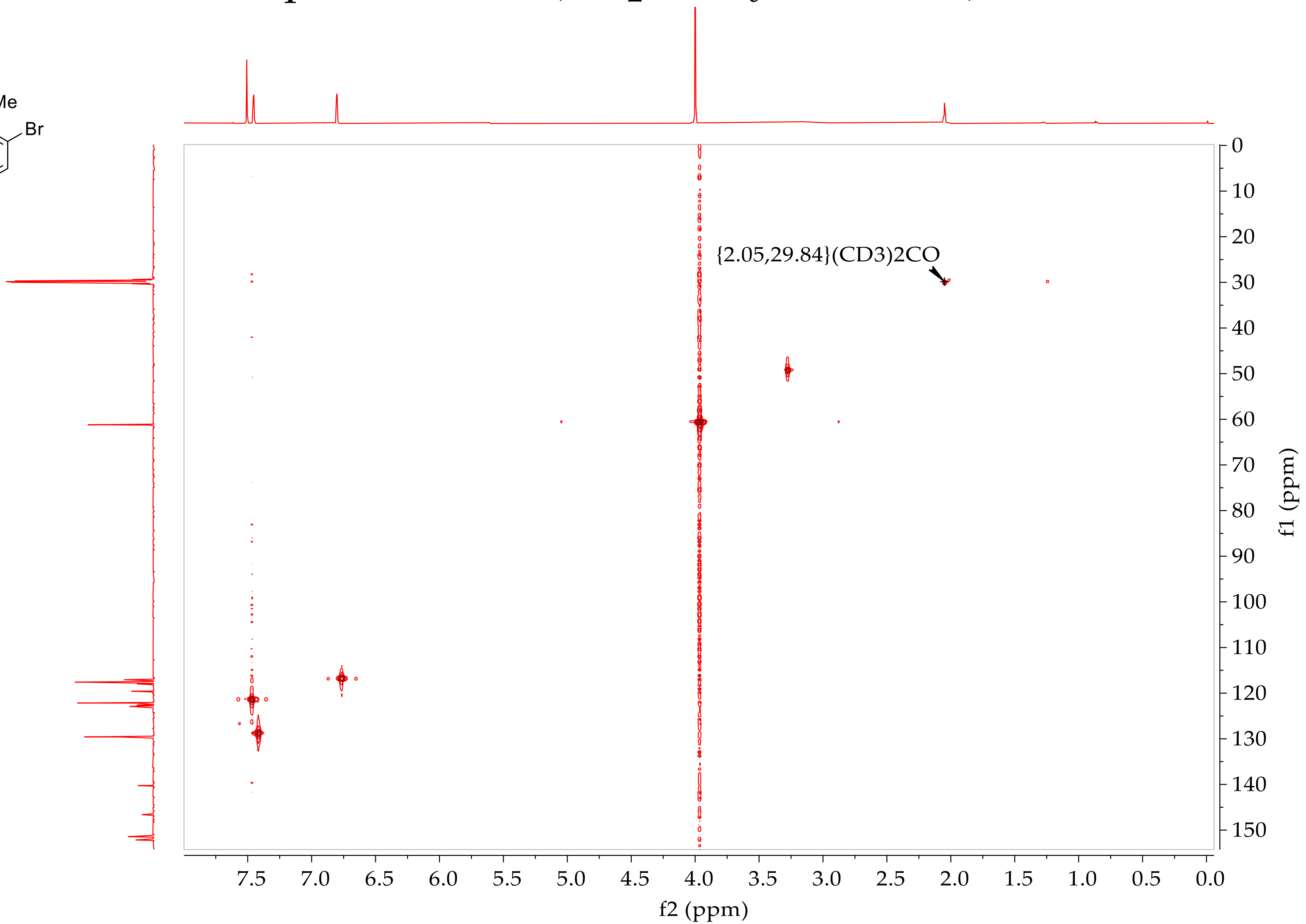

Figure S21. ^{1}H-^{13}C HMBC spectrum of **6** (Me_2CO-d_6, 500 MHz)

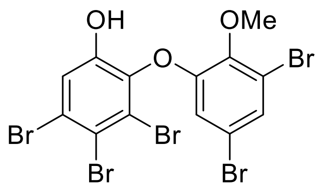

6

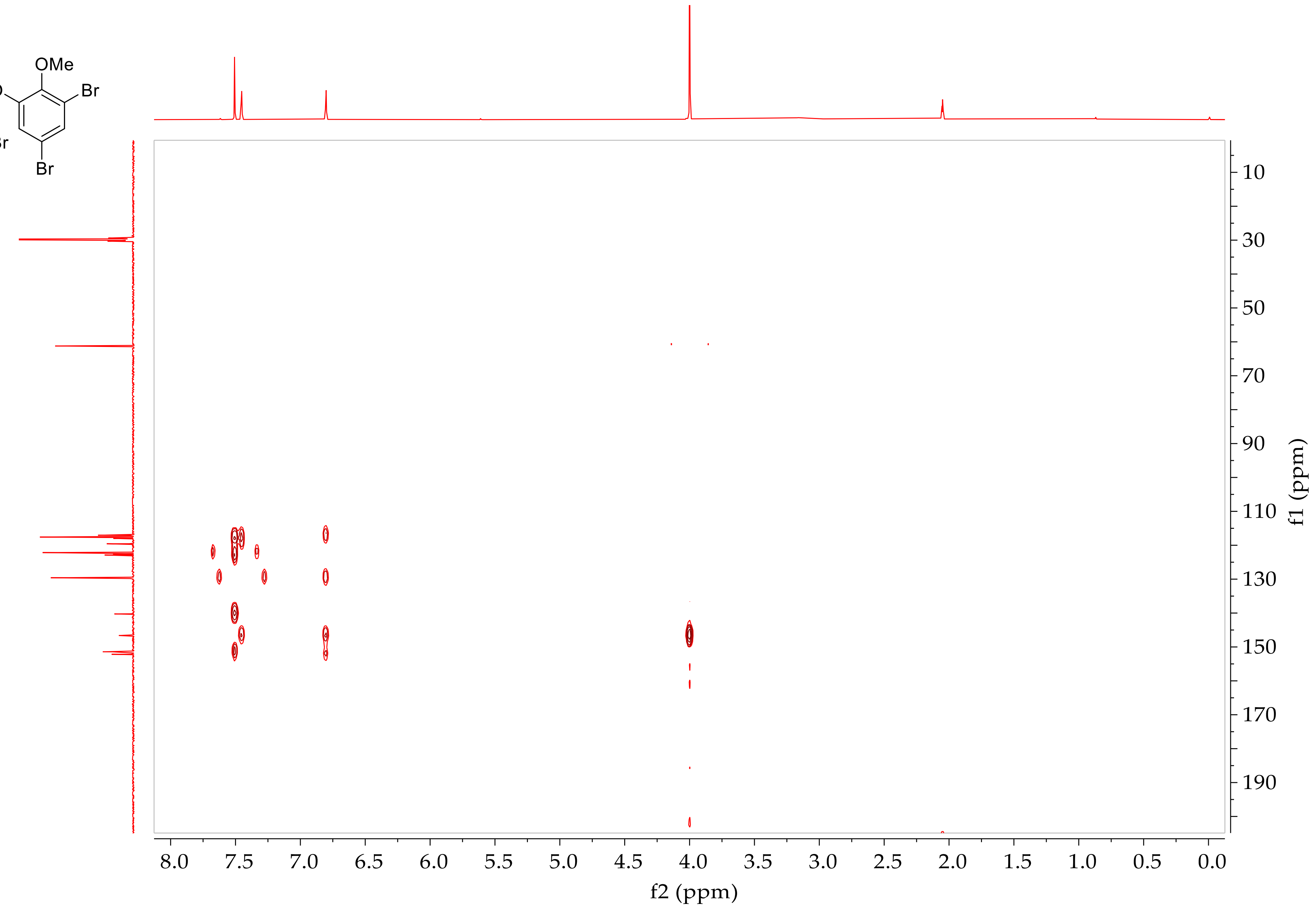

Figure S22. HREIMS of **6**

ターミナル1

SAMPLE : NH-C35-1356
SAMPLE NO.: 770 SCAN NO.: 27

PAGE 1

NO.	PKNO.	P.C.	M/Z(OBS.)	ER(MU)	US	ELEMENTS	
1	234	10.1	603.0111				
2	235	79.8	604.9632				
3	236	8.2	605.6311	-0.0	8.0	C13 H7 Br5 O3	
4	237	31.3	605.9715				
5	238	5.2	606.6461	7.1	7.5	C13 H8 Br5 O3	
6	239	54.1	607.6294	0.2	8.0	C13 H7 Br4 Br*1 O3	
7	240	16.8	608.6710				
8	241	79.7	609.6229	-4.1	8.0	C13 H7 Br3 Br*2 O3	
9	242	9.2	609.9897				
10	243	14.3	610.6332				
11	244	6.8	611.0001				
12	245	100.0	611.6311				← +6.0
13	246	7.9	611.8050				
14	247	8.3	612.0046				
15	248	15.6	612.6488				
16	249	51.1	613.6411				← +18.1
17	250	10.4	613.9119				
18	251	14.2	614.6598				
19	252	10.1	614.9887				

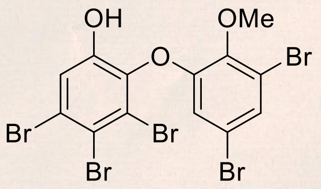

6

Table S14. Equilibrium Geometry DFT ωB97X-D/6-311+G(2d,p) of **6**

```
Job type: Geometry optimization.
Method: RWB97X-D
Basis set: 6-311+G(2D,P)
Number of basis functions: 714
Number of electrons: 284
Parallel Job: 16 threads

SCF model:
 A restricted hybrid HF-DFT SCF calculation will be
 performed using Pulay DIIS + Geometric Direct Minimization


Optimization:
         Step       Energy            Max Grad.       Max Dist.
            1   -13596.053628        0.025644        0.082958
            2   -13596.058798        0.006105        0.058462
            3   -13596.059382        0.001715        0.006906
            4   -13596.059418        0.001786        0.042893
            5   -13596.059498        0.001193        0.010775
            6   -13596.059513        0.000694        0.015156

  Reason for exit: Successful completion
  Quantum Calculation CPU Time :   6:33:10.28
  Quantum Calculation Wall Time:     26:25.66
```

Figure S23. HOMO-LUMO of **6**

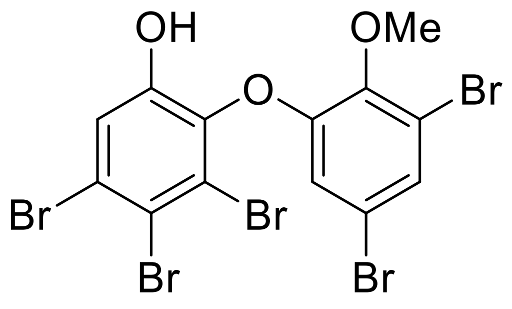

6

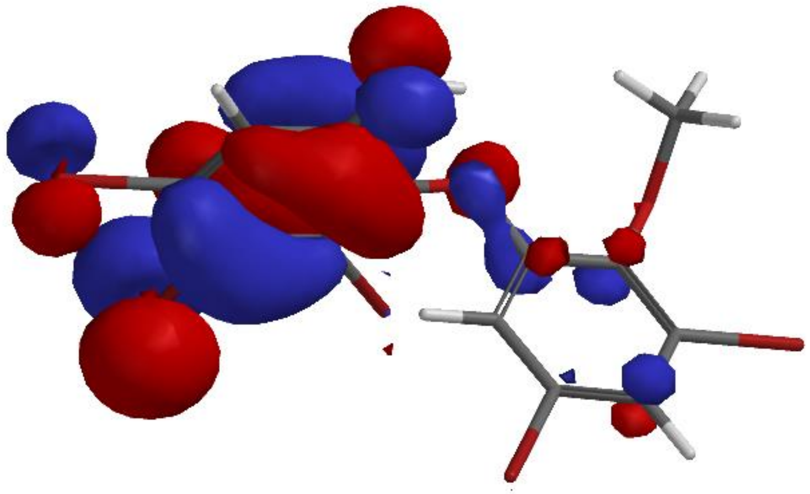

HOMO 6

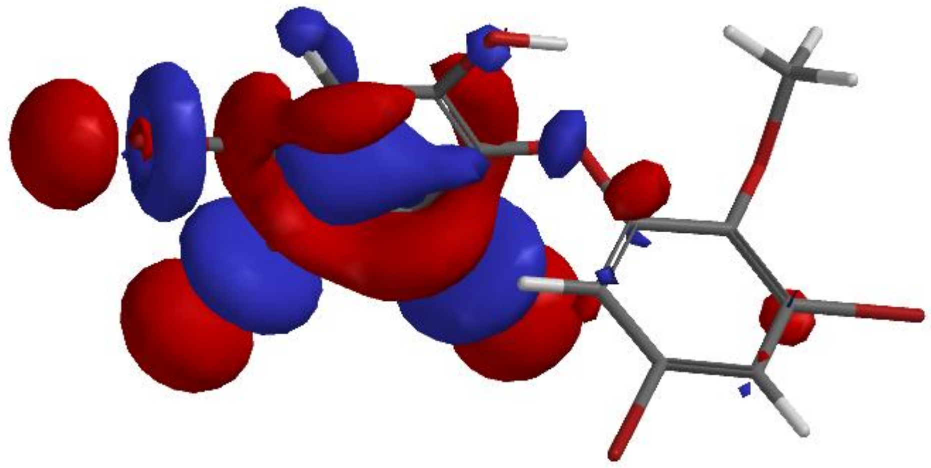

LUMO 6

Figure S24. Calculated ^{13}C NMR Chemical Shift of **6**

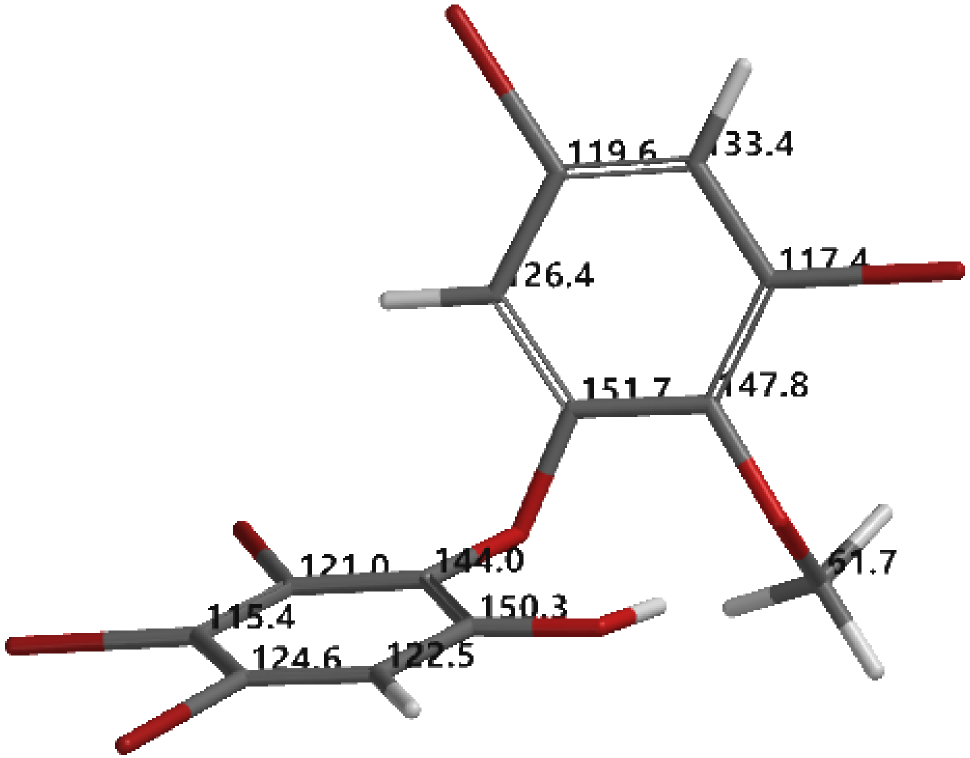

Figure S25. ^{1}H NMR spectrum of **7** (Me_2CO-d_6, 500 MHz)

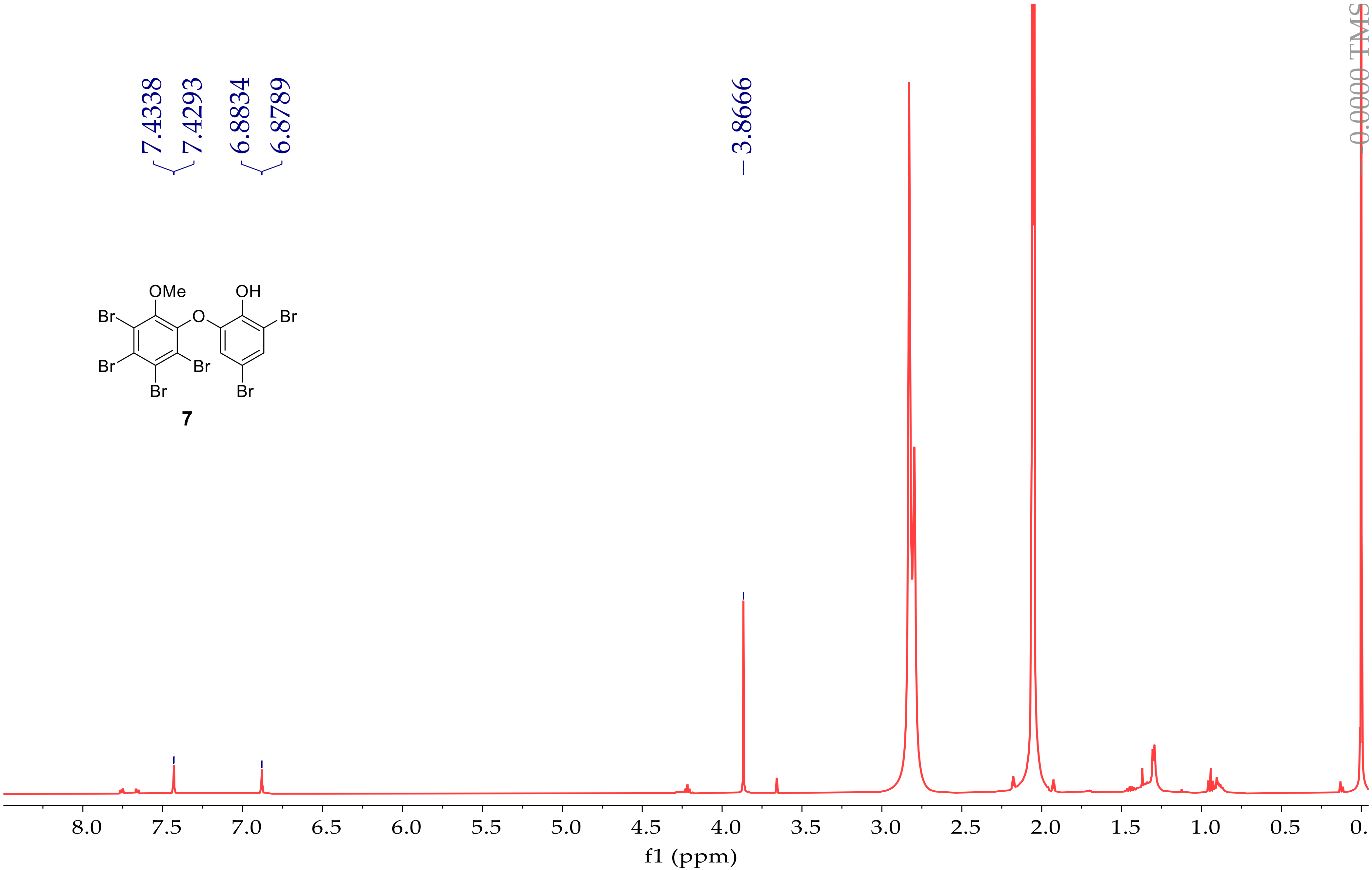

Figure S26. HRESIMS of **7**

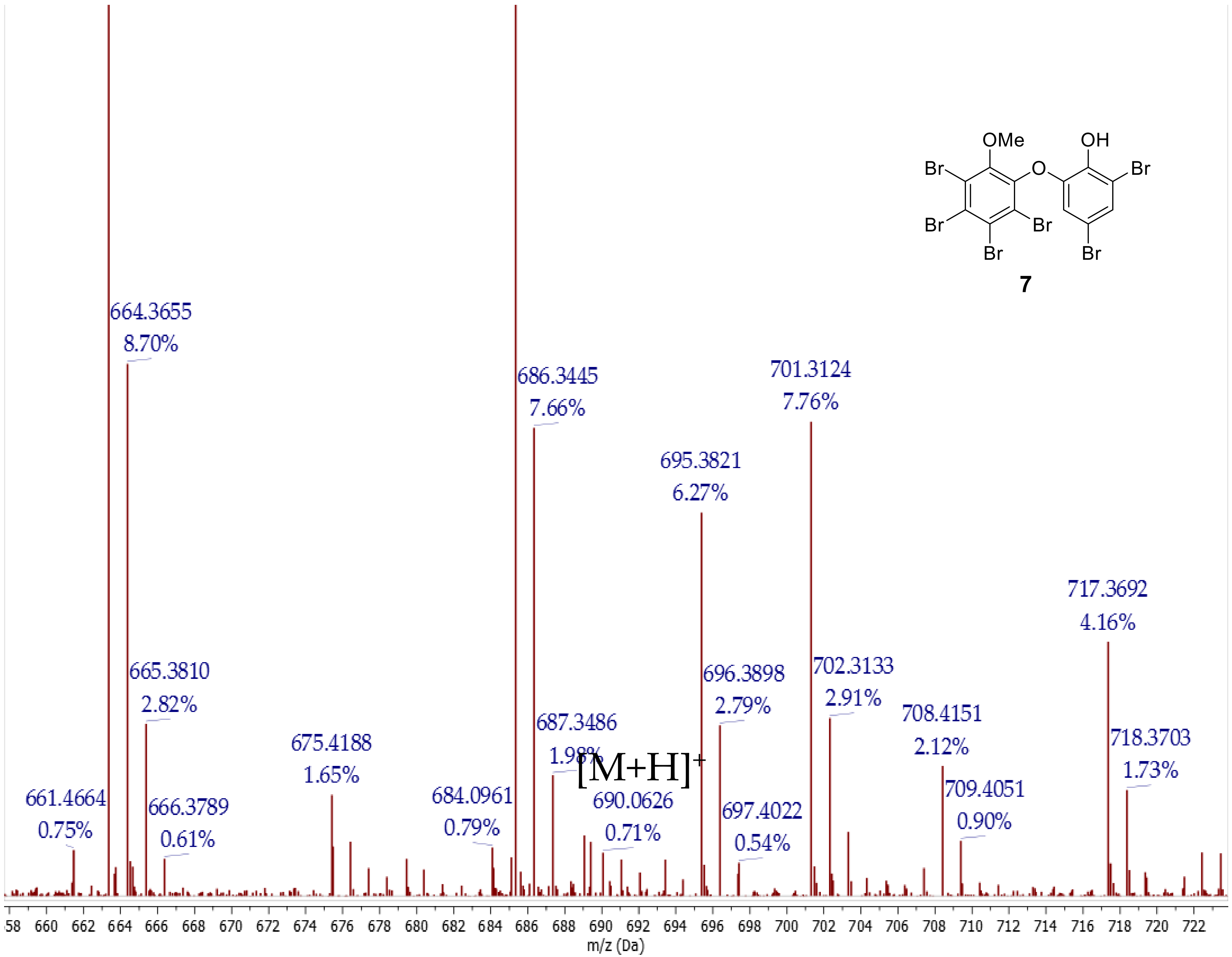

Table S15. Equilibrium Geometry DFT ωB97X-D/6-311+G(2d,p) of **7**

```
Job type: Geometry optimization.
Method: RWB97X-D
Basis set: 6-311+G(2D,P)
Number of basis functions: 756
Number of electrons: 318
Parallel Job: 16 threads

SCF model:
 A restricted hybrid HF-DFT SCF calculation will be
 performed using Pulay DIIS + Geometric Direct Minimization


Optimization:
        Step      Energy            Max Grad.       Max Dist.
           1   -16169.610850        0.028599        0.093509
           2   -16169.618486        0.008688        0.139524
           3   -16169.619389        0.001766        0.099035
           4   -16169.619639        0.001159        0.150255
           5   -16169.619796        0.001193        0.060879
           6   -16169.619824        0.001191        0.020720
           7   -16169.619839        0.000708        0.006034
           8   -16169.619843        0.000359        0.007205
```

Figure S27. HOMO-LUMO of **7**

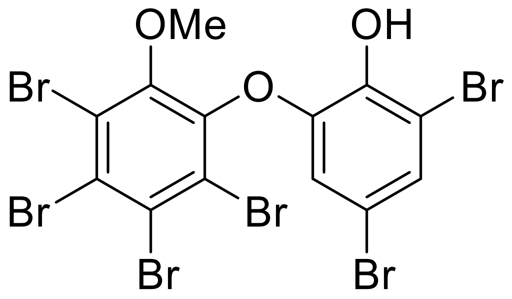

7

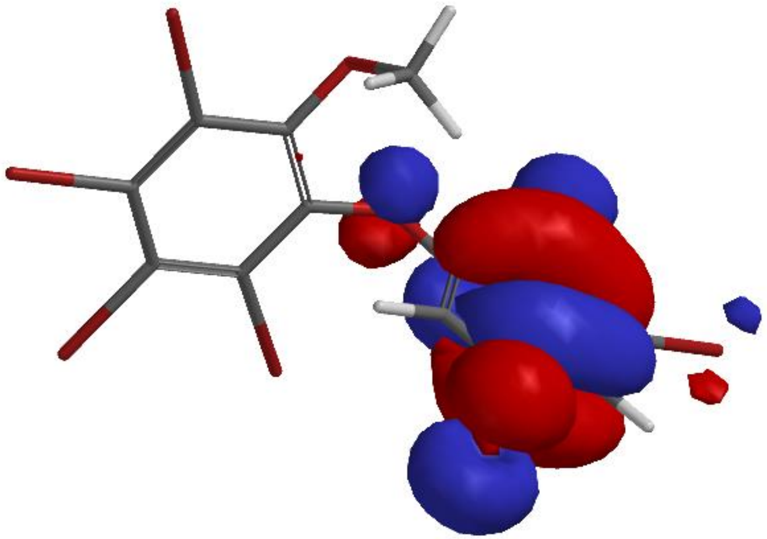

HOMO 7

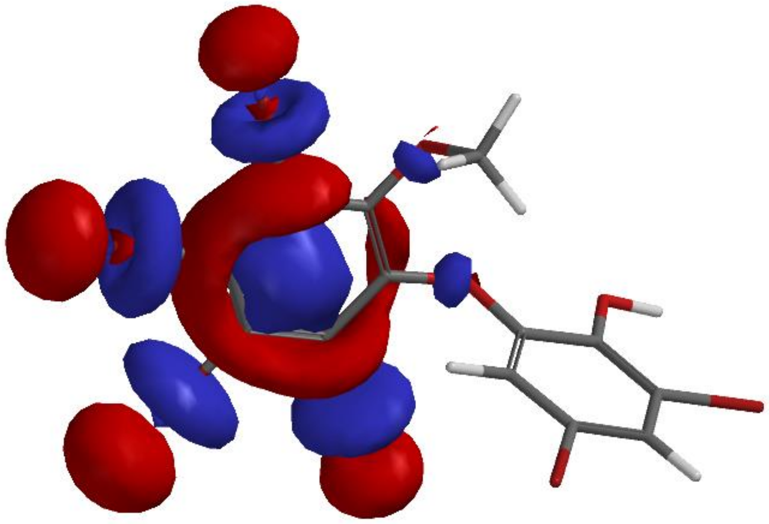

LUMO 7

Figure S28. Calculated ^{13}C NMR Chemical Shift of **7**

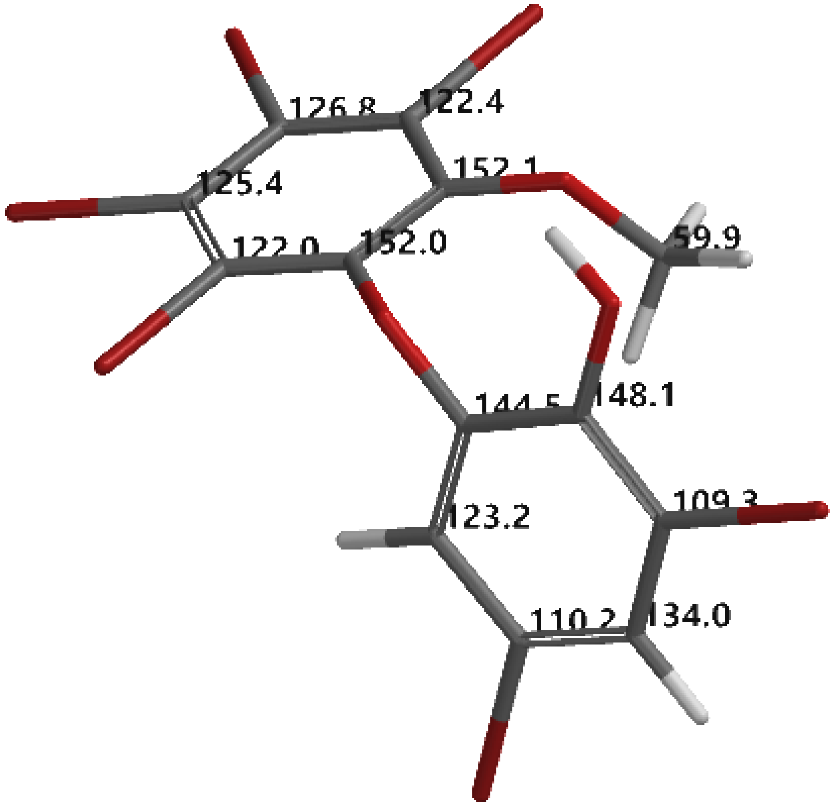

Figure S29. ^{1}H NMR spectrum of **8** ($CDCl_3$, 500 MHz)

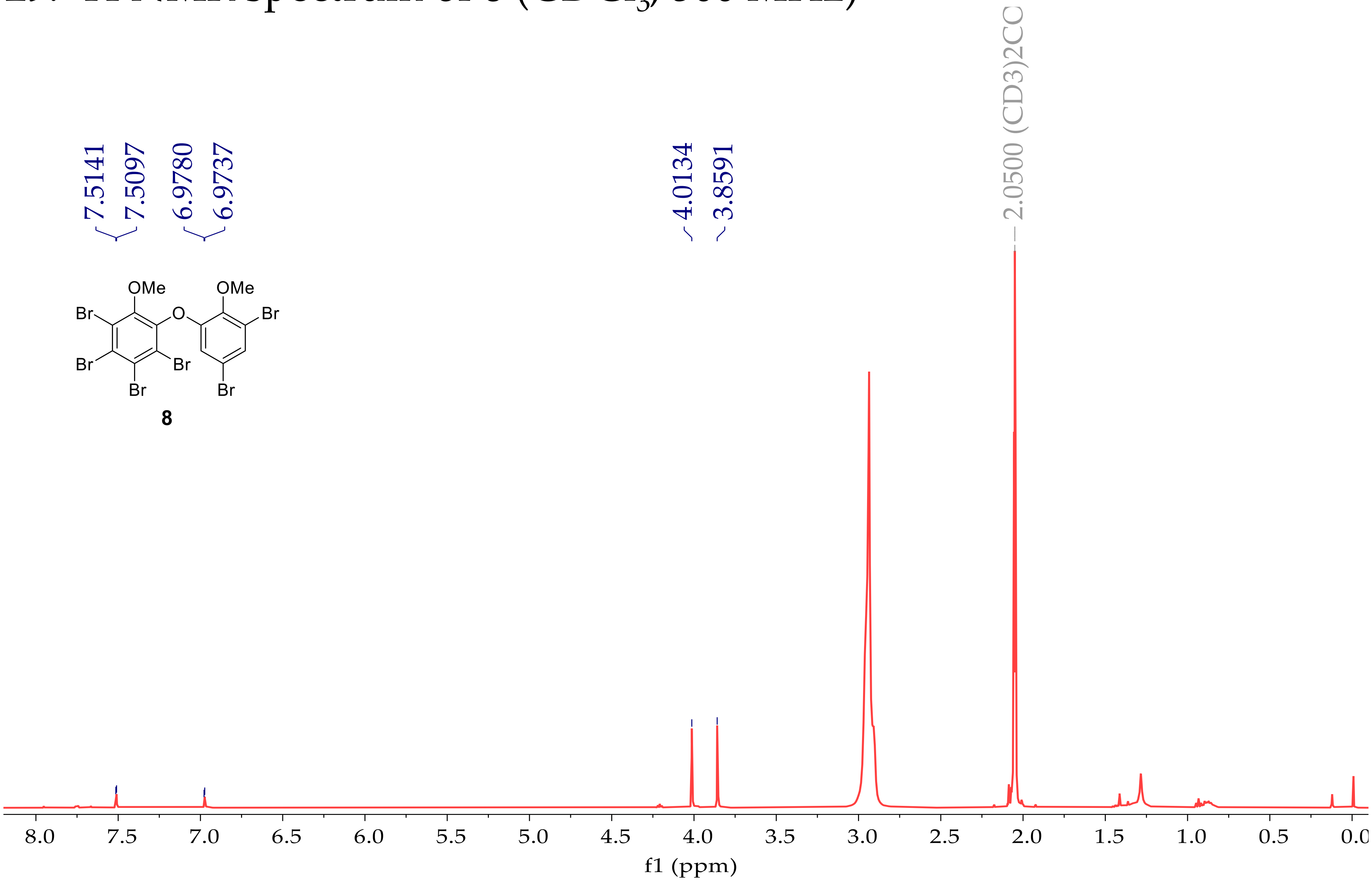

Figure S30. HRESIMS of **8**

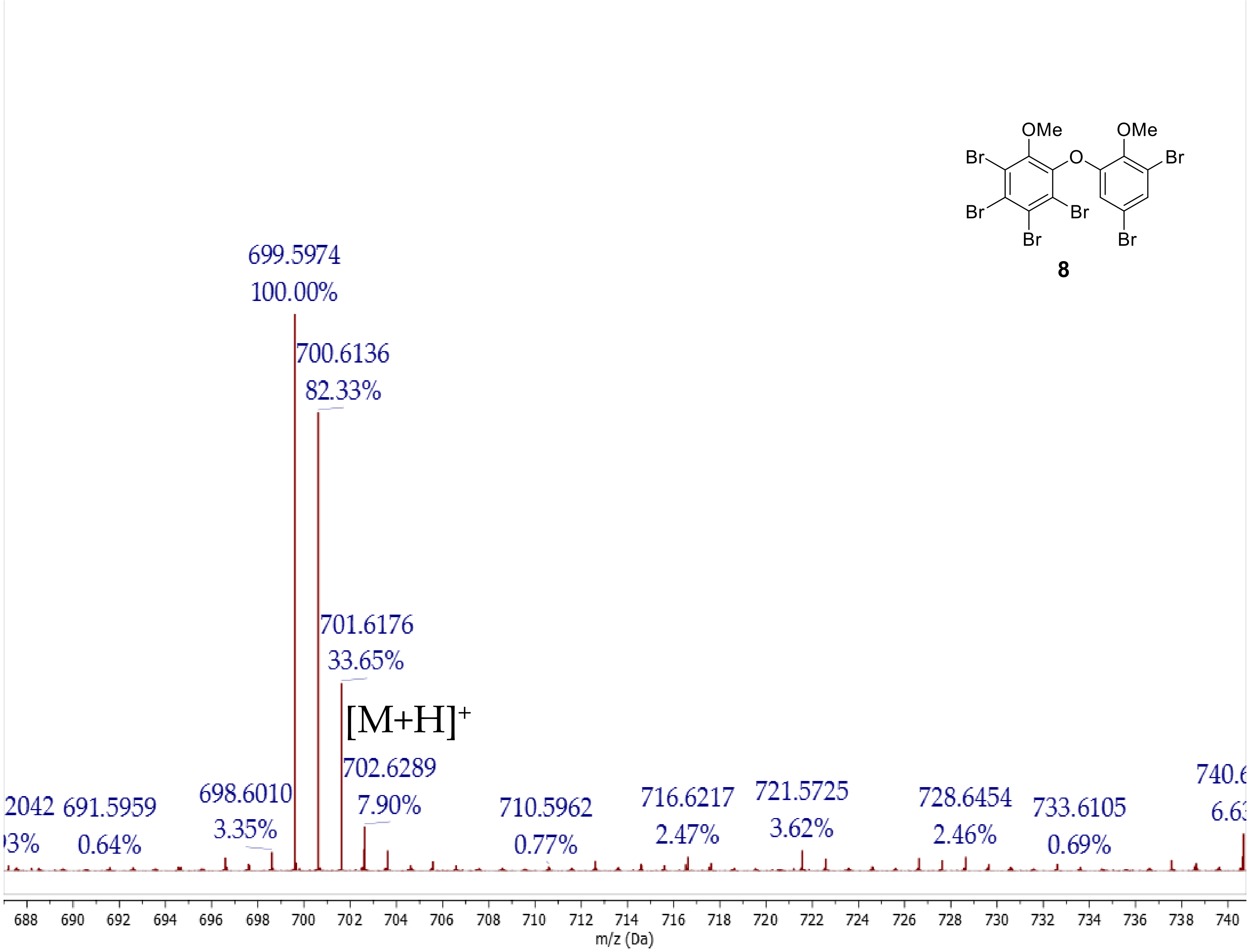

Figure S31. Crystal Structure of **8**

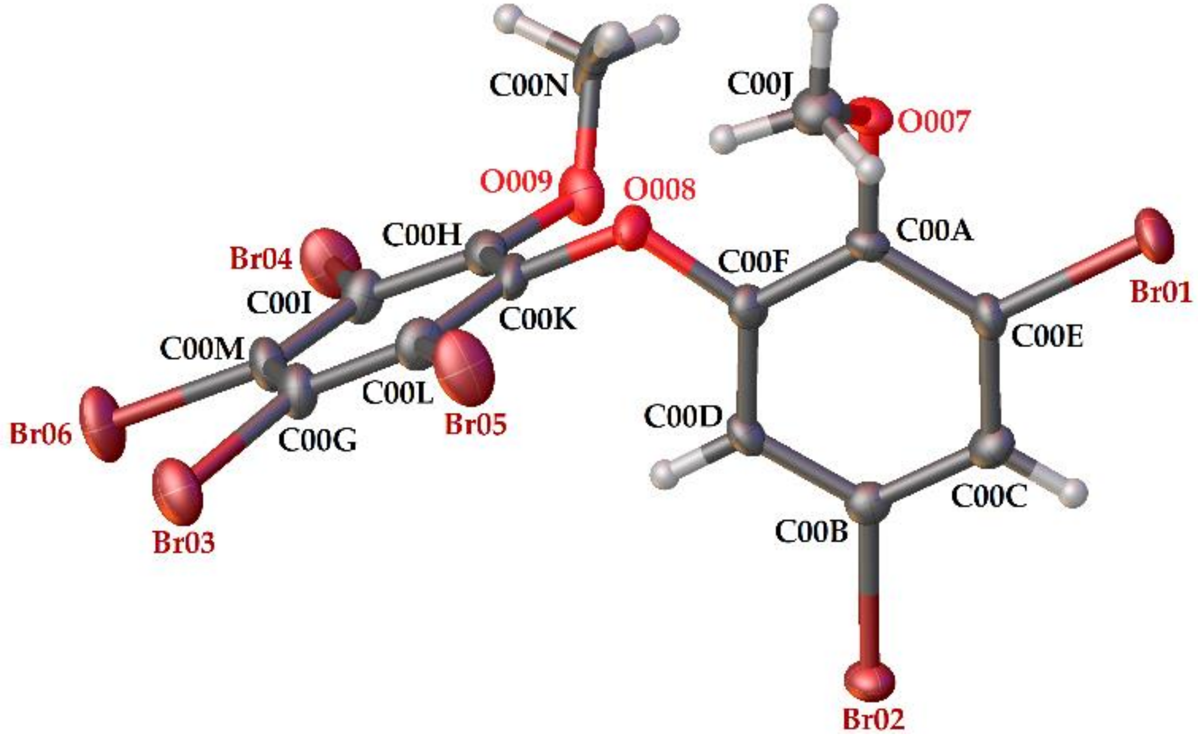

2,3,4,5-tetrabromo-6-(3',5'-dibromo-2'-methoxyphenoxy) anisole

Table S16. Crystal Data & Structure Refinement for **8**

Identification code	190412irai-TN-2-1
Empirical formula	$C_{14}H_8Br_6O_3$
Formula weight	703.66
Temperature/K	113
Crystal system	triclinic
Space group	P-1
a/Å	8.733(3)
b/Å	8.911(3)
c/Å	12.769(4)
α/°	104.185(4)
β/°	100.011(3)
γ/°	101.892(3)
Volume/Å^3	916.0(5)
Z	2
ϱ_{calc}g/cm^3	2.551
μ/mm^{-1}	13.158
F(000)	652.0
Crystal size/mm^3	0.317 × 0.229 × 0.21
Radiation	MoKα (λ = 0.71075)
2Θ range for data collection/°	6.588 to 54.968
Index ranges	-10 ≤ h ≤ 11, -11 ≤ k ≤ 11, -15 ≤ l ≤ 16
Reflections collected	7554
Independent reflections	4046 [R_{int} = 0.0369, R_{sigma} = 0.0427]
Data/restraints/parameters	4046/3/210
Goodness-of-fit on F^2	1.088
Final R indexes [I>=2σ (I)]	R_1 = 0.0422, wR_2 = 0.0964
Final R indexes [all data]	R_1 = 0.0472, wR_2 = 0.0997
Largest diff. peak/hole / e Å^{-3}	1.42/-1.20

Table S17. Fractional Atomic Coordinates (×10⁴) and Equivalent Isotropic Displacement Parameters (Å²×10³) for **8**. Ueq is defined as 1/3 of the trace of the orthogonalized UIJ tensor.

Atom	*x*	*y*	*z*	U(eq)
Br01	2276.2(7)	1367.2(7)	10563.0(4)	28.28(14)
Br02	7526.5(6)	3871.0(7)	8927.1(4)	25.11(13)
Br03	2872.1(8)	3874.3(7)	3447.4(4)	33.07(15)
Br04	3773.5(8)	9527.4(6)	7030.9(5)	36.51(16)
Br05	1413.9(9)	1719.4(7)	5016.7(5)	39.38(17)
Br06	3877.1(9)	7832.3(7)	4419.8(5)	36.83(16)
O007	394(4)	2350(4)	8712(3)	20.7(7)
O008	1522(4)	3791(4)	7275(3)	21.4(7)
O009	2645(5)	7055(5)	8153(3)	28.2(8)
C00A	2004(6)	2697(6)	8750(4)	16.8(9)
C00B	5293(6)	3424(5)	8861(4)	18.2(9)
C00C	4734(6)	2704(6)	9618(4)	20.8(10)
C00D	4274(6)	3806(6)	8068(4)	17.8(9)
C00E	3078(6)	2350(6)	9543(4)	18.4(9)
C00F	2663(6)	3457(6)	8034(4)	18.3(9)
C00G	2705(6)	4899(6)	4885(4)	24.4(11)
C00H	2594(6)	6365(6)	7068(4)	20.4(10)
C00I	3110(6)	7292(6)	6400(4)	23.2(10)
C00J	-555(6)	1008(6)	7776(4)	26.0(11)
C00K	2103(6)	4710(6)	6627(4)	19.8(10)
C00L	2141(6)	3969(6)	5543(4)	23.2(10)
C00M	3146(6)	6553(6)	5305(4)	22.9(10)
C00N	1244(9)	7187(8)	8383(4)	40.6(16)

Table S18. Anisotropic Displacement Parameters (Å^2×10^3) for **8**. The anisotropic displacement factor exponent takes the form: $-2\pi^2[h^2a^{*2}U_{11}+2hka^*b^*U_{12}+\ldots]$.

Atom	U_{11}	U_{22}	U_{33}	U_{23}	U_{13}	U_{12}
Br01	29.0(3)	35.4(3)	23.5(3)	16.9(2)	9.2(2)	2.7(2)
Br02	15.9(2)	32.0(3)	26.8(3)	8.9(2)	5.92(19)	3.8(2)
Br03	42.7(3)	28.1(3)	21.7(3)	4.5(2)	6.5(2)	-1.0(2)
Br04	52.9(4)	15.5(3)	37.7(3)	4.4(2)	15.4(3)	1.6(2)
Br05	56.5(4)	17.4(3)	36.1(3)	4.3(2)	12.5(3)	-5.0(3)
Br06	57.8(4)	23.9(3)	30.2(3)	15.1(2)	14.4(3)	1.7(3)
O007	14.4(16)	24.4(18)	22.8(18)	6.5(14)	6.6(13)	2.1(14)
O008	18.0(16)	24.4(18)	24.9(18)	15.2(15)	4.4(14)	3.2(14)
O009	33(2)	27(2)	17.9(17)	4.4(15)	2.2(15)	0.4(16)
C00A	14(2)	17(2)	19(2)	3.8(18)	6.5(17)	3.1(17)
C00B	18(2)	14(2)	21(2)	2.8(18)	3.1(18)	1.8(18)
C00C	18(2)	21(2)	20(2)	3.2(19)	0.0(19)	4.0(19)
C00D	19(2)	16(2)	17(2)	5.3(18)	7.3(18)	0.4(18)
C00E	22(2)	19(2)	14(2)	5.4(18)	4.6(18)	3.3(19)
C00F	21(2)	14(2)	20(2)	6.7(18)	5.2(19)	2.9(18)
C00G	29(3)	23(3)	19(2)	8(2)	3(2)	3(2)
C00H	18(2)	21(2)	19(2)	5.2(19)	1.1(18)	2.0(19)
C00I	27(3)	16(2)	22(2)	5.8(19)	0(2)	2(2)
C00J	20(2)	24(3)	29(3)	6(2)	2(2)	1(2)
C00K	17(2)	23(2)	20(2)	13(2)	1.5(18)	2.0(19)
C00L	26(3)	13(2)	27(3)	5.8(19)	3(2)	1.6(19)
C00M	27(3)	22(2)	18(2)	9(2)	5(2)	0(2)
C00N	76(4)	42(3)	13(2)	6(2)	2(3)	44(3)

Table S19. Bond Lengths for **8**

Atom	Atom	Length/Å
Br01	C00E	1.891(5)
Br02	C00B	1.891(5)
Br03	C00G	1.885(5)
Br04	C00I	1.878(5)
Br05	C00L	1.882(5)
Br06	C00M	1.889(5)
O007	C00A	1.366(6)
O007	C00J	1.447(6)
O008	C00F	1.396(6)
O008	C00K	1.385(6)
O009	C00H	1.359(6)
O009	C00N	1.329(8)
C00A	C00E	1.394(7)
C00A	C00F	1.397(7)
C00B	C00C	1.387(7)
C00B	C00D	1.384(7)
C00C	C00E	1.396(7)
C00D	C00F	1.368(7)
C00G	C00L	1.397(7)
C00G	C00M	1.383(7)
C00H	C00I	1.392(7)
C00H	C00K	1.387(7)
C00I	C00M	1.401(7)
C00K	C00L	1.390(7)

Table S20. Bond Angles for **8**

Atom	Atom	Atom	Angle/°
C00A	O007	C00J	113.6(4)
C00K	O008	C00F	116.9(4)
C00N	O009	C00H	115.8(4)
O007	C00A	C00E	120.7(4)
O007	C00A	C00F	122.4(4)
C00E	C00A	C00F	116.9(4)
C00C	C00B	Br02	118.0(4)
C00D	C00B	Br02	119.8(4)
C00D	C00B	C00C	122.2(5)
C00B	C00C	C00E	117.3(4)
C00F	C00D	C00B	118.5(5)
C00A	C00E	Br01	119.2(4)
C00A	C00E	C00C	122.5(5)
C00C	C00E	Br01	118.3(4)
O008	C00F	C00A	113.7(4)
C00D	C00F	O008	123.7(4)
C00D	C00F	C00A	122.6(4)
C00L	C00G	Br03	119.3(4)

Atom	Atom	Atom	Angle/°
C00M	C00G	Br03	121.3(4)
C00M	C00G	C00L	119.4(5)
O009	C00H	C00I	120.9(5)
O009	C00H	C00K	120.1(5)
C00K	C00H	C00I	118.9(5)
C00H	C00I	Br04	117.6(4)
C00H	C00I	C00M	120.1(5)
C00M	C00I	Br04	122.2(4)
O008	C00K	C00H	119.0(4)
O008	C00K	C00L	119.8(5)
C00H	C00K	C00L	121.2(5)
C00G	C00L	Br05	122.4(4)
C00K	C00L	Br05	117.8(4)
C00K	C00L	C00G	119.7(5)
C00G	C00M	Br06	120.1(4)
C00G	C00M	C00I	120.5(5)
C00I	C00M	Br06	119.4(4)

Table S21. Torsion Angles for **8**

A	B	C	D	Angle/˚
Br02	C00B	C00C	C00E	-178.6(3)
Br02	C00B	C00D	C00F	179.5(3)
Br03	C00G	C00L	Br05	-3.9(6)
Br03	C00G	C00L	C00K	176.2(4)
Br03	C00G	C00M	Br06	1.8(6)
Br03	C00G	C00M	C00I	-176.0(4)
Br04	C00I	C00M	Br06	-0.3(6)
Br04	C00I	C00M	C00G	177.5(4)
O007	C00A	C00E	Br01	0.7(6)
O007	C00A	C00E	C00C	-179.2(4)
O007	C00A	C00F	O008	-1.2(6)
O007	C00A	C00F	C00D	-180.0(4)
O008	C00K	C00L	Br05	-0.8(6)
O008	C00K	C00L	C00G	179.1(4)
O009	C00H	C00I	Br04	-3.1(7)
O009	C00H	C00I	C00M	175.9(5)
O009	C00H	C00K	O008	6.2(7)
O009	C00H	C00K	C00L	-175.7(5)
C00B	C00C	C00E	Br01	-180.0(3)
C00B	C00C	C00E	C00A	-0.1(7)
C00B	C00D	C00F	O008	179.8(4)
C00B	C00D	C00F	C00A	-1.6(7)
C00C	C00B	C00D	C00F	0.0(7)
C00D	C00B	C00C	C00E	0.9(7)
C00E	C00A	C00F	O008	-179.0(4)
C00E	C00A	C00F	C00D	2.2(7)
C00F	O008	C00K	C00H	-82.3(6)
C00F	O008	C00K	C00L	99.6(5)
C00F	C00A	C00E	Br01	178.5(3)
C00F	C00A	C00E	C00C	-1.3(7)
C00H	C00I	C00M	Br06	-179.2(4)
C00H	C00I	C00M	C00G	-1.4(8)
C00H	C00K	C00L	Br05	-179.0(4)
C00H	C00K	C00L	C00G	1.0(8)
C00I	C00H	C00K	O008	-177.1(4)
C00I	C00H	C00K	C00L	1.0(7)
C00J	O007	C00A	C00E	-107.6(5)
C00J	O007	C00A	C00F	74.6(6)
C00K	O008	C00F	C00A	172.6(4)
C00K	O008	C00F	C00D	-8.6(7)
C00K	C00H	C00I	Br04	-179.7(4)
C00K	C00H	C00I	C00M	-0.8(8)
C00L	C00G	C00M	Br06	-178.9(4)
C00L	C00G	C00M	C00I	3.3(8)
C00M	C00G	C00L	Br05	176.8(4)
C00M	C00G	C00L	C00K	-3.1(8)
C00N	O009	C00H	C00I	103.5(6)
C00N	O009	C00H	C00K	-79.9(6)

Table S22. Hydrogen Atom Coordinates (Å×10^4) and Isotropic Displacement Parameters (Å^2×10^3) for **8**

Atom	*x*	*y*	*z*	U(eq)
H00C	5433.13	2465.87	10153.75	25
H00D	4675.83	4290.02	7569.01	21
H00A	-447.93	1253.01	7096.84	39
H00B	-1665.7	805.46	7809.42	39
H00E	-180.51	73.77	7800.72	39
H00F	812.91	7854.42	7989.34	61
H00G	1400.96	7657.17	9167.9	61
H00H	504.42	6142.63	8158.12	61

Table S23. Refinement Model Description of **8**

Number of restraints - 3, number of constraints - unknown.

Details:

1. Fixed Uiso

At 1.2 times of: All C(H) groups

At 1.5 times of: All C(H,H,H) groups

2. Rigid body (RIGU) restrains O009, C00N with sigma for 1-2 distances of 0.004 and sigma for 1-3 distances of 0.004

3.a Aromatic/amide H refined with riding coordinates: C00C(H00C), C00D(H00D)

3.b Idealized Me refined as rotating group: C00J(H00A,H00B,H00E), C00N(H00F,H00G,H00H)

Table S24. Equilibrium Geometry DFT ωB97X-D/6-311+G(2d,p) of **8**

```
Job type: Geometry optimization.
Method: RWB97X-D
Basis set: 6-311+G(2D,P)
Number of basis functions: 795
Number of electrons: 326
Parallel Job: 16 threads

SCF model:
 A restricted hybrid HF-DFT SCF calculation will be
 performed using Pulay DIIS + Geometric Direct Minimization


Optimization:
         Step      Energy           Max Grad.      Max Dist.
            1   -16208.904060       0.031123       0.116981
            2   -16208.911301       0.008252       0.121129
            3   -16208.912230       0.003044       0.006869
            4   -16208.912278       0.003037       0.093174
            5   -16208.912575       0.002268       0.020735
            6   -16208.912629       0.001779       0.016953
            7   -16208.912658       0.001025       0.017400
            8   -16208.912668       0.000458       0.007091
```

Figure S32. HOMO-LUMO of **8**

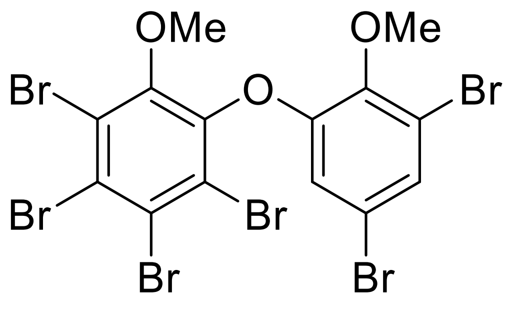

8

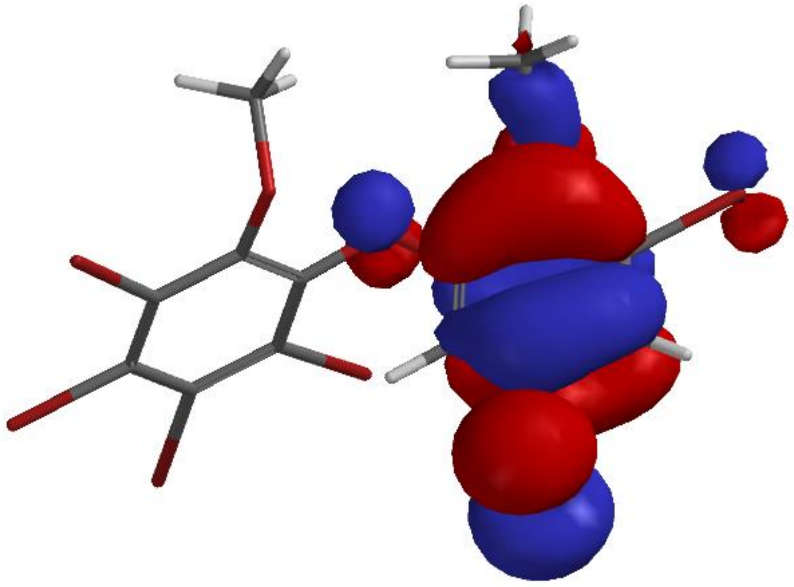

HOMO 8

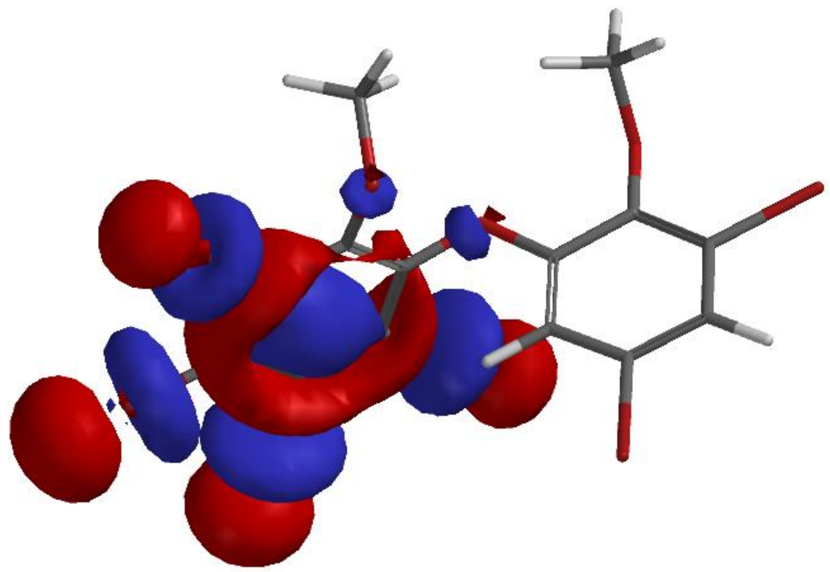

LUMO 8

Figure S33. Calculated ^{13}C NMR Chemical Shift of **8**

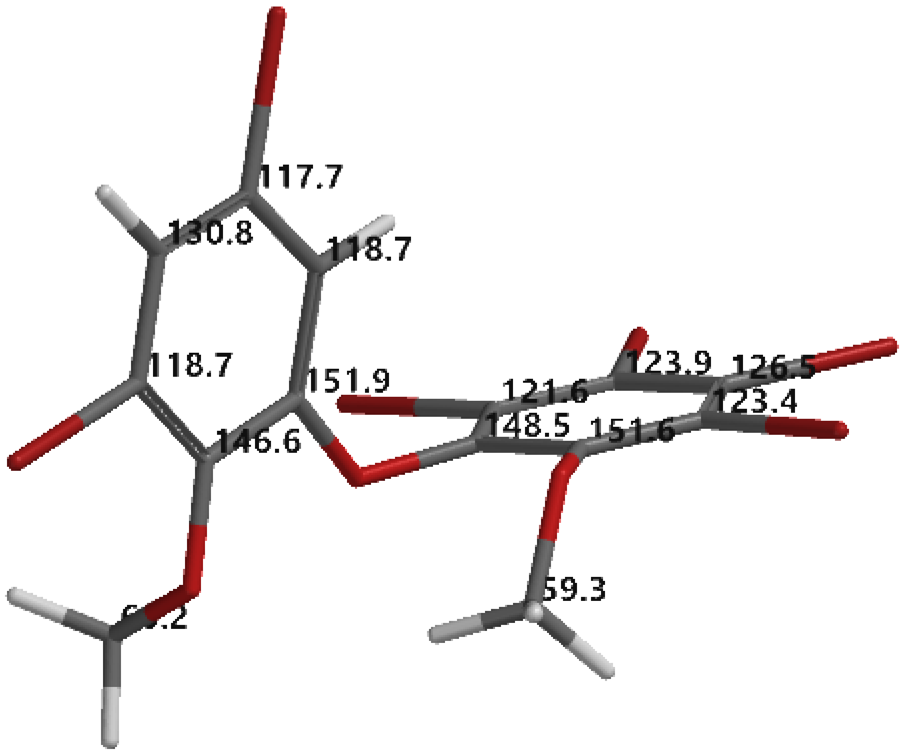

Figure S34. ^{1}H NMR spectrum of **9** ($CDCl_3$, 500 MHz)

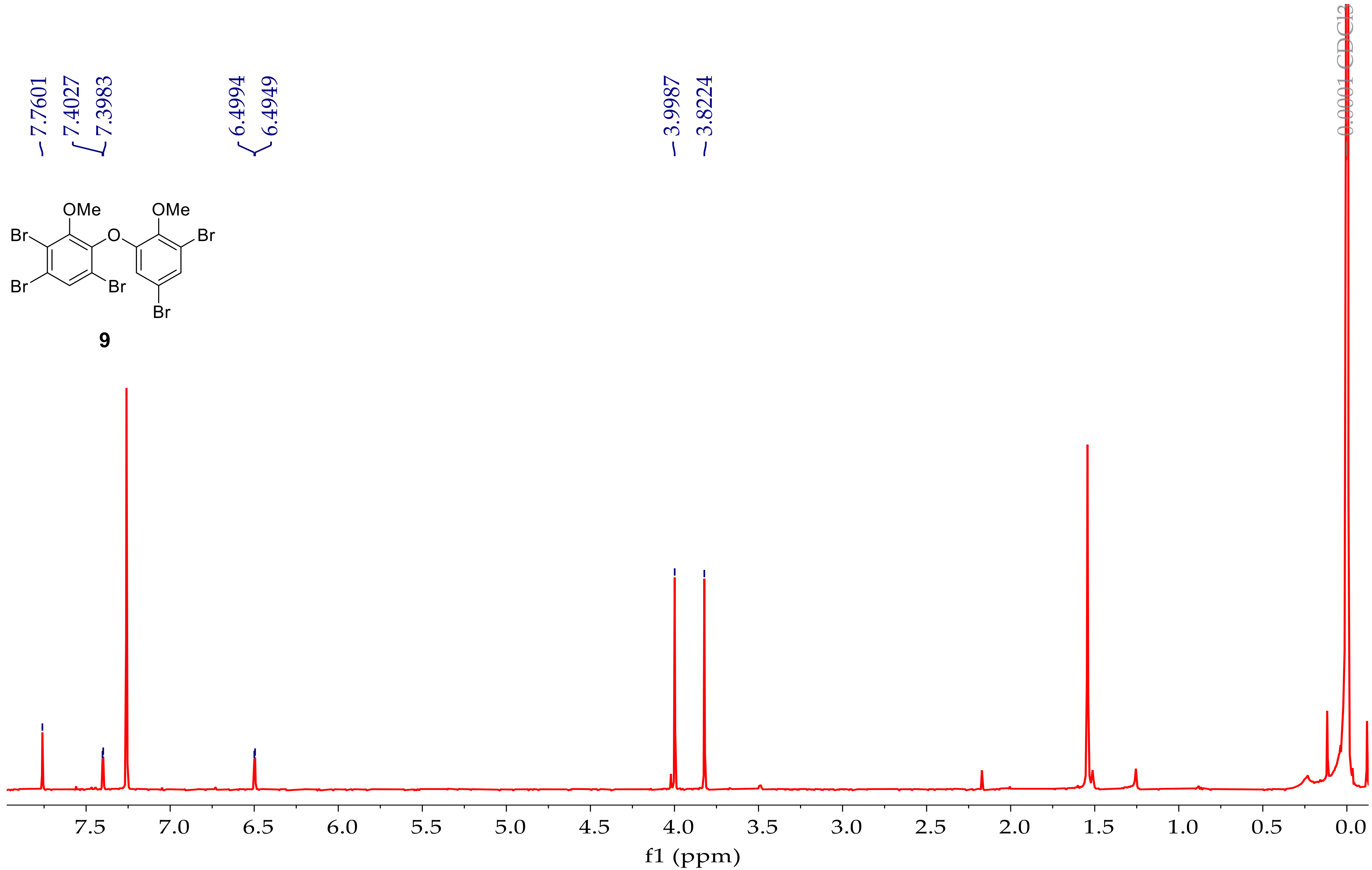

Figure S35. HRESIMS of **9**

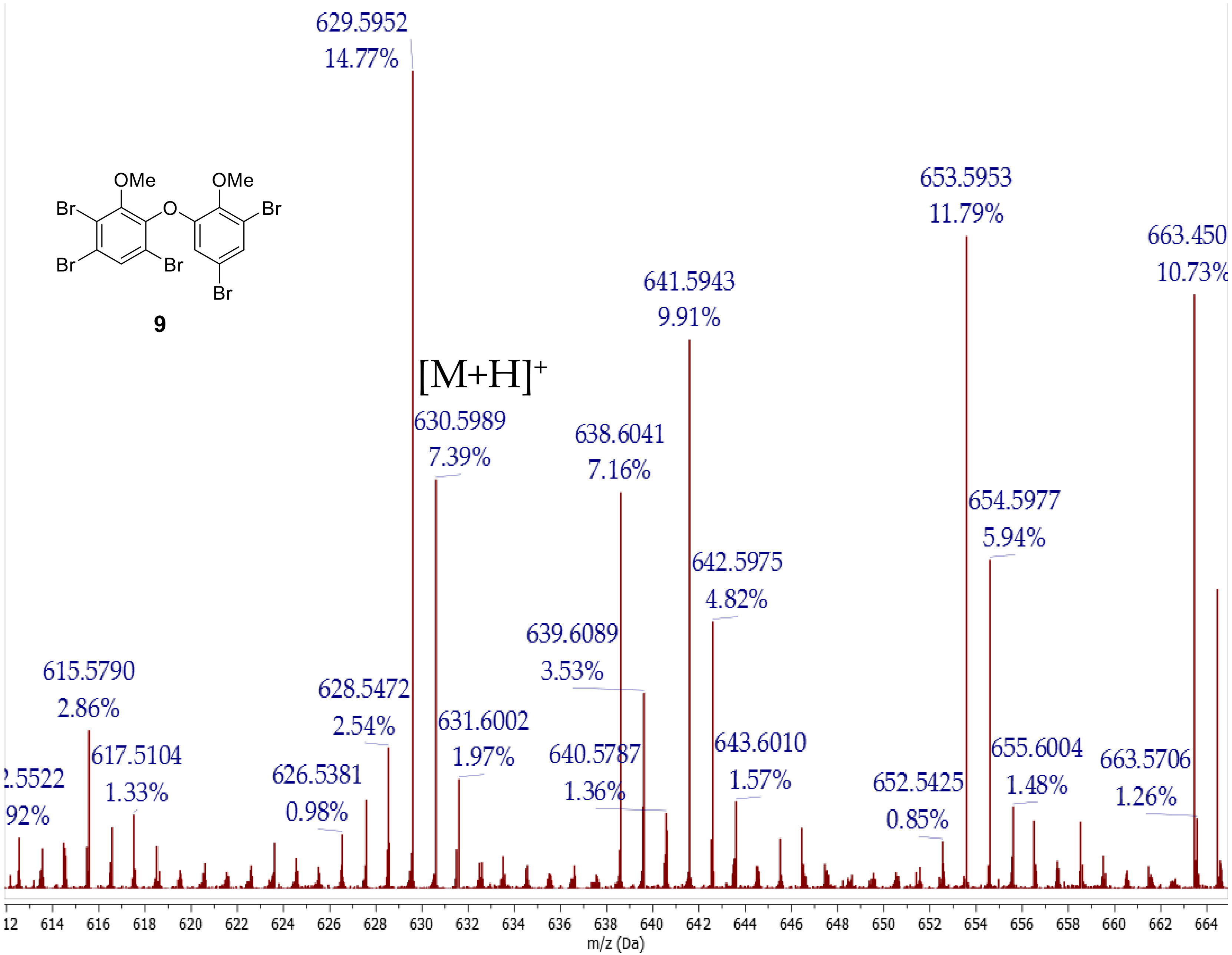

Figure S36. Crystal Structure of **9**

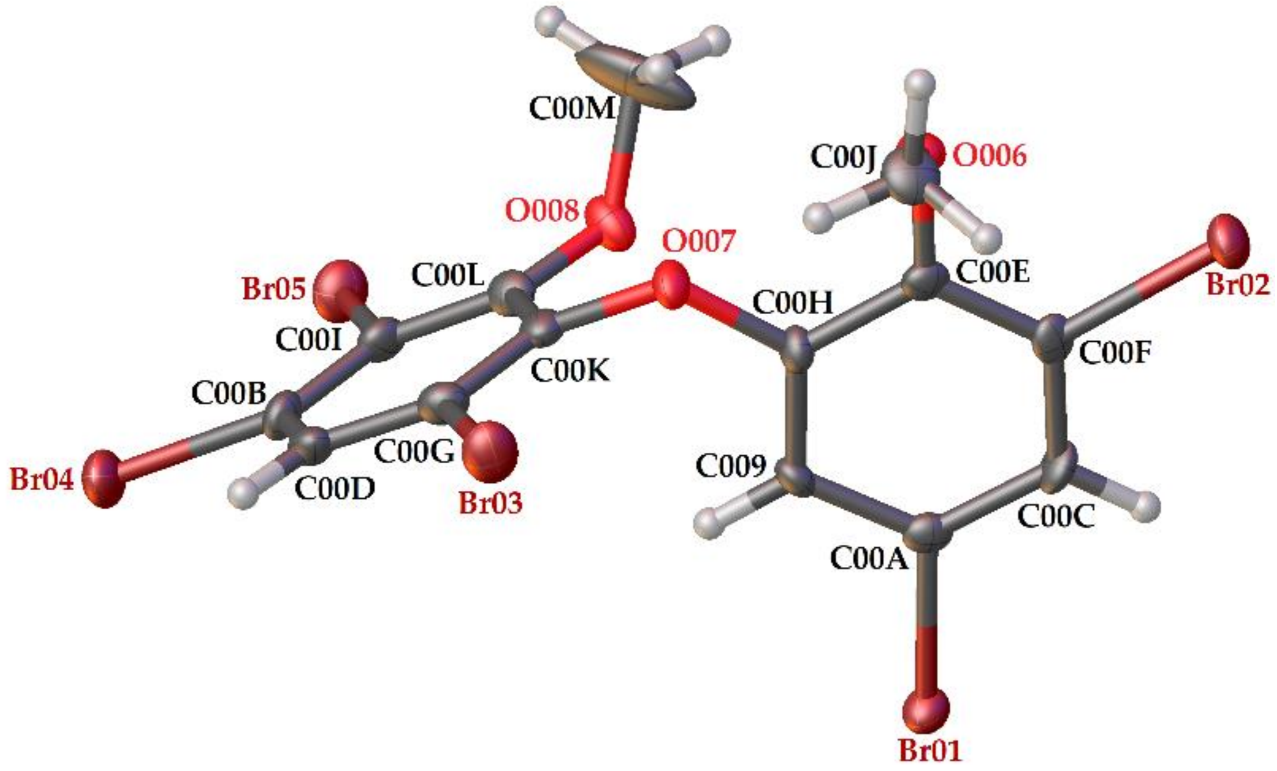

2,3,4,5-tetrabromo-6-(3',5'-dibromo-2'-methoxyphenoxy) anisole

Table S25. Crystal Data and Structure Refinement for **9**

Identification code	190412irai-TN-7
Empirical formula	$C_{14}H_9Br_5O_3$
Formula weight	624.76
Temperature/K	113.15
Crystal system	orthorhombic
Space group	$Pca2_1$
a/Å	6.9014(15)
b/Å	11.940(3)
c/Å	21.090(5)
α/°	90
β/°	90
γ/°	90
Volume/Å^3	1737.9(7)
Z	4
ρ_{calc}g/cm^3	2.388
μ/mm^{-1}	11.569
F(000)	1168.0
Crystal size/mm^3	0.26 × 0.11 × 0.06
Radiation	MoKα (λ = 0.71075)
2Θ range for data collection/°	6.82 to 54.952
Index ranges	-8 ≤ h ≤ 8, -15 ≤ k ≤ 15, -27 ≤ l ≤ 22
Reflections collected	17770
Independent reflections	3584 [R_{int} = 0.0623, R_{sigma} = 0.0509]
Data/restraints/parameters	3584/1/201
Goodness-of-fit on F^2	1.059
Final R indexes [I>=2σ (I)]	R_1 = 0.0359, wR_2 = 0.0648
Final R indexes [all data]	R_1 = 0.0480, wR_2 = 0.0681
Largest diff. peak/hole / e Å^{-3}	0.61/-0.60
Flack parameter	0.087(12)

Table S26. Fractional Atomic Coordinates ($\times10^4$) and Equivalent Isotropic Displacement Parameters ($Å^2\times10^3$) for **9**. Ueq is defined as 1/3 of the trace of the orthogonalized UIJ tensor.

Atom	*x*	*y*	*z*	U(eq)
Br01	7455.4(13)	530.8(7)	6203.2(4)	22.52(18)
Br02	3198.4(13)	3974.1(8)	7432.3(4)	25.8(2)
Br03	-533.9(14)	1031.0(8)	4576.7(4)	31.2(2)
Br04	4843.4(15)	1132.5(8)	2567.4(4)	31.9(2)
Br05	7279.5(13)	3188.6(9)	3310.0(5)	31.0(2)
O006	1251(8)	4088(4)	6129(3)	21.0(13)
O007	2018(8)	2887(5)	5088(3)	21.0(14)
O008	5380(8)	3753(5)	4563(3)	25.0(14)
C009	4541(12)	1838(7)	5627(4)	17.4(18)
C00A	5516(11)	1669(6)	6191(4)	18.3(17)
C00B	4003(12)	1709(7)	3355(4)	20.5(18)
C00C	5164(12)	2280(8)	6735(4)	20.8(19)
C00D	2306(14)	1270(7)	3609(4)	24(2)
C00E	2669(12)	3275(6)	6161(4)	18.6(17)
C00F	3731(12)	3093(7)	6702(4)	19.7(19)
C00G	1700(13)	1665(7)	4195(4)	21.8(19)
C00H	3120(12)	2656(7)	5621(4)	16.9(17)
C00I	5014(12)	2548(8)	3671(4)	23.8(19)
C00J	-666(11)	3639(8)	6221(5)	28(2)
C00K	2702(13)	2498(7)	4511(4)	21.1(18)
C00L	4356(12)	2961(7)	4245(4)	20.0(19)
C00M	4487(17)	4814(10)	4615(8)	74(5)

Table S27. Anisotropic Displacement Parameters ($Å^2×10^3$) for **9**. The anisotropic displacement factor exponent takes the form: $-2\pi^2[h^2a^{*2}U_{11}+2hka^*b^*U_{12}+…]$.

Atom	U_{11}	U_{22}	U_{33}	U_{23}	U_{13}	U_{12}
Br01	22.8(4)	24.8(4)	19.9(4)	0.8(4)	-0.4(4)	6.5(4)
Br02	31.2(5)	28.5(5)	17.6(4)	-5.9(4)	1.9(4)	2.8(4)
Br03	34.9(5)	30.9(6)	27.7(5)	1.3(5)	4.9(4)	-9.7(4)
Br04	48.0(6)	31.8(5)	15.9(4)	-1.9(4)	4.2(4)	3.6(4)
Br05	30.7(5)	36.2(6)	26.0(5)	2.7(4)	5.5(4)	-3.4(4)
O006	23(3)	17(3)	23(3)	0(3)	1(3)	3(2)
O007	23(3)	26(3)	14(3)	-4(3)	0(2)	7(2)
O008	21(3)	27(4)	28(3)	-10(3)	-2(3)	0(3)
C009	22(4)	13(4)	17(4)	-5(3)	2(3)	0(4)
C00A	18(4)	15(4)	22(5)	5(4)	4(4)	-2(3)
C00B	25(4)	25(5)	12(4)	4(4)	2(4)	1(4)
C00C	18(4)	29(5)	15(4)	3(4)	-7(4)	1(4)
C00D	39(5)	14(4)	20(5)	2(4)	-5(4)	0(4)
C00E	22(4)	15(4)	19(4)	5(4)	4(4)	-1(4)
C00F	24(4)	21(5)	14(4)	3(3)	6(4)	-6(4)
C00G	24(5)	17(4)	25(5)	3(4)	-1(4)	-2(4)
C00H	17(4)	21(5)	13(4)	-3(3)	-2(3)	-1(4)
C00I	32(5)	20(5)	19(4)	6(4)	4(4)	1(4)
C00J	18(4)	34(5)	31(5)	-2(4)	7(4)	4(4)
C00K	27(5)	18(4)	19(4)	1(4)	-3(4)	8(4)
C00L	15(4)	21(5)	23(5)	0(4)	-5(3)	0(4)
C00M	37(6)	28(6)	156(14)	-39(8)	-13(8)	-3(5)

Table S28. Bond Lengths for **9**

Atom	Atom	Length/Å
Br01	C00A	1.907(8)
Br02	C00F	1.901(8)
Br03	C00G	1.898(9)
Br04	C00B	1.890(9)
Br05	C00I	1.899(9)
O006	C00E	1.380(9)
O006	C00J	1.441(10)
O007	C00H	1.386(10)
O007	C00K	1.385(10)
O008	C00L	1.358(10)
O008	C00M	1.413(12)
C009	C00A	1.381(12)
C009	C00H	1.385(11)
C00A	C00C	1.381(12)
C00B	C00D	1.390(12)
C00B	C00I	1.391(13)
C00C	C00F	1.388(12)
C00D	C00G	1.387(12)
C00E	C00F	1.373(12)
C00E	C00H	1.394(11)
C00G	C00K	1.383(12)
C00I	C00L	1.384(12)
C00K	C00L	1.386(12)

Table S29. Bond Angles for **9**

Atom	Atom	Atom	Angle/°
C00E	O006	C00J	112.5(6)
C00K	O007	C00H	117.3(6)
C00L	O008	C00M	115.8(7)
C00A	C009	C00H	117.1(8)
C009	C00A	Br01	117.2(6)
C00C	C00A	Br01	119.2(6)
C00C	C00A	C009	123.6(7)
C00D	C00B	Br04	117.3(7)
C00D	C00B	C00I	120.7(8)
C00I	C00B	Br04	121.9(6)
C00A	C00C	C00F	117.0(7)
C00G	C00D	C00B	117.9(8)
O006	C00E	C00H	119.4(8)
C00F	C00E	O006	122.1(8)
C00F	C00E	C00H	118.5(8)
C00C	C00F	Br02	119.0(6)
C00E	C00F	Br02	118.8(7)

Atom	Atom	Atom	Angle/°
C00E	C00F	C00C	122.2(8)
C00D	C00G	Br03	119.2(7)
C00K	C00G	Br03	119.2(7)
C00K	C00G	C00D	121.6(8)
O007	C00H	C00E	115.8(7)
C009	C00H	O007	122.5(7)
C009	C00H	C00E	121.6(8)
C00B	C00I	Br05	120.8(7)
C00L	C00I	Br05	118.5(7)
C00L	C00I	C00B	120.7(8)
O007	C00K	C00L	120.2(8)
C00G	C00K	O007	119.6(8)
C00G	C00K	C00L	120.2(8)
O008	C00L	C00I	120.6(8)
O008	C00L	C00K	120.4(8)
C00I	C00L	C00K	118.8(8)

Table S30. Torsion Angles for **9**

A	B	C	D	Angle/°
Br01	C00A	C00C	C00F	-179.6(6)
Br03	C00G	C00K	O007	-2.6(11)
Br03	C00G	C00K	C00L	179.0(6)
Br04	C00B	C00D	C00G	178.4(7)
Br04	C00B	C00I	Br05	1.6(11)
Br04	C00B	C00I	C00L	179.5(7)
Br05	C00I	C00L	O008	-4.4(11)
Br05	C00I	C00L	C00K	179.9(6)
O006	C00E	C00F	Br02	1.1(11)
O006	C00E	C00F	C00C	-179.6(8)
O006	C00E	C00H	O007	-2.5(11)
O006	C00E	C00H	C009	179.4(7)
O007	C00K	C00L	O008	3.8(12)
O007	C00K	C00L	C00I	179.5(8)
C009	C00A	C00C	C00F	0.8(13)
C00A	C009	C00H	O007	-178.7(7)
C00A	C009	C00H	C00E	-0.8(12)
C00A	C00C	C00F	Br02	-179.3(6)
C00A	C00C	C00F	C00E	1.4(13)
C00B	C00D	C00G	Br03	-176.7(6)
C00B	C00D	C00G	C00K	2.3(13)
C00B	C00I	C00L	O008	177.7(8)
C00B	C00I	C00L	C00K	2.0(13)
C00D	C00B	C00I	Br05	-177.6(6)
C00D	C00B	C00I	C00L	0.3(13)
C00D	C00G	C00K	O007	178.4(8)
C00D	C00G	C00K	C00L	0.0(13)
C00F	C00E	C00H	O007	-179.1(7)
C00F	C00E	C00H	C009	2.9(12)
C00G	C00K	C00L	O008	-177.8(8)
C00G	C00K	C00L	C00I	-2.1(13)
C00H	O007	C00K	C00G	112.5(9)
C00H	O007	C00K	C00L	-69.1(10)
C00H	C009	C00A	Br01	179.3(6)
C00H	C009	C00A	C00C	-1.1(13)
C00H	C00E	C00F	Br02	177.5(6)
C00H	C00E	C00F	C00C	-3.2(13)
C00I	C00B	C00D	C00G	-2.4(12)
C00J	O006	C00E	C00F	-98.5(9)
C00J	O006	C00E	C00H	85.1(9)
C00K	O007	C00H	C009	-17.0(11)
C00K	O007	C00H	C00E	164.9(7)
C00M	O008	C00L	C00I	113.5(11)
C00M	O008	C00L	C00K	-70.9(12)

Table S31. Hydrogen Atom Coordinates (Å×10^4) and Isotropic Displacement Parameters (Å^2×10^3) for **9**

Atom	*x*	*y*	*z*	U(eq)
H009	4827.61	1419.61	5266.82	21
H00C	5856.47	2151.82	7106.42	25
H00D	1599.05	728.47	3392.07	29
H00A	-993.9	3162.57	5870.23	42
H00B	-1581.68	4242.78	6246.43	42
H00E	-699.22	3213.84	6606.7	42
H00F	4394.88	5149.86	4203.06	111
H00G	5246.52	5284.57	4887.94	111
H00H	3211.6	4726.98	4790.67	111

Table S32. Refinement model description for **9**

Number of restraints - 1, number of constraints - unknown.

Details:

1. Fixed Uiso

At 1.2 times of: All C(H) groups

At 1.5 times of: All C(H,H,H) groups

2.a Aromatic/amide H refined with riding coordinates: C009(H009), C00C(H00C), C00D(H00D)

2.b Idealized Me refined as rotating group: C00J(H00A,H00B,H00E), C00M(H00F,H00G,H00H)

Table S33. Equilibrium Geometry DFT ωB97X-D/6-311+G(2d,p) of **9**

```
Job type: Geometry optimization.
Method: RWB97X-D
Basis set: 6-311+G(2D,P)
Number of basis functions: 753
Number of electrons: 292
Parallel Job: 16 threads

SCF model:
 A restricted hybrid HF-DFT SCF calculation will be
 performed using Pulay DIIS + Geometric Direct Minimization


Optimization:
         Step      Energy          Max Grad.      Max Dist.
            1   -13635.346267      0.025715       0.101967
            2   -13635.352033      0.007462       0.126962
            3   -13635.352956      0.002650       0.005913
            4   -13635.352998      0.002584       0.083121
            5   -13635.353359      0.003436       0.044216
            6   -13635.353470      0.002677       0.038853
            7   -13635.353545      0.001050       0.030200
            8   -13635.353572      0.000731       0.011531
            9   -13635.353579      0.000452       0.007713
```

Figure S37. HOMO-LUMO of **9**

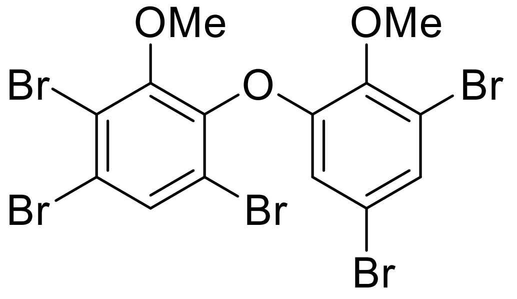

9

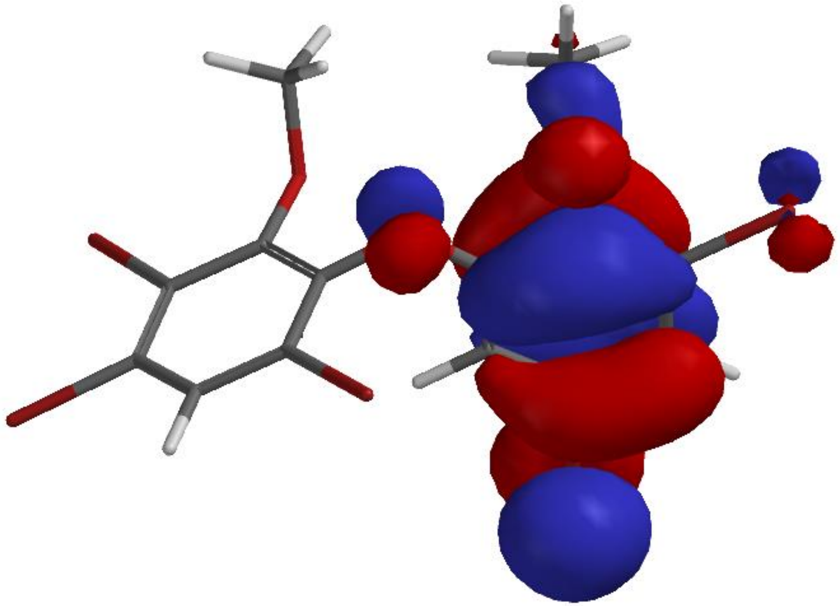

HOMO 9

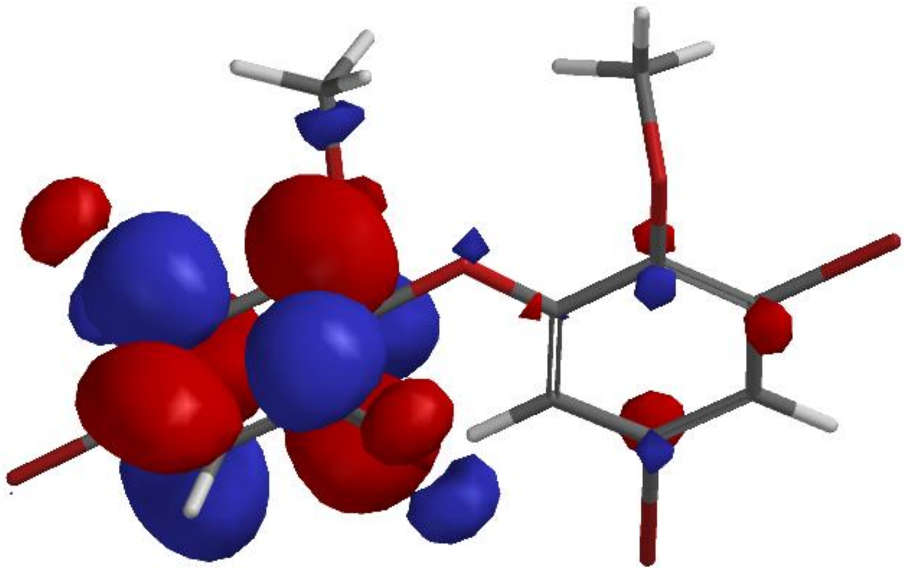

LUMO 9

Figure S38. Calculated ^{13}C NMR Chemical Shift of **9**

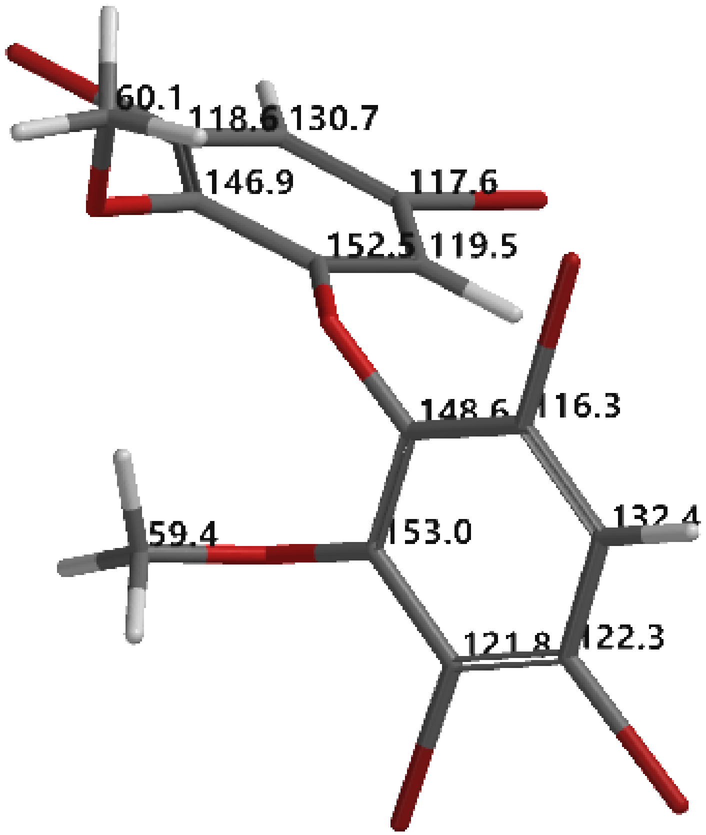

Figure S39. ^{1}H NMR spectrum of **10** (Me_2CO-d_6, 500 MHz)

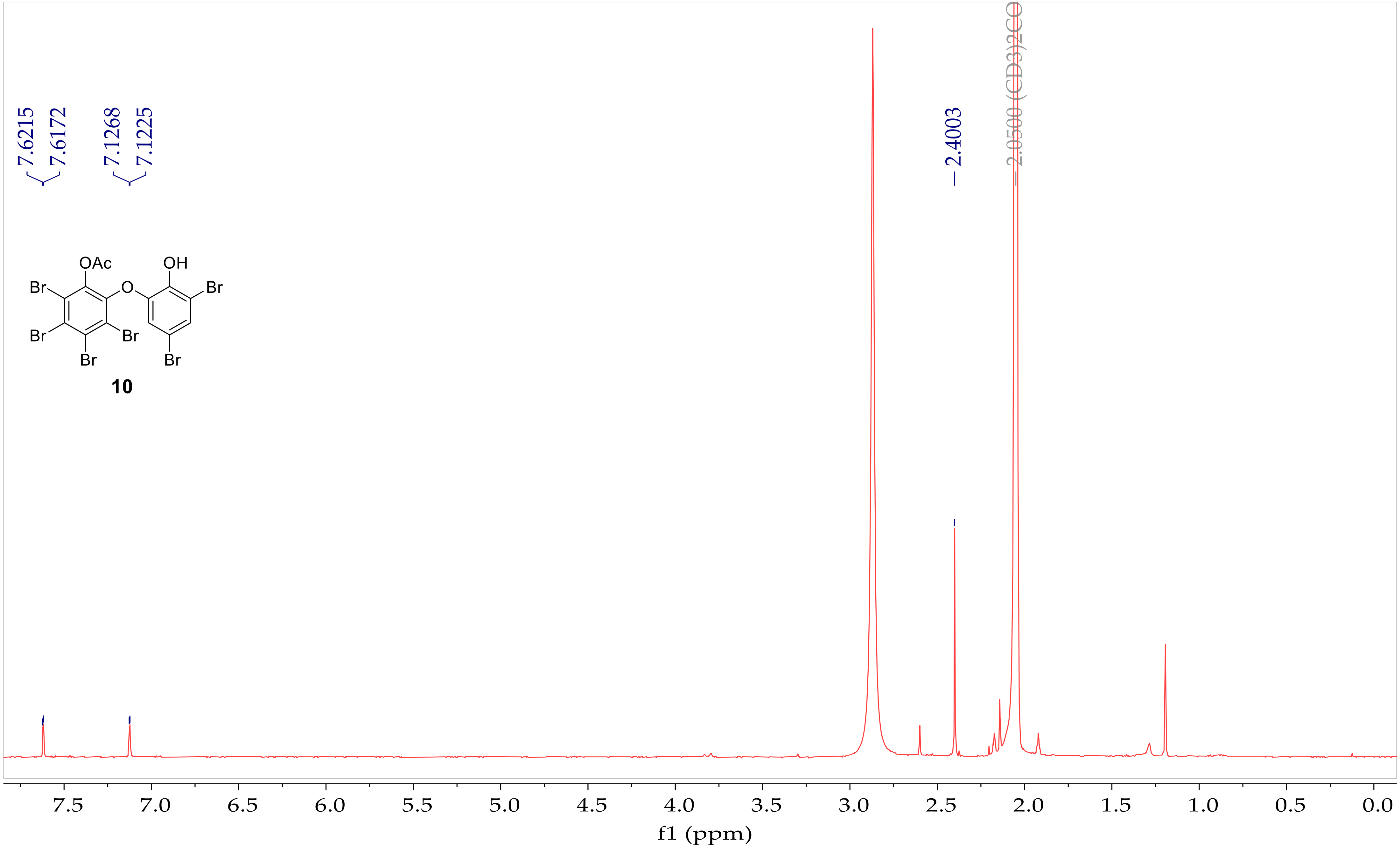

Figure S40. ^{1}H NMR spectrum of **10** ($CDCl_3$, 500 MHz)

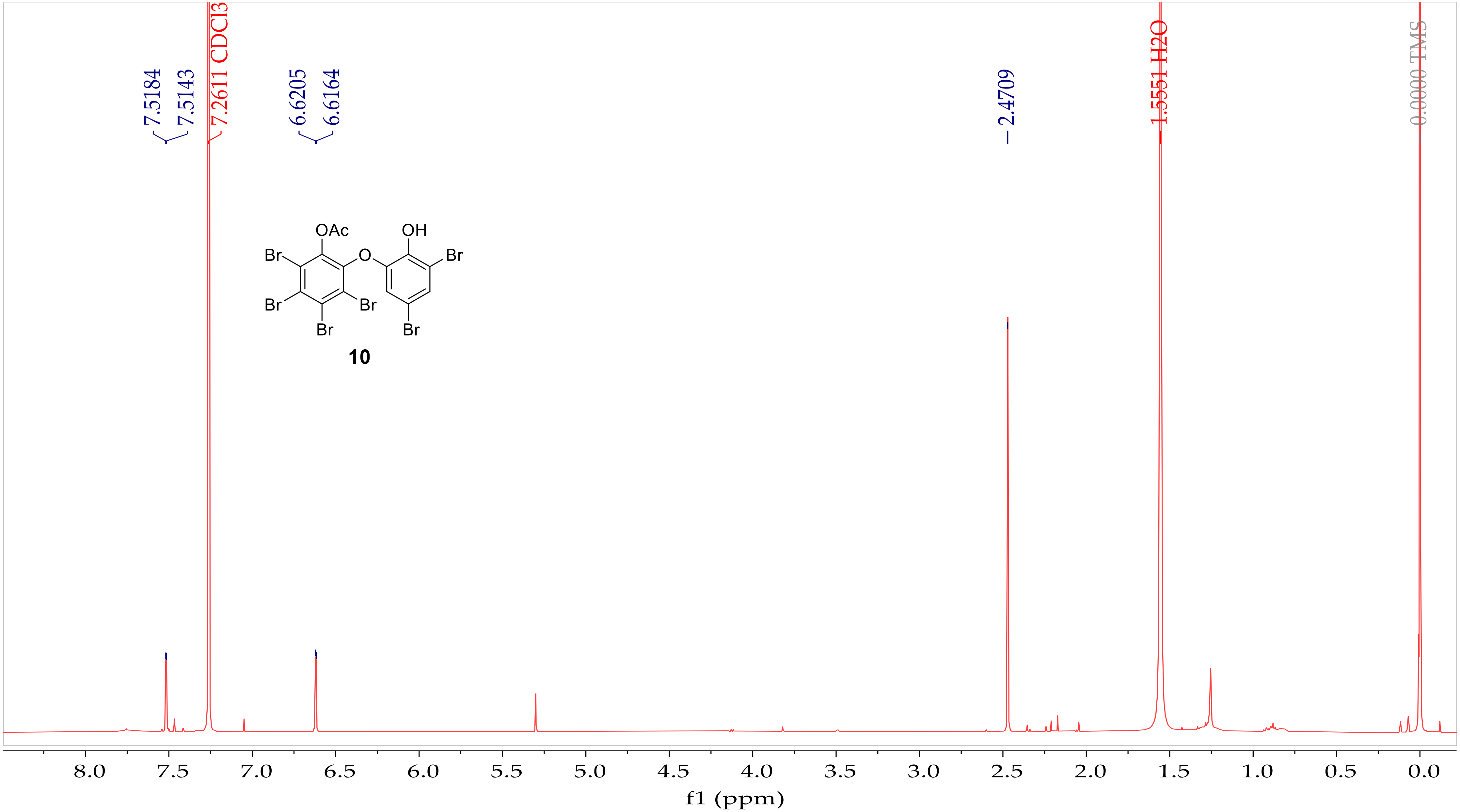

Figure S41. ^{1}H-^{1}H COSY spectrum of **10** ($CDCl_3$, 500 MHz)

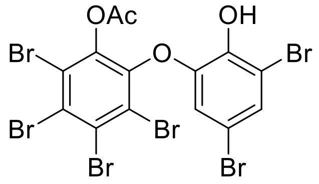

10

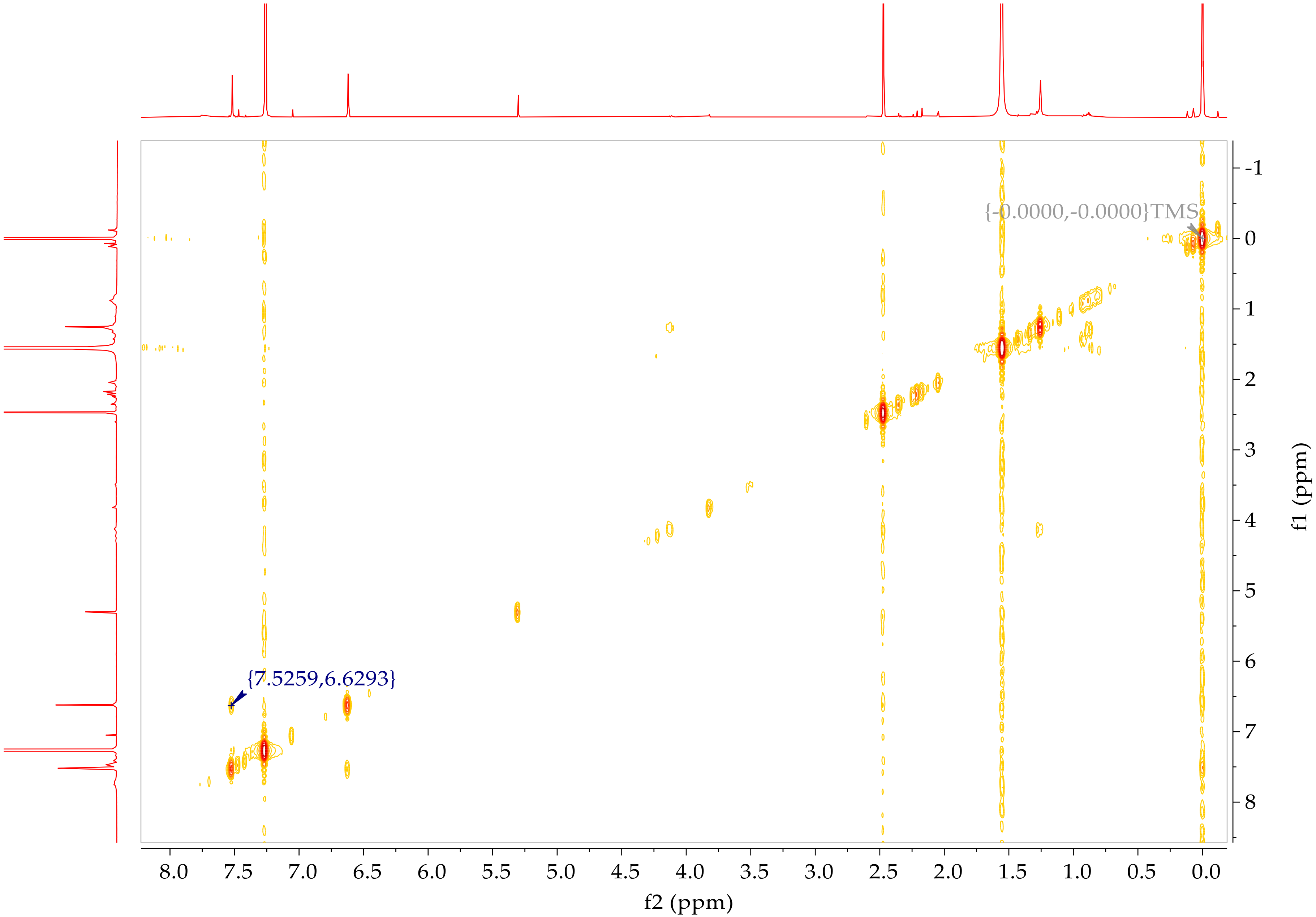

Figure S42. ^{1}H-^{13}C HSQC spectrum of **10** ($CDCl_3$, 500 MHz)

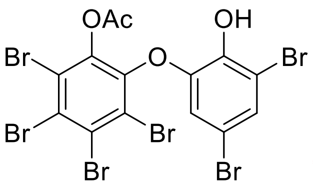

10

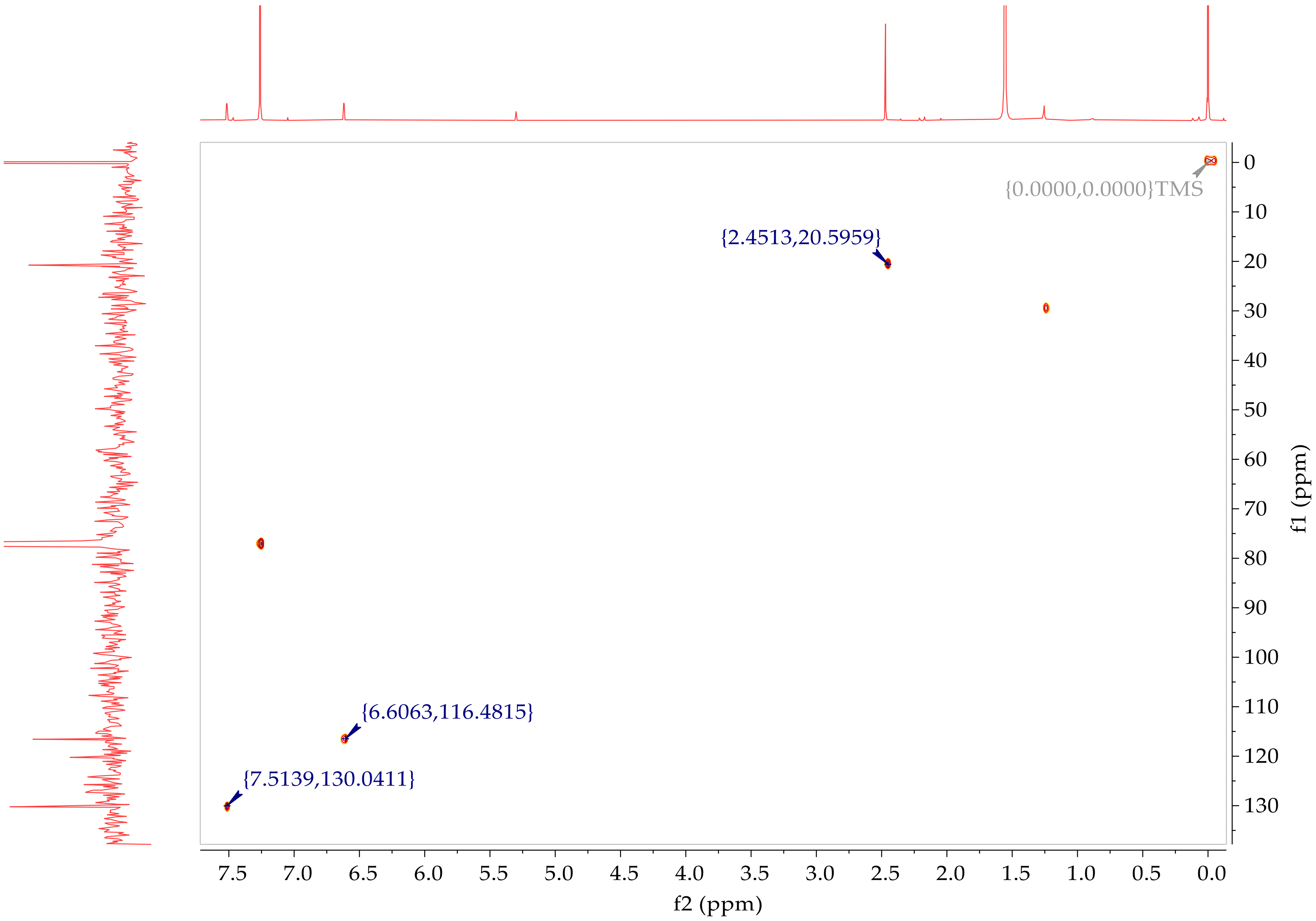

Figure S43. ^{1}H-^{13}C HMBC spectrum of **10** ($CDCl_3$, 500 MHz)

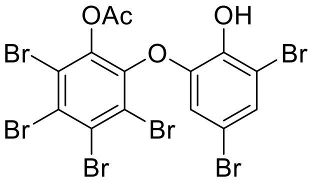

10

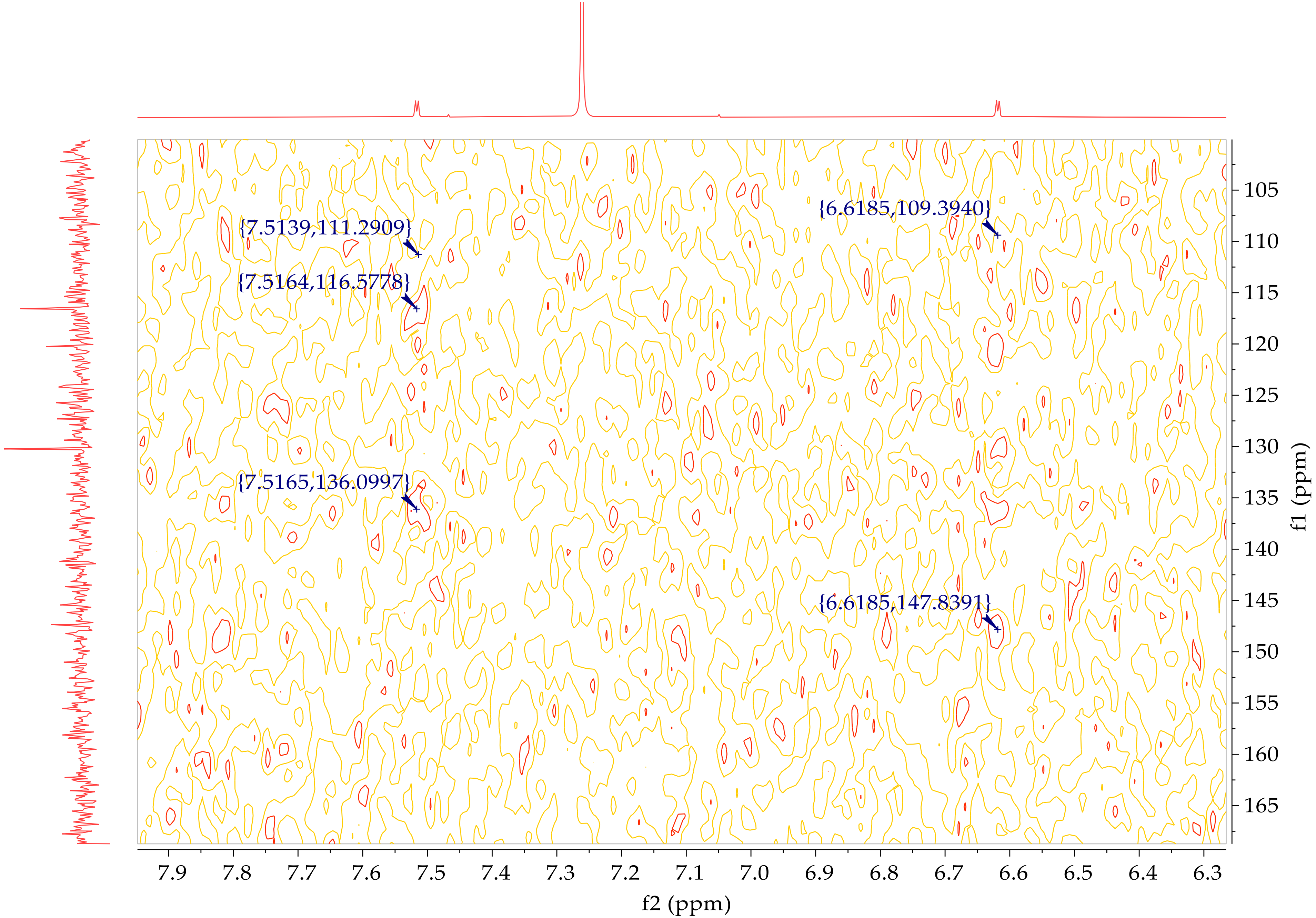

Figure S44. HRESIMS of **10**

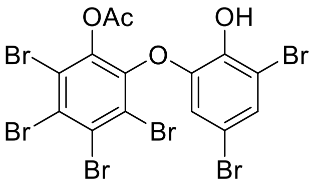

10

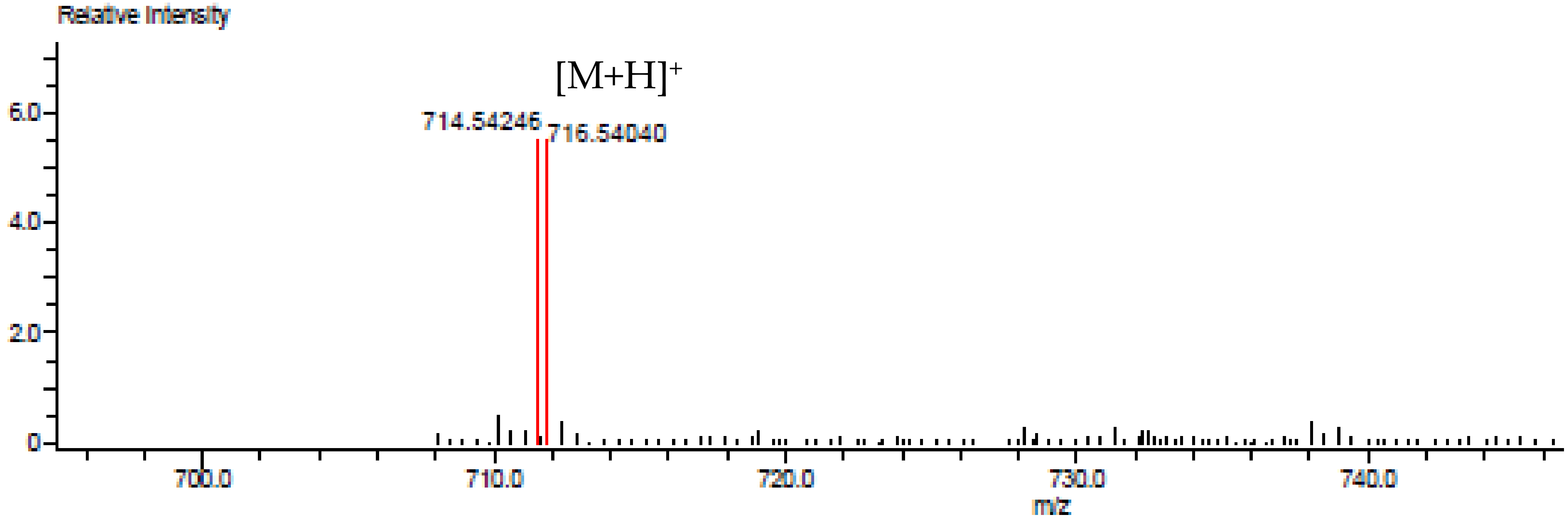

Table S34. Equilibrium Geometry DFT ωB97X-D/6-311+G(2d,p) of **10**

```
Job type: Geometry optimization.
Method: RWB97X-D
Basis set: 6-311+G(2D,P)
Number of basis functions: 810
Number of electrons: 332
Parallel Job: 16 threads

SCF model:
 A restricted hybrid HF-DFT SCF calculation will be
 performed using Pulay DIIS + Geometric Direct Minimization


Optimization:
         Step      Energy           Max Grad.       Max Dist.
            1   -16282.961523       0.039596        0.099841
            2   -16282.973455       0.009160        0.056260
            3   -16282.974471       0.004227        0.035504
            4   -16282.974697       0.001591        0.011823
            5   -16282.974728       0.000611        0.013237
            6   -16282.974736       0.000430        0.011420
```

Figure 45. HOMO-LUMO of **10**

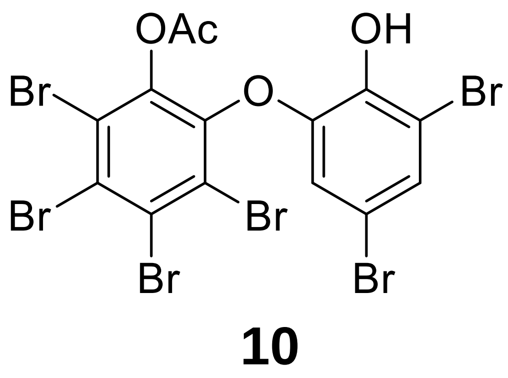

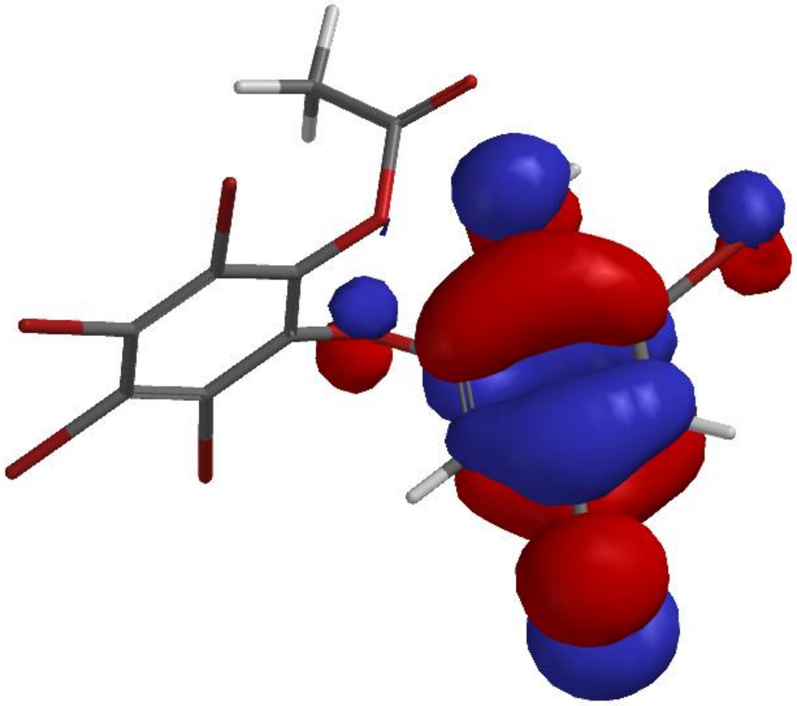

HOMO 10

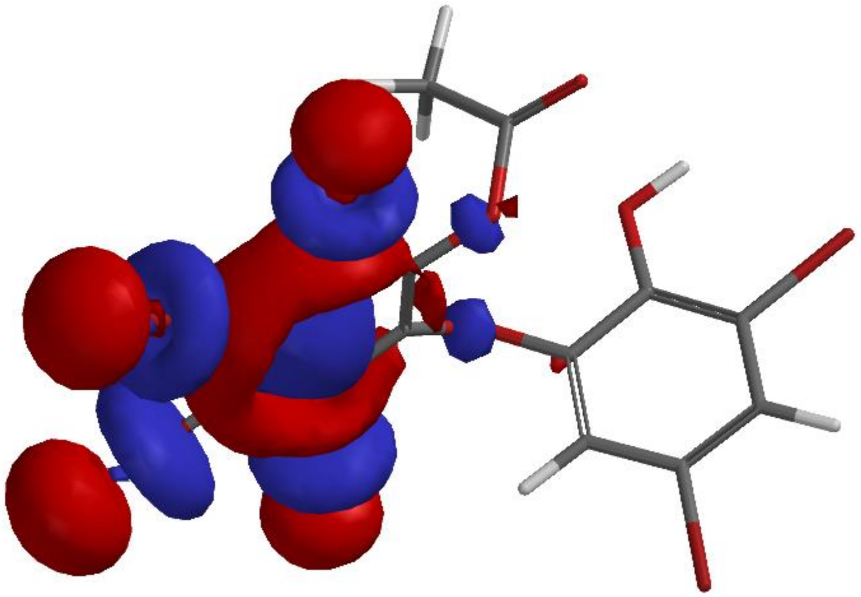

LUMO 10

Figure S46. Calculated ^{13}C NMR Chemical Shift of **10**

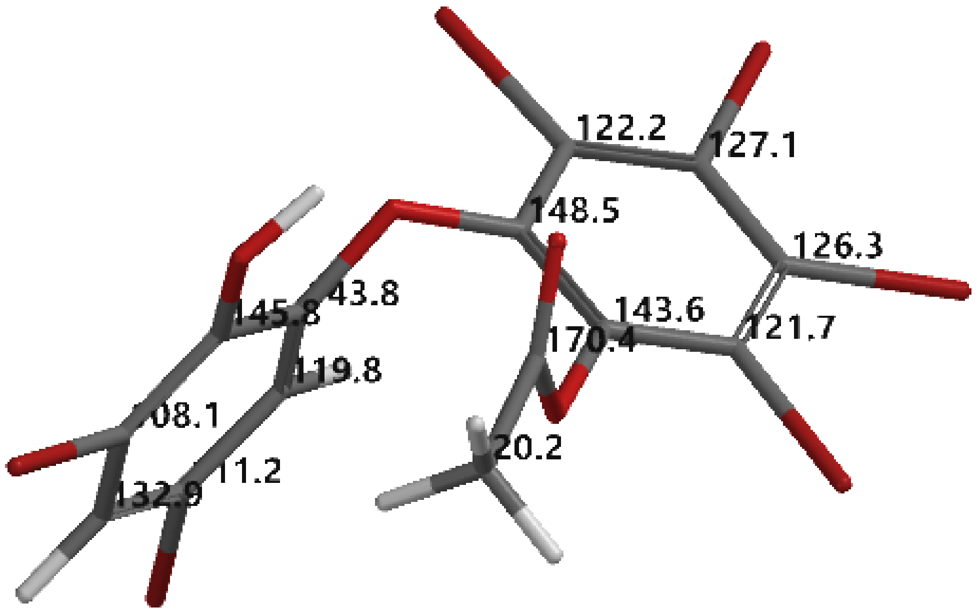

Figure S47. ^1H NMR spectrum of **11** (Me_2CO-d_6, 500 MHz)

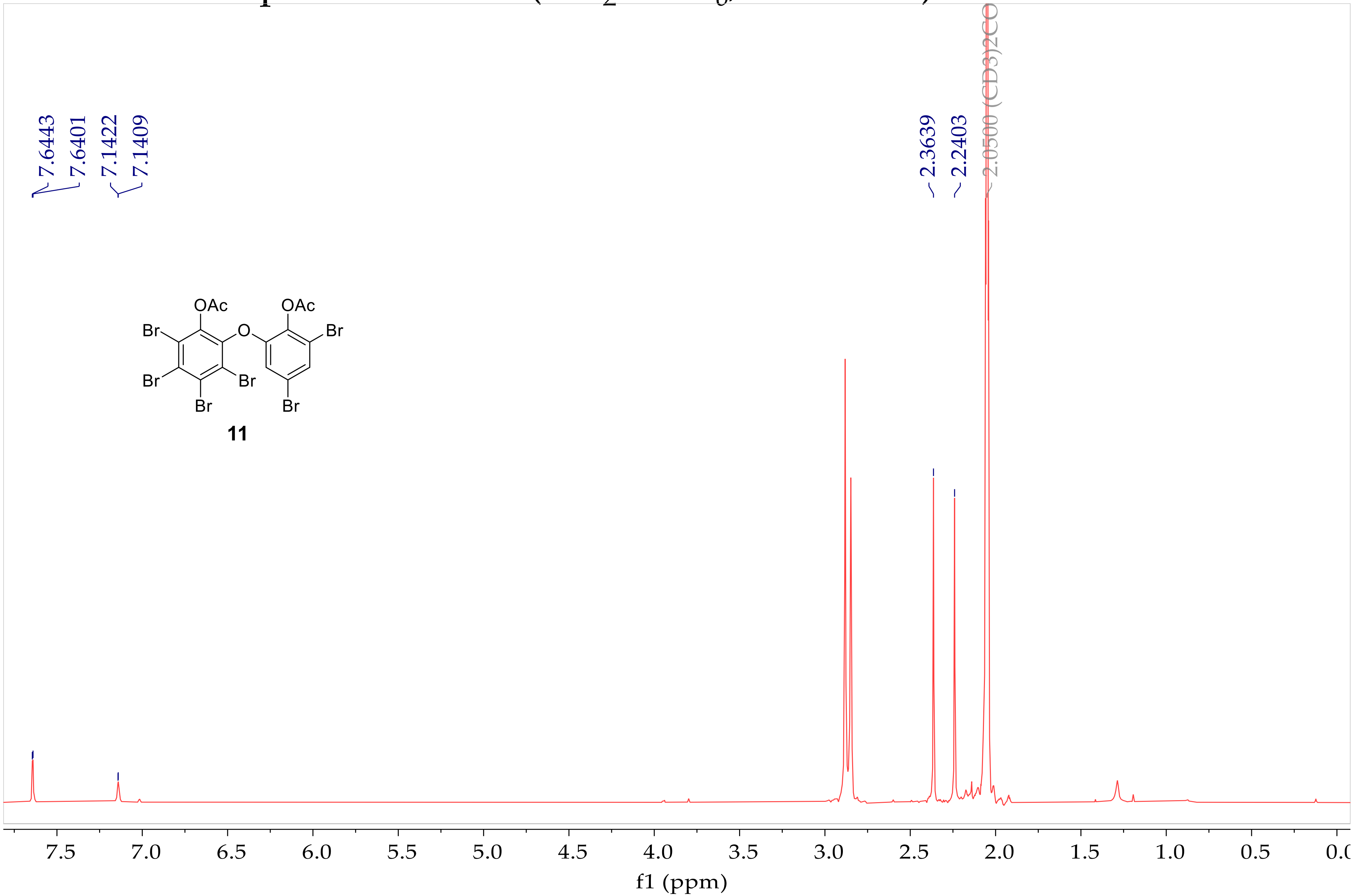

Figure S48. HRESIMS of **11**

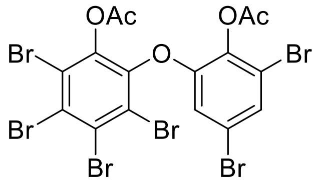

11

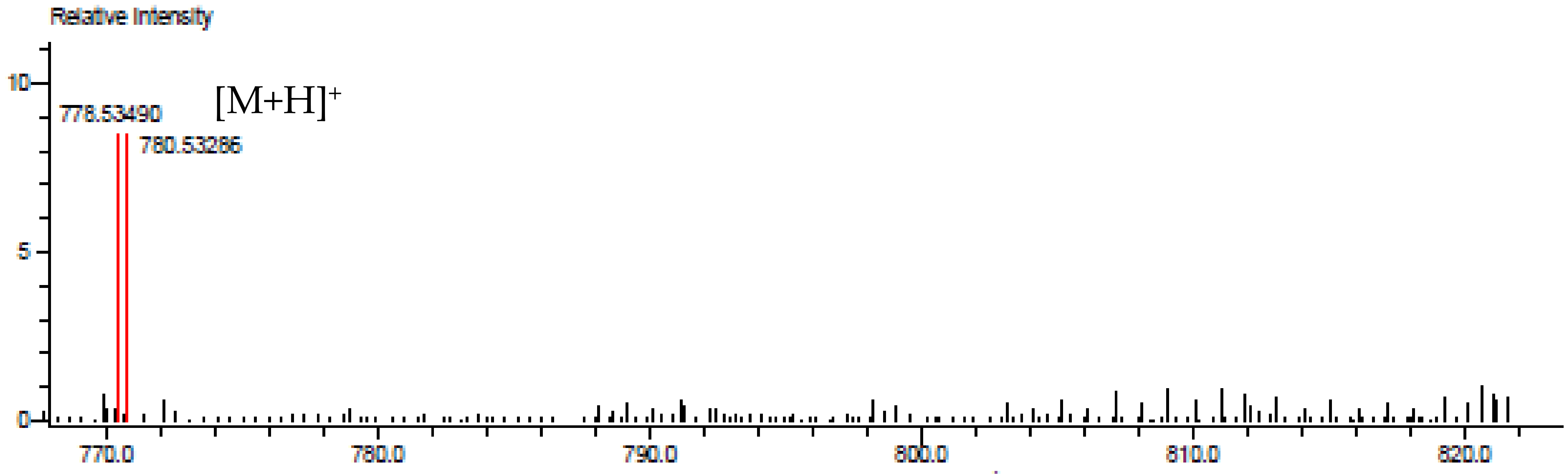

Table S35. Equilibrium Geometry DFT ωB97X-D/6-311+G(2d,p) of **11**

```
Job type: Geometry optimization.
Method: RWB97X-D
Basis set: 6-311+G(2D,P)
Number of basis functions: 903
Number of electrons: 354
Parallel Job: 16 threads

SCF model:
 A restricted hybrid HF-DFT SCF calculation will be
 performed using Pulay DIIS + Geometric Direct Minimization


Optimization:
         Step      Energy          Max Grad.      Max Dist.
            1   -16435.596645      0.053312       0.109488
            2   -16435.613906      0.009515       0.084379
            3   -16435.615225      0.005318       0.043742
            4   -16435.615707      0.005185       0.060976
            5   -16435.615985      0.005939       0.074446
            6   -16435.616297      0.004642       0.075823
            7   -16435.616595      0.003118       0.101938
            8   -16435.616834      0.003743       0.090140
            9   -16435.617030      0.004368       0.036928
           10   -16435.617070      0.002291       0.040489
           11   -16435.617133      0.000930       0.015841
           12   -16435.617140      0.000620       0.014268
```

Figure S49. HOMO-LUMO of **11**

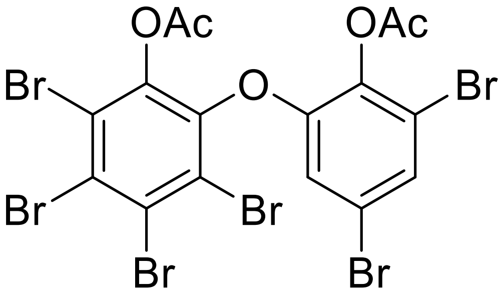

11

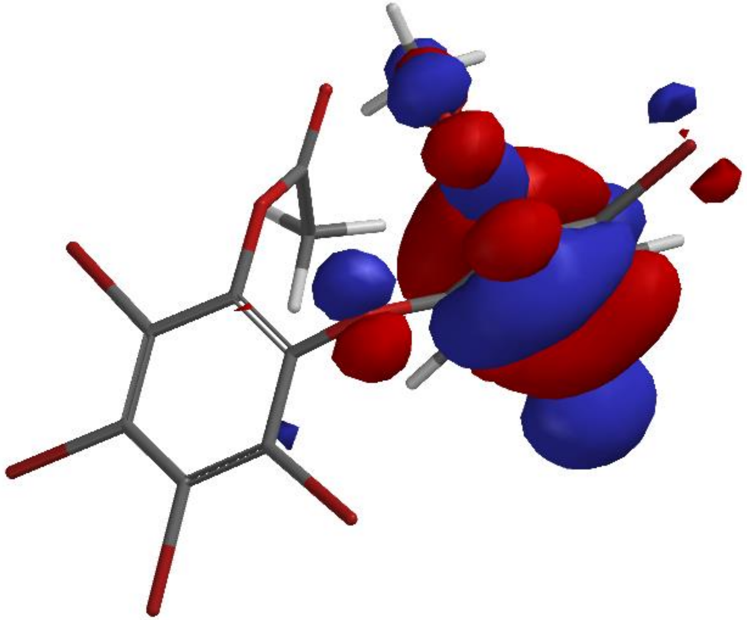

HOMO 11

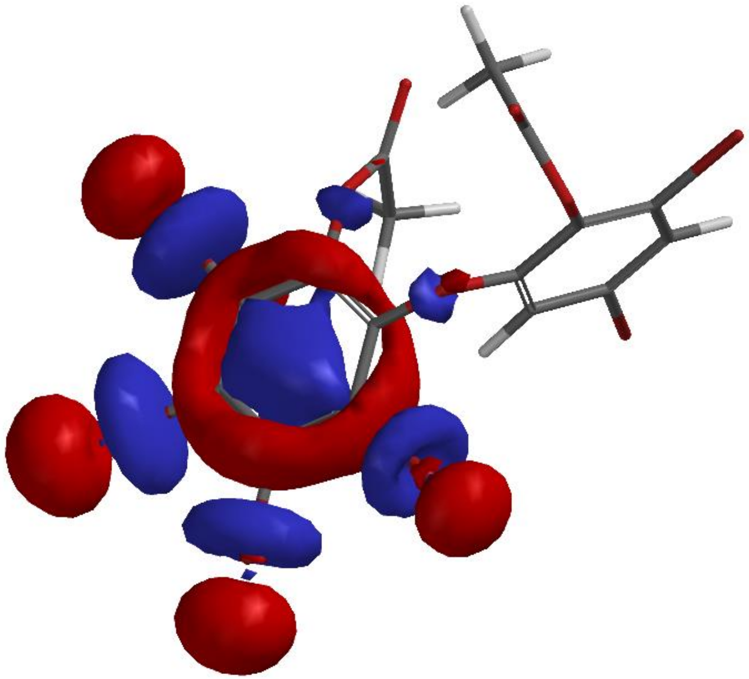

LUMO 11

Figure S50. Calculated ^{13}C NMR Chemical Shift of **11**

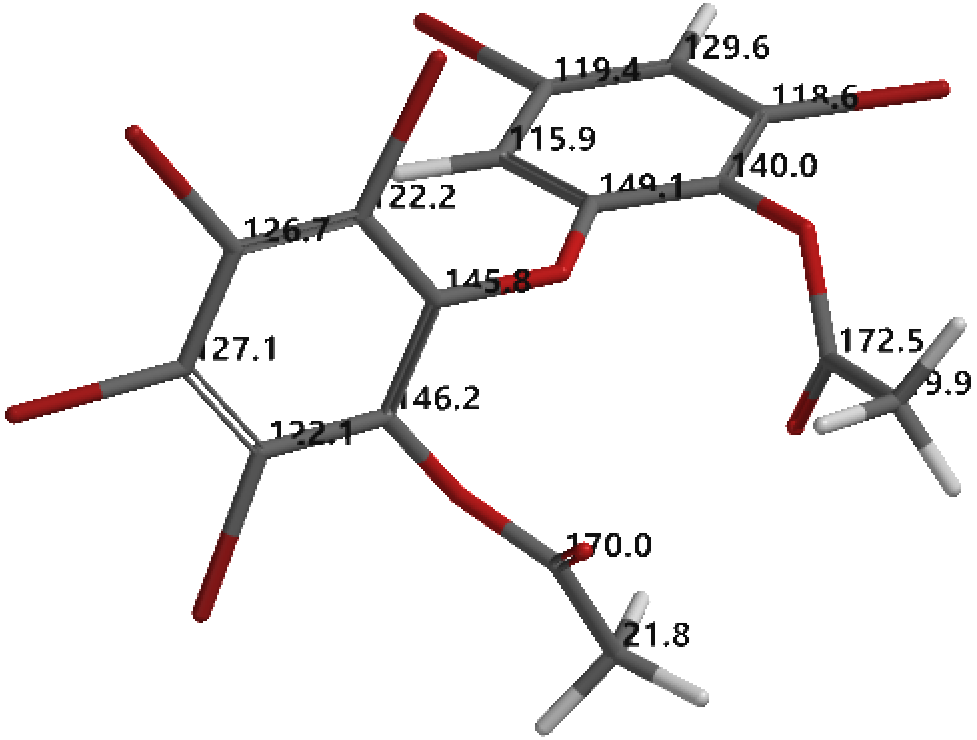

Figure S51. ^{1}H NMR spectrum of **12** (Me_2CO-d_6, 500 MHz)

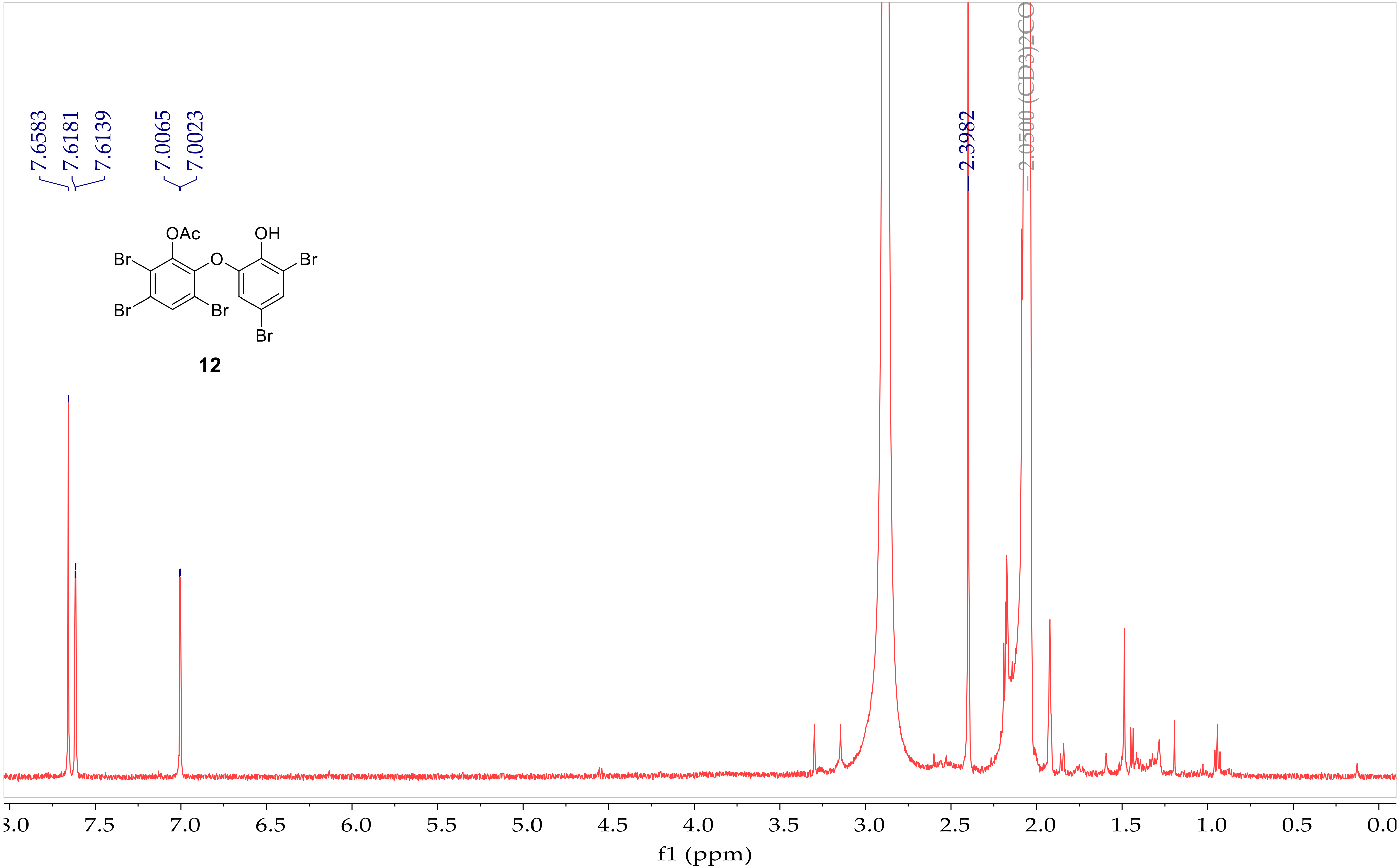

Figure S52. ^{1}H NMR spectrum of **12** ($CDCl_3$, 500 MHz)

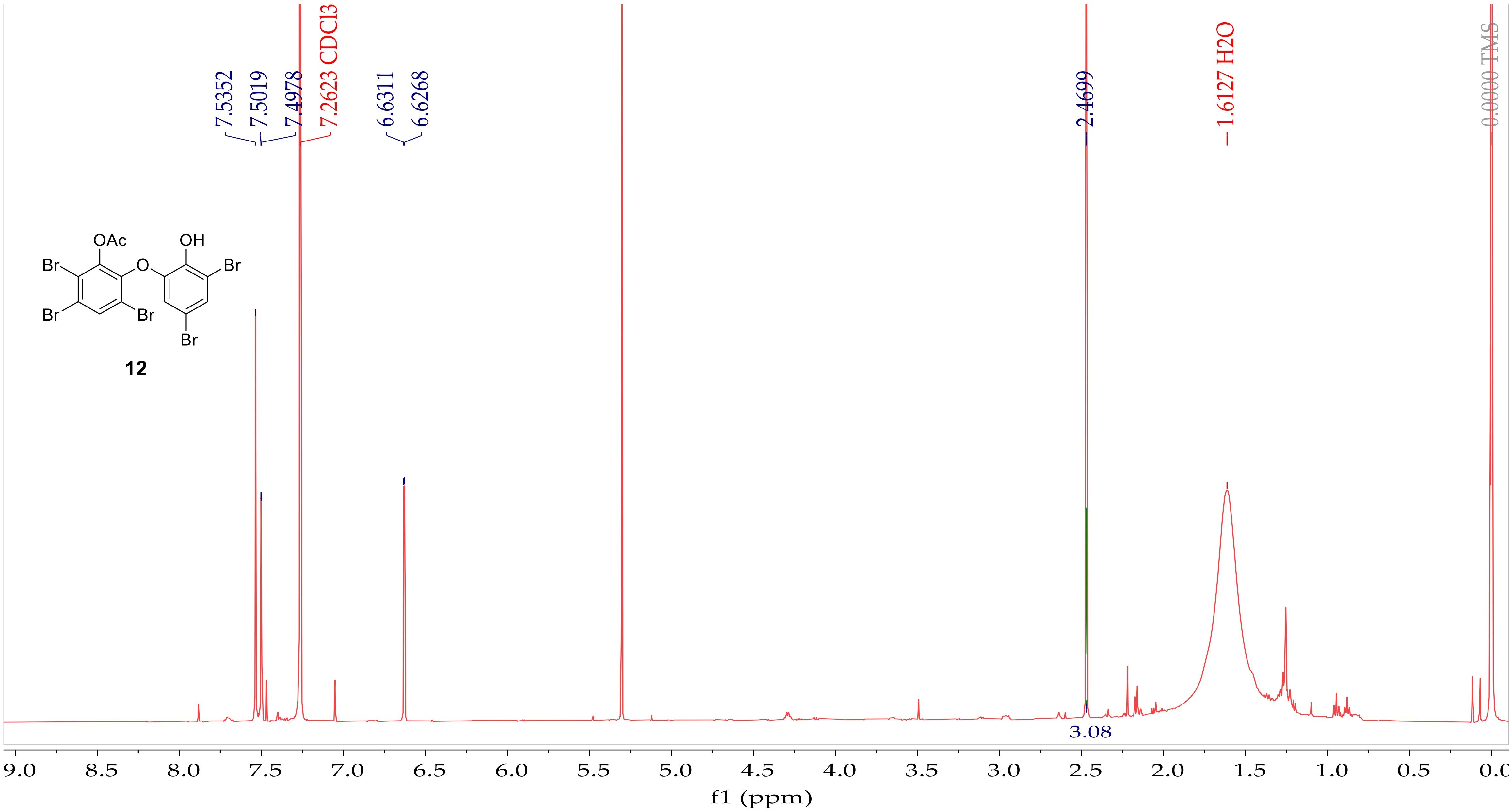

Figure S53. ^{1}H-^{1}H COSY spectrum of **12** ($CDCl_3$, 500 MHz)

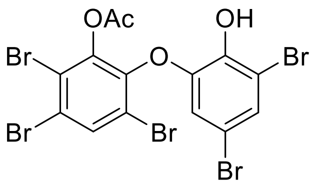

12

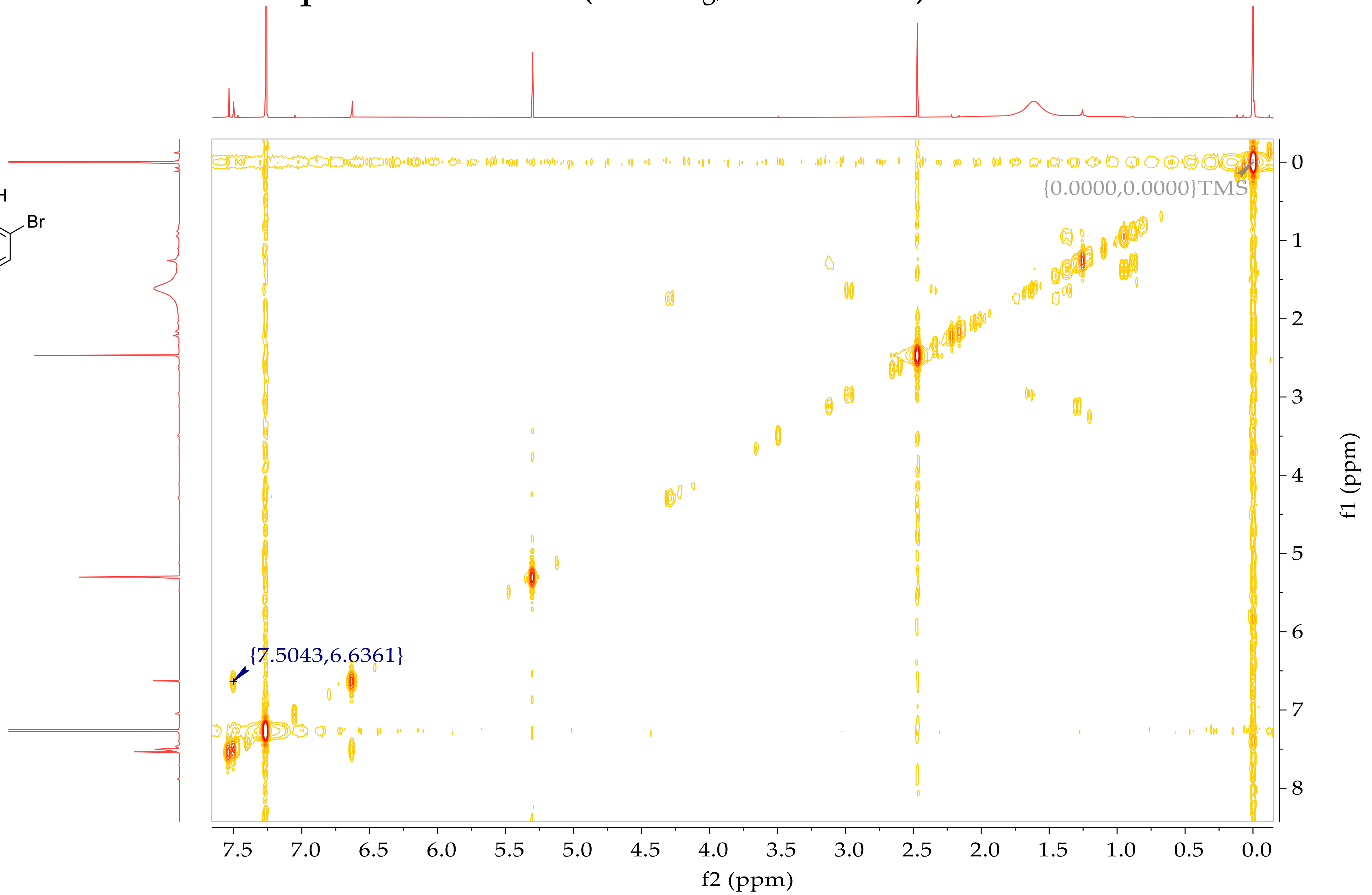

Figure S54. ^{1}H-^{13}C HSQC spectrum of **12** ($CDCl_3$, 500 MHz)

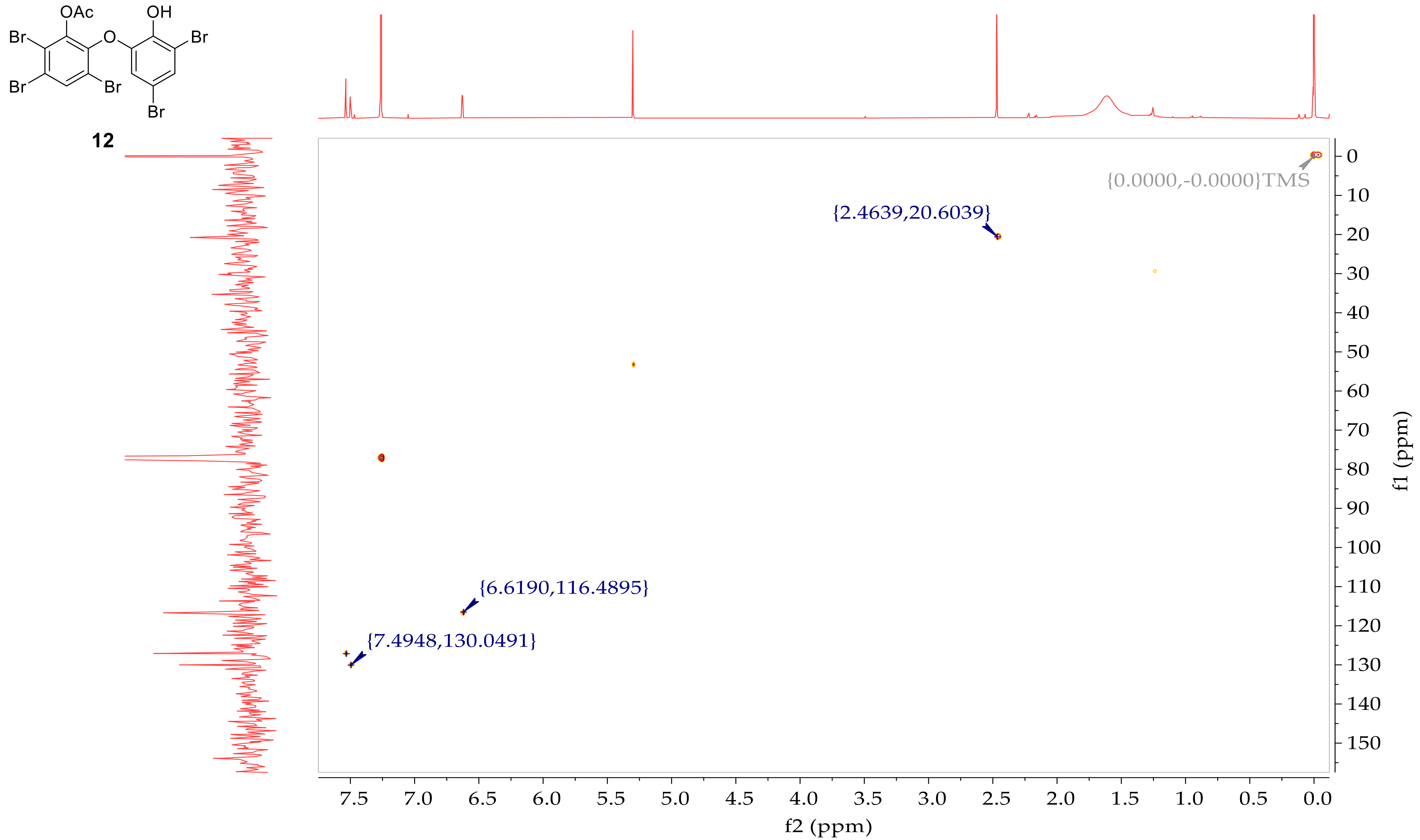

Figure S55. ^{1}H-^{13}C HMBC spectrum of **12** ($CDCl_3$, 500 MHz)

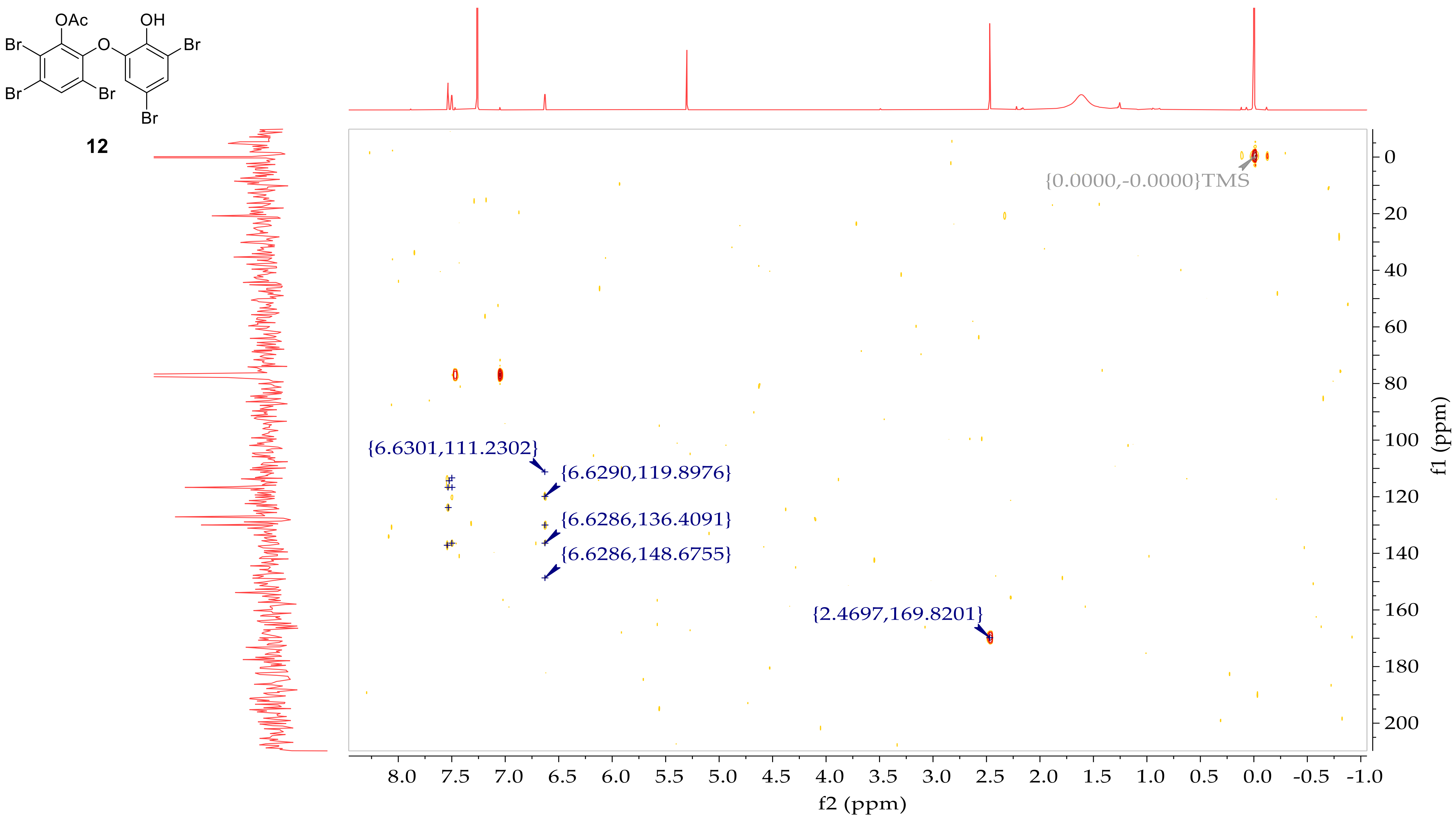

Figure S56. HRESIMS of **12**

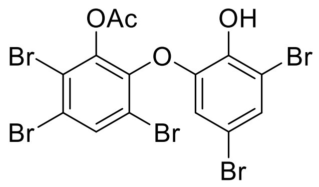

12

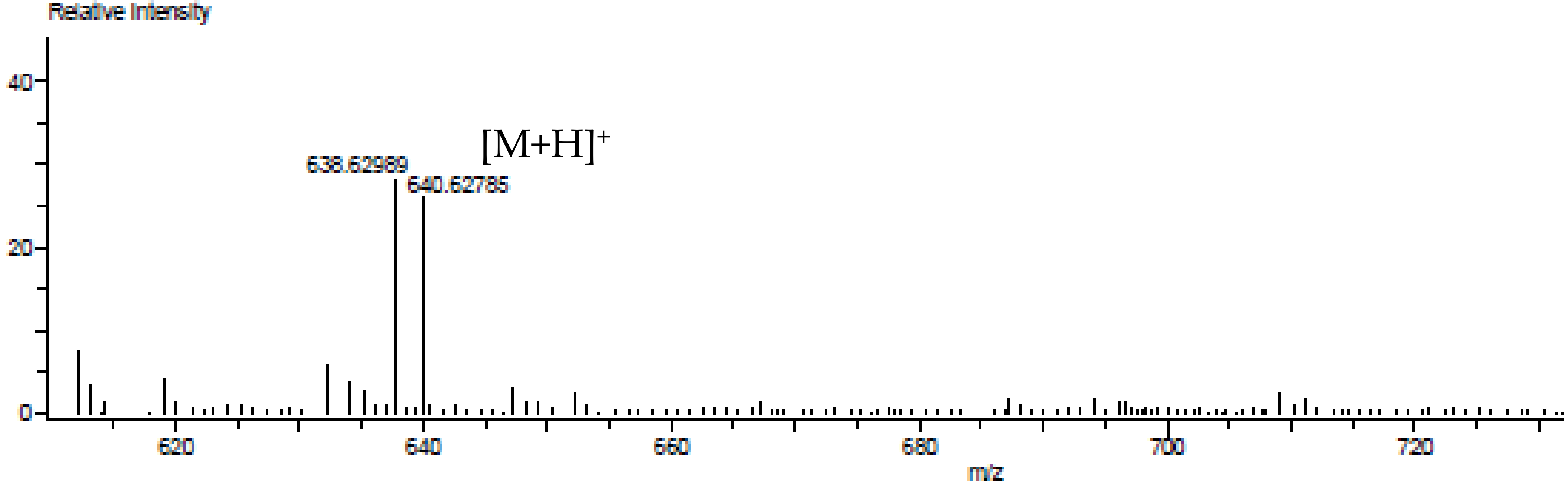

Table S36. Equilibrium Geometry DFT ωB97X-D/6-311+G(2d,p) of **12**

```
Job type: Geometry optimization.
Method: RWB97X-D
Basis set: 6-311+G(2D,P)
Number of basis functions: 768
Number of electrons: 298
Parallel Job: 16 threads

SCF model:
 A restricted hybrid HF-DFT SCF calculation will be
 performed using Pulay DIIS + Geometric Direct Minimization


Optimization:
         Step      Energy           Max Grad.      Max Dist.
            1   -13709.404352       0.038647       0.111242
            2   -13709.414372       0.014645       0.050709
            3   -13709.415618       0.004164       0.040775
            4   -13709.415825       0.001370       0.009811
            5   -13709.415855       0.000568       0.007578
            6   -13709.415859       0.000290       0.008302
```

Figure S57. HOMO-LUMO of **12**

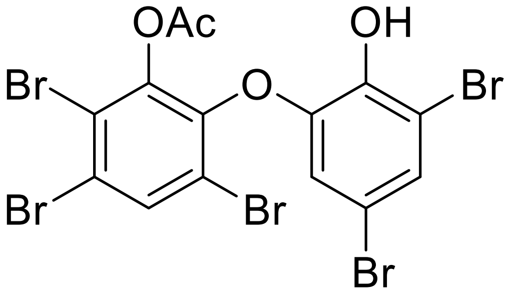

12

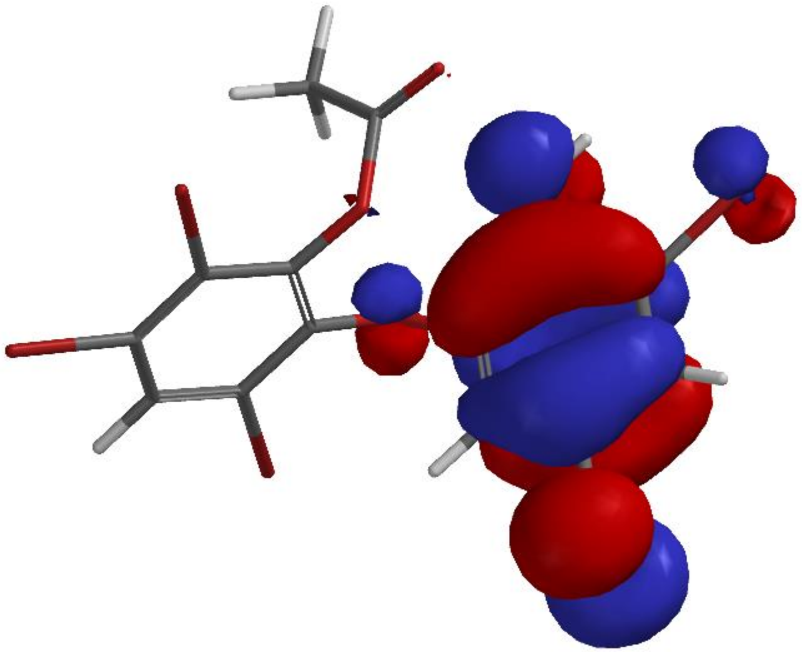

HOMO 12

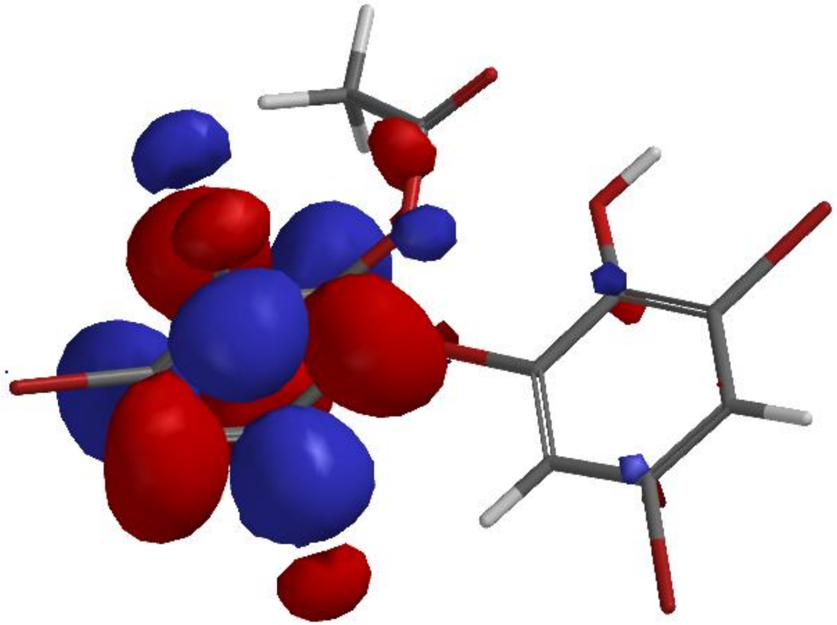

LUMO 12

Figure S58. Calculated ^{13}C NMR Chemical Shift of **12**

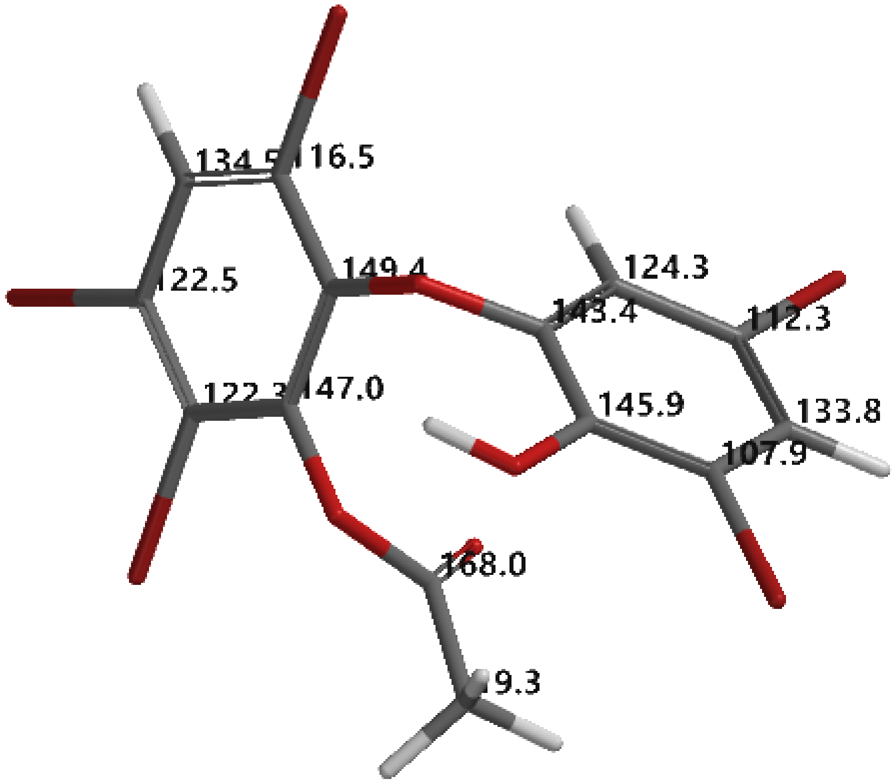

Figure S59. ^{1}H NMR spectrum of **13** (Me_2CO-d_6, 500 MHz)

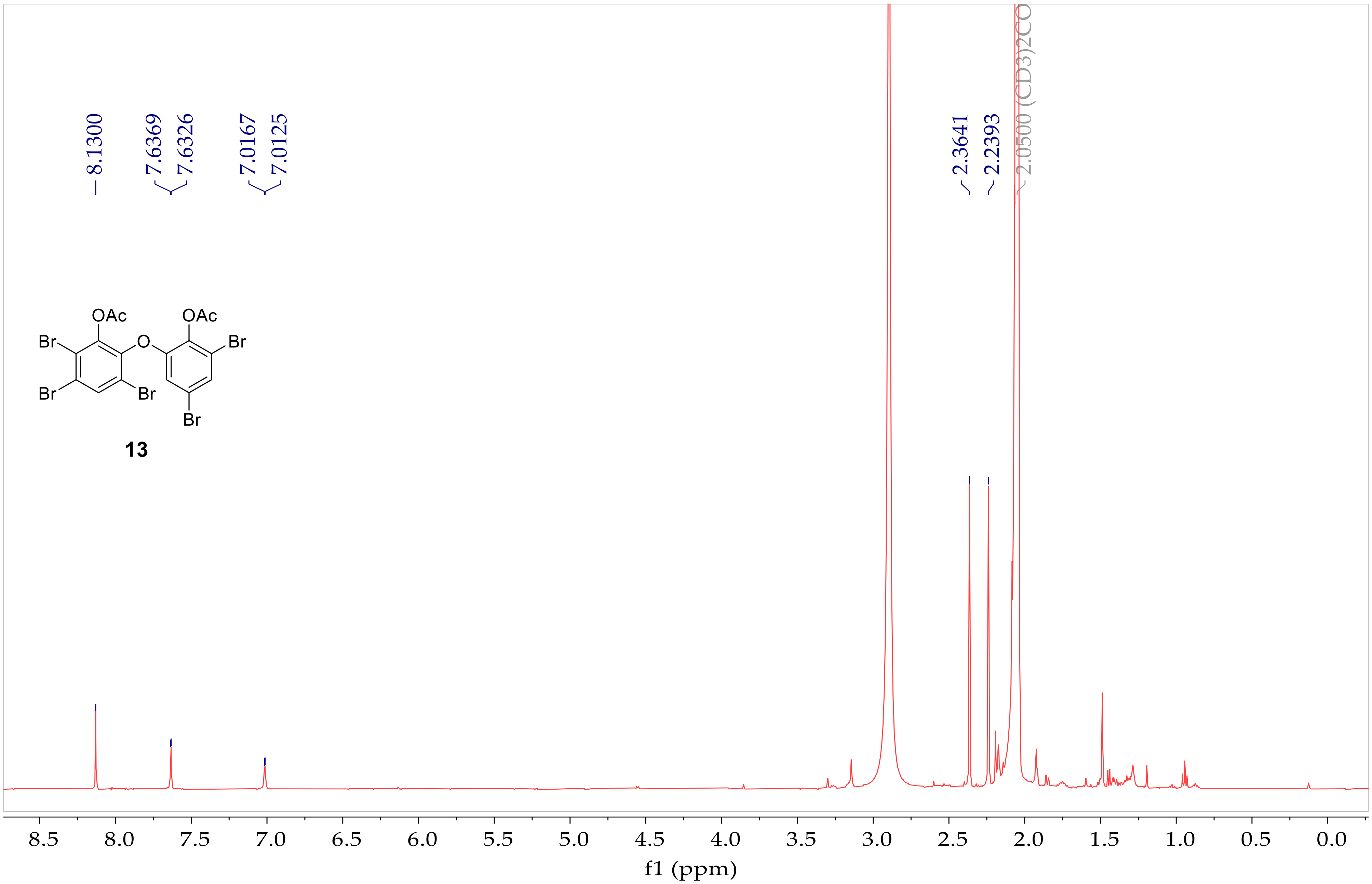

Figure S60. ^{1}H NMR spectrum of **13** ($CDCl_3$, 500 MHz)

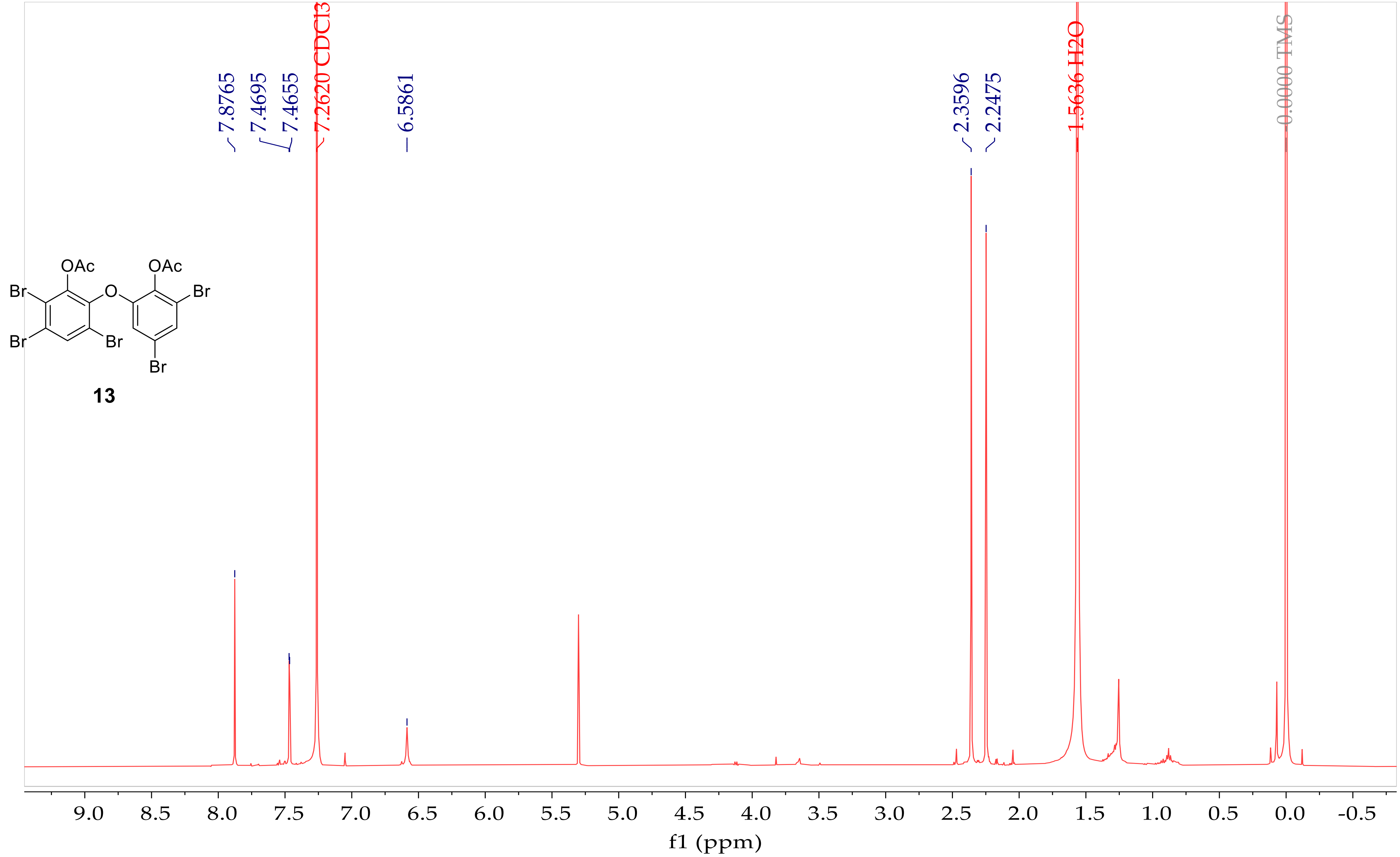

Figure S61. ^1H-^1H COSY spectrum of **13** ($CDCl_3$, 500 MHz)

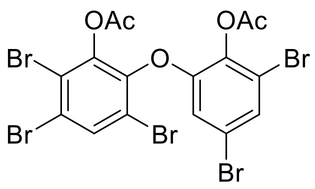

13

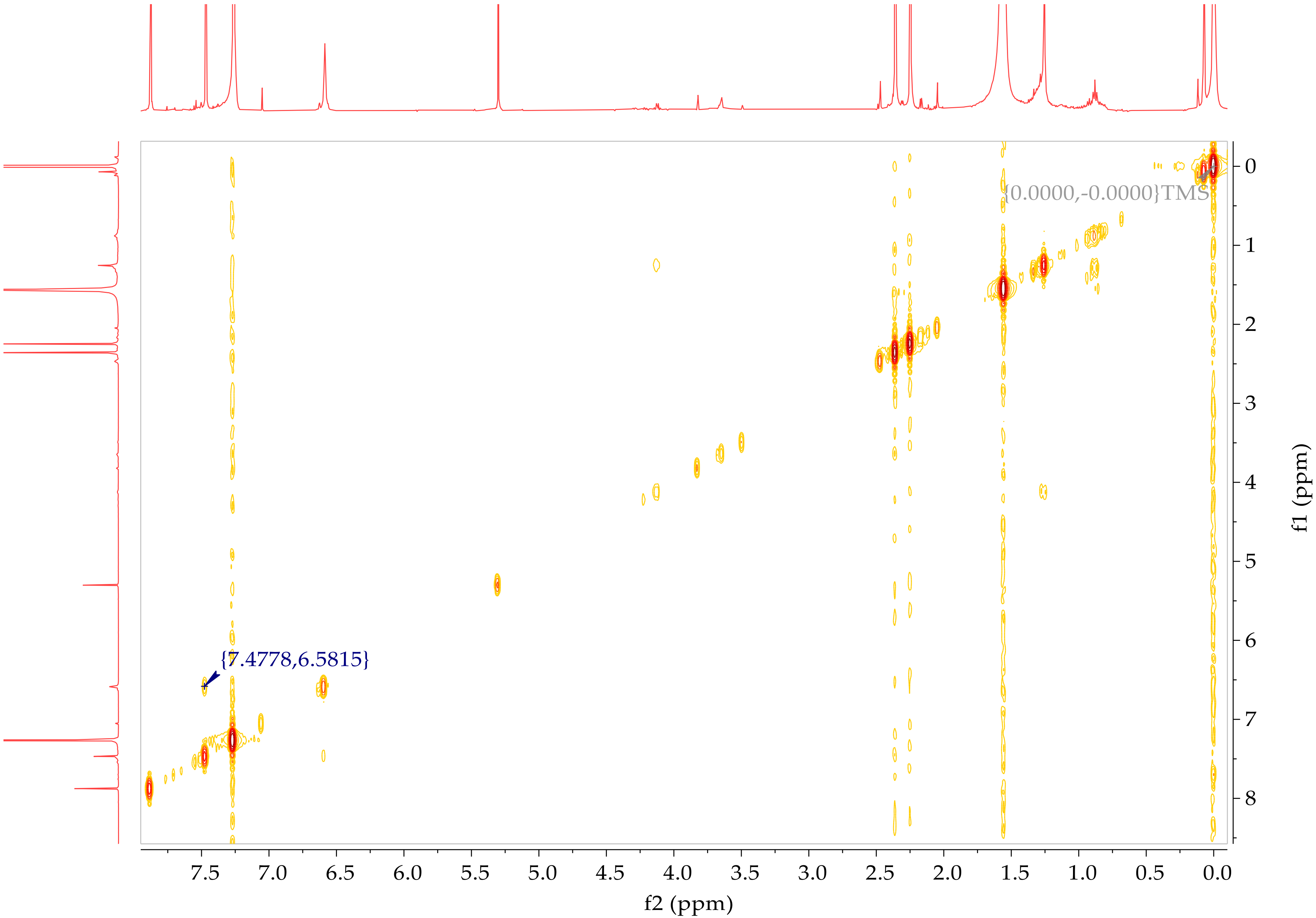

Figure S62. ^{1}H-^{13}C HSQC spectrum of **13** ($CDCl_3$, 500 MHz)

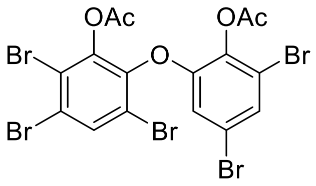

13

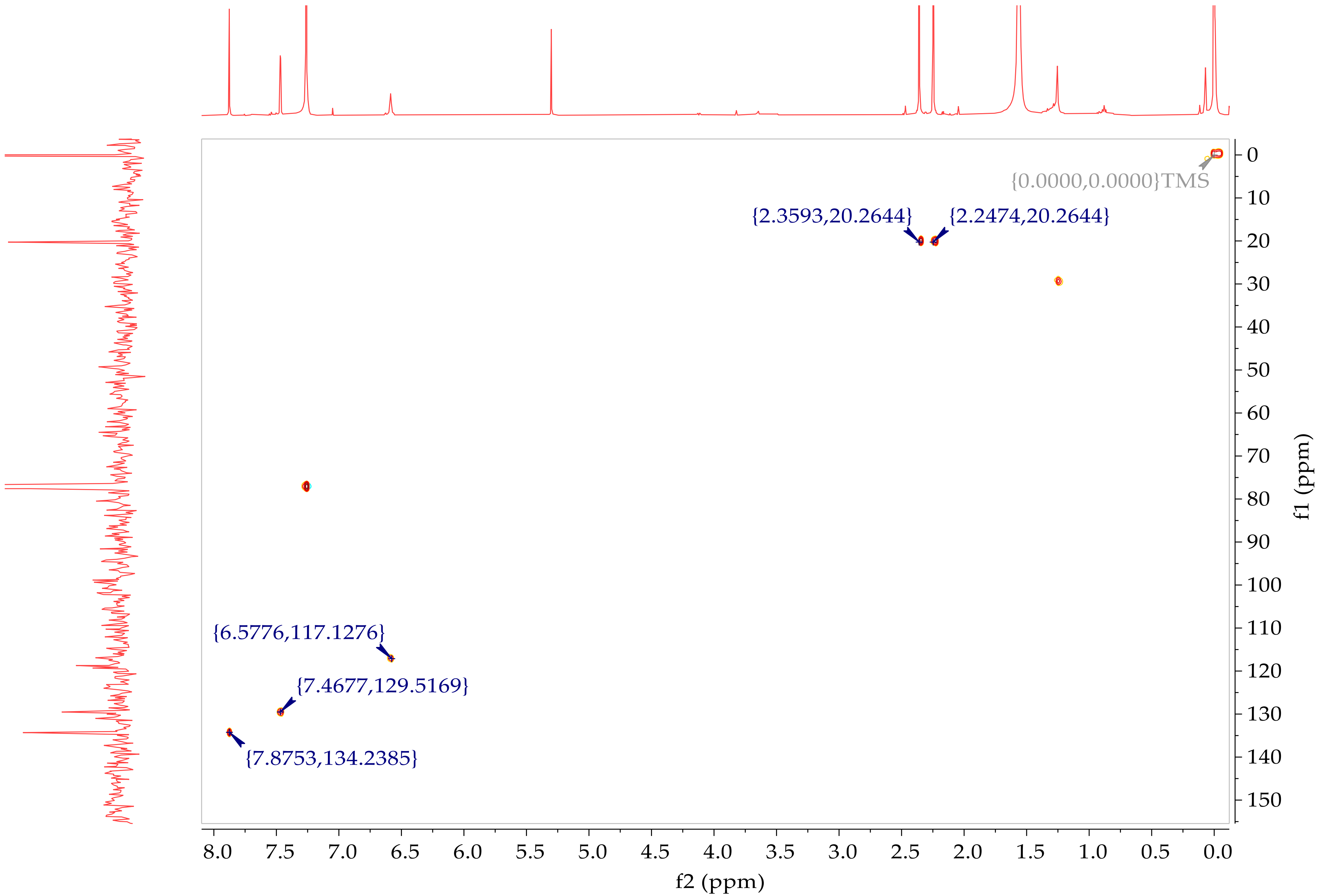

Figure S63. ^{1}H-^{13}C HMBC spectrum of **13** ($CDCl_3$, 500 MHz)

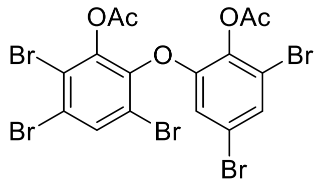

13

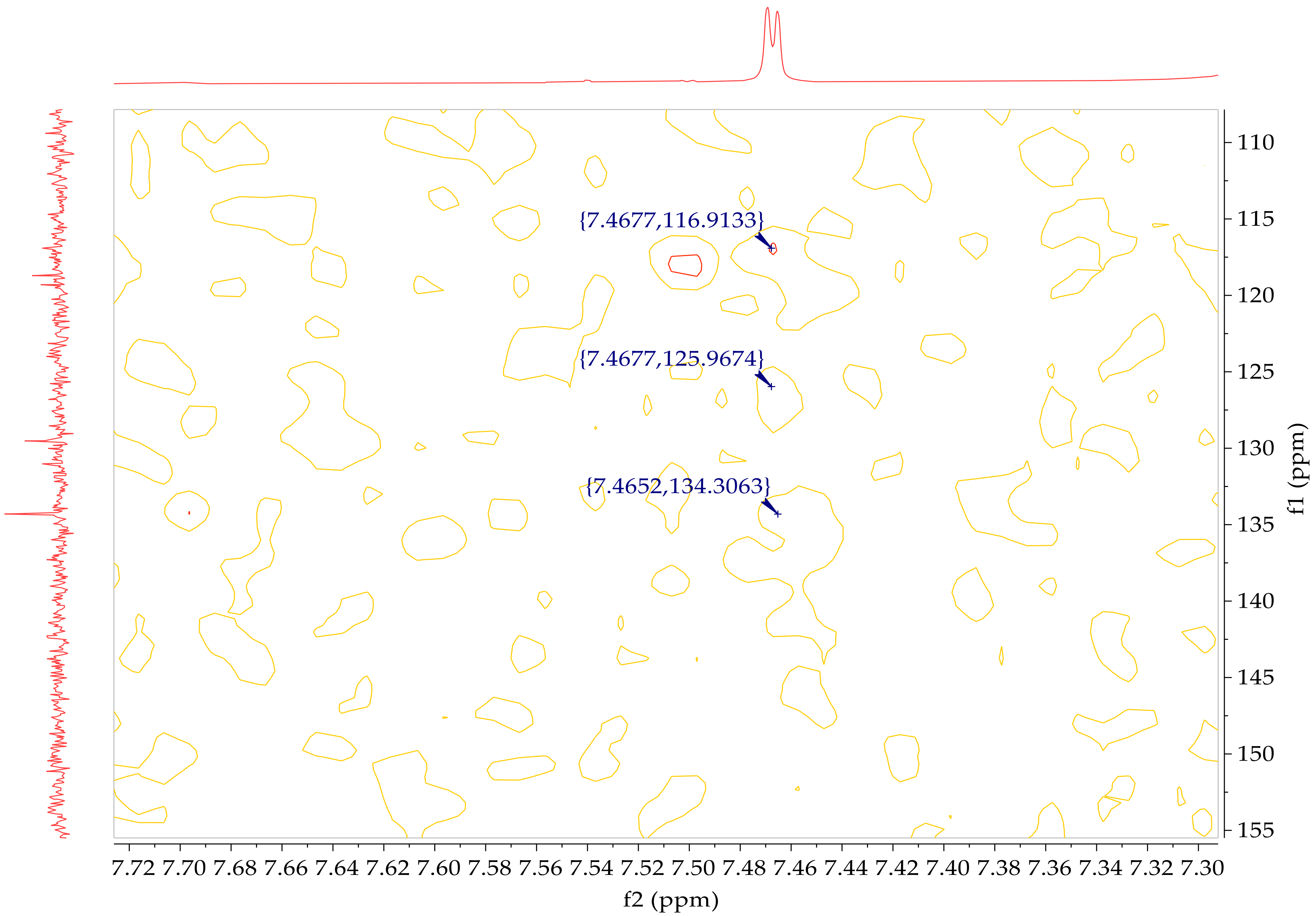

Figure S64. ^{1}H-^{13}C HMBC spectrum of **13** ($CDCl_3$, 500 MHz)

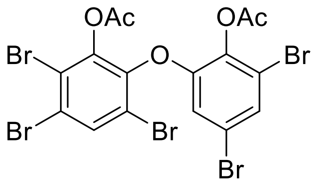

13

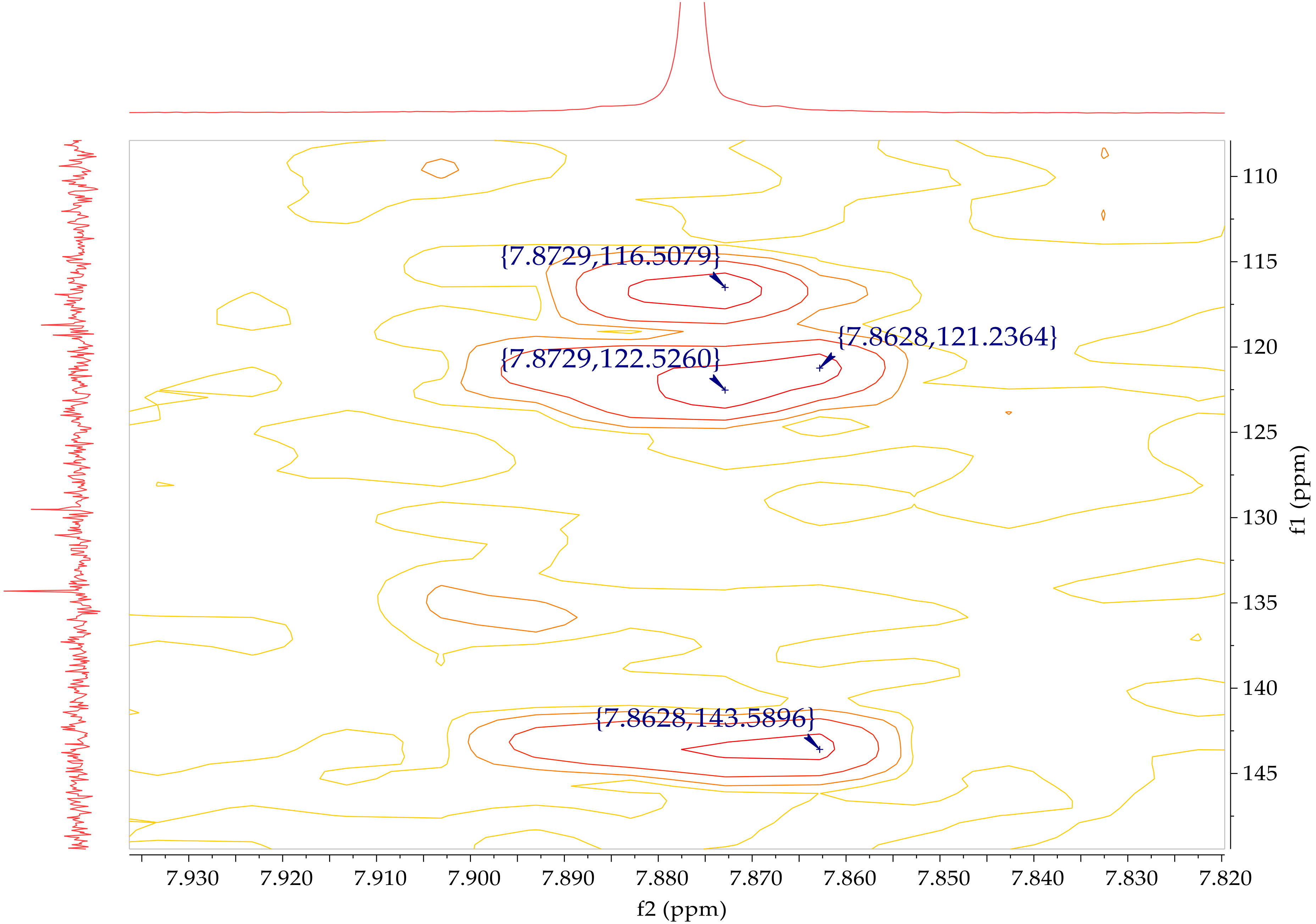

Figure S65. HRESIMS of **13**

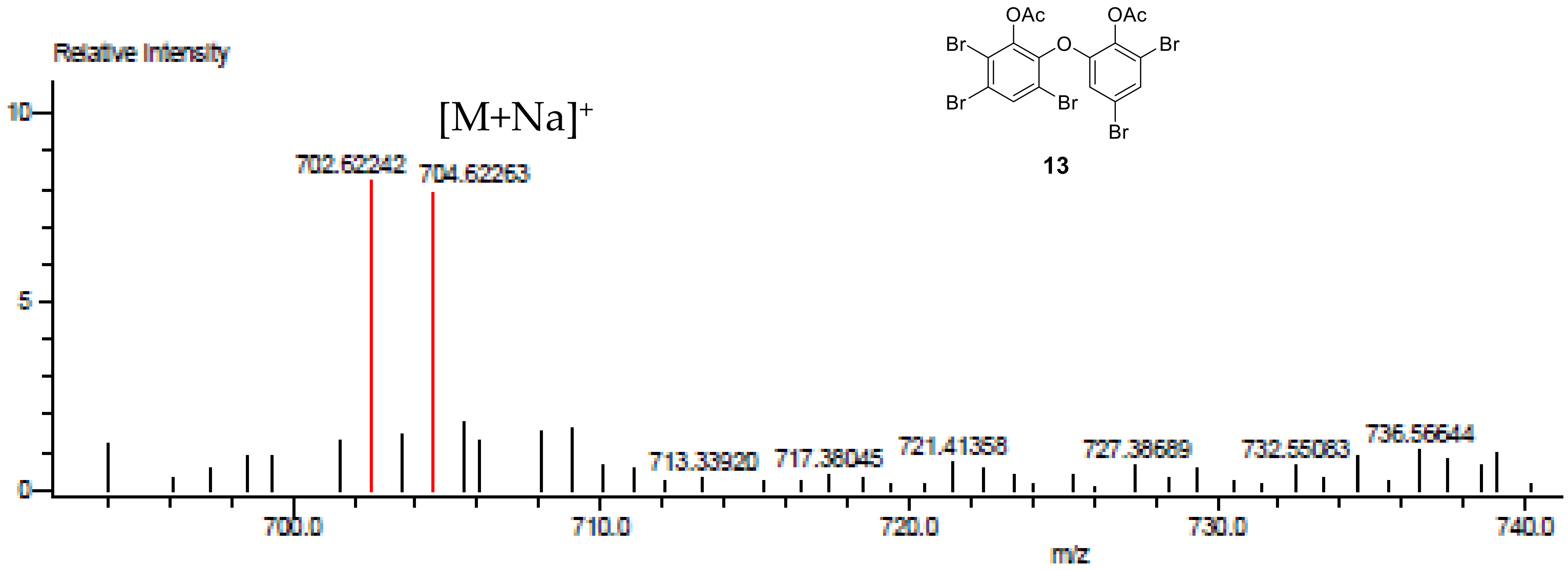

Table S37. Equilibrium Geometry DFT ωB97X-D/6-311+G(2d,p) of **13**

```
Job type: Geometry optimization.
Method: RWB97X-D
Basis set: 6-311+G(2D,P)
Number of basis functions: 861
Number of electrons: 320
Parallel Job: 16 threads

SCF model:
 A restricted hybrid HF-DFT SCF calculation will be
 performed using Pulay DIIS + Geometric Direct Minimization


Optimization:
         Step      Energy            Max Grad.      Max Dist.
            1   -13862.043081        0.045313        0.117283
            2   -13862.058089        0.010116        0.074929
            3   -13862.059689        0.005803        0.033053
            4   -13862.059993        0.001959        0.034129
            5   -13862.060035        0.001562        0.008661
            6   -13862.060046        0.000761        0.014733
            7   -13862.060050        0.000256        0.008008
```

Figure S66. HOMO-LUMO of **13**

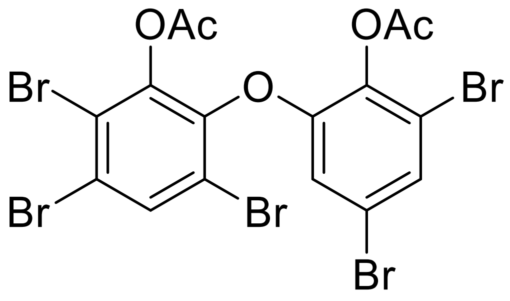

13

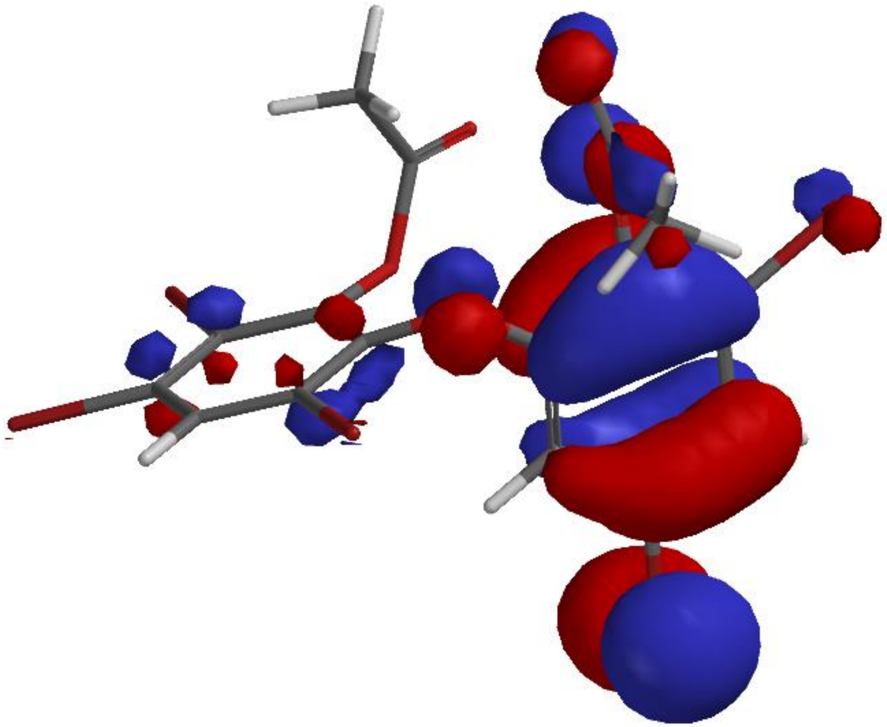

HOMO 13

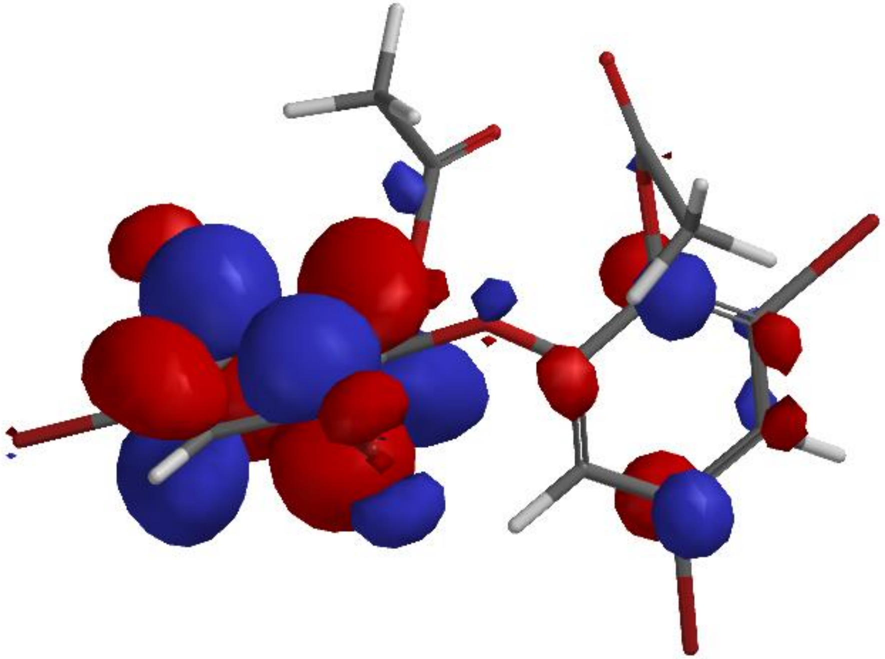

LUMO 13

Figure S67. Calculated ^{13}C NMR Chemical Shift of **13**

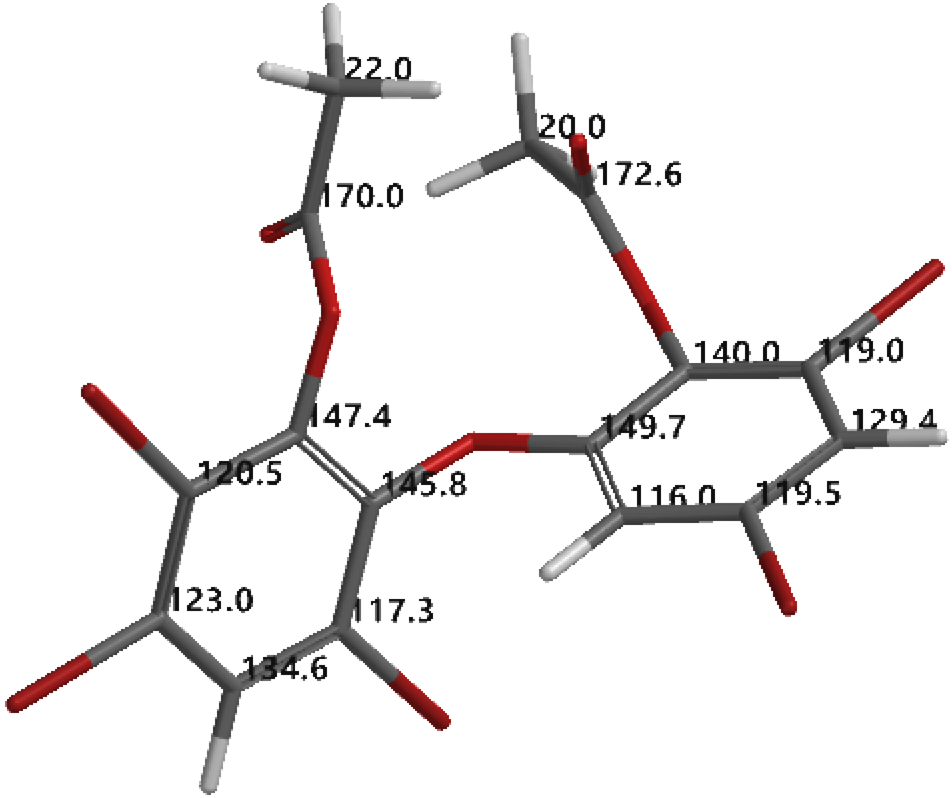

Figure S68. ^{1}H NMR spectrum of **14** (as a mixture with compound 2) (CD_3OD, 500 MHz)

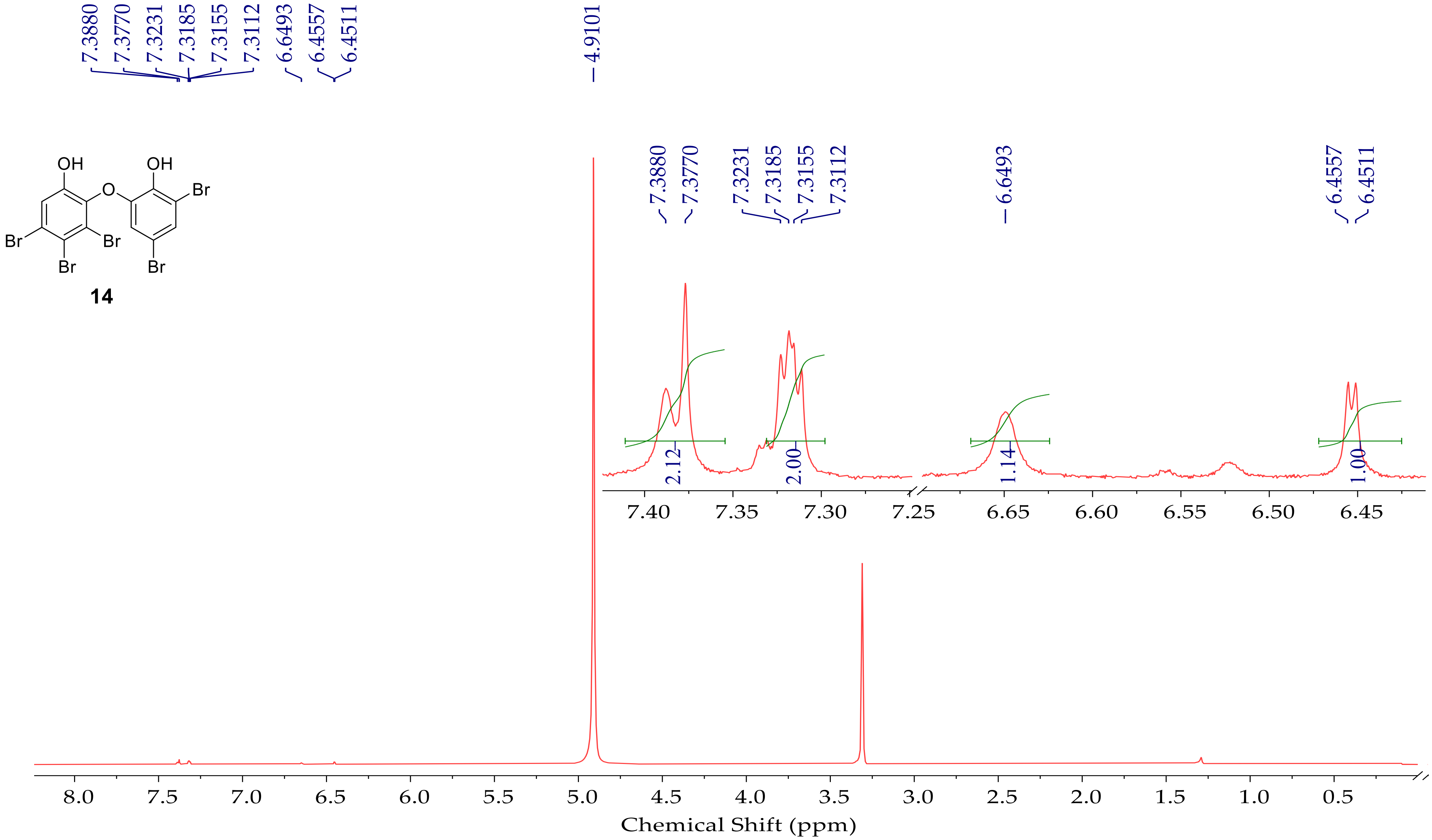

14

Table S38. Equilibrium Geometry DFT ωB97X-D/6-311+G(2d,p) of **14**

```
Job type: Geometry optimization.
Method: RWB97X-D
Basis set: 6-311+G(2D,P)
Number of basis functions: 675
Number of electrons: 276
Parallel Job: 16 threads

SCF model:
 A restricted hybrid HF-DFT SCF calculation will be
 performed using Pulay DIIS + Geometric Direct Minimization


Optimization:
        Step      Energy           Max Grad.      Max Dist.
           1   -13556.764367       0.026802       0.092144
           2   -13556.769213       0.008273       0.054314
           3   -13556.769651       0.001336       0.001260
           4   -13556.769660       0.001153       0.010465
           5   -13556.769685       0.000329       0.002076
           6   -13556.769686       0.000126       0.000620
```

Figure S69. HOMO-LUMO of **14**

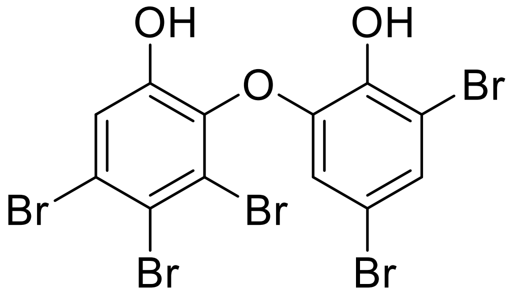

14

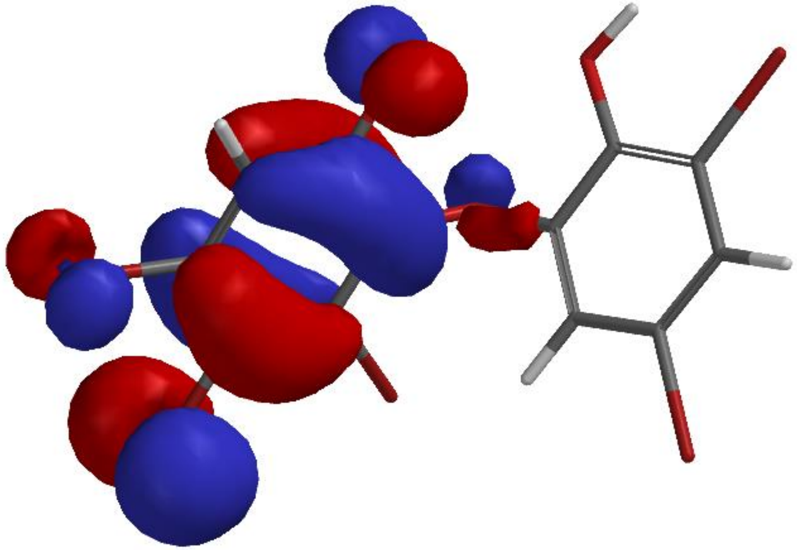

HOMO 14

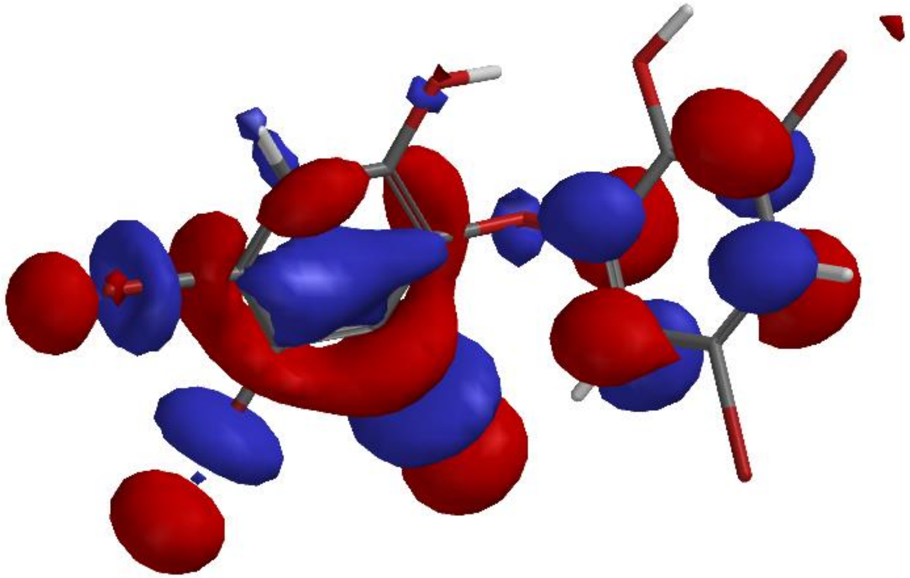

LUMO 14

Figure S70. Calculated ^{13}C NMR Chemical Shift of **14**

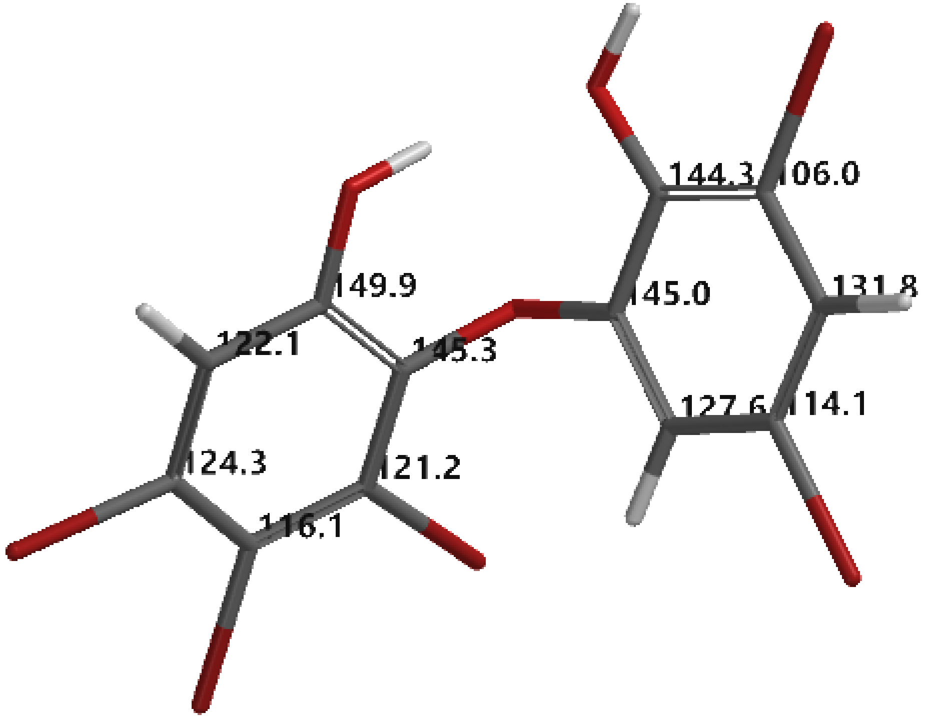

Table S39. Equilibrium Geometry DFT ωB97X-D/6-311+G(2d,p) of **15**

```
Job type: Geometry optimization.
Method: RWB97X-D
Basis set: 6-311+G(2D,P)
Number of basis functions: 675
Number of electrons: 276
Parallel Job: 16 threads

SCF model:
 A restricted hybrid HF-DFT SCF calculation will be
 performed using Pulay DIIS + Geometric Direct Minimization


Optimization:
        Step       Energy          Max Grad.       Max Dist.
           1   -13556.762016       0.021476        0.076590
           2   -13556.766688       0.007328        0.035406
           3   -13556.767190       0.001804        0.014344
           4   -13556.767172       0.001841        0.123542
           5   -13556.767366       0.001495        0.052741
           6   -13556.767419       0.001206        0.007993
           7   -13556.767443       0.000188        0.002668
           8   -13556.767443       0.000053        0.001092
```

Figure S71. HOMO-LUMO of **15**

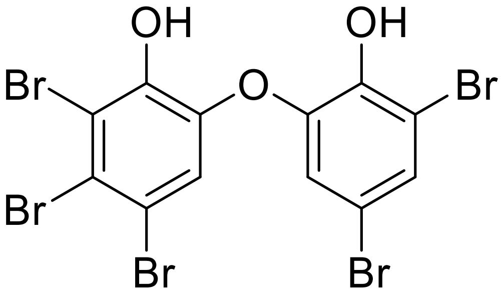

15

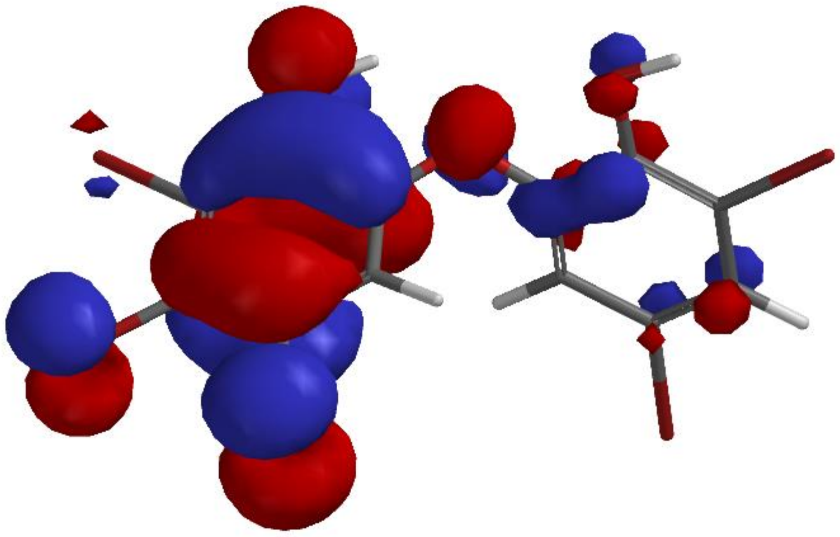

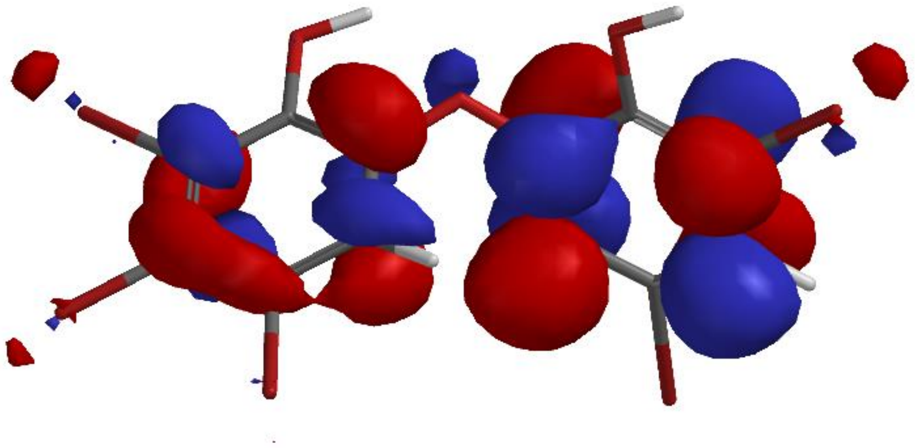

HOMO 15

LUMO 15

Figure S72. Calculated ^{13}C NMR Chemical Shift of **15**

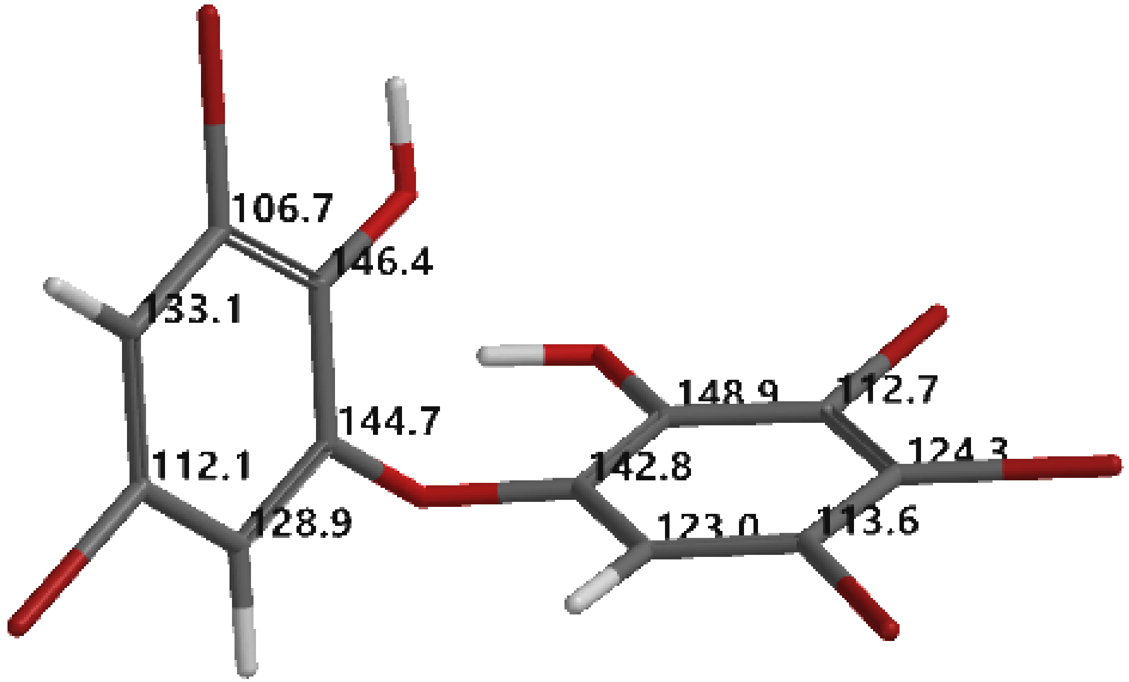

Figure S73. ^{1}H NMR spectrum of **16** (Me_2CO-d_6, 500 MHz)

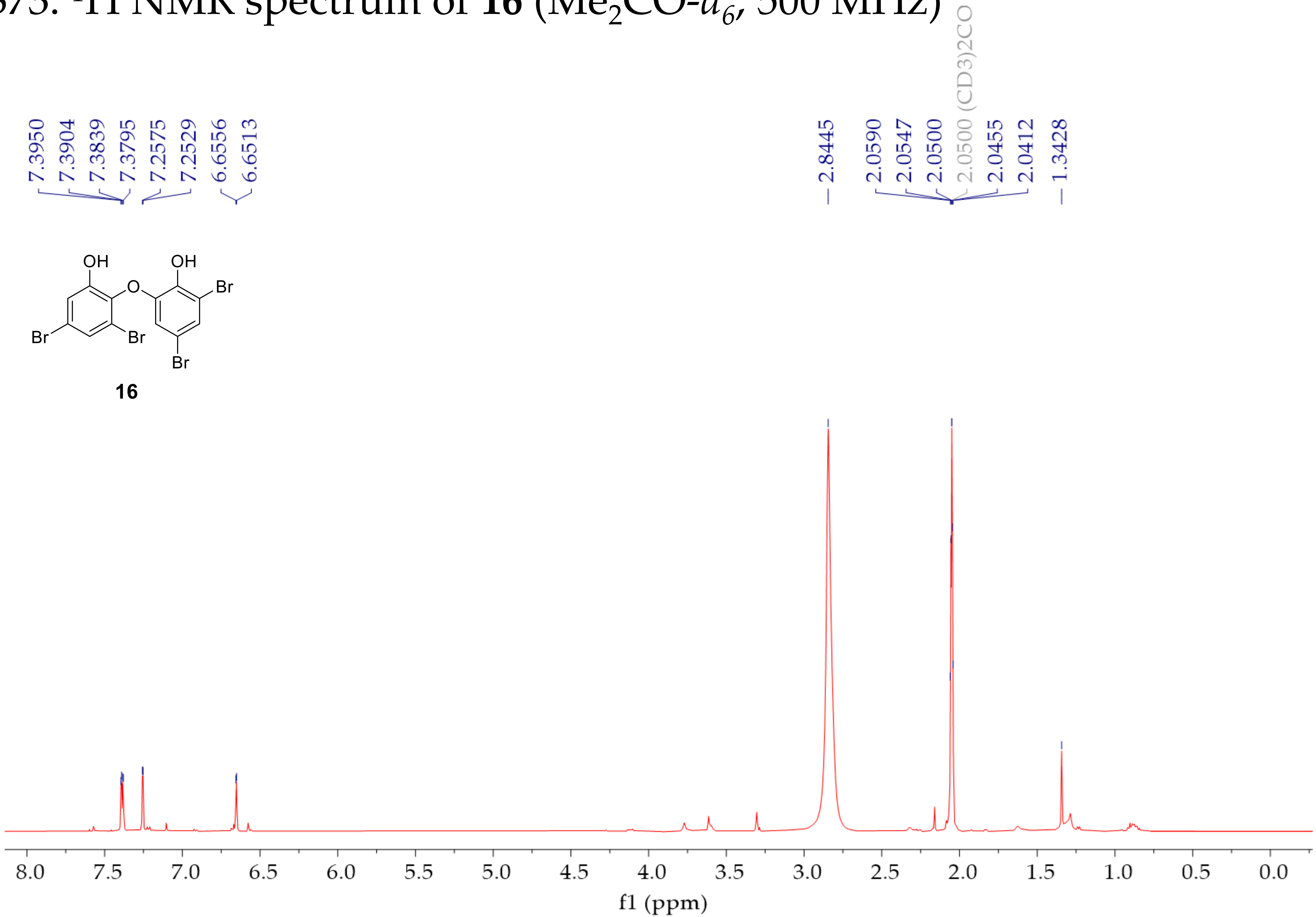

Figure S74. ^{1}H NMR spectrum of **16** (Me_2CO-d_6, 500 MHz)

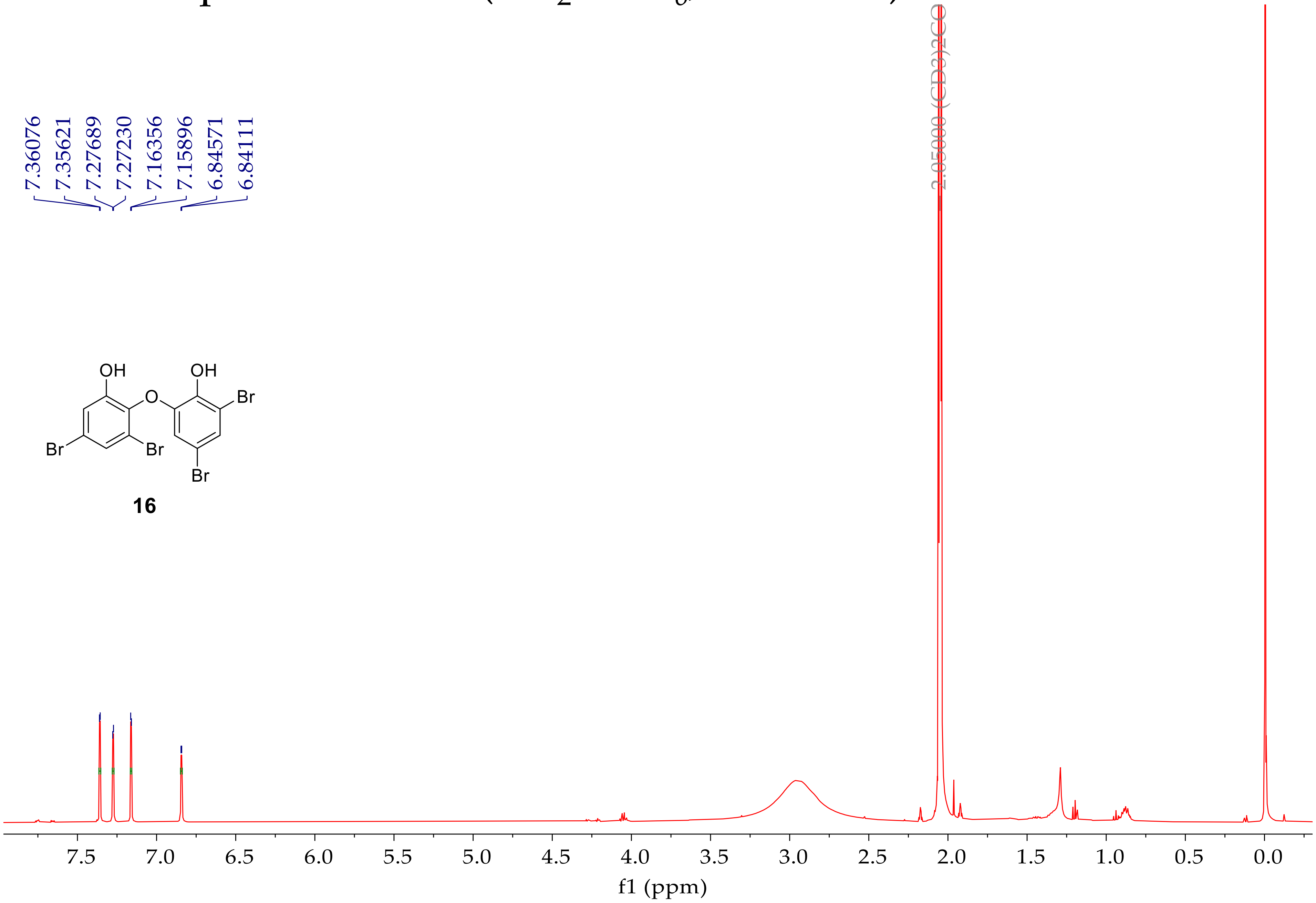

Figure S75. ^{1}H-^{1}H COSY spectrum of **16** (Me_2CO-d_6, 500 MHz)

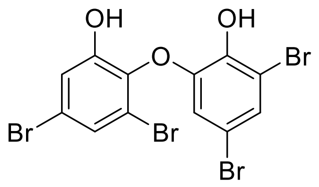

16

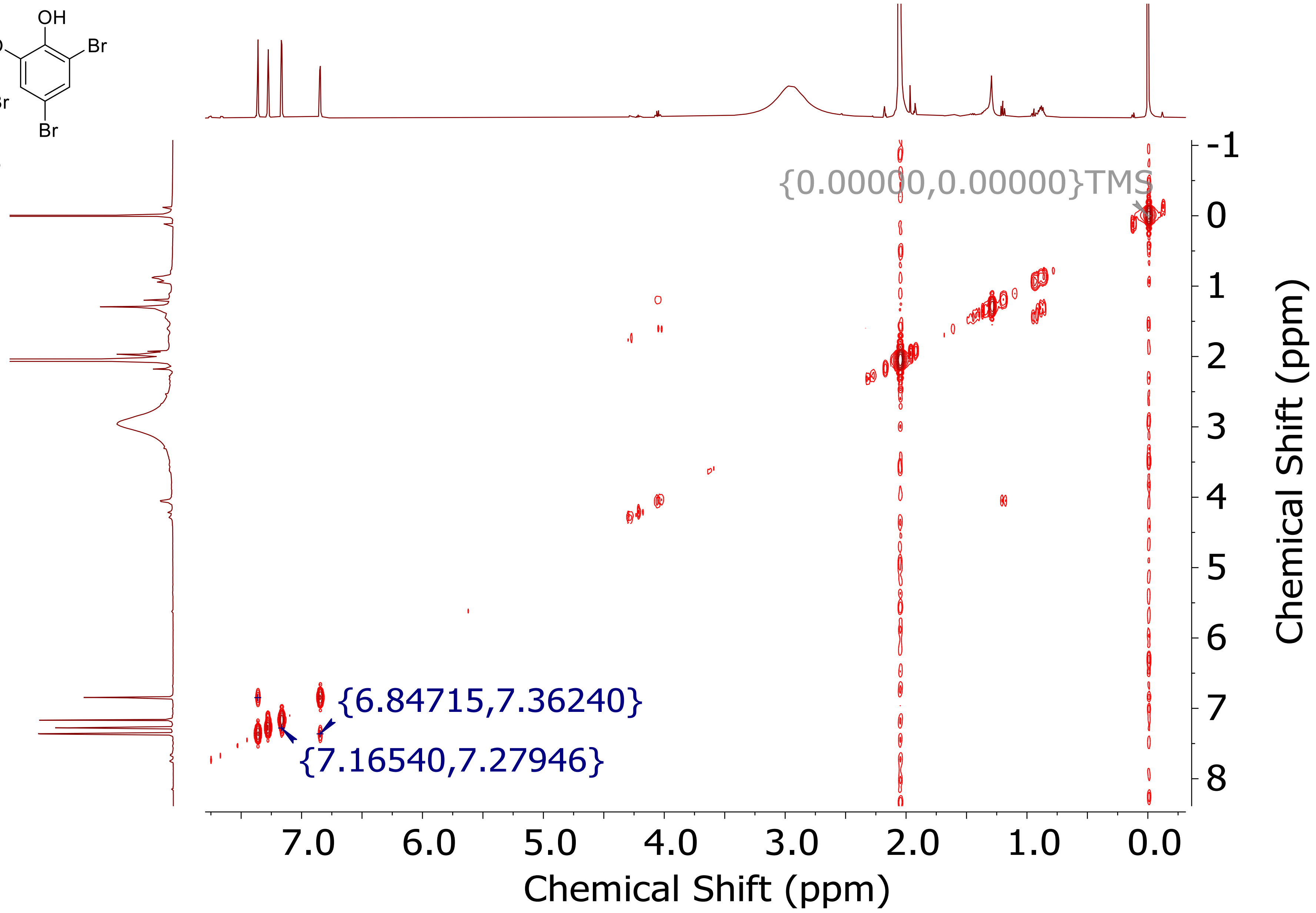

Figure S76. ^{1}H-^{13}C HSQC spectrum of **16** (Me$_2$CO-d_6, 500 MHz)

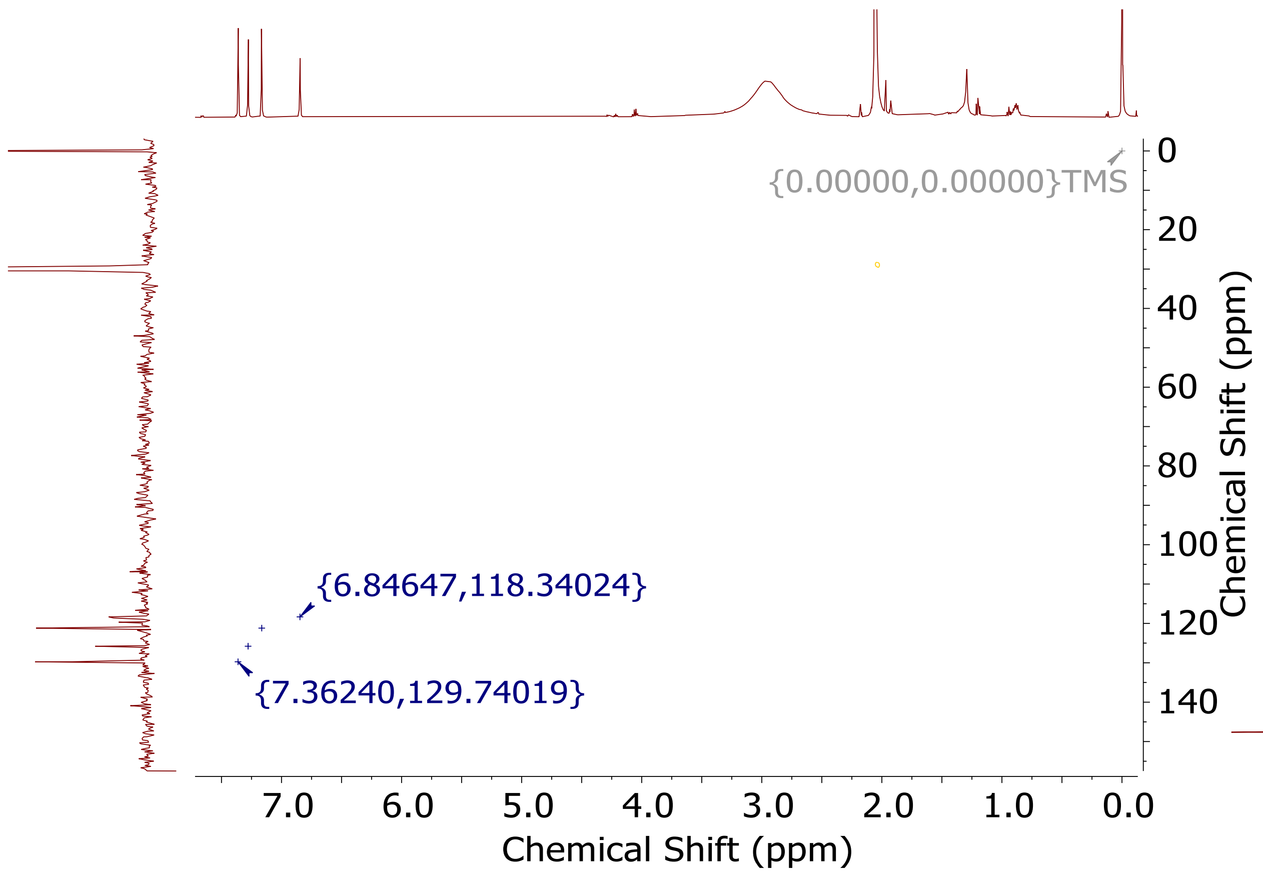

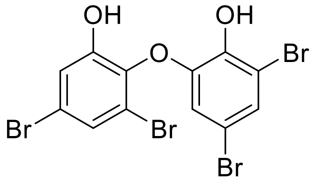

16

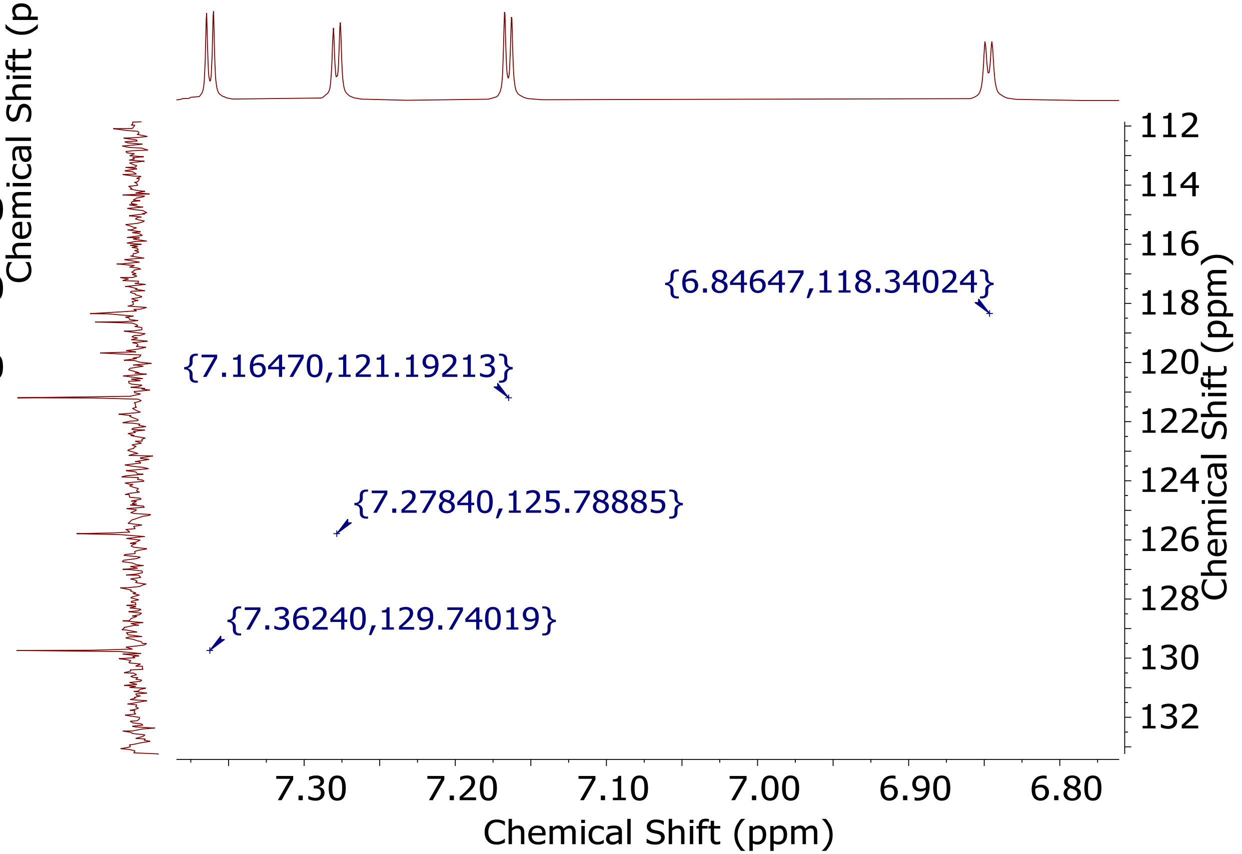

Figure S77. ^{1}H-^{13}C HMBC spectrum of **16** (Me_2CO-d_6, 500 MHz)

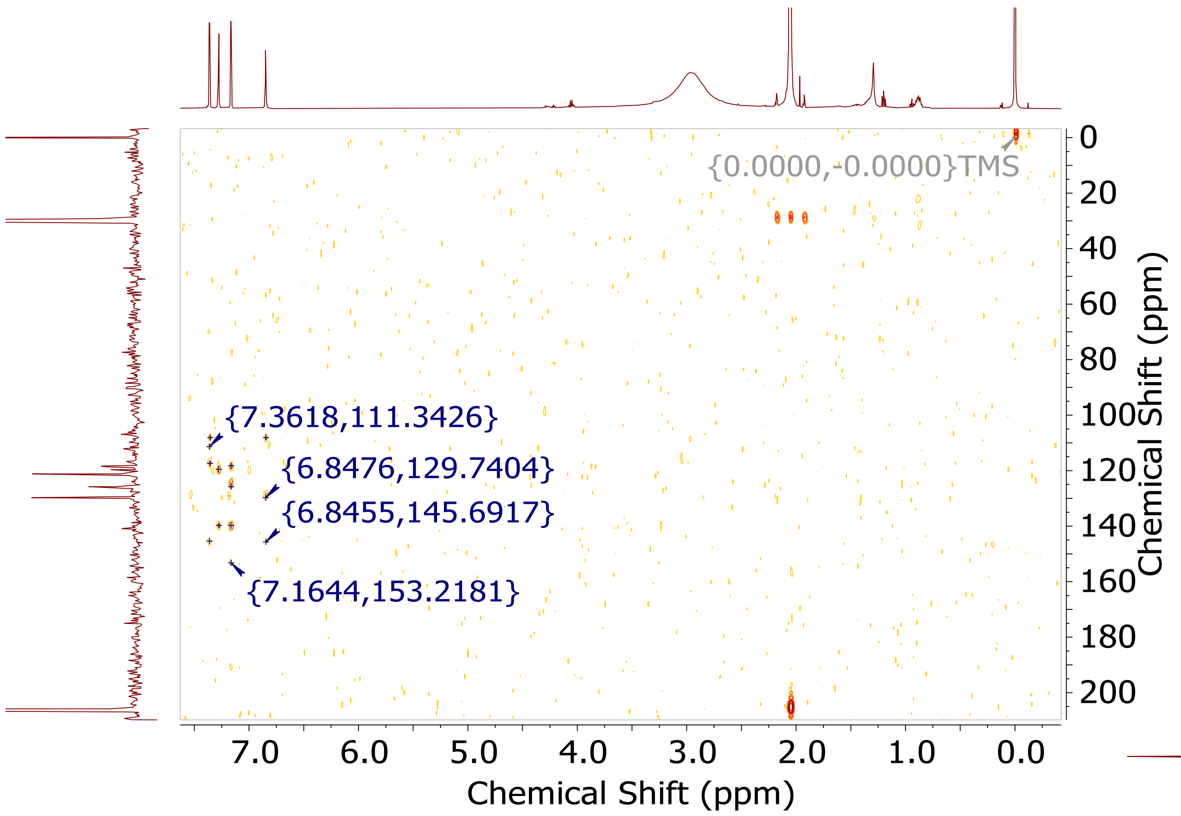

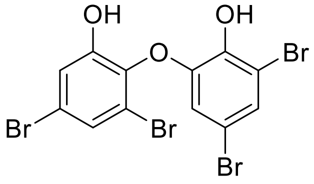

16

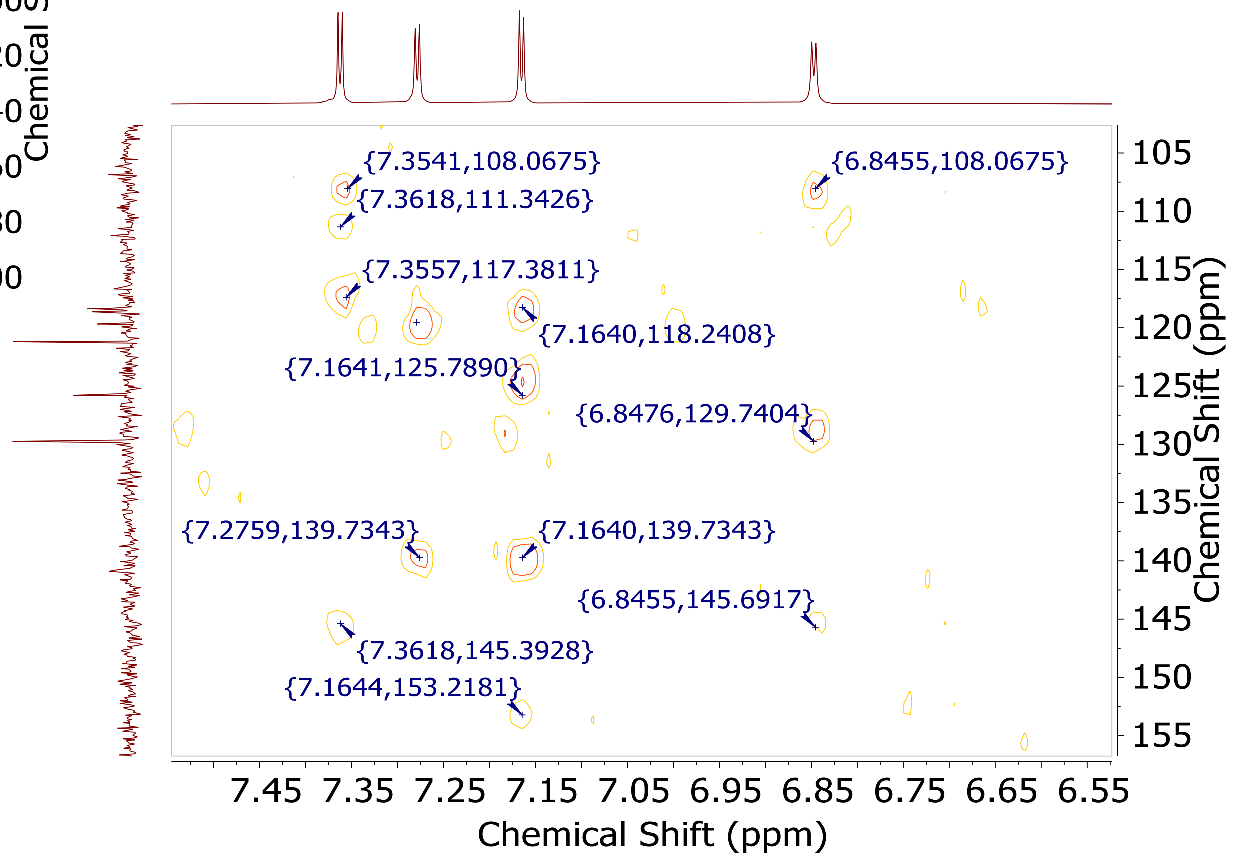

Figure S78. HREIMS of **16**

ターミナル1

SAMPLE : PBDE-192C-3
SAMPLE NO.: 772 SCAN NO.: 15

PAGE 1

NO.	PKNO.	P.C.	M/Z(OBS.)	ER(MU)	US	ELEMENTS
1	205	8.0	504.9696			
2	206	17.6	513.6962	- 8.7	8.0	C12 H6 Br4 O3
3	207	2.8	514.7133			
4	208	69.4	515.7007	- 2.2	8.0	C12 H6 Br3 Br*1 O3
5	209	10.5	516.7071	0.8	8.0	C11 C*1 H6 Br3 Br*1 O3
6	210	4.9	516.9696			
7	211	100.0	517.6907	-10.1	8.0	C12 H6 Br2 Br*2 O3
8	212	1.1	517.9964			
9	213	14.4	518.6998	- 4.4	8.0	C11 C*1 H6 Br2 Br*2 O3
10	214	59.7	519.6914	- 7.4	8.0	C12 H6 Br1 Br*3 O3
11	215	8.2	520.7143			
12	216	22.9	521.6952	- 1.6	8.0	C12 H6 Br*4 O3
13	217	1.8	522.7074			
14	218	2.2	523.9968			
15	219	1.8	528.9992			
16	220	7.5	530.9664			
17	221	1.3	537.0021			
18	222	1.8	541.0110			
19	223	1.0	542.6993			

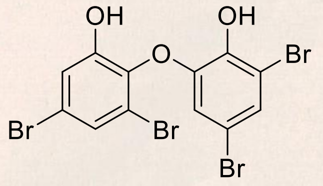

16

Table S40. Equilibrium Geometry DFT ωB97X-D/6-311+G(2d,p) of **16**

```
Job type: Geometry optimization.
Method: RWB97X-D
Basis set: 6-311+G(2D,P)
Number of basis functions: 633
Number of electrons: 242
Parallel Job: 16 threads

SCF model:
 A restricted hybrid HF-DFT SCF calculation will be
 performed using Pulay DIIS + Geometric Direct Minimization


Optimization:
         Step       Energy          Max Grad.      Max Dist.
            1   -10983.204306       0.019514       0.105574
            2   -10983.208599       0.006637       0.048535
            3   -10983.208980       0.001289       0.001567
            4   -10983.208992       0.001103       0.009585
            5   -10983.209019       0.000365       0.001983
            6   -10983.209019       0.000137       0.000376
```

Figure S79. HOMO-LUMO of **16**

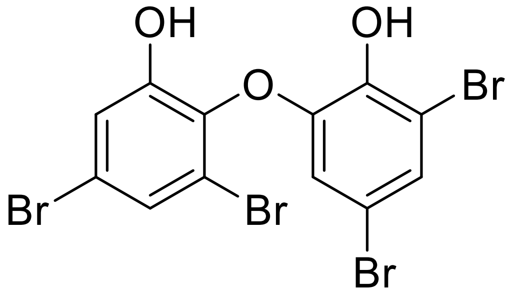

16

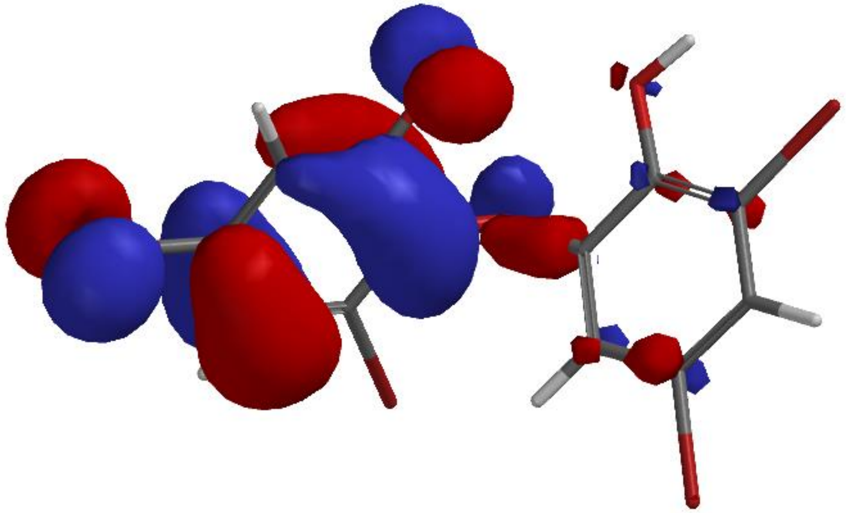

HOMO 16

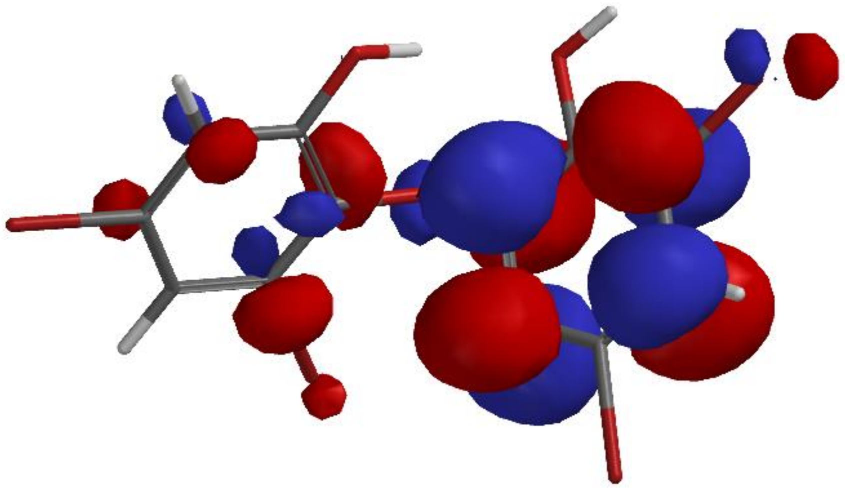

LUMO 16

Figure S80. Calculated ^{13}C NMR Chemical Shift of **16**

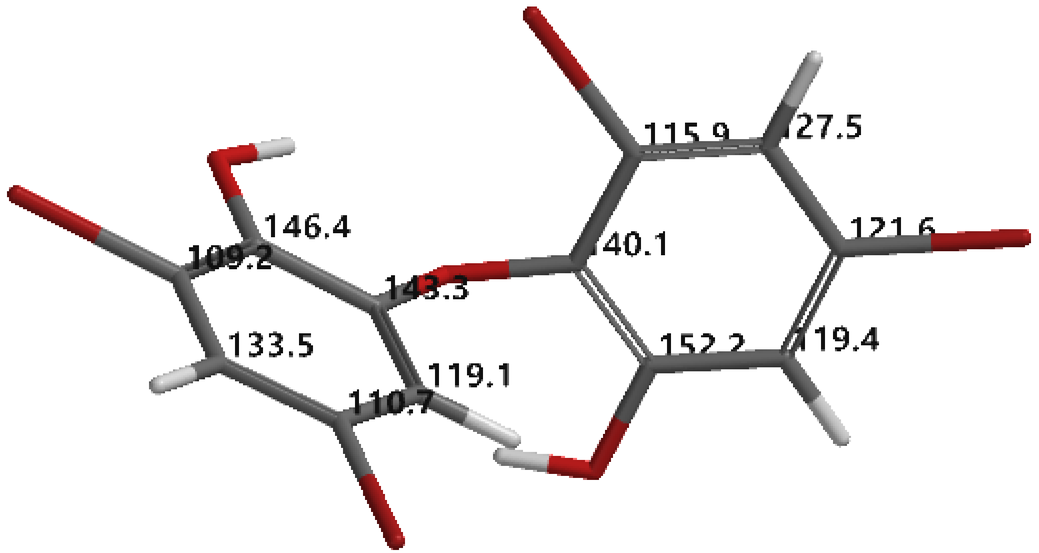

Figure S81. ^1H NMR spectrum of **17** (Me_2CO-d_6, 500 MHz)

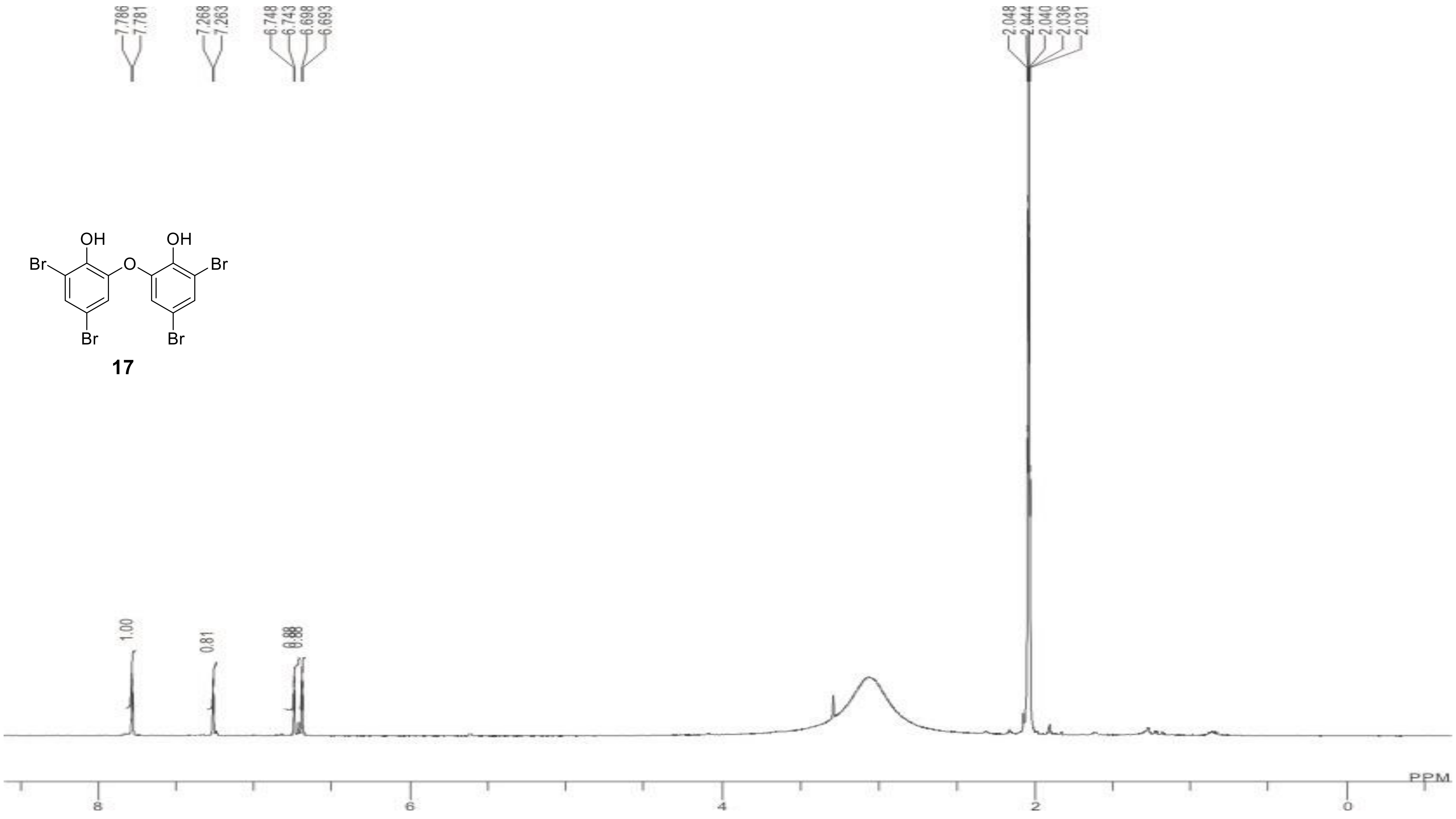

Figure S82. LREIMS of **17**

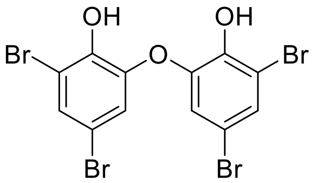

17

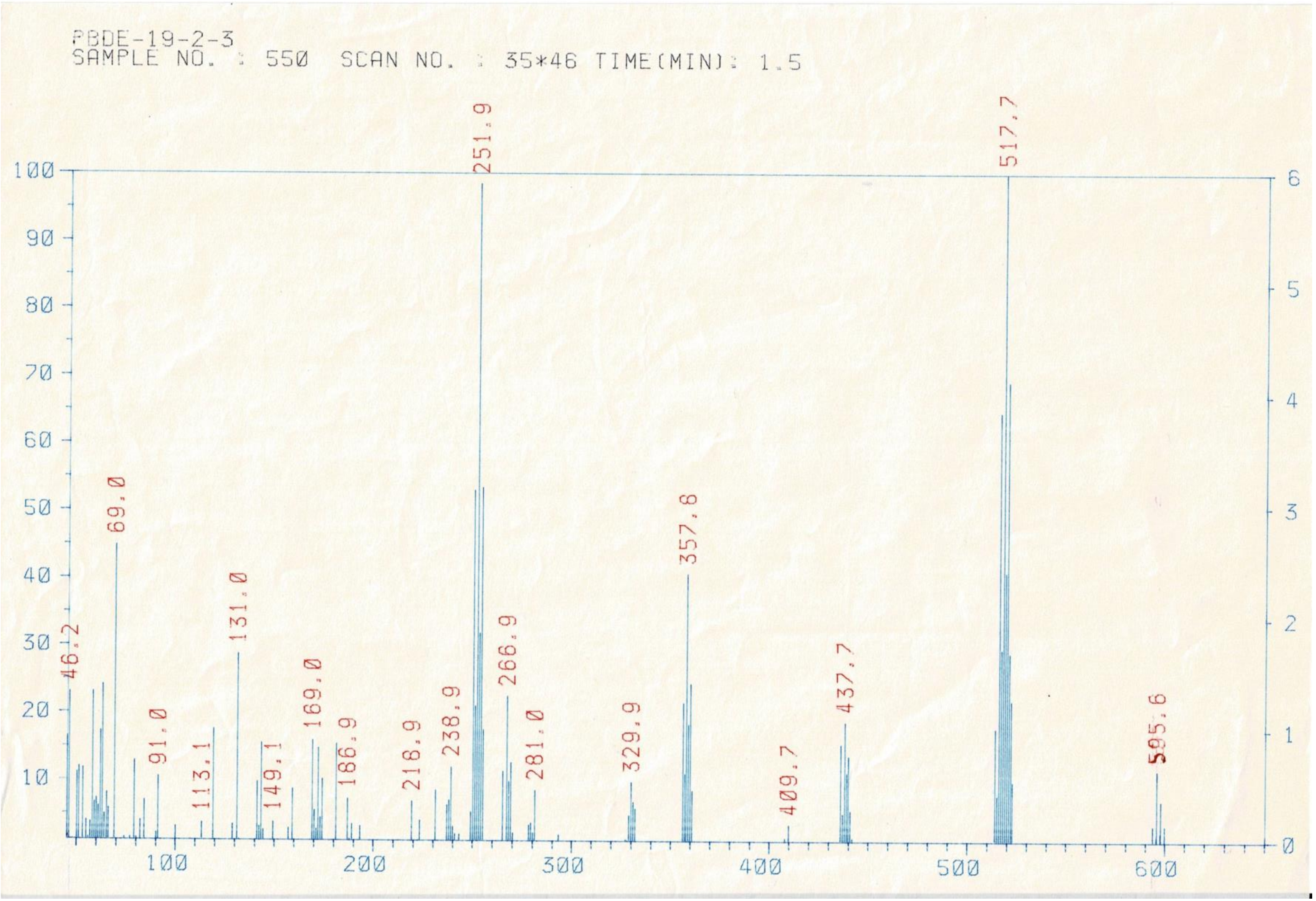

Table S41. Equilibrium Geometry DFT ωB97X-D/6-311+G(2d,p) of **17**

```
Job type: Geometry optimization.
Method: RWB97X-D
Basis set: 6-311+G(2D,P)
Number of basis functions: 633
Number of electrons: 242
Parallel Job: 16 threads

SCF model:
 A restricted hybrid HF-DFT SCF calculation will be
 performed using Pulay DIIS + Geometric Direct Minimization


Optimization:
         Step       Energy           Max Grad.       Max Dist.
            1   -10983.201771       0.019121        0.075432
            2   -10983.205709       0.007524        0.038952
            3   -10983.206070       0.001657        0.012114
            4   -10983.206114       0.001665        0.159896
            5   -10983.206307       0.002395        0.019355
            6   -10983.206382       0.001516        0.019983
            7   -10983.206418       0.000385        0.010043
            8   -10983.206423       0.000390        0.030467
```

Figure S83. HOMO-LUMO of **17**

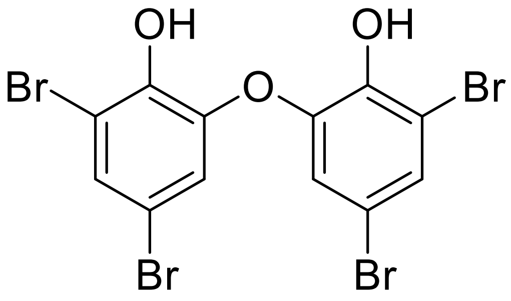

17

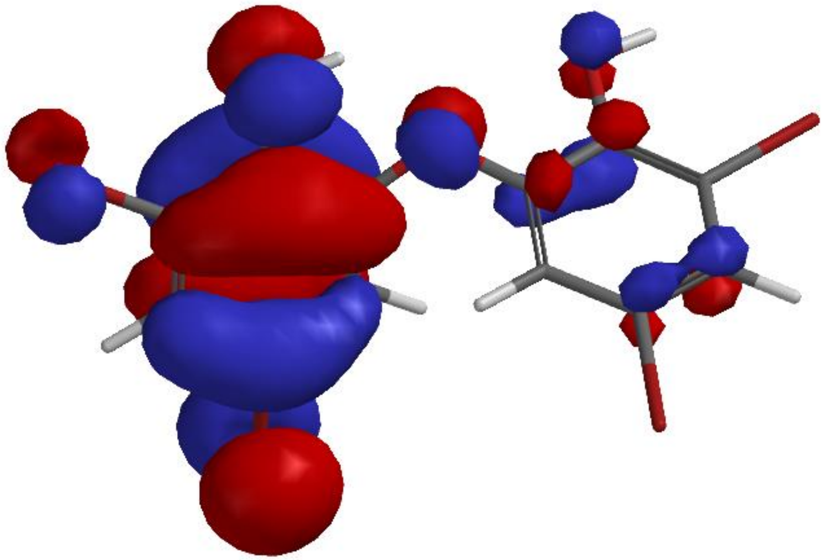

HOMO 17

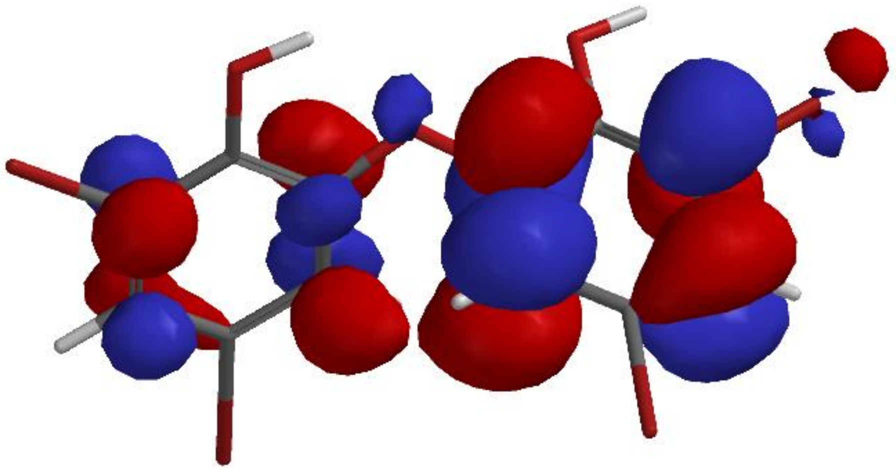

LUMO 17

Figure S84. Calculated ^{13}C NMR Chemical Shift of **17**

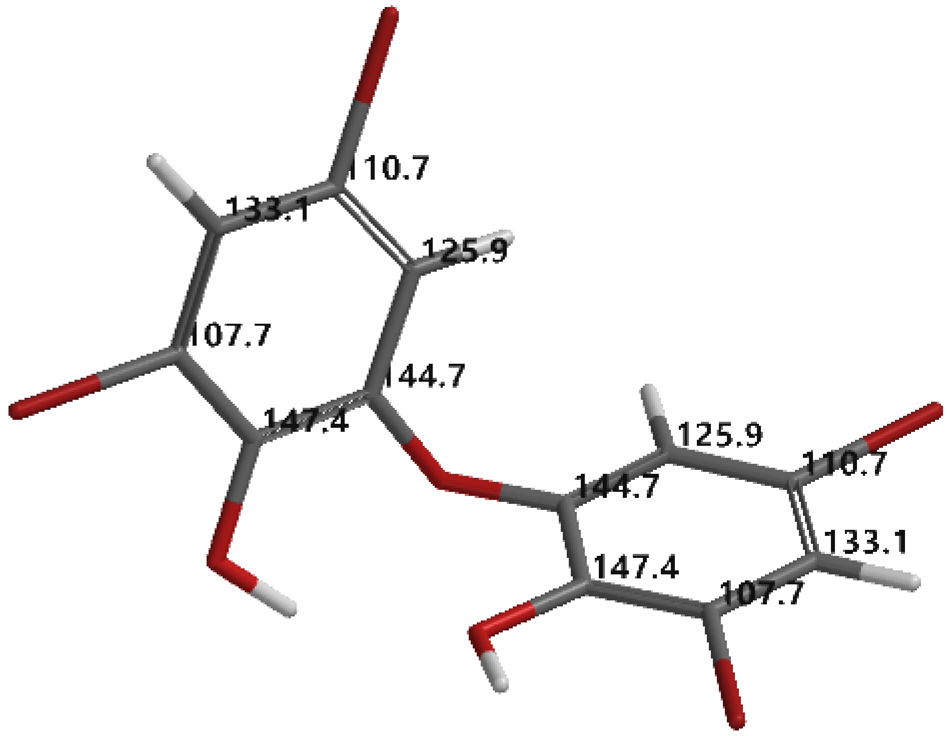

Figure S85. ^1H NMR spectrum of **18** (Me_2CO-d_6, 500 MHz)

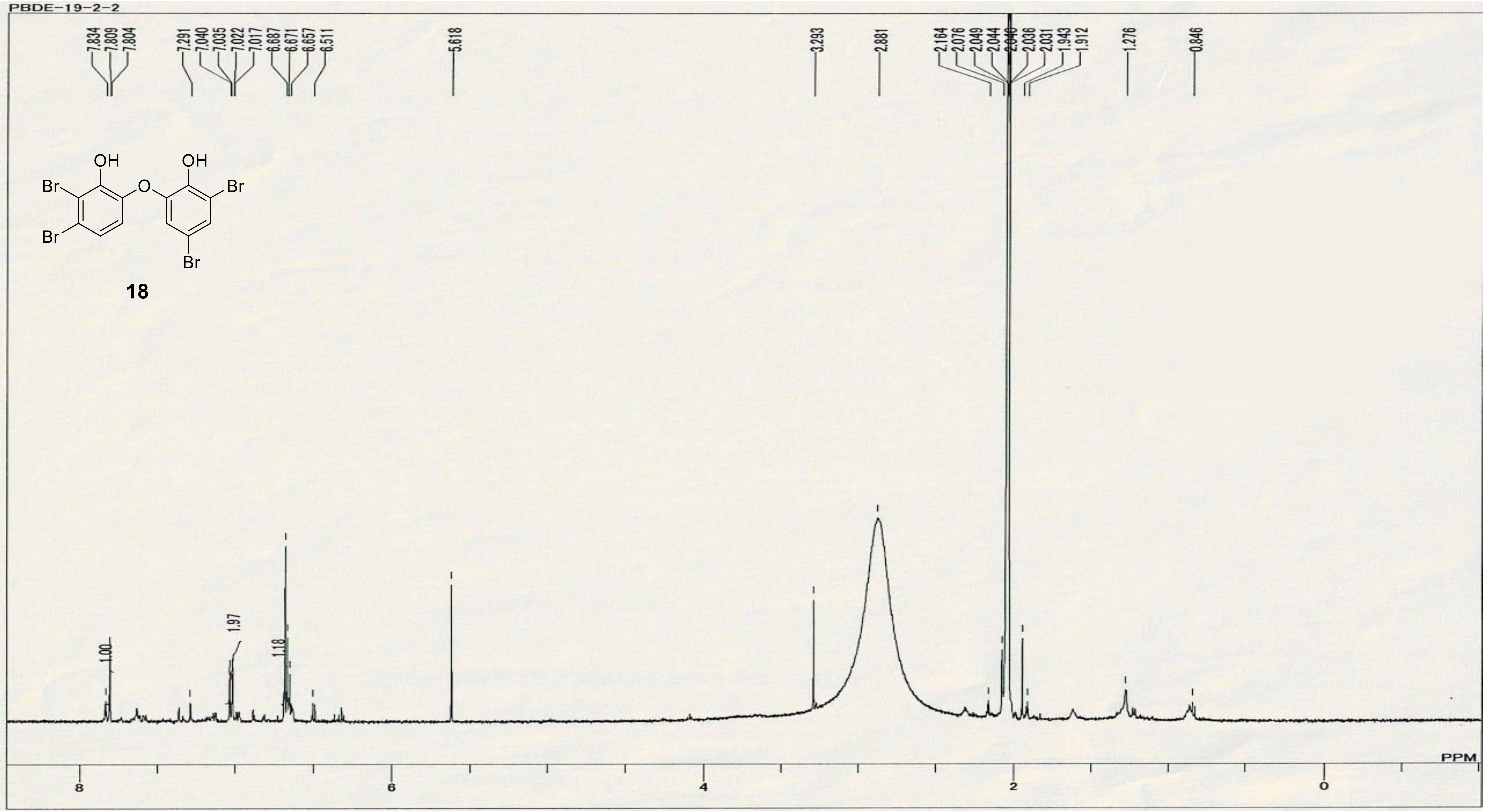

Figure S86. LREIMS of **18**

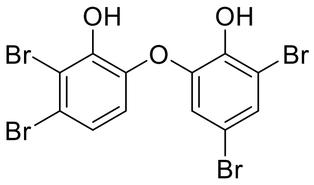

18

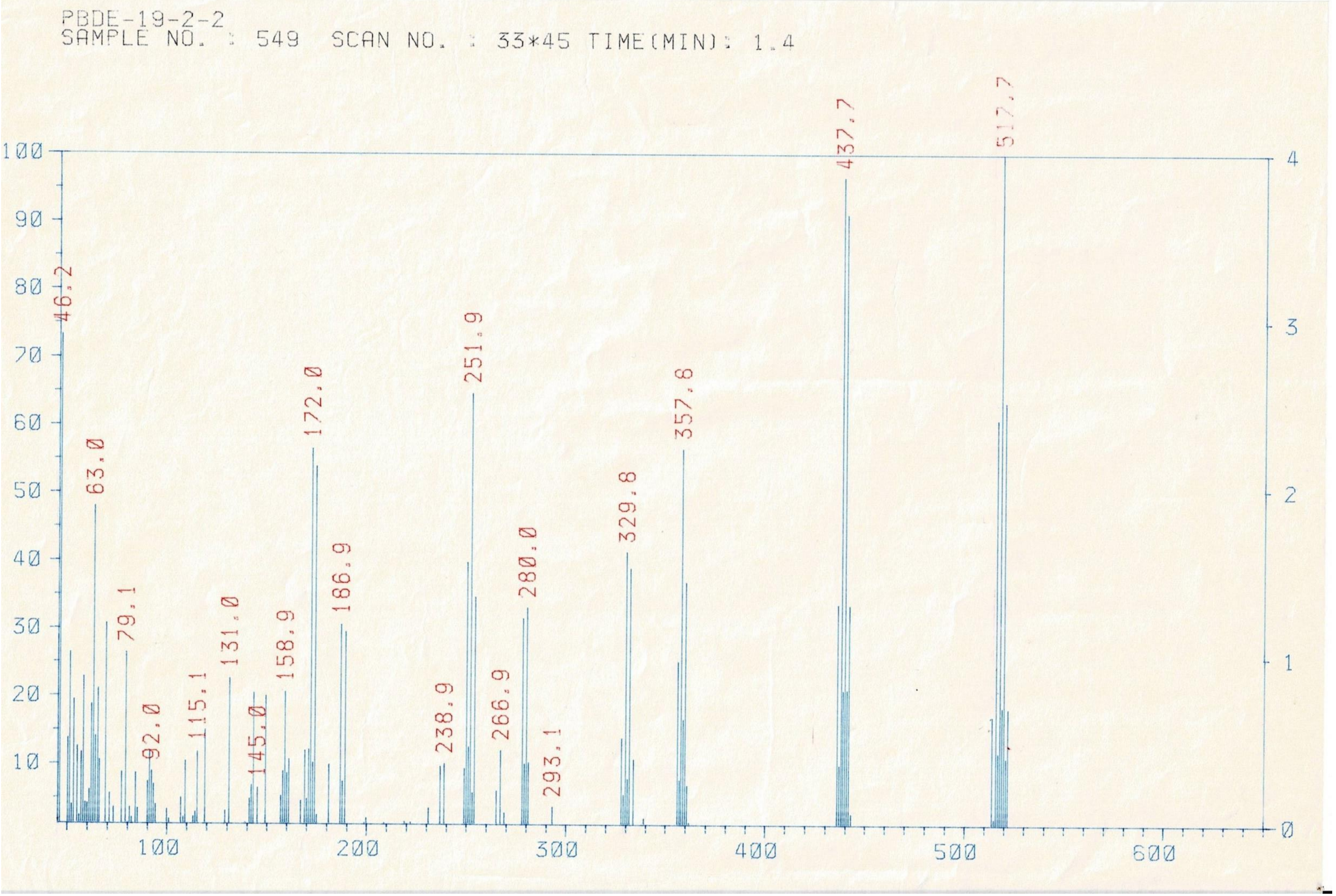

Table S42. Equilibrium Geometry DFT ωB97X-D/6-311+G(2d,p) of **18**

```
Job type: Geometry optimization.
Method: RWB97X-D
Basis set: 6-311+G(2D,P)
Number of basis functions: 633
Number of electrons: 242
Parallel Job: 16 threads

SCF model:
 A restricted hybrid HF-DFT SCF calculation will be
 performed using Pulay DIIS + Geometric Direct Minimization


Optimization:
        Step      Energy          Max Grad.      Max Dist.
           1   -10983.197652       0.019940       0.073170
           2   -10983.202008       0.007085       0.029451
           3   -10983.202442       0.001721       0.012466
           4   -10983.202490       0.001711       0.135443
           5   -10983.202707       0.001536       0.016656
           6   -10983.202752       0.000794       0.024183
           7   -10983.202769       0.000340       0.018511
```

Figure S87. HOMO-LUMO of **18**

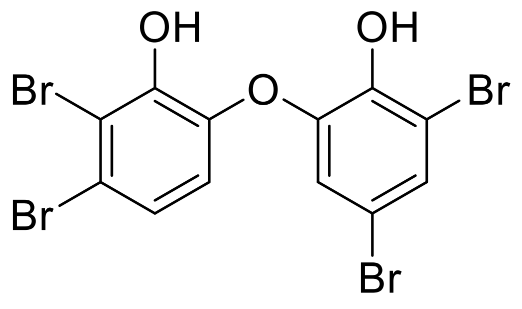

18

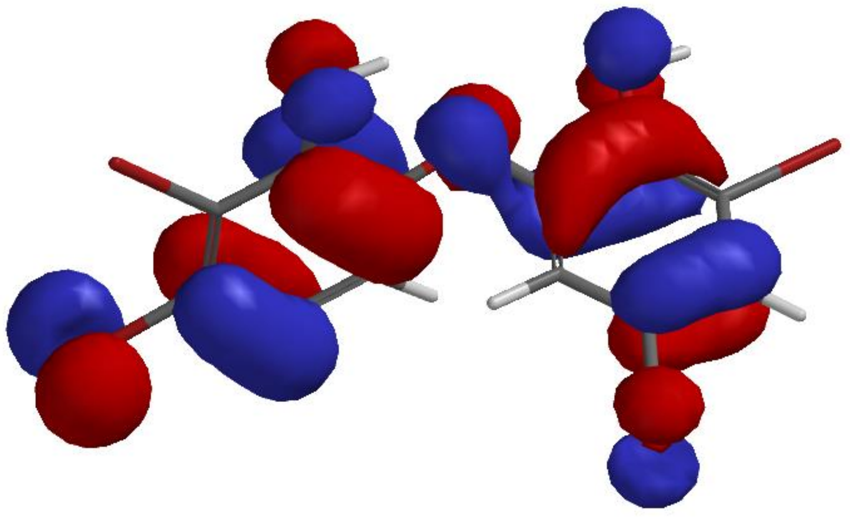

HOMO 18

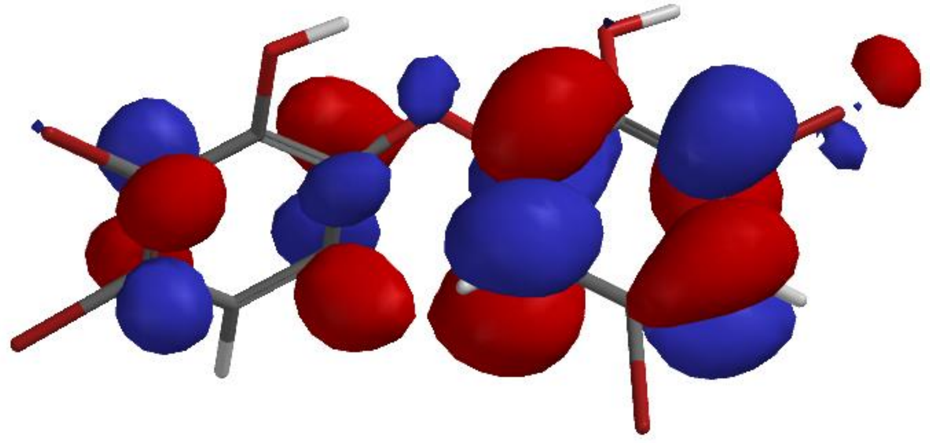

LUMO 18

Figure S88. Calculated ^{13}C NMR Chemical Shift of **18**

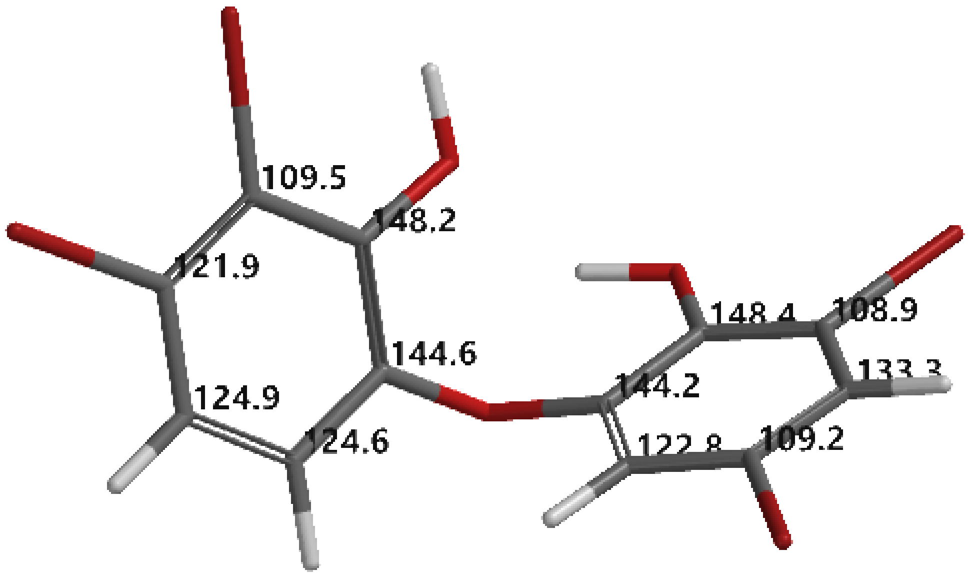

Table S43. Equilibrium Geometry DFT ωB97X-D/6-311+G(2d,p) of **19**

```
Job type: Geometry optimization.
Method: RWB97X-D
Basis set: 6-311+G(2D,P)
Number of basis functions: 633
Number of electrons: 242
Parallel Job: 16 threads

SCF model:
 A restricted hybrid HF-DFT SCF calculation will be
 performed using Pulay DIIS + Geometric Direct Minimization


Optimization:
        Step       Energy           Max Grad.      Max Dist.
           1   -10983.200602        0.016693       0.073750
           2   -10983.204147        0.006753       0.031396
           3   -10983.204508        0.001359       0.003948
           4   -10983.204523        0.001317       0.090499
           5   -10983.204695        0.001149       0.027379
           6   -10983.204725        0.000761       0.027626
           7   -10983.204743        0.000361       0.013578
```

Figure S89. HOMO-LUMO of **19**

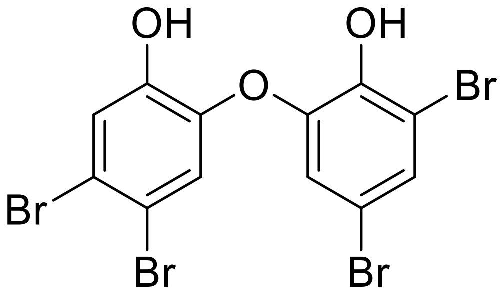

19

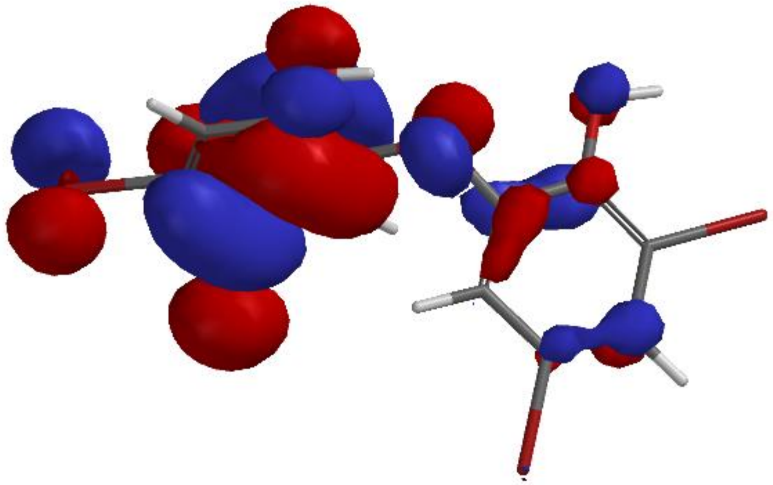

HOMO 19

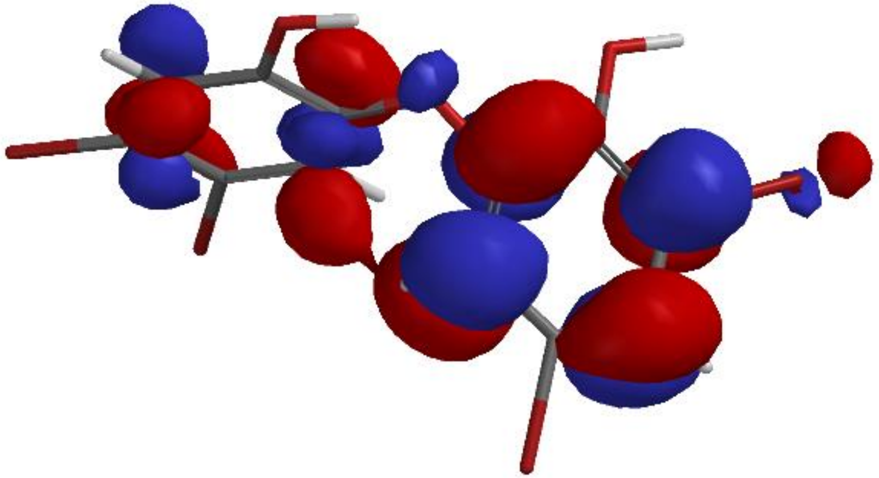

LUMO 19

Figure S90. Calculated ^{13}C NMR Chemical Shift of **19**

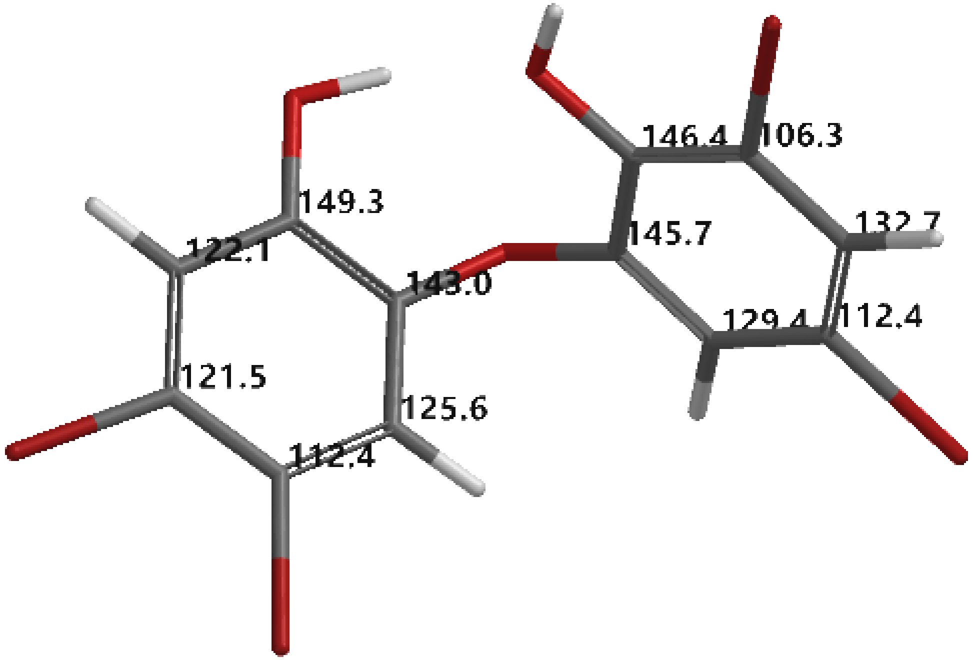

Figure S91. ^{1}H NMR spectrum of **20** (Me_2CO-d_6, 500 MHz)

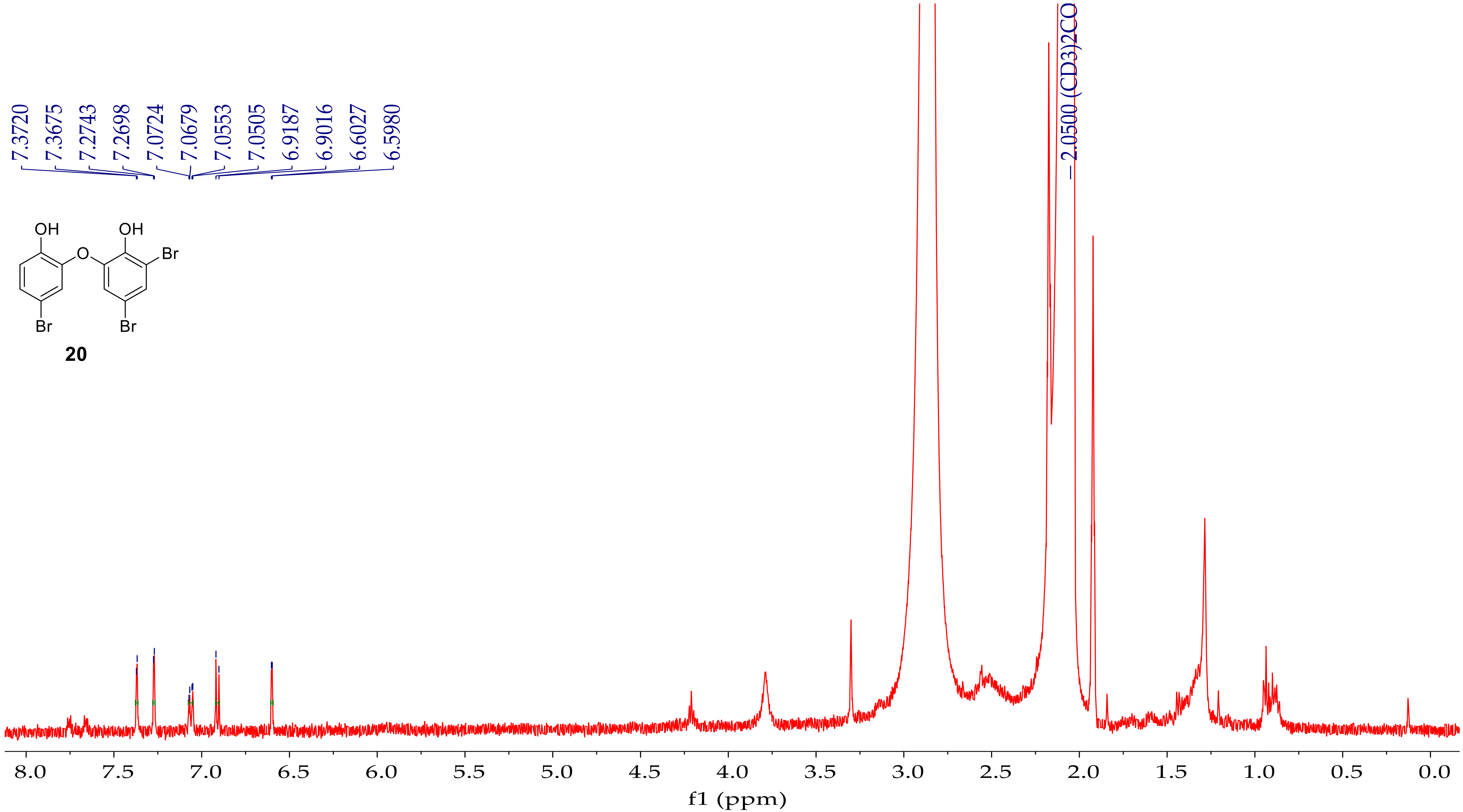

Figure S92. ^{1}H NMR spectrum of **20** (CD_3OD, 500 MHz)

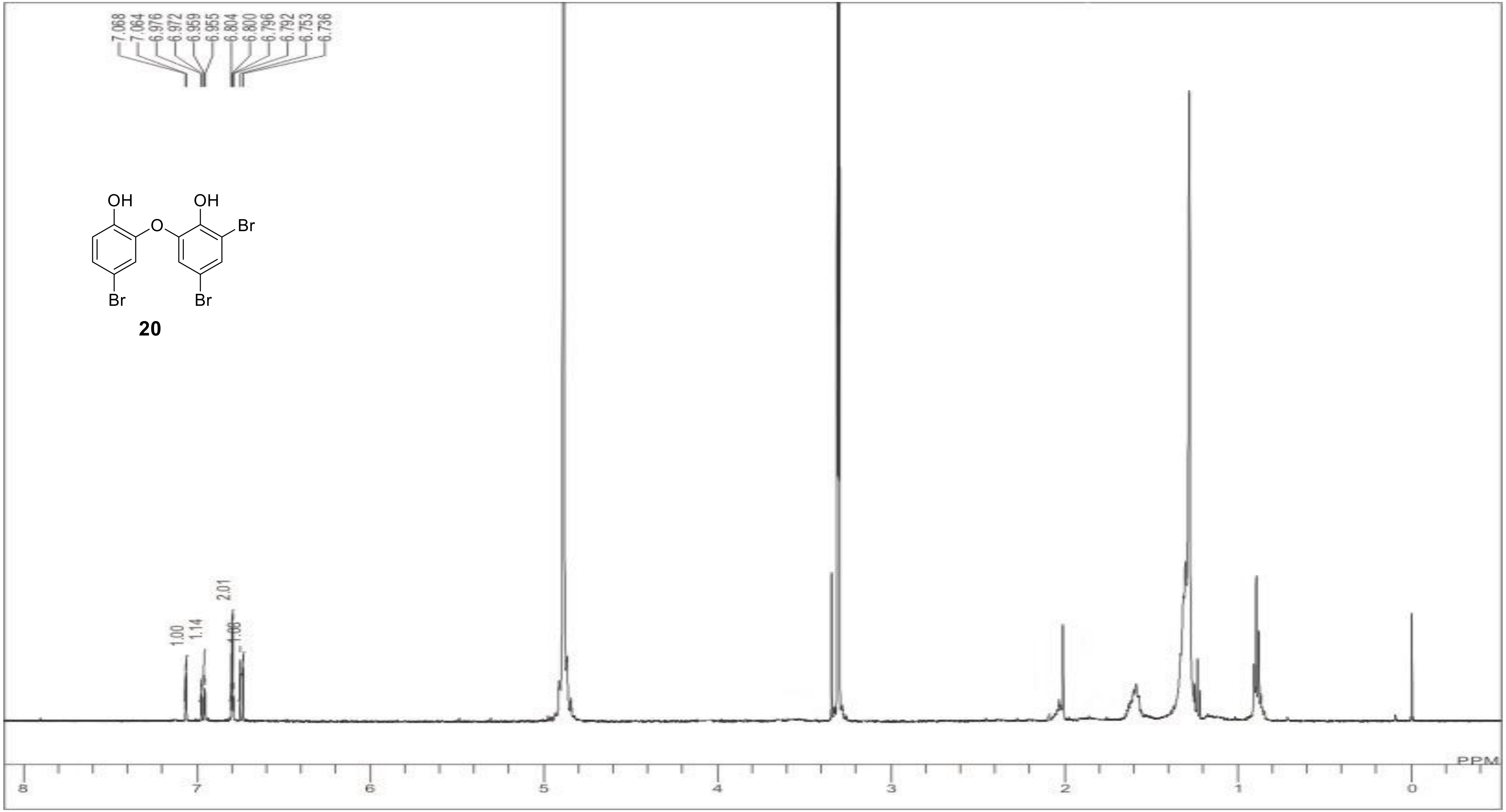

Figure S93. HRESIMS of **20**

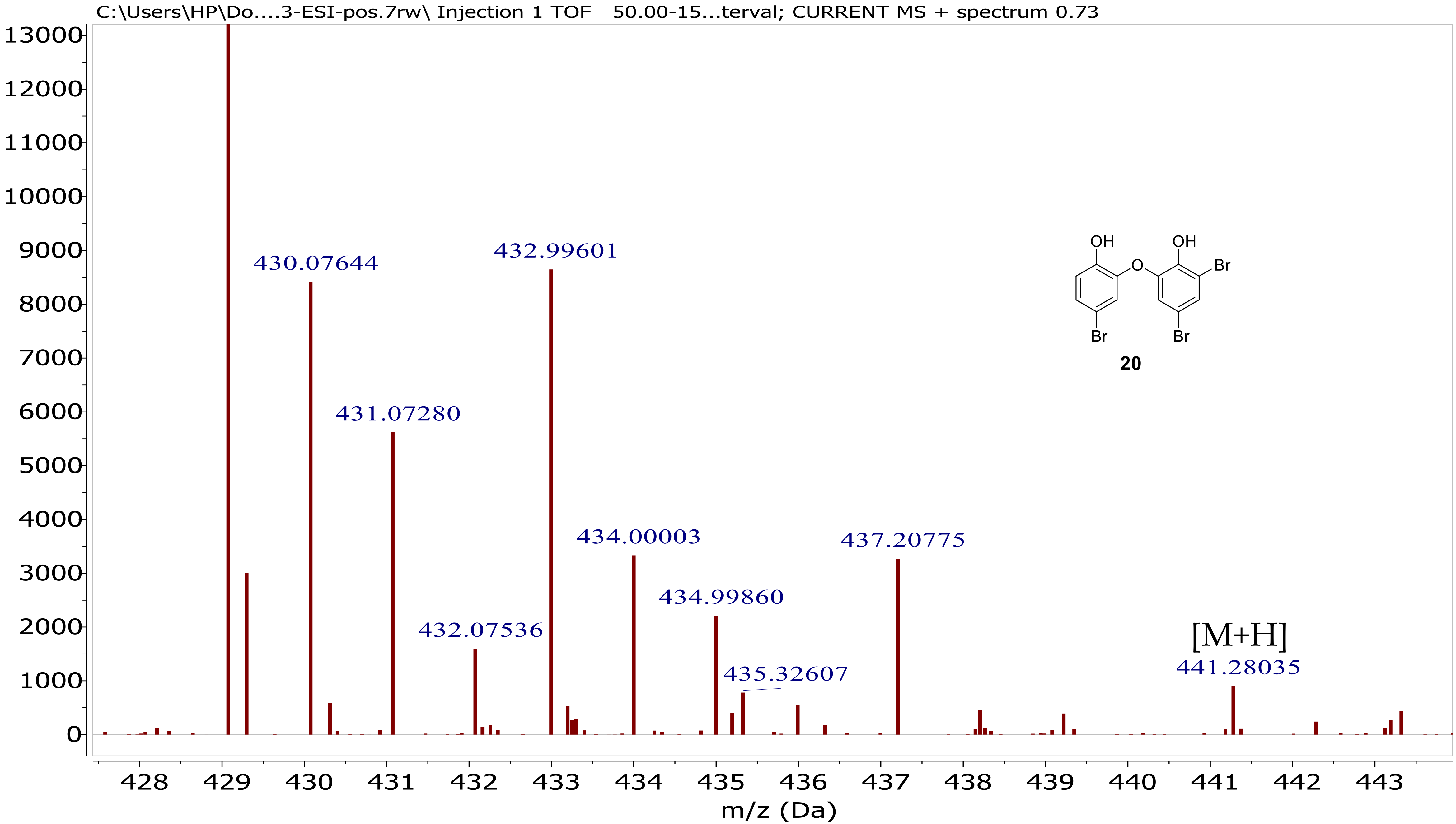

Table S44. Equilibrium Geometry DFT ωB97X-D/6-311+G(2d,p) of **20**

```
Job type: Geometry optimization.
Method: RWB97X-D
Basis set: 6-311+G(2D,P)
Number of basis functions: 591
Number of electrons: 208
Parallel Job: 16 threads

SCF model:
 A restricted hybrid HF-DFT SCF calculation will be
 performed using Pulay DIIS + Geometric Direct Minimization


Optimization:
         Step      Energy          Max Grad.      Max Dist.
            1    -8409.634220       0.017535       0.085287
            2    -8409.637712       0.006305       0.030190
            3    -8409.638038       0.001863       0.014808
            4    -8409.638082       0.001851       0.137845
            5    -8409.638268       0.001677       0.035937
            6    -8409.638312       0.001098       0.028919
            7    -8409.638336       0.000524       0.018643
            8    -8409.638346       0.000611       0.120349
```

Figure S94. HOMO-LUMO of **20**

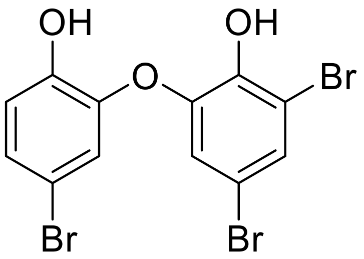

20

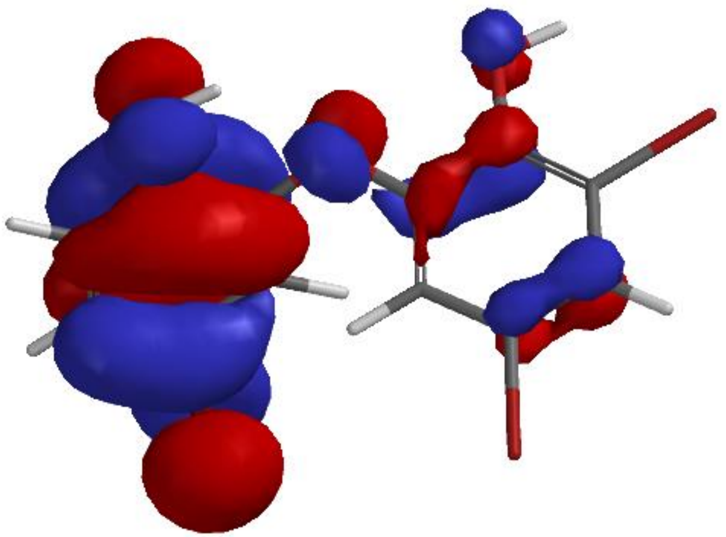

HOMO 20

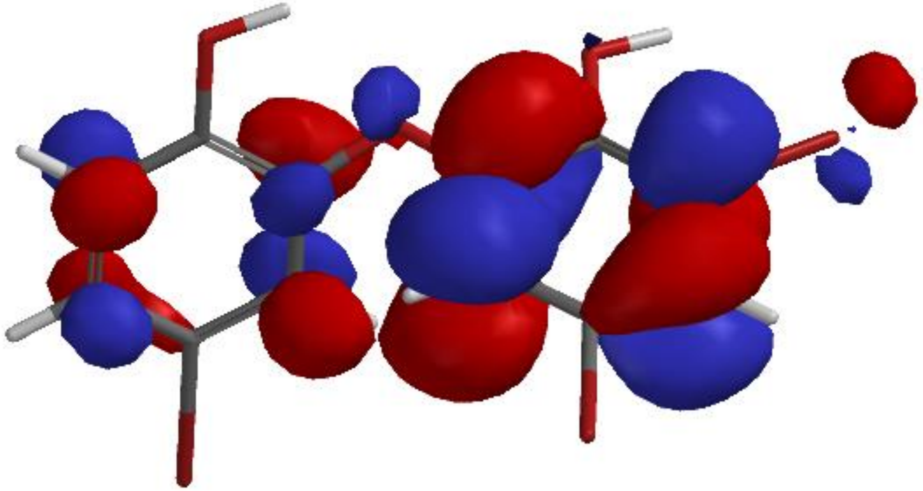

LUMO 20

Figure S95. Calculated ^{13}C NMR Chemical Shift of **20**

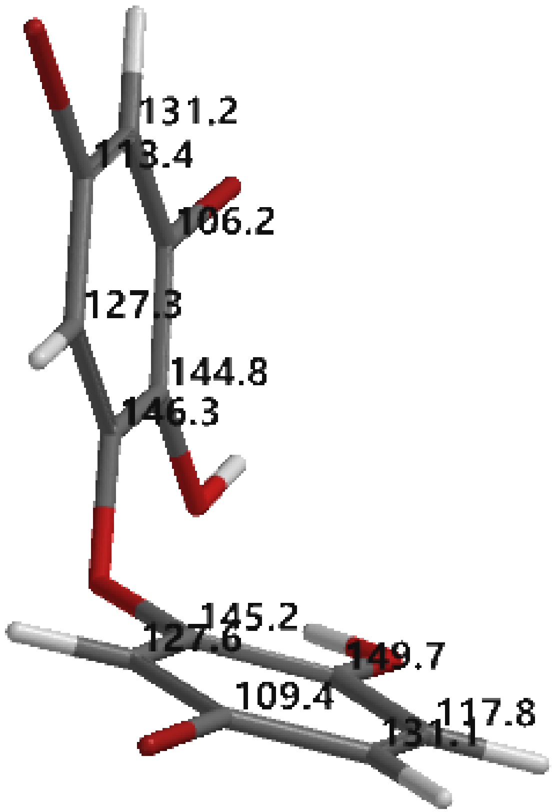

Table S45. Equilibrium Geometry DFT ωB97X-D/6-311+G(2d,p) of **21**

```
Job type: Geometry optimization.
Method: RWB97X-D
Basis set: 6-311+G(2D,P)
Number of basis functions: 591
Number of electrons: 208
Parallel Job: 16 threads

SCF model:
 A restricted hybrid HF-DFT SCF calculation will be
 performed using Pulay DIIS + Geometric Direct Minimization


Optimization:
         Step      Energy          Max Grad.      Max Dist.
            1    -8409.632240      0.017496       0.095000
            2    -8409.636292      0.005800       0.091007
            3    -8409.636732      0.001794       0.005237
            4    -8409.636767      0.000924       0.007042
            5    -8409.636781      0.000384       0.003723
```

Figure S96. HOMO-LUMO of **21**

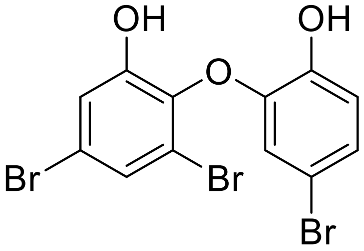

21

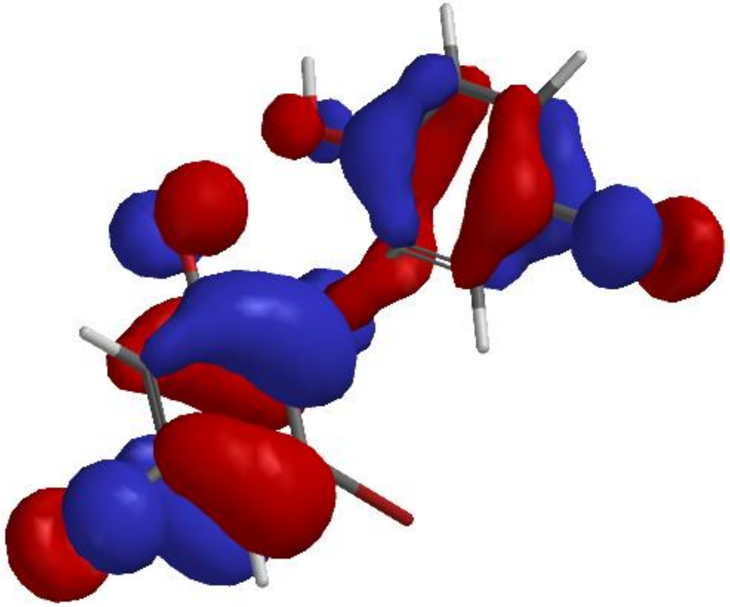

HOMO 21

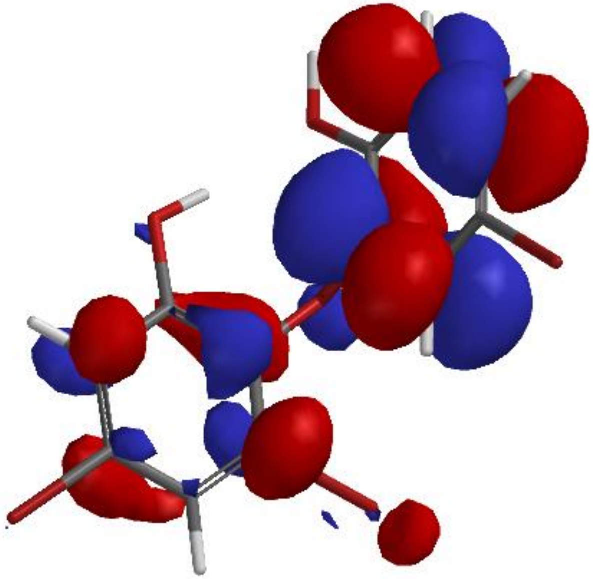

LUMO 21

Figure S97. Calculated ^{13}C NMR Chemical Shift of **21**

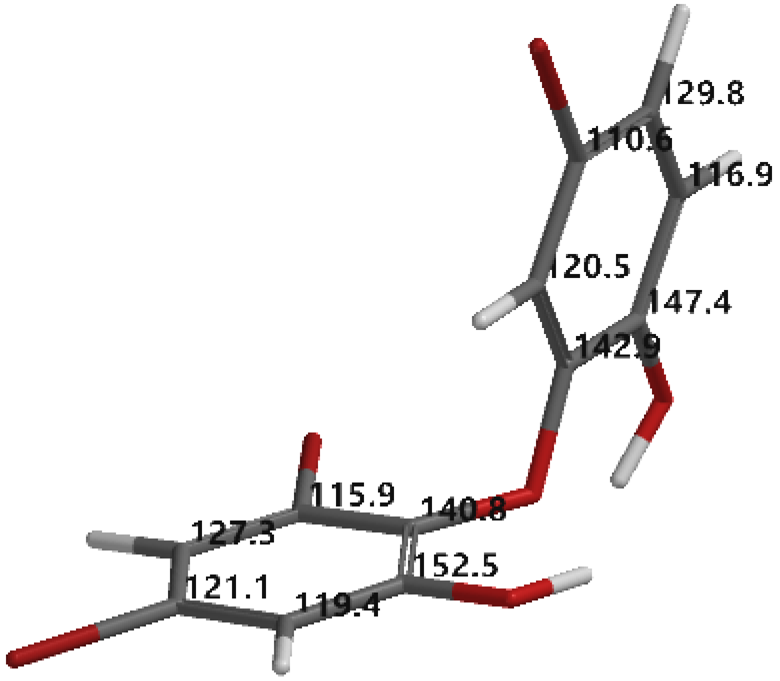

Table S46. Equilibrium Geometry DFT ωB97X-D/6-311+G(2d,p) of **22**

```
Job type: Geometry optimization.
Method: RWB97X-D
Basis set: 6-311+G(2D,P)
Number of basis functions: 591
Number of electrons: 208
Parallel Job: 16 threads

SCF model:
 A restricted hybrid HF-DFT SCF calculation will be
 performed using Pulay DIIS + Geometric Direct Minimization


Optimization:
         Step       Energy          Max Grad.      Max Dist.
            1    -8409.634727       0.015772       0.105842
            2    -8409.639110       0.006119       0.052560
            3    -8409.639489       0.001724       0.004920
            4    -8409.639516       0.000940       0.005607
            5    -8409.639524       0.000223       0.002318
```

Figure S98. HOMO-LUMO of **22**

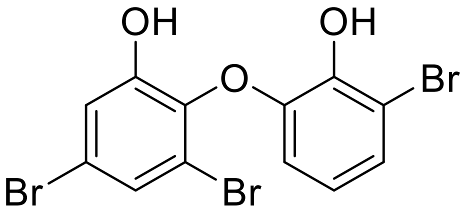

22

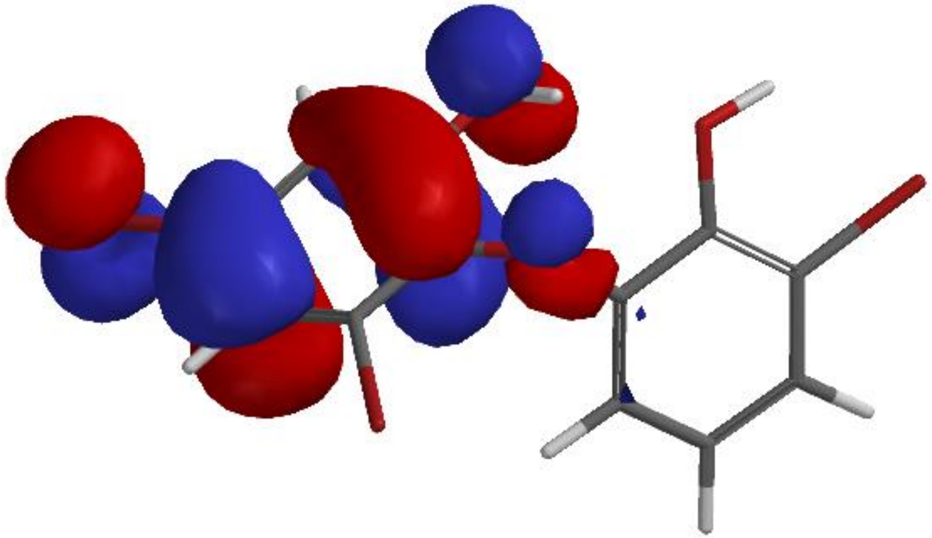

HOMO 22

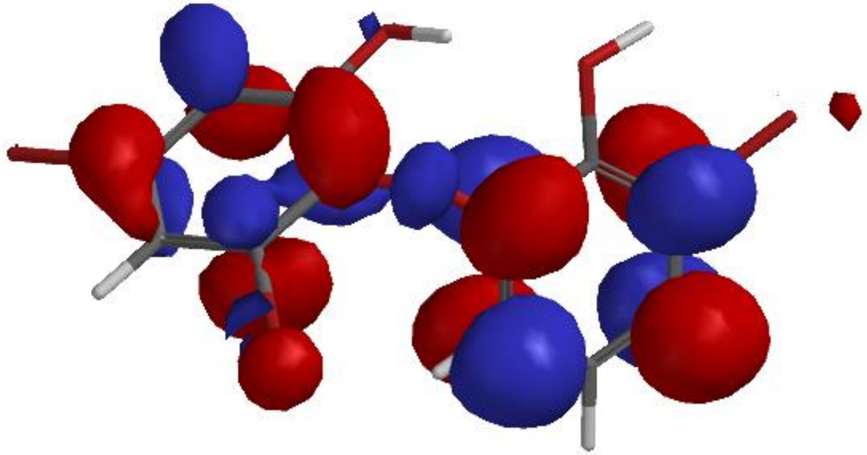

LUMO 22

Figure S99. Calculated ^{13}C NMR Chemical Shift of **22**

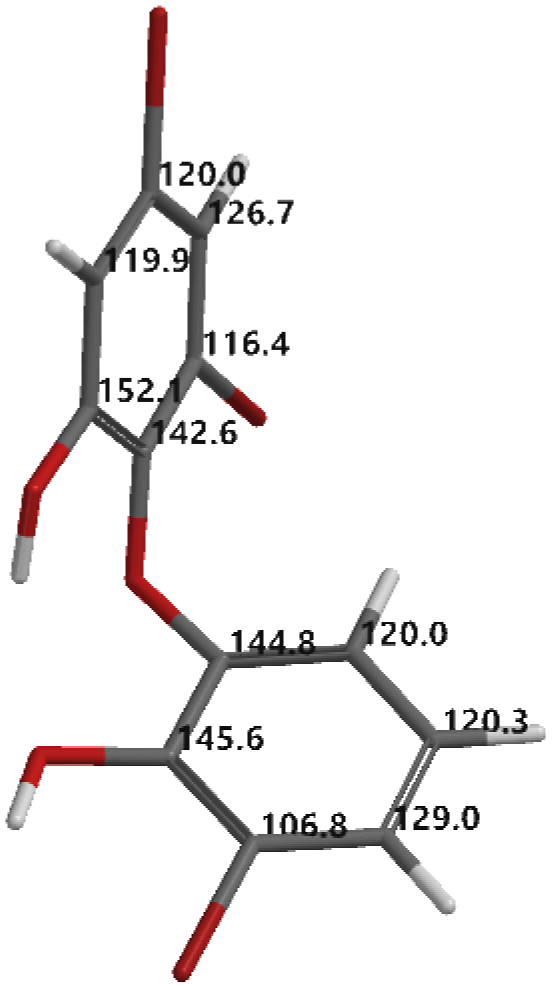

Figure S100. ^{1}H NMR spectrum of **23** (Me_2CO-d_6, 500 MHz)

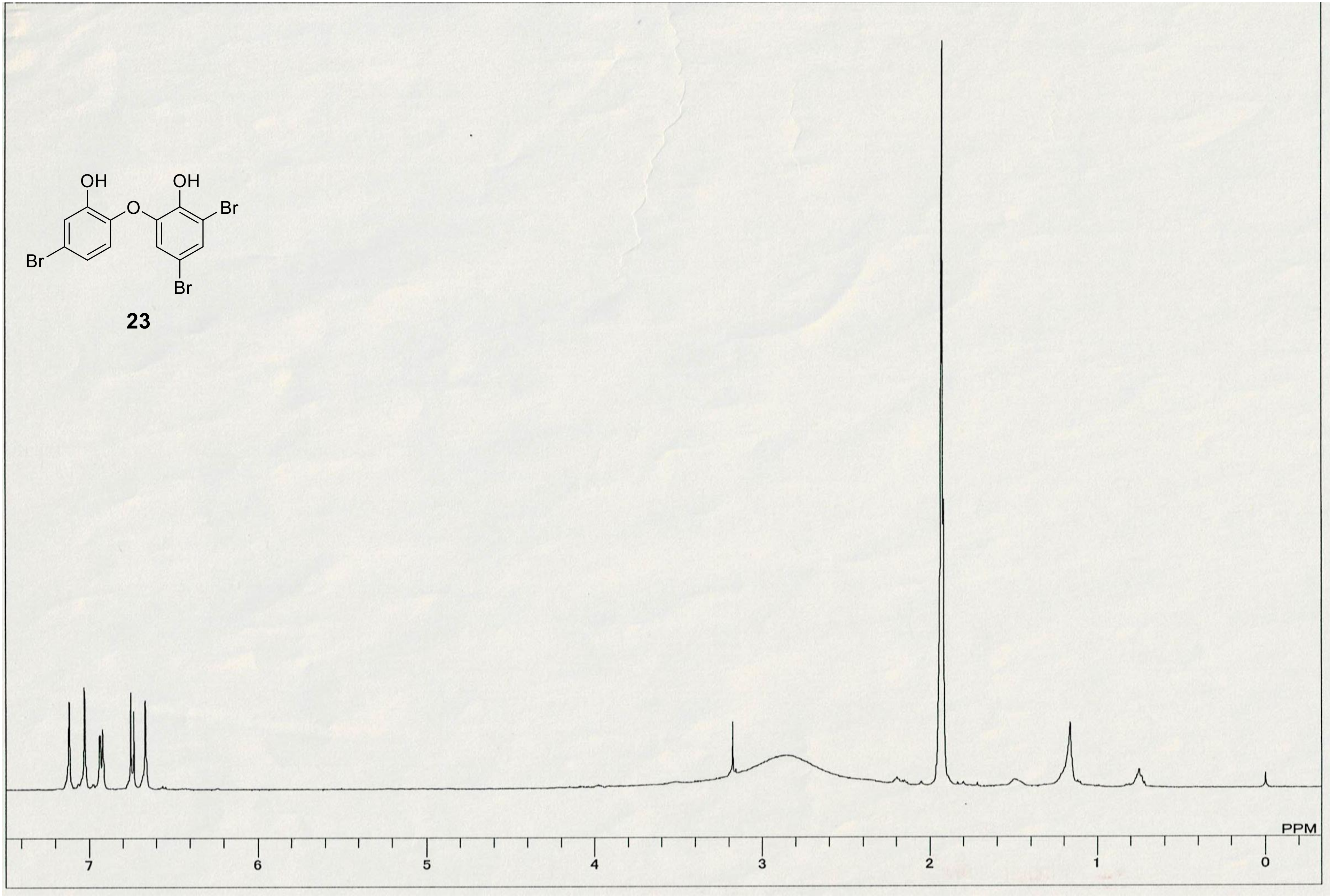

Figure S101. HREIMS of **23**

ターミナル1

SAMPLE : PBDE-110-2A-4
SAMPLE NO.: 760   SCAN NO.: 17

PAGE 1

NO.	PKNO.	P.C.	M/Z(OBS.)	ER(MU)	US	ELEMENTS
1	218	12.6	399.9919			
2	219	5.0	400.9856			
3	220	64.6	404.9760			
4	221	7.6	405.9796			
5	222	6.5	411.9447			
6	223	17.6	416.9760			
7	224	8.5	418.9779			
8	225	3.5	419.7777	-21.8	8.0	C12 H7 Br3 O2
				14.5	9.0	C11 H3 Br3 O3
9	226	7.8	423.9619			
10	227	6.2	424.9922			
11	228	5.5	426.9992			
12	229	4.5	428.9871			
13	230	81.9	430.9728			
14	231	10.1	431.9631			
15	232	11.6	435.7957	1.2	8.0	C12 H7 Br3 O3
16	233	7.3	436.9904			
17	234	10.2	437.7800	27.3	13.0	C14 H1 Br3 O2
				-30.1	7.0	C12 H9 Br3 O3

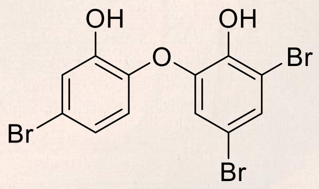

23

Table S47. Equilibrium Geometry DFT ωB97X-D/6-311+G(2d,p) of **23**

```
Job type: Geometry optimization.
Method: RWB97X-D
Basis set: 6-311+G(2D,P)
Number of basis functions: 591
Number of electrons: 208
Parallel Job: 16 threads

SCF model:
 A restricted hybrid HF-DFT SCF calculation will be
 performed using Pulay DIIS + Geometric Direct Minimization


Optimization:
       Step      Energy          Max Grad.      Max Dist.
          1    -8409.634389      0.016739       0.065263
          2    -8409.637882      0.006159       0.034225
          3    -8409.638259      0.002213       0.011345
          4    -8409.638300      0.002233       0.185322
          5    -8409.638611      0.001981       0.018988
          6    -8409.638682      0.001090       0.087197
          7    -8409.638765      0.001048       0.030177
          8    -8409.638795      0.000857       0.119546
          9    -8409.638899      0.001216       0.127146
         10    -8409.638961      0.002789       0.048644
         11    -8409.639026      0.001687       0.083467
         12    -8409.639074      0.001206       0.052942
         13    -8409.639073      0.001148       0.034654
         14    -8409.639087      0.000590       0.012529
```

Figure S102. HOMO-LUMO of **23**

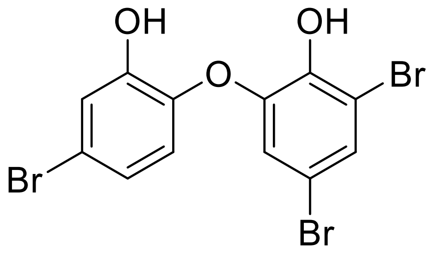

23

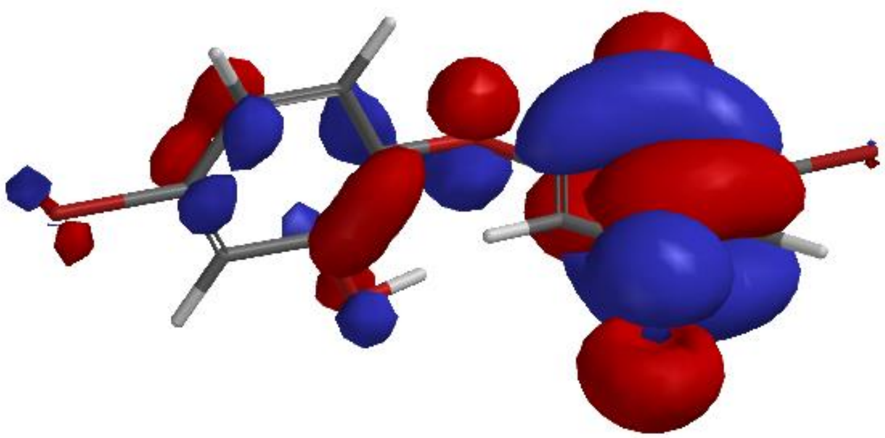

HOMO 23

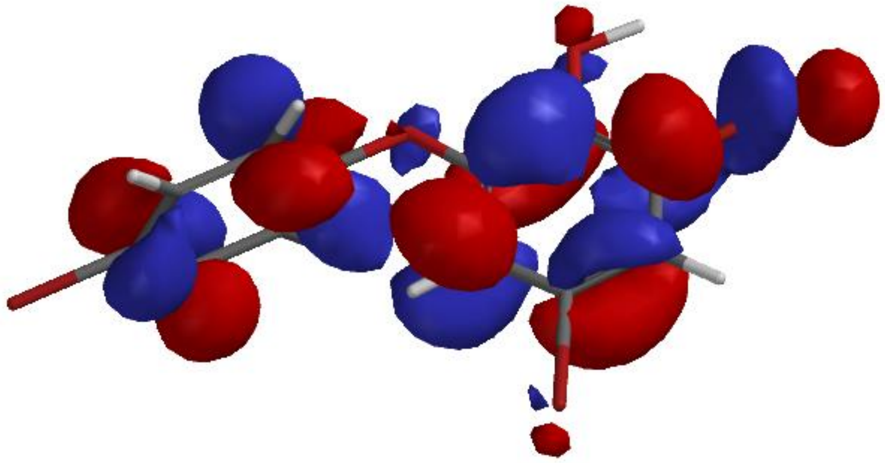

LUMO 23

Figure S103. Calculated ^{13}C NMR Chemical Shift of **23**

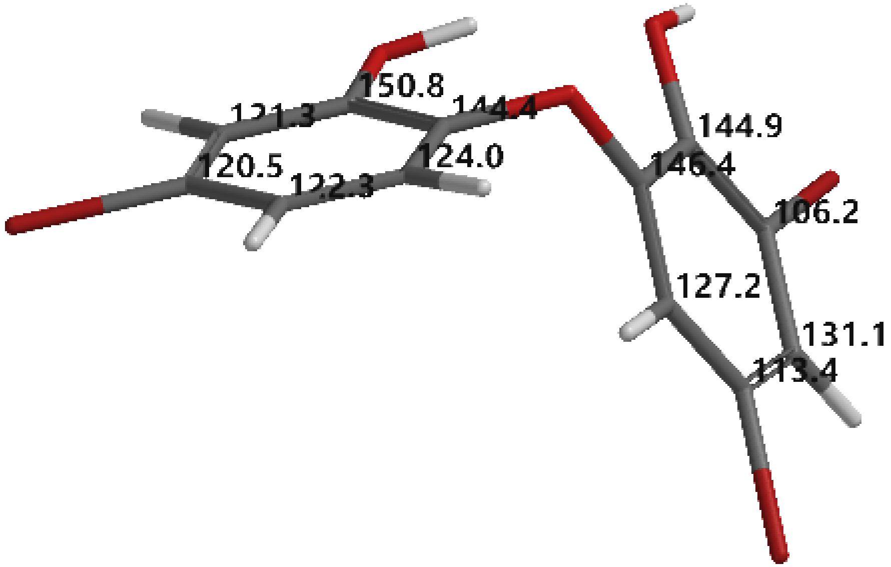

Table S48. Equilibrium Geometry DFT ωB97X-D/6-311+G(2d,p) of **24**

```
Job type: Geometry optimization.
Method: RWB97X-D
Basis set: 6-311+G(2D,P)
Number of basis functions: 591
Number of electrons: 208
Parallel Job: 16 threads

SCF model:
 A restricted hybrid HF-DFT SCF calculation will be
 performed using Pulay DIIS + Geometric Direct Minimization


Optimization:
         Step       Energy          Max Grad.      Max Dist.
            1    -8409.632444       0.016787       0.082560
            2    -8409.636402       0.007465       0.031322
            3    -8409.636768       0.001545       0.010429
            4    -8409.636797       0.001521       0.126229
            5    -8409.636910       0.001247       0.049118
            6    -8409.636960       0.000858       0.059241
            7    -8409.636992       0.000807       0.028110
            8    -8409.637008       0.000818       0.125739
            9    -8409.637075       0.001628       0.045330
           10    -8409.637126       0.002153       0.154828
           11    -8409.637395       0.003611       0.155314
           12    -8409.637600       0.005031       0.224109
           13    -8409.637523       0.010662       0.147016
           14    -8409.638102       0.003629       0.018859
           15    -8409.638261       0.002728       0.132207
           16    -8409.638372       0.003278       0.032379
           17    -8409.638441       0.002243       0.133479
           18    -8409.638608       0.001795       0.136145
           19    -8409.638762       0.001576       0.099217
           20    -8409.638863       0.001767       0.036126
           21    -8409.638910       0.001083       0.044834
           22    -8409.638912       0.000696       0.023248
```

Figure S104. HOMO-LUMO of **24**

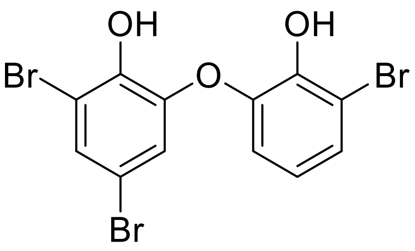

24

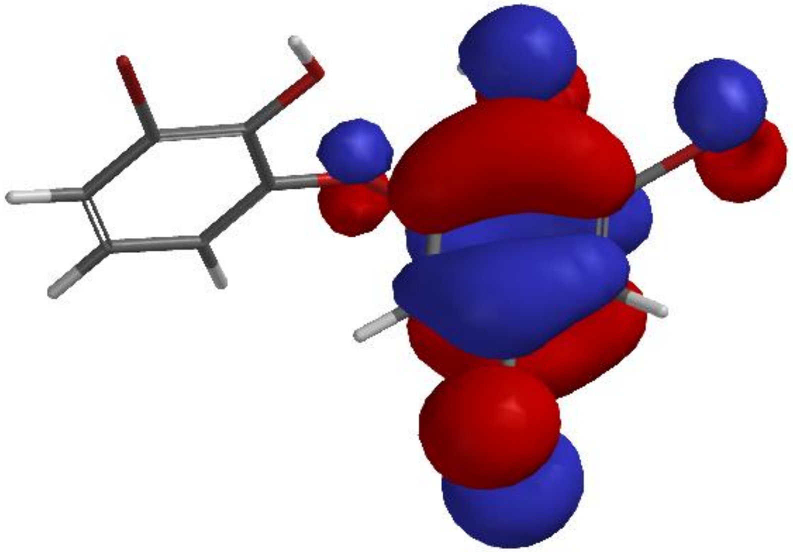

HOMO 24

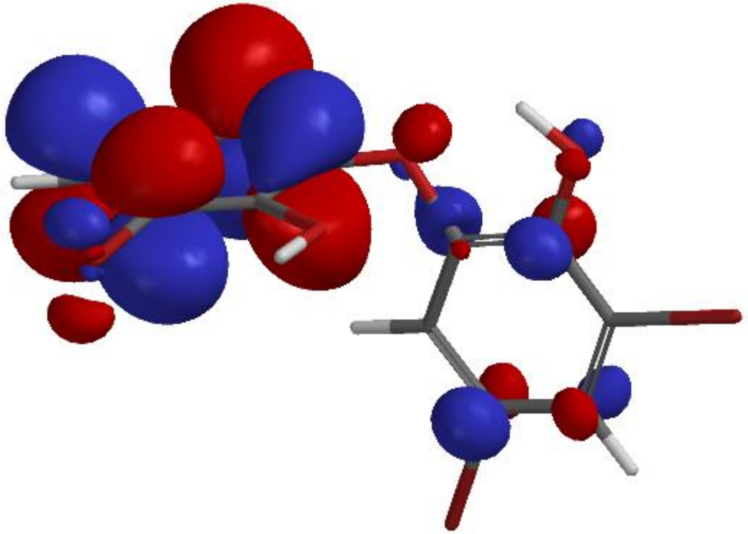

LUMO 24

Figure S105. Calculated ^{13}C NMR Chemical Shift of **24**

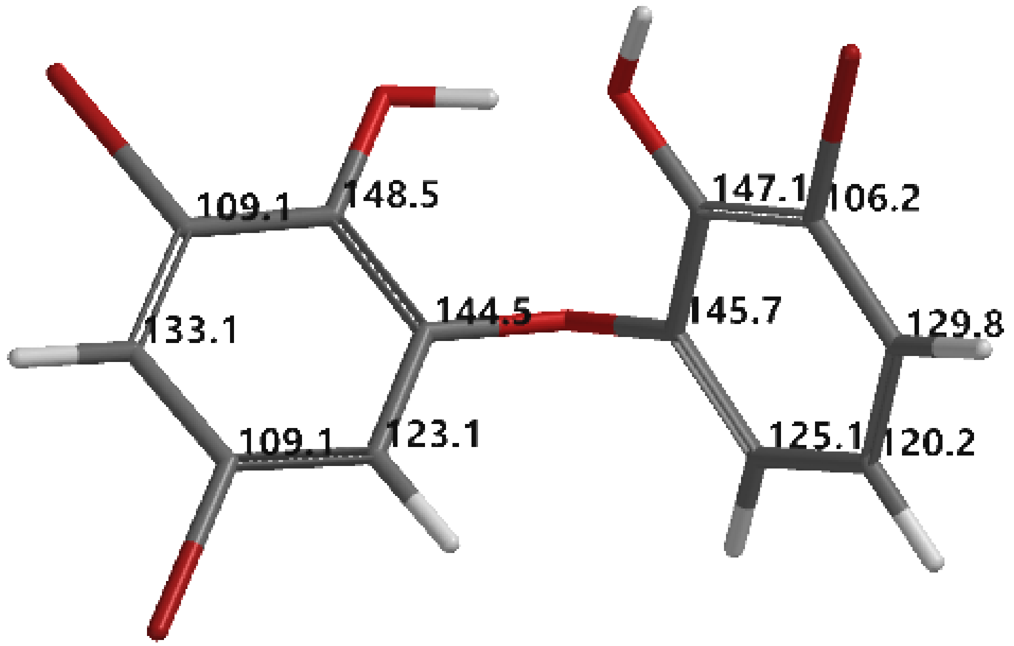

Table S49. Equilibrium Geometry DFT ωB97X-D/6-311+G(2d,p) of **25**

```
Job type: Geometry optimization.
Method: RWB97X-D
Basis set: 6-311+G(2D,P)
Number of basis functions: 549
Number of electrons: 174
Parallel Job: 16 threads

SCF model:
 A restricted hybrid HF-DFT SCF calculation will be
 performed using Pulay DIIS + Geometric Direct Minimization


Optimization:
         Step      Energy          Max Grad.      Max Dist.
            1    -5836.062719       0.016246       0.054932
            2    -5836.065642       0.006155       0.026482
            3    -5836.065966       0.001450       0.017384
            4    -5836.066013       0.001454       0.087323
            5    -5836.066128       0.001001       0.061258
            6    -5836.066146       0.000780       0.087930
            7    -5836.066193       0.000980       0.038614
            8    -5836.066212       0.000899       0.081279
            9    -5836.066254       0.000949       0.190805
           10    -5836.063372       0.027098       0.122667
           11    -5836.066322       0.001013       0.113282
           12    -5836.066419       0.003404       0.047946
           13    -5836.066492       0.002043       0.036870
           14    -5836.066518       0.000259       0.014888
           15    -5836.066519       0.000223       0.008911
```

Figure S106. HOMO-LUMO of **25**

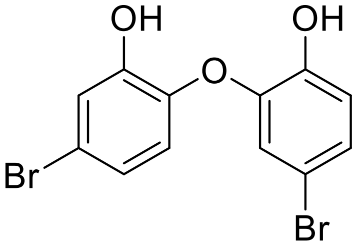

25

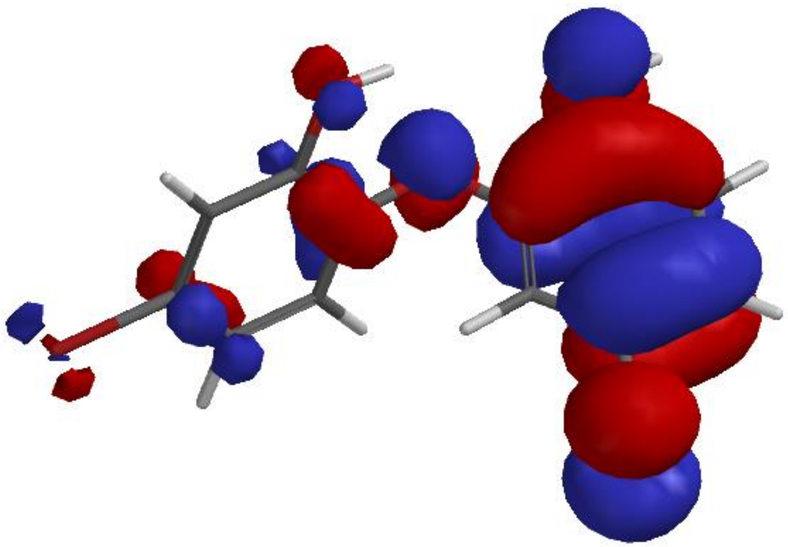

HOMO 25

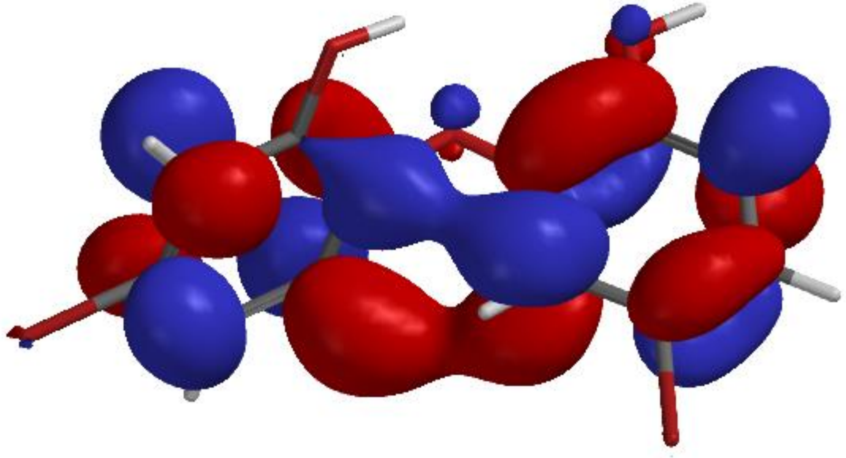

LUMO 25

Figure S107. Calculated ^{13}C NMR Chemical Shift of **25**

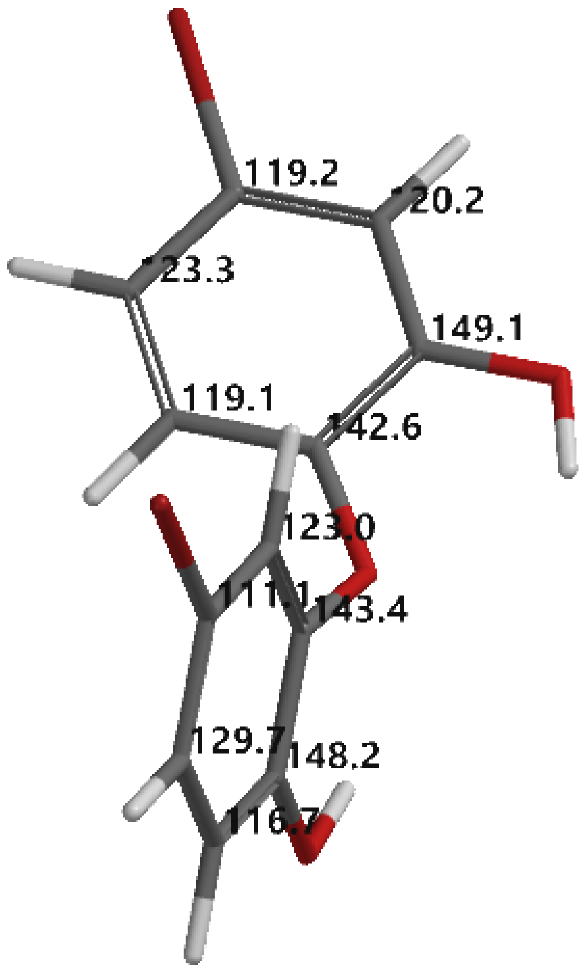

Figure S108. ^{1}H NMR spectrum of **26** (CD_3OD, 500 MHz)

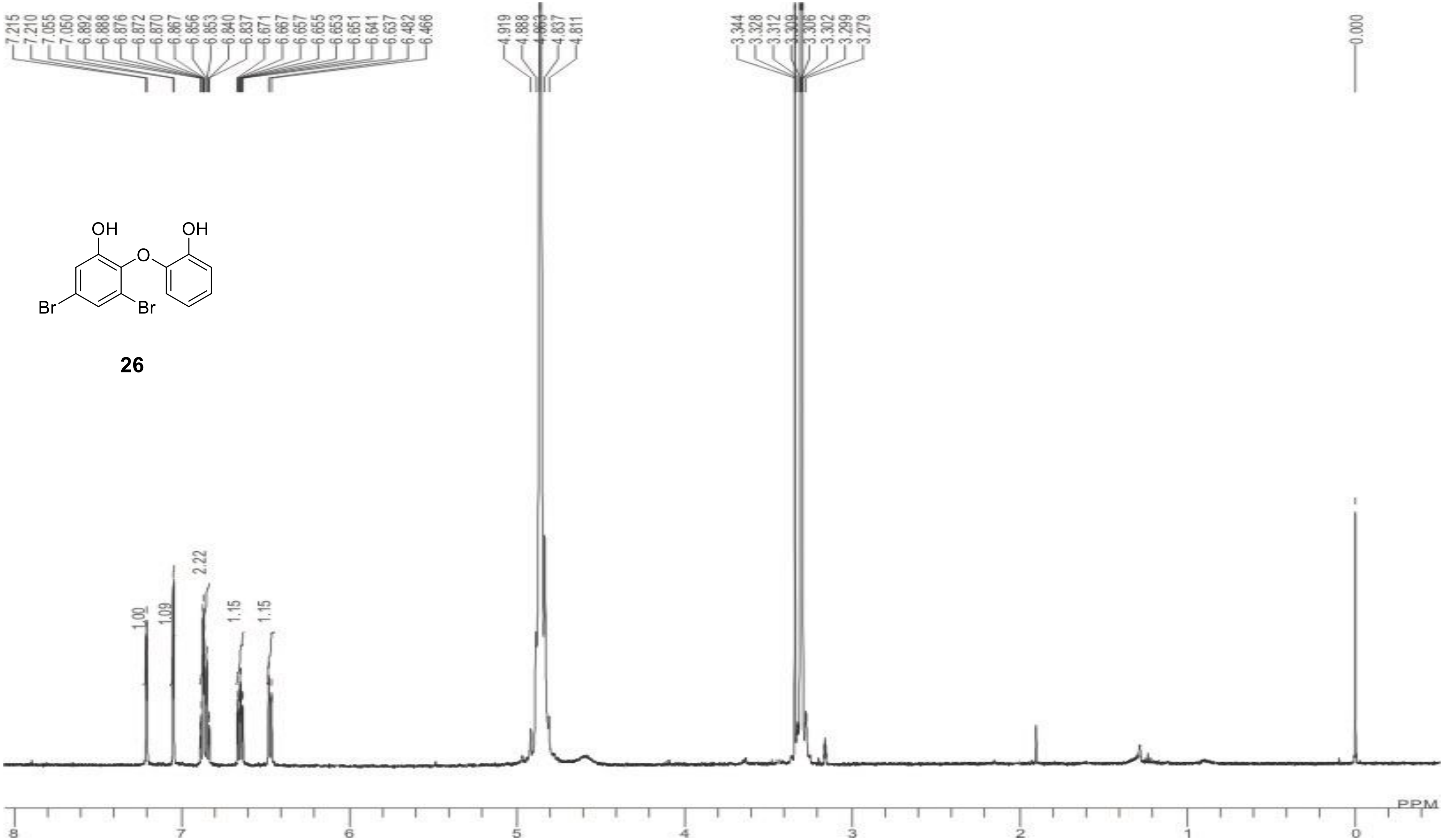

Figure S109. HREIMS of **26**

ターミナル1

SAMPLE : PBDE-110-31
SAMPLE NO.: 759 SCAN NO.: 10

PAGE 3

NO.	PKNO.	P.C.	M/Z(OBS.)	ER(MU)	US	ELEMENTS
36	254	0.6	338.0081			
37	255	0.5	339.0316			
38	256	0.6	339.8710	-38.7	8.0	C13 H10 Br2 O1
				- 2.3	9.0	C12 H6 Br2 O2
				34.0	10.0	C11 H2 Br2 O3
39	257	0.4	341.0106			
40	258	0.4	341.8780	-11.0	8.0	C12 H8 Br2 O2
				25.3	9.0	C11 H4 Br2 O3
41	259	43.1	342.9792			
42	260	5.2	343.9887			
43	261	1.0	344.9995			
44	262	2.2	346.9730			
45	263	0.4	347.9721			
46	264	0.7	348.9776			
47	265	0.6	349.9687			
48	266	1.1	351.0080			
49	267	11.1	354.9792			
50	268	2.0	355.9720			
51	269	0.7	356.9661			
52	270	1.8	357.8824	- 1.5	8.0	C12 H8 Br2 O3

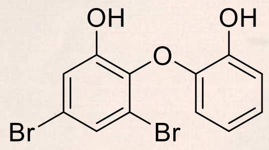

26

Table S50. Equilibrium Geometry DFT ωB97X-D/6-311+G(2d,p) of **26**

```
Job type: Geometry optimization.
Method: RWB97X-D
Basis set: 6-311+G(2D,P)
Number of basis functions: 549
Number of electrons: 174
Parallel Job: 16 threads

SCF model:
 A restricted hybrid HF-DFT SCF calculation will be
 performed using Pulay DIIS + Geometric Direct Minimization


Optimization:
        Step      Energy          Max Grad.      Max Dist.
           1    -5836.061537       0.017002       0.108180
           2    -5836.065659       0.005031       0.109740
           3    -5836.066063       0.002040       0.007664
           4    -5836.066100       0.000938       0.005806
           5    -5836.066109       0.000241       0.003234
```

Figure S110. HOMO-LUMO of **26**

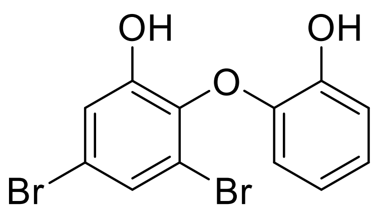

26

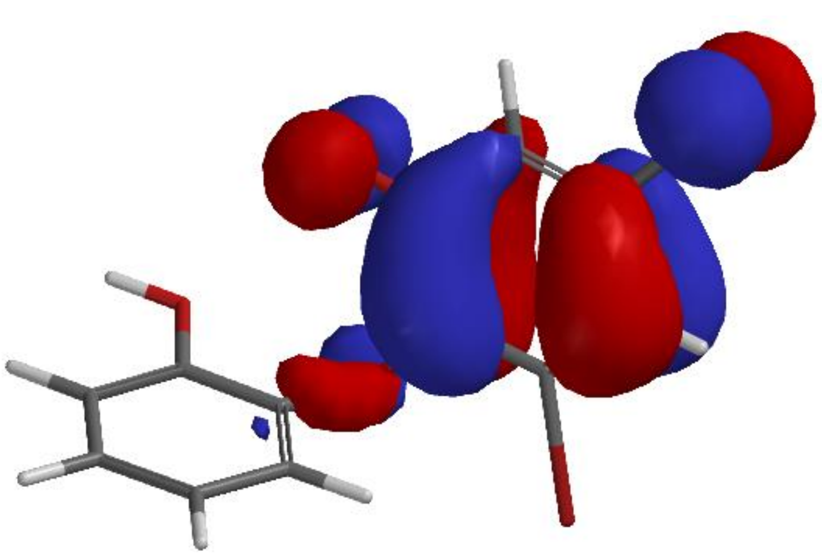

HOMO 26

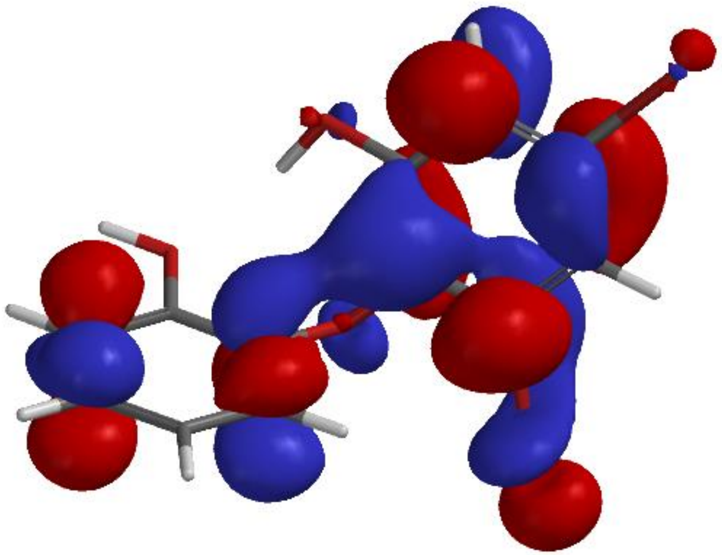

LUMO 26

Figure S111. Calculated ^{13}C NMR Chemical Shift of **26**

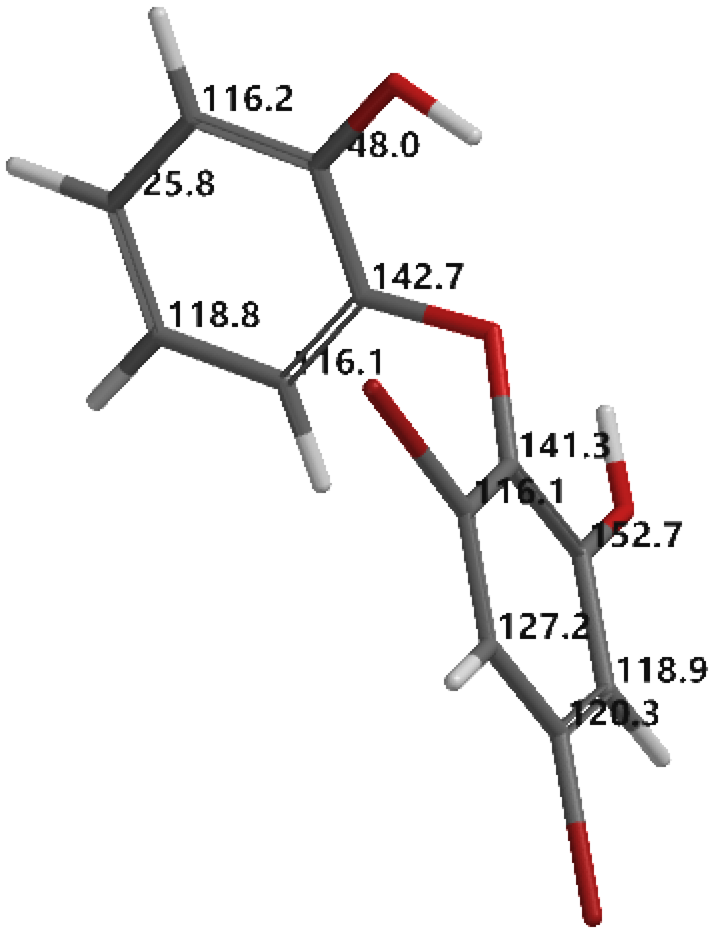

Table S51. Equilibrium Geometry DFT ωB97X-D/6-311+G(2d,p) of **27**

```
Job type: Geometry optimization.
Method: RWB97X-D
Basis set: 6-311+G(2D,P)
Number of basis functions: 549
Number of electrons: 174
Parallel Job: 16 threads

SCF model:
 A restricted hybrid HF-DFT SCF calculation will be
 performed using Pulay DIIS + Geometric Direct Minimization


Optimization:
         Step       Energy          Max Grad.       Max Dist.
            1    -5836.063149       0.017000        0.060994
            2    -5836.066525       0.006244        0.029606
            3    -5836.066856       0.001845        0.014476
            4    -5836.066899       0.001919        0.167341
            5    -5836.067112       0.001970        0.050329
            6    -5836.067205       0.001829        0.157225
            7    -5836.067413       0.002573        0.085936
            8    -5836.067511       0.004582        0.057463
            9    -5836.067630       0.002905        0.075926
           10    -5836.067774       0.001150        0.044421
           11    -5836.067822       0.000970        0.021432
           12    -5836.067830       0.000263        0.012001
```

Figure S112. HOMO-LUMO of **27**

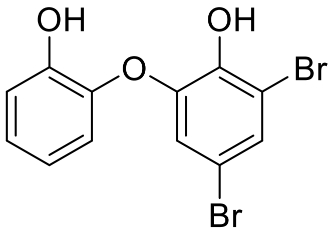

27

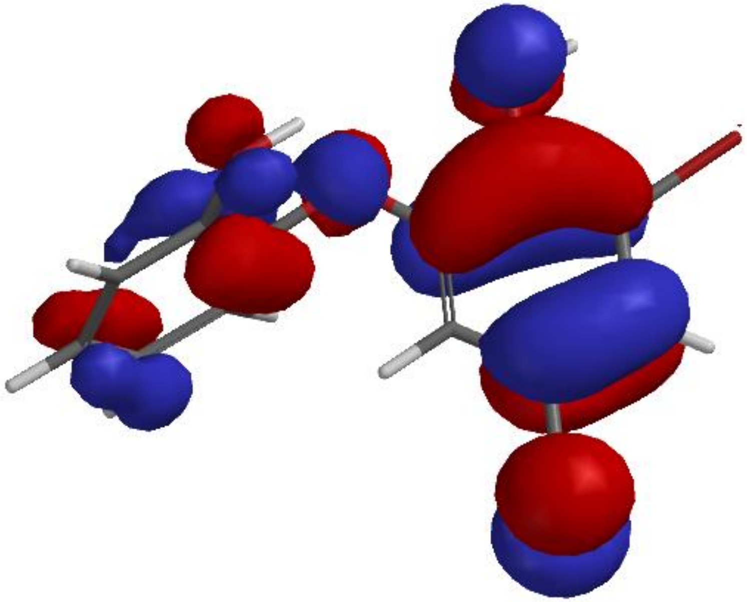

HOMO 27

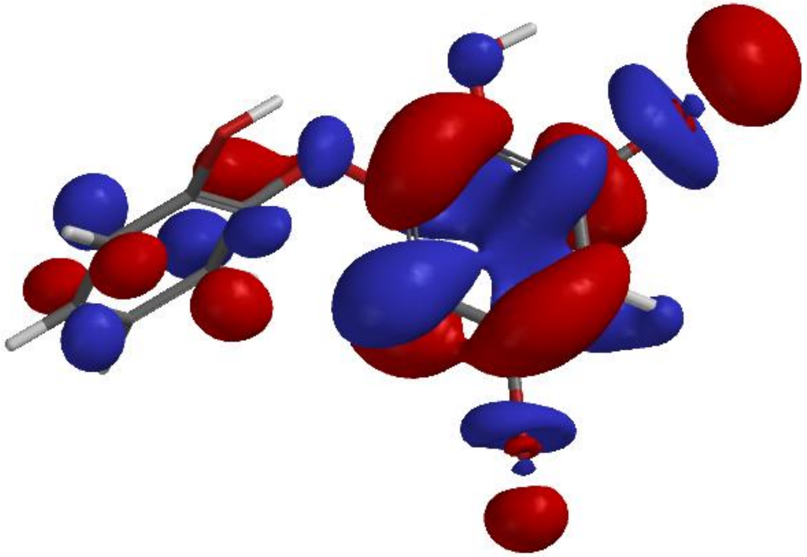

LUMO 27

Figure S113. Calculated ^{13}C NMR Chemical Shift of **27**

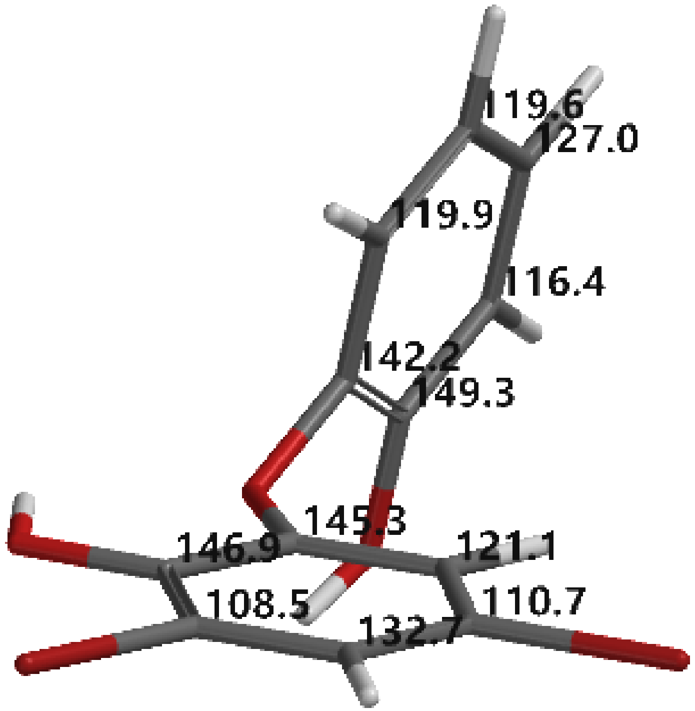

Table S52. Equilibrium Geometry DFT ωB97X-D/6-311+G(2d,p) of **28**

```
Job type: Geometry optimization.
Method: RWB97X-D
Basis set: 6-311+G(2D,P)
Number of basis functions: 507
Number of electrons: 140
Parallel Job: 16 threads

SCF model:
 A restricted hybrid HF-DFT SCF calculation will be
 performed using Pulay DIIS + Geometric Direct Minimization


Optimization:
         Step       Energy            Max Grad.      Max Dist.
            1    -3262.491099       0.016820       0.061238
            2    -3262.493993       0.006810       0.029800
            3    -3262.494318       0.001731       0.018173
            4    -3262.494368       0.001732       0.125139
            5    -3262.494530       0.001178       0.068377
            6    -3262.494592       0.000941       0.091630
            7    -3262.494643       0.001489       0.152496
            8    -3262.494801       0.002630       0.146192
            9    -3262.494680       0.005622       0.109539
           10    -3262.494877       0.001934       0.013981
           11    -3262.494916       0.001601       0.055904
           12    -3262.494984       0.000956       0.048517
           13    -3262.495022       0.000974       0.043055
           14    -3262.495030       0.000291       0.015718
```

Figure S114. HOMO-LUMO of **28**

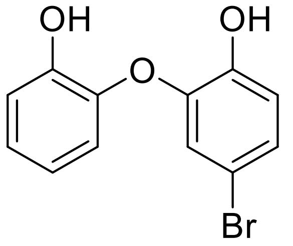

28

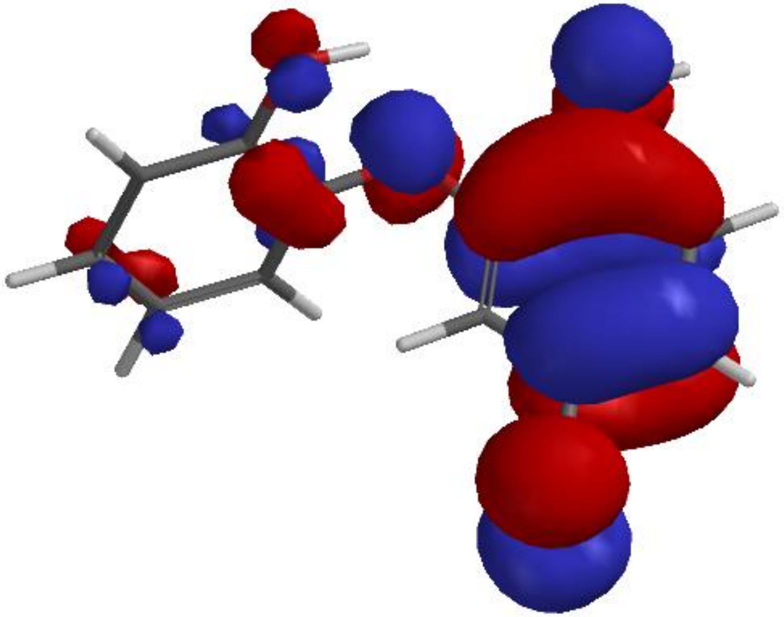

HOMO 28

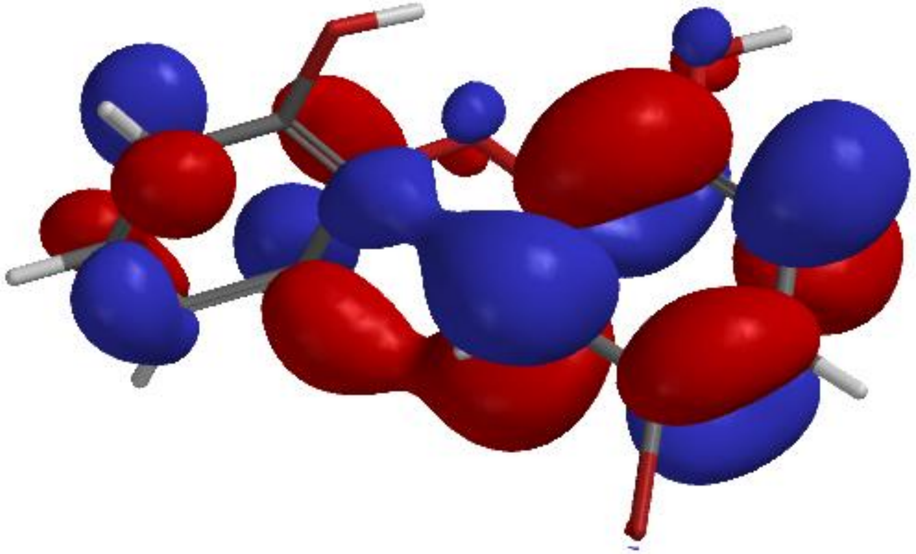

LUMO 28

Figure S115. Calculated ^{13}C NMR Chemical Shift of **28**

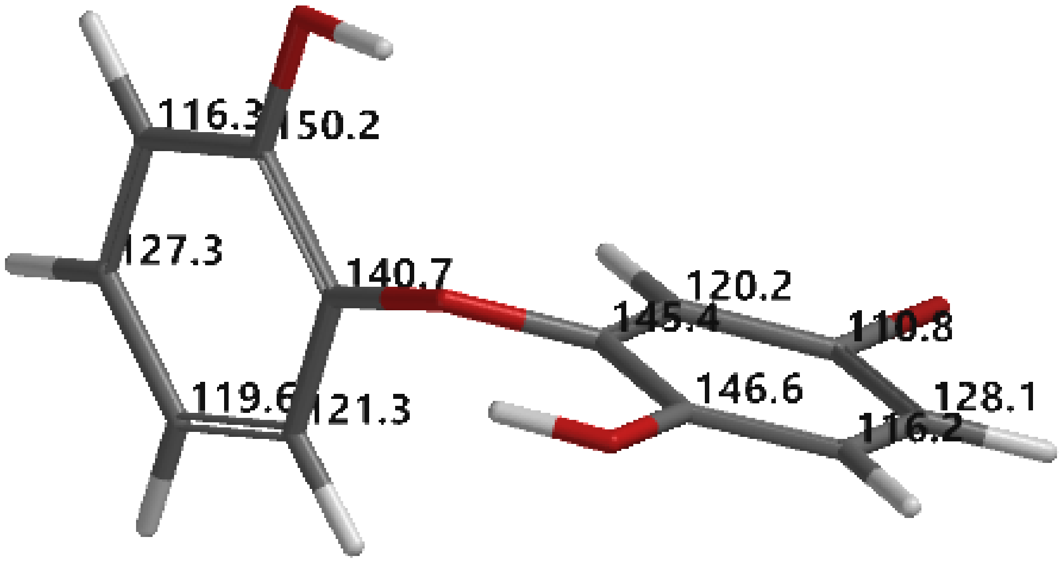

Table S53. Equilibrium Geometry DFT ωB97X-D/6-311+G(2d,p) of **29**

```
Job type: Geometry optimization.
Method: RWB97X-D
Basis set: 6-311+G(2D,P)
Number of basis functions: 507
Number of electrons: 140
Parallel Job: 16 threads

SCF model:
 A restricted hybrid HF-DFT SCF calculation will be
 performed using Pulay DIIS + Geometric Direct Minimization


Optimization:
         Step       Energy          Max Grad.       Max Dist.
            1    -3262.493067       0.016774        0.060603
            2    -3262.496457       0.006546        0.032003
            3    -3262.496841       0.001784        0.011009
            4    -3262.496872       0.001785        0.135742
            5    -3262.497002       0.001114        0.021546
            6    -3262.497030       0.000804        0.051283
            7    -3262.497098       0.000724        0.103100
            8    -3262.497164       0.001238        0.161182
            9    -3262.497355       0.001839        0.149424
           10    -3262.497318       0.004700        0.087265
           11    -3262.497447       0.001239        0.032768
           12    -3262.497478       0.000822        0.052254
           13    -3262.497503       0.000267        0.019419
           14    -3262.497505       0.000102        0.002946
```

Figure S116. HOMO-LUMO of **29**

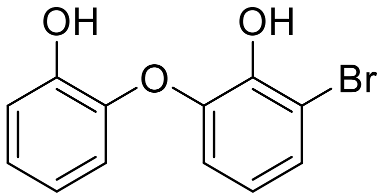

29

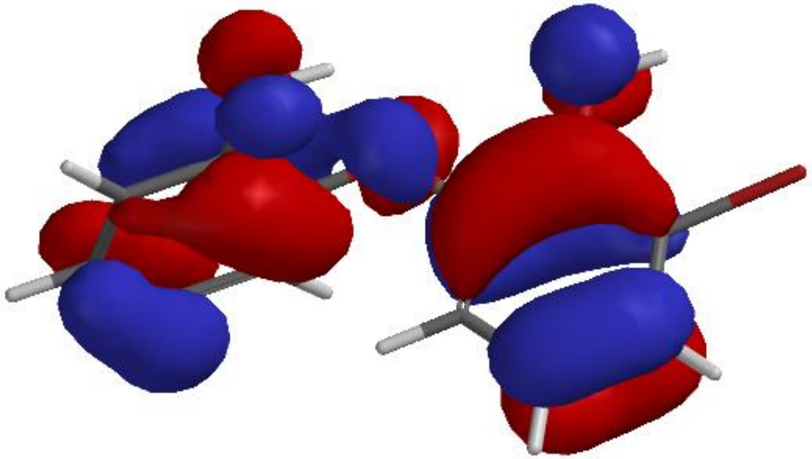

HOMO 29

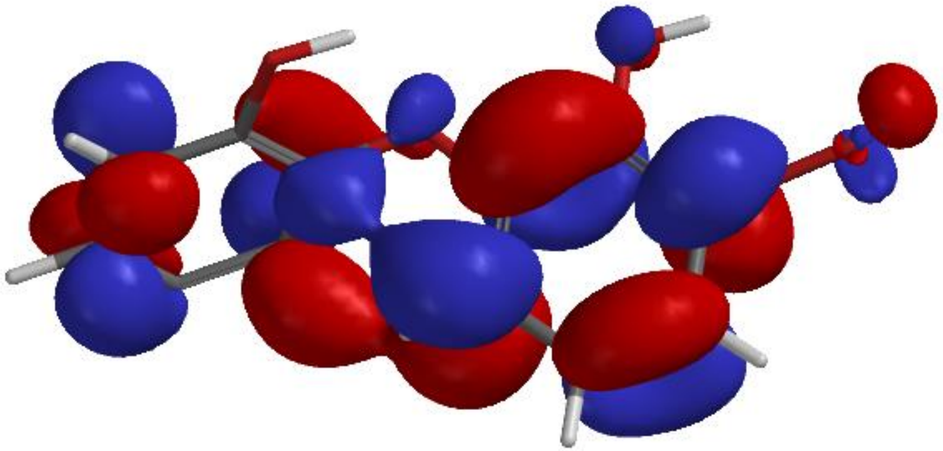

LUMO 29

Figure S117. Calculated ^{13}C NMR Chemical Shift of **29**

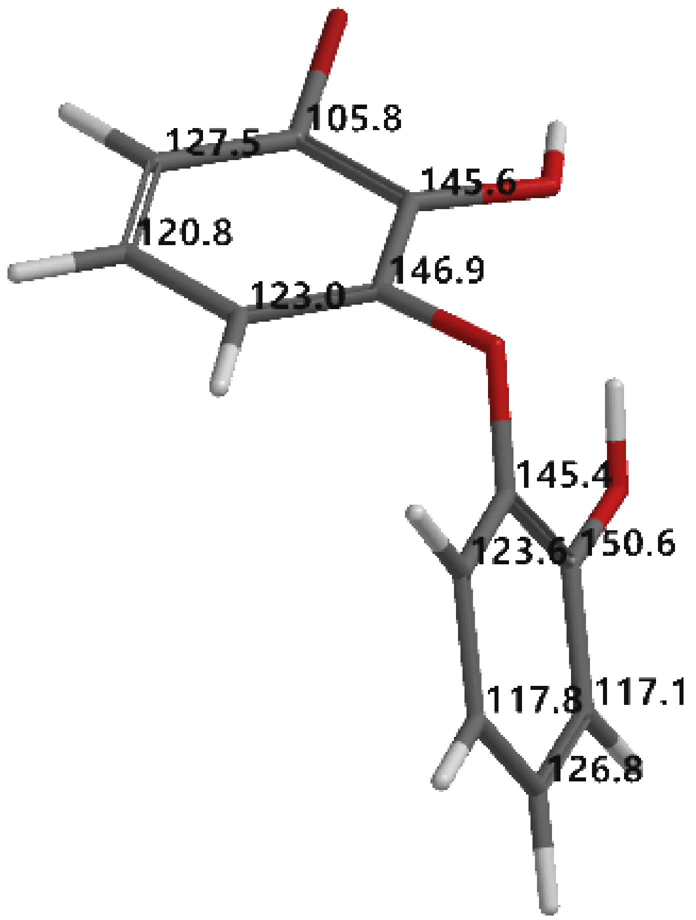

Table S54. Equilibrium Geometry DFT ωB97X-D/6-311+G(2d,p) of **30**

```
Job type: Geometry optimization.
Method: RWB97X-D
Basis set: 6-311+G(2D,P)
Number of basis functions: 465
Number of electrons: 106
Parallel Job: 16 threads

SCF model:
 A restricted hybrid HF-DFT SCF calculation will be
 performed using Pulay DIIS + Geometric Direct Minimization


Optimization:
         Step       Energy          Max Grad.       Max Dist.
            1     -688.920186        0.016180        0.049697
            2     -688.922801        0.007123        0.022371
            3     -688.923050        0.001042        0.004455
            4     -688.923061        0.000928        0.051355
            5     -688.923115        0.000467        0.029738
            6     -688.923131        0.000541        0.039364
```

Figure S118. HOMO-LUMO of **30**

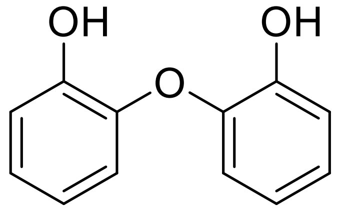

30

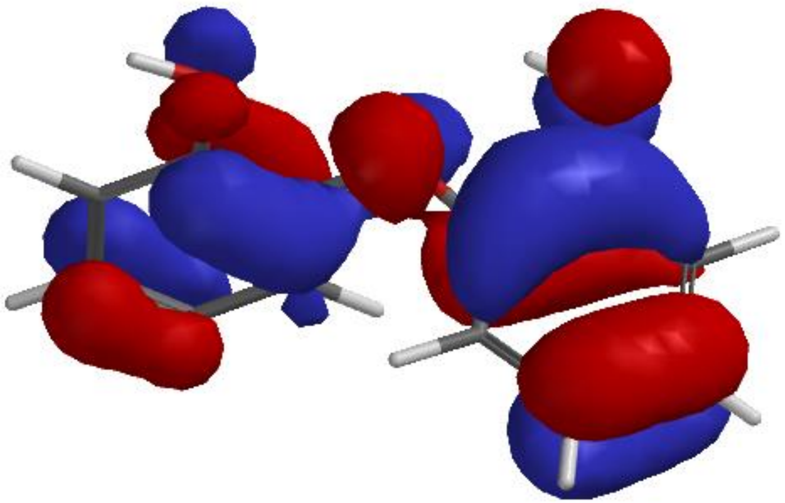

HOMO 30

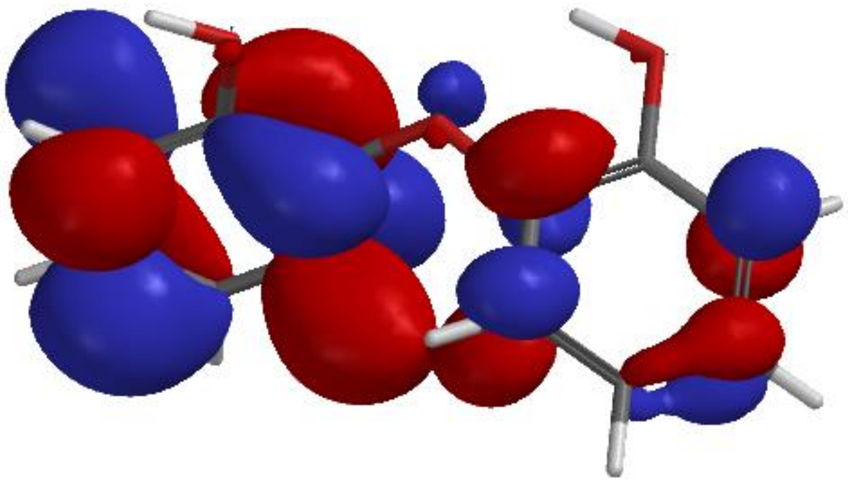

LUMO 30

Figure S119. Calculated ^{13}C NMR Chemical Shift of **30**

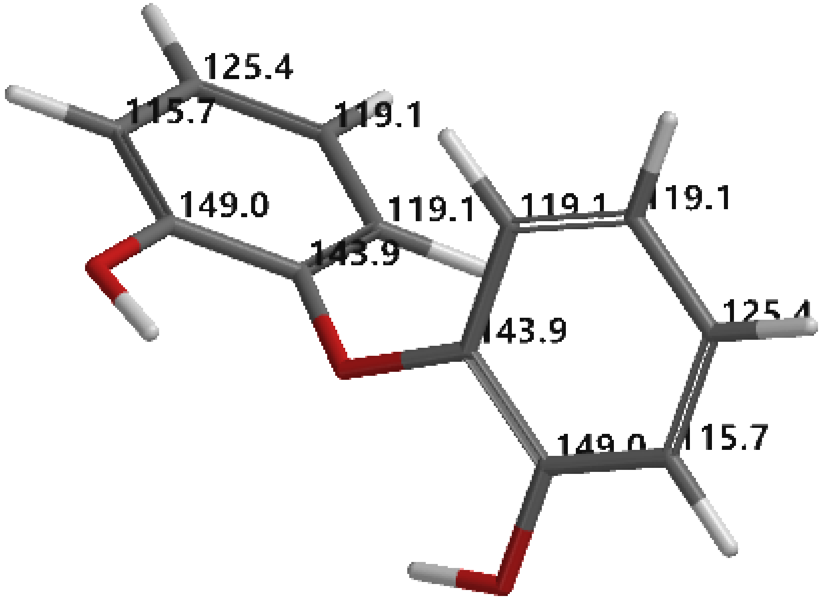

Table S55. ^{1}H NMR Data of **1–5**

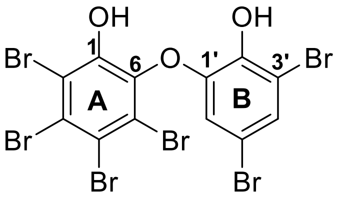

1

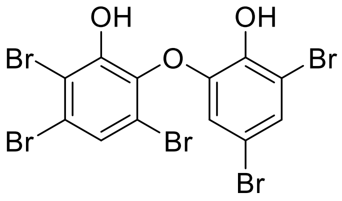

2

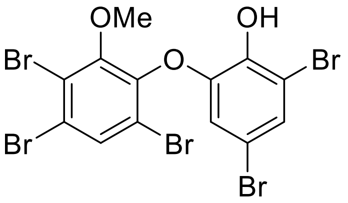

3

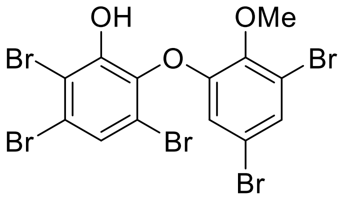

4

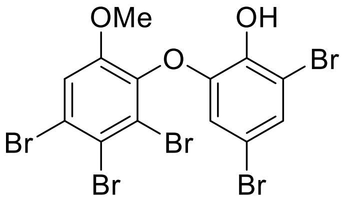

5

Pos	Compound					
	**1** (Me_2CO-d_6)	**1** (CD_3OD)	**2** (Me_2CO-d_6)	**3** ($CDCl_3$)	**4** (Me_2CO-d_6)	**5** ($CDCl_3$)
H4′	7.40 (1H, d, *J* = 2.2 Hz)	7.35 (1H, d, *J* = 2.2 Hz)	7.38 (1H, d, *J* = 2.3 Hz)	7.38 (1H, d, *J* = 2.3 Hz)	7.47 (1H, d, *J* = 2.3 Hz)	7.47 (1H, d, *J* = 2.3 Hz)
H6′	6.83 (1H, d, *J* = 2.2 Hz)	6.47 (1H, d, *J* = 2.2 Hz)	6.79 (1H, d, *J* = 2.3 Hz)	6.56 (1H, d, *J* = 2.3 Hz)	6.81 (1H, d, *J* = 2.3 Hz)	6.52 (1H, d, *J* = 2.3 Hz)
H2	-	-	-	-	-	7.31 (1H, s)
H4	-	-	7.47 (1H, s)	7.75 (1H, s)	7.64 (1H, s)	-
OMe′ (Ring B)	-	-	-	-	4.00 (3H, s)	-
OMe (Ring A)	-	-	-	3.81 (3H, s)	-	3.79 (3H, s)

Table S56. NMR Data of **6** in $Me_2CO\text{-}d_6$

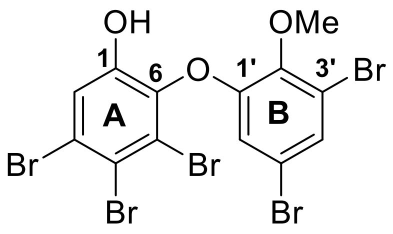

6

Pos	δ_C (mult.)	δ_H (J in Hz)	HMBC
1	151.3 C		
2	122.0 CH	7.50 (1H, s)	1, 3, 4, 6
3	122.5 C		
4	116.9 C		
5	122.7 C		
6	140.1 C		
2′-OMe (Ring B)	61.1 CH_3	3.99 (3H, s)	2'
1'	152.0 C		
2'	146.5 C		
3'	119.4 C		
4'	129.4 CH	7.40 (1H, d, J = 2.5)	2', 3', 5', 6'
5'	117.8 C		
6'	117.4 CH	6.78 (1H, d, J = 2.5)	1', 2', 3', 5'

Table S57. ^{1}H NMR Data of **7–9**

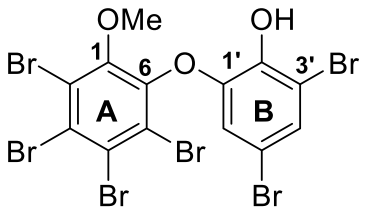

7

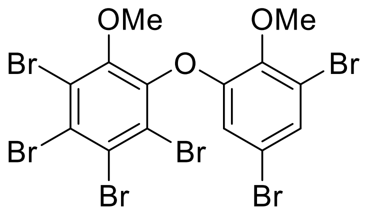

8

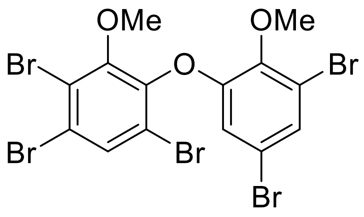

9

**Pos**	**Compound**		
	**7** (Me_2CO-d_6)	**8** ($CDCl_3$)	**9** ($CDCl_3$)
H4′	7.43 (1H, d, J = 2.2 Hz)	7.50 (1H, d, J = 2.2 Hz)	7.40 (1H, d, J = 2.2 Hz)
H6′	6.88 (1H, d, J = 2.2 Hz)	6.97 (1H, d, J = 2.2 Hz)	6.50 (1H, d, J = 2.2 Hz)
H2	-	-	-
H4	-	-	7.76 (1H, s)
OMe′ (Ring B)	-	4.00 (3H, s)	4.00 (3H, s)
OMe (Ring A)	3.87 (3H, s)	3.85 (3H, s)	3.82 (3H, s)

Table S58. ^{1}H NMR Data of **10–13**

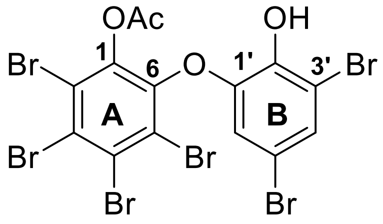

10

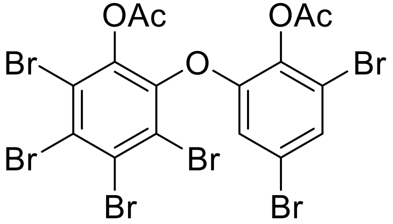

11

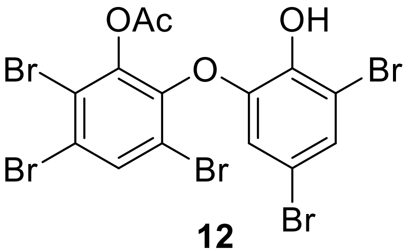

12

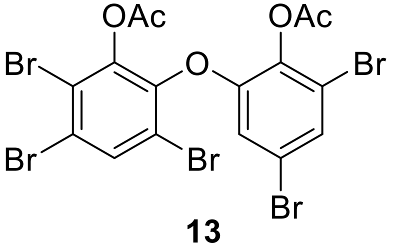

13

**Pos**	**Compound**						
	**10 ($CDCl_3$)**	**10 (Me_2CO-d_6)**	**11 (Me_2CO-d$_6$)**	**12 ($CDCl_3$)**	**12 (Me_2CO-d_6)**	**13 ($CDCl_3$)**	**13 (Me_2CO-d_6)**
H4′	7.51 (1H, d, J = 2.1 Hz)	7.62 (1H, d, J = 2.2 Hz)	7.64 (1H, d, J = 2.1 Hz)	7.50 (1H, d, J = 2.1 Hz)	7.62 (1H, d, J = 2,1 Hz)	7.47 (1H, d, J = 2.0 Hz)	7.63 (1H, d, J = 2.1 Hz)
H6′	6.62 (1H, d, J = 2.1 Hz)	7.12 (1H, d, J = 2.2 Hz)	7.14 (1H, d, J = 2.1 Hz)	6.63 (1H, d, J = 2.1 Hz)	7.00 (1H, d, J = 2,1 Hz)	6.58 (1H, d, J = 2.0 Hz)	7.01 (1H, d, J = 2.1 Hz)
H2	-	-	-	-	-	-	-
H4	-	-	-	7.54 (1H, s)	7.66 (1H, s)	7.87 (s, 1H)	8.13 (s, 1H)
OAc′ (Ring B)	-	-	2.36 (3H, s)	-	-	2.36 (s, 3H)	2.36 (s, 3H)
OAc (Ring A)	2.47 (3H, s)	2.40 (3H, s)	2.24 (3H, s)	2.47 (3H, s)	2.40 (3H, s)	2.25 (s, 3H)	2.24 (s, 3H)

Table S59. ^{1}H NMR Data of **2, 14, 16–18**

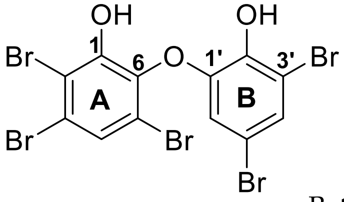

2

Ratio **2:14**
1:1

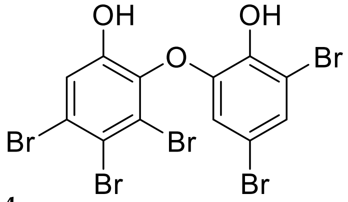

14

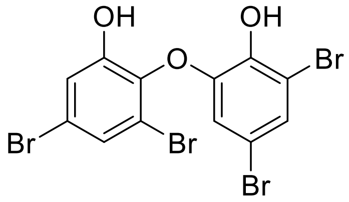

16

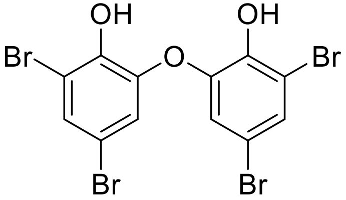

17

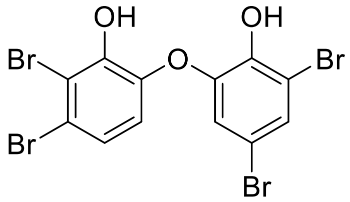

18

**Pos**	**Compound**					
	**2** (CD_3OD)	**14** (CD_3OD)	**16** (Me_2CO-d_6)	**16** (Me_2CO-d_6)	**17** (Me_2CO-d_6)	**18** (Me_2CO-d_6)
H4′	7.32 (1H, d, J = 2.3 Hz)	7.31 (1H, d, J = 2.3 Hz)	7.38 (1H, d, J = 2.5 Hz)	7.36 (1H, d, J = 2.3 Hz)	7.27 (1H, d, J = 2.5 Hz)	7.81 (1H, d, J = 2.5 Hz)
H6′	6.65 (1H, bs)	6.45 (1H, d, J = 2.3 Hz)	6.64 (1H, d, J = 2.5 Hz)	6.84 (1H, d, J = 2.3 Hz)	6.69 (1H, d, J = 2.5 Hz)	6.67 (d, overlapped)
H2	-	7.38 (1H, s)	7.24 (1H, d, J = 2.5 Hz)	7.16 (1H, d, J = 2.3 Hz)		-
H3	-	-	-	-	7.78 (1H, d, J = 2.5 Hz)	-
H4	7.39 (1H, bs)	-	7.37 (1H, d, J = 2.5 Hz)	7.27 (1H, d, J = 2.3 Hz)	-	7.03 (1H, dd, J = 8.5 Hz)
H5	-	-	-	-	6.74 (1H, d, J = 2.5 Hz)	7.03 (1H, dd, J = 8.5 Hz)

Table S60. ^{1}H NMR Data of **20, 24, 26**

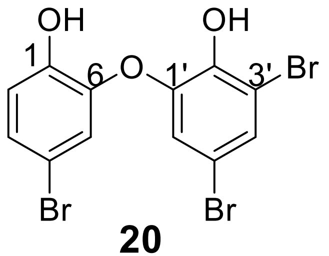

20

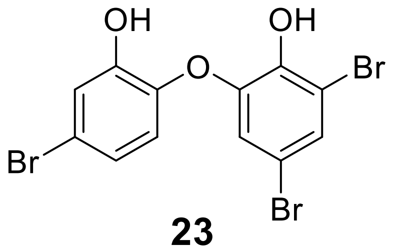

23

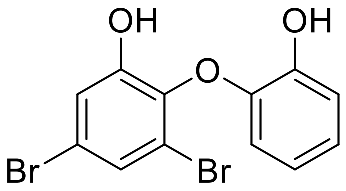

26

Pos	Compound			
	**20** (Me_2CO-d_6)	**20** (CD_3OD)	**23** (Me_2CO-d_6)	**26** (CD_3OD)
H3′	-	-		6.87 (1H, d, *J* = 2.0 Hz)
H4′	7.37 (1H, d, *J* = 2.3 Hz)	7.07 (1H, d, *J* = 2.0 Hz)	7.03 (1H, d, *J* = 2.0 Hz)	6.87 (1H, dq, *J* = 8.0, 2.0 Hz)
H5′	-	-		6.62 (1H, td, *J* = 8.0, 2.0 Hz)
H6′	6.60 (1H, d, *J* = 2.3 Hz)	6.79 (1H, q, *J* = 2.0 Hz)	6.67 (1H, d, *J* = 2.0 Hz)	6.48 (1H, d, *J* = 8.0 Hz)
H2	6.91 (1H, d, *J* = 8.5 Hz)	6.74 (1H, d, *J* = 8.5 Hz)	7.12 (1H, d, *J* = 2.0 Hz)	7.05 (1H, d, *J* = 2.5 Hz)
H3	7.06 (1H, dd, *J* = 8.5, 2.3 Hz)	6.97 (1H, dd, *J* = 8.5, 2.0 Hz)		-
H4	-	-	6.93 (1H, dd, *J* = 8.5, 2.0 Hz)	7.22 (1H, d, *J* = 2.5 Hz)
H5	7.27 (1H, d, *J* = 2.3 Hz)	6.79 (1H, q, *J* = 2.0 Hz)	6.75 (1H, d, *J* = 8.5 Hz)	-

Table S61. E_{HOMO}, E_{LUMO}, and ΔE for **1–30**

Compound	E_{HOMO} (eV)	E_{LUMO} (eV)	ΔE (E_{LUMO}-E_{HOMO}) (eV)
1	-8.67	-0.05	8.62
2	-8.75	0.27	9.02
3	-8.48	0.33	8.81
4	-8.65	0.21	8.86
5	-8.48	0.28	8.76
6	-8.79	0.22	9.01
7	-8.52	-0.08	8.44
8	-8.74	-0.12	8.62
9	-8.71	0.29	9.00
10	-8.60	-0.26	8.34
11	-9.12	-0.47	8.65
12	-8.56	0.14	8.70
13	-9.10	-0.02	9.08
14	-8.62	0.20	8.82
15	-8.48	0.18	8.66
16	-8.76	0.38	9.14
17	-8.44	0.27	8.71
18	-8.51	0.35	8.86
19	-8.37	0.29	8.66
20	-8.30	0.43	8.73
21	-8.67	0.60	9.27
22	-8.66	0.63	9.29
23	-8.52	0.56	9.08
24	-8.27	0.59	8.86
25	-8.31	0.72	9.03
26	-8.56	0.82	9.38
27	-8.39	0.71	9.10
28	-8.19	0.94	9.13
29	-8.34	0.99	9.33
30	-7.99	1.13	9.12
